# Adaptive Evolution and Functional Redesign of Core Metabolic Proteins in Snakes

**DOI:** 10.1371/journal.pone.0002201

**Published:** 2008-05-21

**Authors:** Todd A. Castoe, Zhi J. Jiang, Wanjun Gu, Zhengyuan O. Wang, David D. Pollock

**Affiliations:** 1 Department of Biochemistry and Molecular Genetics, University of Colorado School of Medicine, Aurora, Colorado, United States of America; 2 Department of Biological Sciences, Biological Computation and Visualization Center, Louisiana State University, Baton Rouge, Louisiana, United States of America; University of California, Berkeley, United States of America

## Abstract

**Background:**

Adaptive evolutionary episodes in core metabolic proteins are uncommon, and are even more rarely linked to major macroevolutionary shifts.

**Methodology/Principal Findings:**

We conducted extensive molecular evolutionary analyses on snake mitochondrial proteins and discovered multiple lines of evidence suggesting that the proteins at the core of aerobic metabolism in snakes have undergone remarkably large episodic bursts of adaptive change. We show that snake mitochondrial proteins experienced unprecedented levels of positive selection, coevolution, convergence, and reversion at functionally critical residues. We examined Cytochrome C oxidase subunit I (COI) in detail, and show that it experienced extensive modification of normally conserved residues involved in proton transport and delivery of electrons and oxygen. Thus, adaptive changes likely altered the flow of protons and other aspects of function in CO, thereby influencing fundamental characteristics of aerobic metabolism. We refer to these processes as “evolutionary redesign” because of the magnitude of the episodic bursts and the degree to which they affected core functional residues.

**Conclusions/Significance:**

The evolutionary redesign of snake COI coincided with adaptive bursts in other mitochondrial proteins and substantial changes in mitochondrial genome structure. It also generally coincided with or preceded major shifts in ecological niche and the evolution of extensive physiological adaptations related to lung reduction, large prey consumption, and venom evolution. The parallel timing of these major evolutionary events suggests that evolutionary redesign of metabolic and mitochondrial function may be related to, or underlie, the extreme changes in physiological and metabolic efficiency, flexibility, and innovation observed in snake evolution.

## Introduction

When biological molecules were first sequenced and compared, it was expected that innovation and divergence at the morphological level would be explained by molecular innovation and divergence. Compelling evidence for functional evolutionary innovation in proteins is rare [1], however, and often insufficient to substantially explain the uniqueness, diversity, and complexity of species [2], [3]. In contrast, we show here that strong adaptive evolution in snake mitochondrial proteins appears to have led to functional innovation and reorganization. Our research suggests that snake oxidative metabolism is mechanistically and functionally unique among vertebrates, and that snakes are thus an ideal model for developing hypotheses of alternative metabolic function. Evolutionary redesign of these core metabolic proteins, along with alteration of mitochondrial genome structure [4]–[6], may well have contributed to the unique metabolic and physiological adaptations that underlie the evolution and radiation of snakes.

Snakes produce toxic venoms (some highly toxic, with elaborate injection systems) and may consume prey that exceeds their own body mass, leading to massive and rapid change in metabolic demand [7]–[9]. The two main lineages of snakes are ecologically and morphologically distinct: the vast majority of familiar snakes, including the large constrictors and all venomous species, belong to the Alethinophidia (“advanced snakes”), whereas the Scolecophidia (“blind snakes”) are small, subterranean, mostly tropical, and rarely seen. Alethinophidian snakes, particularly pythons, have recently become heavily utilized as a model for extreme physiological and metabolic regulation [10]–[12], as well as a model for metabolic efficiency [13]. To accommodate consumption of large prey at infrequent intervals, snake oxidative metabolism can fluctuate up to 4000% [12], and their organs, including the heart and gut, can enlarge 40–100% within 48 hours [10]–[12].

Alethinophidian snakes also have atypical mitochondrial genomes (mtDNA), with most genes shortened compared to other vertebrates, and with duplicate control regions. The nearly identical control regions are maintained by an unknown mechanism of concerted evolution [4]–[6], and both are predicted to function in most species [5]. Here, we have conducted extensive molecular evolutionary analyses of selection and coevolution in snake mitochondria and evaluated the results in the context of the structure and function of snake mitochondrial proteins. Our results provide multiple lines of evidence that snake mitochondrial proteins have undergone a remarkable and unique process of adaptive evolutionary redesign that involved a strong episodic burst of accelerated amino acid replacement, particularly at residues that are normally highly conserved across vertebrates. In-depth analyses of the evolution of snake COI suggest that the core functions of this protein, including proton transport, electron transport, and oxygen reduction to water, may have been essentially redesigned. This conclusion implies that snake aerobic metabolism is particularly unique among vertebrates, and may partially explain, at the molecular level, the extraordinary physiology and metabolic flexibility of snakes.

## Results and Discussion

Mitochondrial protein-coding genes normally have low rates of non-synonymous change (changes that alter amino acids) compared to synonymous change (*dN*/*dS*), due to strong purifying selection to conserve protein function [14], [15]. Consistent with this, the median *dN*/*dS* ratio (inferred from codon-based selection analyses) for the tetrapod mitochondrial dataset is 0.12, and for cytochrome C oxidase subunit 1 (COI), the most conserved mitochondrial protein, it is 0.02. In contrast, along the branch leading to snakes the *dN*/*dS* for all proteins combined is 25-fold higher (3.14), and is 40-fold higher for COI (0.81). Along the branch leading to the Alethinophidia, these ratios are 14× and 60-fold higher, respectively (1.63 and 1.21). The *dN*/*dS* ratios of Cytochrome b (CytB) are also substantially higher on these branches (snakes = 1.56; Alethinophidia = 2.48) compared to the tetrapod median (0.06). Furthermore, multiple sites in all 13 mitochondrial proteins are inferred to have experienced positive selection early in snake evolution, with the highest number of sites occurring in COI and CytB (Table 1).

**Table 1:** Unique replacement sites and results of branch-site analyses of positive selection for mitochondrial protein-coding genes early in snake evolution.

Gene	Gene Length (codons)	Sites with Unique Replacements	Positively Selected Sites					
			Snake Branch			Alethinophidia Branch		
			Sites PP>90	Sites PP>95	Sites PP>99	Sites PP>90	Sites PP>95	Sites PP>99
**ATP6**	221	3	6	5	3	15	6	4
**ATP8**	45	2	0	0	0	7	5	0
**COI**	509	23	7	3	0	27	12	5
**COII**	226	10	4	3	1	25	15	9
**COIII**	259	9	7	3	2	12	6	4
**CytB**	366	19	9	4	1	36	16	10
**ND1**	313	4	2	1	1	9	2	2
**ND2**	335	5	5	0	0	23	8	4
**ND3**	109	2	0	0	0	9	5	3
**ND4**	443	4	1	0	0	11	5	2
**ND4L**	93	3	1	0	0	6	2	1
**ND5**	619	12	9	3	2	14	8	3
**ND6**	143	1	1	1	1	21	8	2

The number of sites per gene inferred as being under positive selection based on branch-site model analyses in PAML are shown at two posterior probability (PP) significance levels. The branch-site model of positive selection was tested for two main snake lineages: the lineage leading to all snakes (“Snake Branch”) and the lineage leading to the Alethinophidia (“Alethinophidia Branch”); relative support for selection at sites is based on posterior probability (PP) support from Bayes empirical Bayes estimates in PAML. Note: in this table, “sites” refer to amino acid sites.

This evidence appears to suggest that a punctuated burst of positively selected amino acid replacement may have occurred early in snake evolution. In contrast, more recent (terminal) snake branches have typical *dN*/*dS* ratios, indicating that strong purifying selection to conserve protein function subsequently resumed (Figures S1, S2, S3, S4, S5, S6, S7, S8). The accuracy of these ratios may be questioned, however, because these analyses involve comparisons of highly divergent sequences. In such cases, the *dS* component of standard *dN*/*dS* calculations may be underestimated at more ancient phylogenetic depths (or particularly long branches of the tree) due to saturation of synonymous changes. In mitochondrial genomes, the rapid relative rates of transition (purine
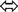
purine or pyrimidine
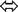
pyrimidine) substitutions make them a particular concern with regard to saturation and *dS* underestimation bias.

Based on these concerns, we treat the traditional codon-based *dN*/*dS* estimates with caution. We suggest that transversion-only *dN*/*dS* comparisons should be more reliable estimators of the relative acceleration of amino acid replacement rates compared to the neutral rate, and can be more reliably used to verify that the high *dN*/*dS* ratios along deep branches in snakes. Because transversion (TV) substitution dynamics in mtDNA are far more consistent than transitions, they substitute at a much slower rate, and are thus much less prone to saturation, the use of exclusively transversions for relative rate comparisons (e.g., *dN*/*dS*) can eliminate many potential errors [5], [15], [16].

**Figure 1:**
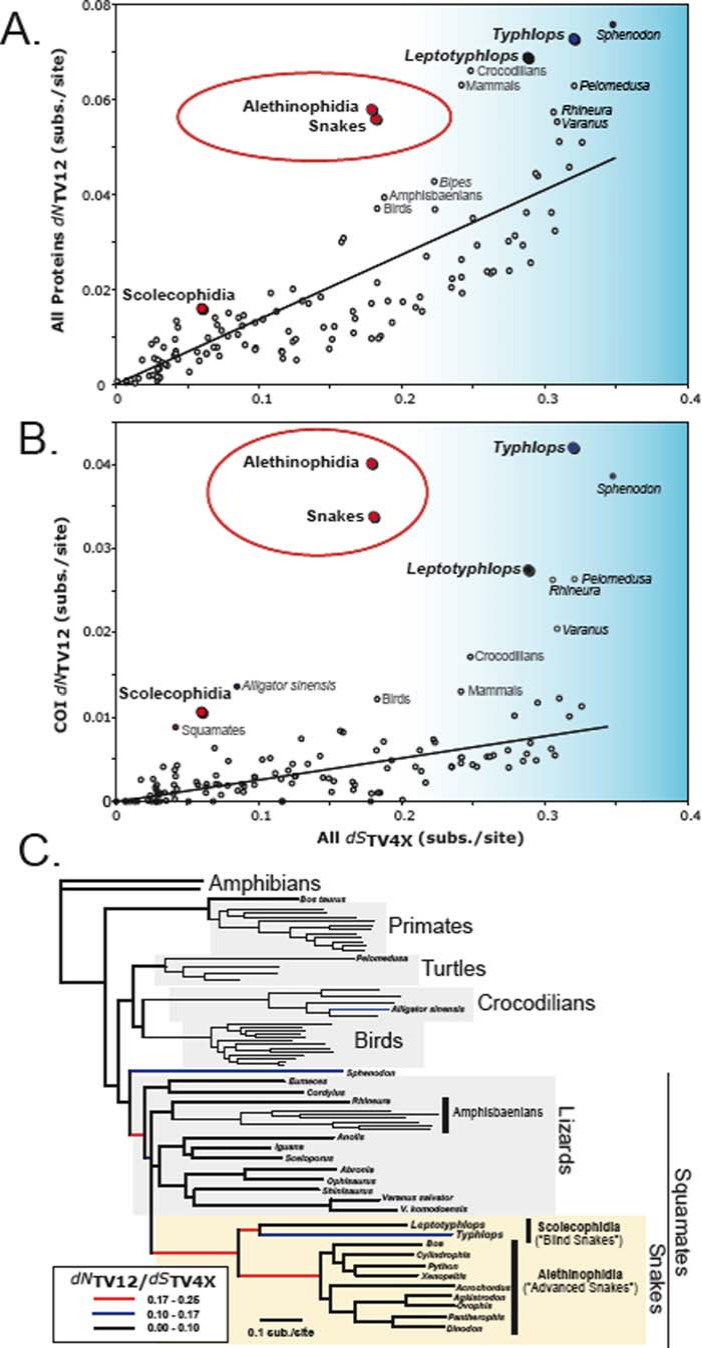
Mitochondrial proteins have experienced extraordinarily elevated rates of amino acid replacement early in the evolution of snakes. The conservative transversion-based approximations of the relative rates of non-synonymous to synonymous substitution (*dN*_TV12_ / *dS*_TV4x_) rates are shown as open or colored circles for each branch of the phylogenetic tree; linear regression lines (excluding points in the red ellipse) are shown in black (A and B). The calculations shown are from (A) all mitochondrial proteins and (B) cytochrome C oxidase subunit 1 (COI). Blue-shaded areas of A and B indicate very long branches with high *dS*_TV4x_ values where the (*dN*_TV12_ / *dS*_TV4x_) estimate may be inaccurate, possibly due to *dS*_TV4x_ saturation and underestimation. Note that early snake branches have very high *dN*_TV12_, far greater than branches of comparable length (*dS*_TV4x_). This is strong evidence for extraordinarily accelerated rates of amino acid replacement early in snake evolution. The phylogenetic tree of relationships among species in our comparative dataset is shown in (C). Branches with extremely high values of *dN*_TV12_ / *dS*_TV4X_ for COI are indicated with colored lines (black, blue, red) following the key in the bottom left. The circles for branches in (A) and (B) were colored according to the same legend for ratios of COI (*dN*_TV12_ / *dS*_TV4x_).

To estimate a transversion-based *dN*/*dS* estimate, the synonymous transversion rate was averaged over all 3^rd^ codon positions in the mtDNA with conserved four-fold redundancy (*dS*_TV4X_; Figure S9), while the non-synonymous transversion rate was measured at first and second codon positions (*dN*_TV12_) for each gene under consideration. Non-synonymous transversions at first and second codon positions result primarily in amino acid replacements with radical physico-chemical differences and major functional effects, but there is a strong correlation between *dN*_TV12_ and overall amino acid replacement rates (r^2^ = 0.95; p<<0.001; Figure S10). The *dN*_TV12_/*dS*_TV4X_ ratios strongly support the finding that mitochondrial proteins endured massive bursts of amino acid replacement early in snake evolution compared to other tetrapod lineages (Figure 1). For all mitochondrial proteins combined, *dN*_TV12_ values at the base of snake origins were about 3-fold greater than expected compared to other tetrapod lineages (Figure 1A), and were most accelerated in COI (Figures 1B–C). For example, along the branch leading to the Alethinophidia, COI experienced 10-fold more *dN*_TV12_ (and thus radical amino acid substitutions) than expected (Figures 1B–1C). As before, high ratios are not maintained in descendant snake lineages (see also Figure S11), indicating that strong purifying selection subsequently dominated snake mitochondrial evolution (Figure 1).

To study the impact of the most functionally relevant amino acid replacements in snake mitochondrial proteins, we considered the “unique sites” with replacements in snakes and otherwise conserved across most tetrapods (see details in Methods). In addition to their high *dN*/*dS* ratios, COI and CytB have the greatest number (23 and 19, respectively, out of 97 total) and concentration of unique sites among mitochondrial proteins (Table 1). Since the function and structure of COI are relatively well understood compared to other mitochondrial proteins [17], [18], unique sites in COI (Table 2) were examined in further detail. Amino acid replacements at the 23 unique COI sites are concentrated in the earliest branches in the snake tree; the three deepest snake branches experienced 25–31 estimated changes at these sites (5–8 along the branches leading to all snakes, 11–15 to the Alethinophidia, and 8 to the Scolecophidia). Nine sites had reversions or multiple replacements, usually leading to parallel or convergent evolution, and about half of these sites (14) underwent substantial changes in polarity or charge (Table 2).

**Table 2:** Unique substitutions and coevolutionary clusters formed by these sites in snake COI.

			Alethinophidian Snakes										Blind Snakes	
Coevolutionary Clusters	Site	Non-snake Tetrapods	*A. piscivorus*	*O. okinavensis*	*P. slowinskii*	*D. semicarinatus*	*A. granulatus*	*B. constrictor*	*C. ruffus*	*P. regius*	*X. unicolor*		*L. dulcis*	*T. reticulatus*
7	**26**	A	S	S	S	S	S	S	S	S	S		A	S
1	**35**	L	I	I	I	I	I	I	I	V	I		L	M
1	**37**	I	M	M	M	M	M	M	M	M	M		I	V
1	**54**	Y	F	F	F	F	Y	F	F	F	F		Y	Y
	**89**	A	T	T	A	A	A	A	A	A	A		A	A
7	**108**	S	A	A	A	A	A	A	A	A	A		A	S
	**174**	P	K	K	K	K	K	A	A	A	P		K	P
8	**194**	L	M	M	M	M	M	M	M	M	M		L	L
5	**205**	G	A	A	A	A	A	A	A	A	A		A	A
5	**231**	Y	F	F	F	F	F	F	F	F	F		Y	F
3	**256**	H	S	S	S	S	S	S	S	S	S		H	H
3	**258**	V	I	I	I	I	I	I	I	I	I		I	I
4	**266**	E	N	N	N	N	N	N	N	N	N		E	E
4	**267**	P	T	T	T	T	T	T	T	T	T		P	P
	**272**	G	S	S	S	S	S	S	S	S	S		S	S
8	**281**	G	A	A	A	A	A	A	A	A	A		A	S
	**286**	I	V	V	V	V	V	V	V	V	V		V	I
6	**299**	V	I	I	I	I	I	I	I	I	I		V	V
6	**301**	T	S	S	S	S	S	S	S	S	S		T	T
	**353**	L	M	M	M	M	M	M	M	M	M		L	L
	**438**	R	R	R	G	R	R	R	R	R	R		R	R
2	**443**	Y	F	F	F	F	F	F	F	F	F		Y	Y
2	**447**	Y	F	F	F	F	Y	F	F	F	F		F	F

Amino acid replacements in snake COI amino acid sequences from the conserved consensus amino acid in tetrapods are shaded in gray. Spatially clustered sites that experience apparent coevolutionary replacements are numbered.

Further support for the role of selection in snake mitochondrial protein evolution comes from evidence for coevolution within the COI protein. We use a method of coevolutionary analysis that measures the significance of the pairwise coevolutionary signal based on the log likelihood ratio of independent versus dependent evolution on a phylogenetic tree [19]. To avoid over-parameterization in these pairwise site analyses, the protein data is recoded into reduced alphabet base on physicochemical characteristics of amino acids at each site (e.g., polarity, volume, or charge). In a previous analysis of 231 vertebrate COI sequences, 11% (p<0.05; 3.7% at p<0.01) of all possible pairs of COI sites were found to be significantly coevolving with respect to polarity [19]. This dataset excluded snakes, and when eleven snake COI sequences were added the coevolutionary signal increased to 59.8% of site pairs (p<0.05; 22.0% at p<0.01) according to polarity. Thus, the coevolutionary signal in snake COI sequences is inordinately strong.

**Figure 2:**
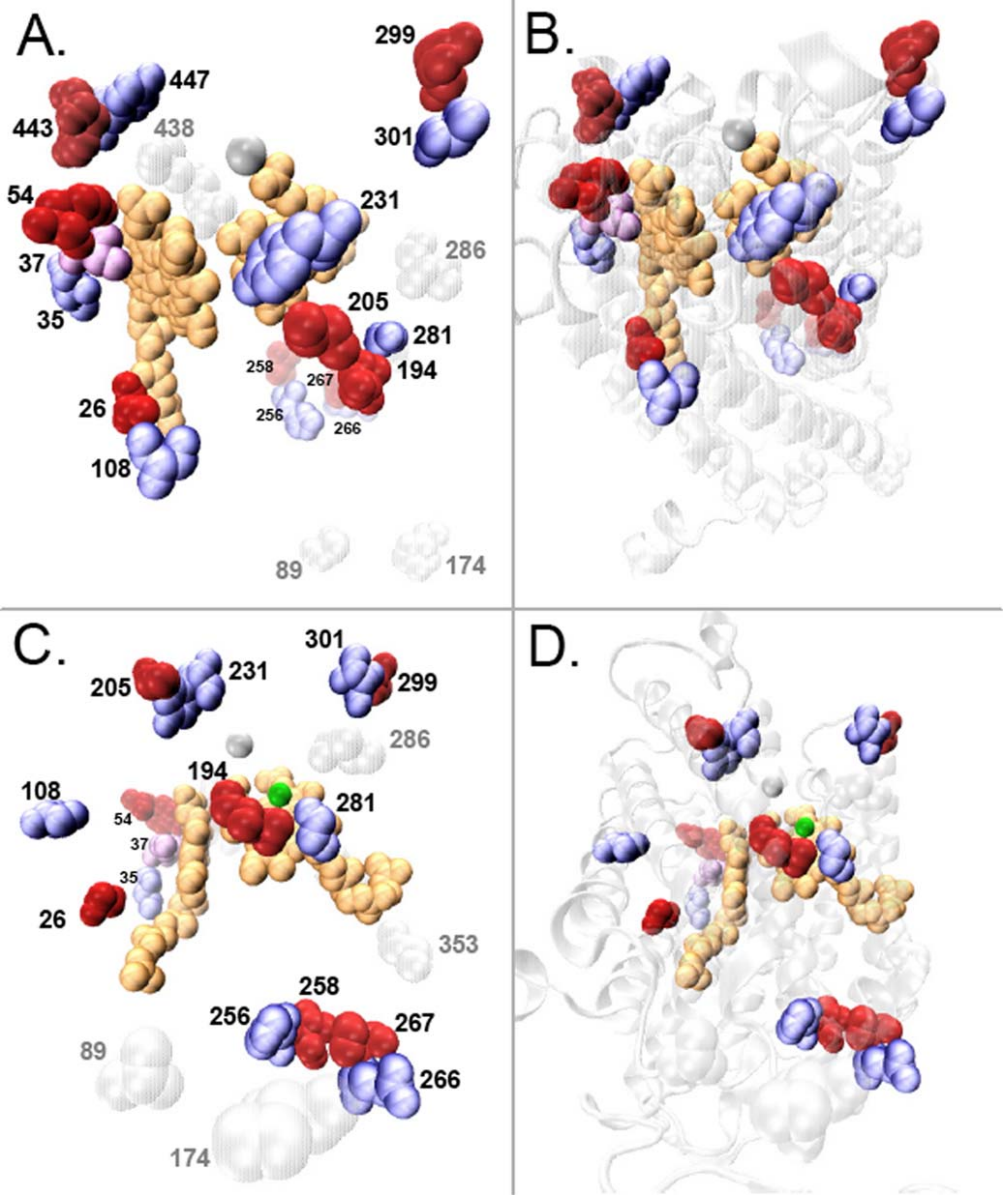
Unique amino acid replacements in Cytochrome C oxidase subunit 1 form tight spatial clusters. The twenty-three unique amino acid replacements in the cytochrome C oxidase subunit 1 (COI) protein of snakes form seven pairs and one triplet of spatially clustered amino acid replacements, concentrated at the core functional region of the COI protein. The seven spatially adjacent pairs of amino acid residues, strongly suggestive of coevolutionary adaptive change, are shown in blue/red paired spacefill combinations, and one triplet cluster is shown in a blue/purple/red combination. Unique sites that did not form clusters are shown in gray spacefill representations. The two heme groups are shown in gold spacefill shapes, the COI backbone in white, and the magnesium and copper atoms are shown as magenta and green balls, respectively. Two different perspectives are depicted, one in A and B, and a second in C and D; Figure sets A/B and C/D are the same views with B and D showing the ribbon structure of the COI backbone in transparent grey.

The 23 unique snake sites show an excessively high degree of coevolution with each other in this analysis: among all possible combinations of unique site pairs, 66% and 89% have significantly coevolved (p<0.05; 28% and 36% at p<0.01) according to polarity and volume, respectively (Table S4). When these 23 unique sites are visualized on the structure of cow CO, seventeen of these 23 unique sites clearly form structurally clustered pairs or triplets, most of which appear to be in physical contact, and these clusters occur primarily in the core functional regions of the COI protein (Figure 2, Table 3).

**Table 3:** Spatial clustering of unique residues in snakes.

Cluster Number	Residues	Cα Distance	Location
1	**35L** – 37I – 54Y	5.0 Å*, 10.6 Å*	H Channel
2	**443Y** – 447Y	6.7 Å*	H Channel
3	**256A** – 258V	5.6 Å*	K Channel
4	**266E** – 267P	3.8 Å*	K Channel
5	26A – 108S	11.9 Å	D Channel
6	205G – 231Y	6.3 Å*	O_2_ Delivery
7	**299V** – **301T**	5.5 Å*	O_2_ Delivery
8	194L – 281G	6.9 Å*	O_2_ Delivery

Sites at which positive selection was detected with greater than 90% posterior probability (Table S3) are shown in bold, as well as associations with functional regions. Distances between residue alpha carbons (Cα) were calculated from the bovine CO structure. All residue pairs marked with a ^*^ have closest side-chain van der Waals contact distances (excluding hydrogen) ≤5.0 Å.

To our knowledge, such a high proportion of physically close (or touching) clusters of replaced residues has not been previously observed in any protein, nor has this degree of concentrated coevolutionary change been previously reported for a protein. The physical clustering of unique sites strongly supports the hypothesis that these sites have coevolved, independent of the statistical coevolution analysis. Therefore, such tight physically paired coevolving residues at otherwise conserved (and therefore presumably functionally critical) sites are unlikely to have occurred without the influence of strong positive selection for evolutionary redesign.

The structural basis of CO function is complex. Oxidative phosphorylation is carried out by five complexes that generate a proton gradient and drive the synthesis of ATP. CO is the penultimate complex in this chain, where the reduction of oxygen is coupled to proton pumping [17], [18]. Of the 13 CO subunits, the three encoded by the mitochondrial genome (I, II, and III; Figure S11) are at the structural and functional core of the complex [17], [18]. A copper atom and two heme groups in COI are critical to the coordinated electron transport, oxygen reduction, and proton pumping function of CO [17], [18]. Protons transported or “pumped” along three putative channels (D, H, and K; Figures S12, S13, S14, S15, S16, S17) from the mitochondrial matrix to the mitochondrial intermembrane space contribute to the proton gradient utilized by the ATP synthase complex to produce ATP, and also facilitate the reduction of oxygen to water [17], [18], [20]–[23]. The functionality of the 10–14 polar amino acids comprising the proton channels is supported by both mutagenesis experiments [20], [24] and bioenergetic analyses [18], and all three channels are highly conserved in most vertebrates [17], [18]. To understand the structural effect of substitutions in snakes, comparisons were made to orthologous positions in the bovine heart CO structure, and all position numbers and distance measurements are based on the cow CO structure [17], [18].

The three core COI proton channels appear to have been extensively redesigned during the evolution of snakes. At least two unique site residues (unique residues) are located in or adjacent to each of three proposed channels (Table 3), and most other unique residues are distributed around these channels (Tables S5, S6, S7, S8, S9, S10; Figures S16 and S17). Remarkably, four unique residues (A26S, L35I, Y54F, and L353M) are directly adjacent to the two heme groups. A fifth unique residue (R438G in *Pantherophis*) is positioned between the heme groups and the Cu_A_; this replacement would substantially alter the functional role of residue 438 in the electron transfer pathway critical for oxygen reduction [25]. Six unique residues (L194M, G205L, G272S, G281A/S, I286V, and T301S) are immediately adjacent to the pathway of oxygen delivery to the heme a_3_/Cu_B_ reaction center [17], [18], including two clustered pairs at the interface of the CO homodimer (Table 3). Unique residues are located in the most structurally and functionally important regions of the COI protein.

**Figure 3:**
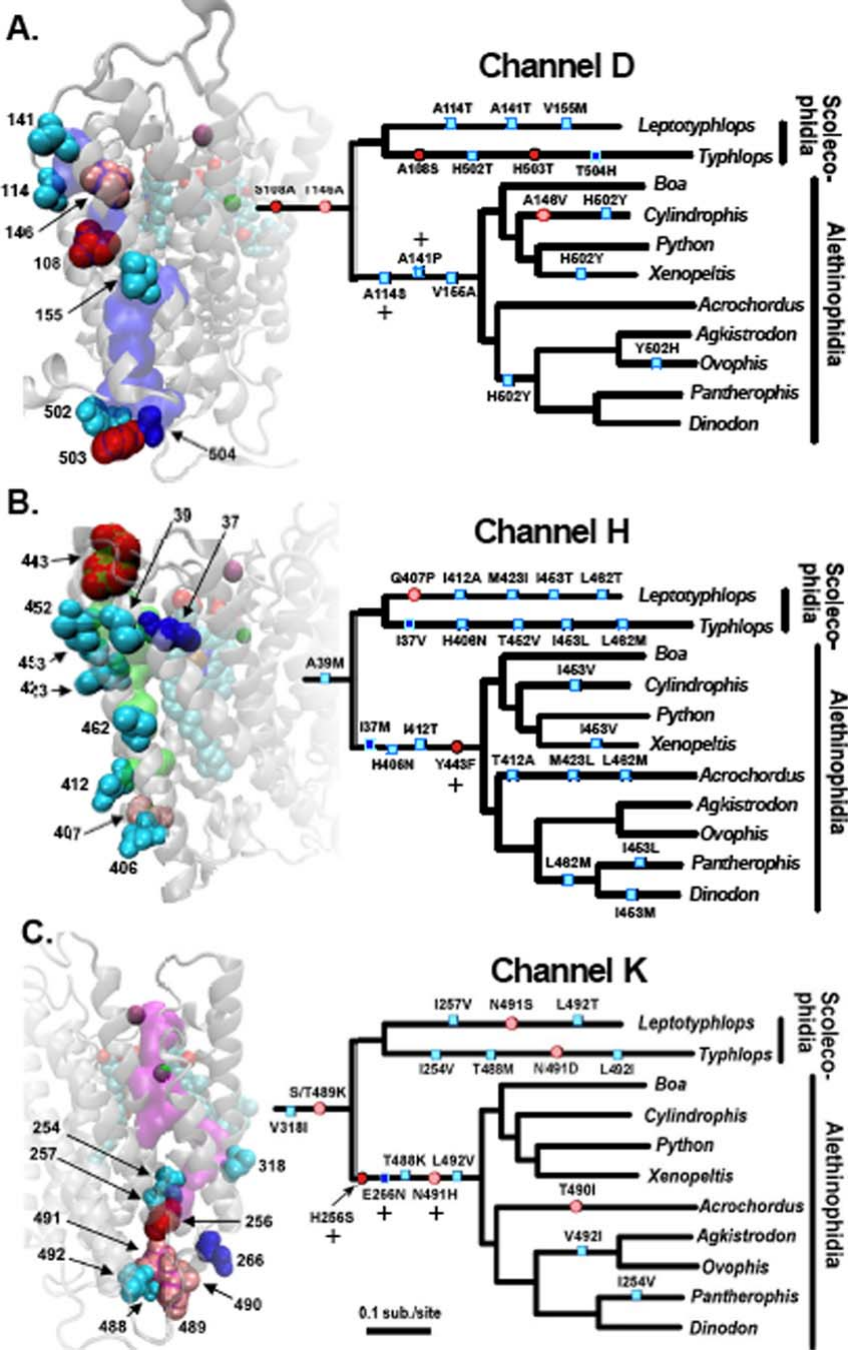
Amino acid replacements at residues within and directly adjacent to the three proton transport channels (D, H, and K) within cytochrome C oxidase subunit 1 (COI) have substantially altered COI structure and presumably function in snakes. On the small snake phylogenetic trees shown, amino acid replacements that occur in snakes at sites comprising the channel are indicated with ovals, and changes at sites adjacent to each channel are indicated with rectangles. Deep colors (dark red and dark blue) indicate replacements at unique sites (see text) whereas lighter colors (light red and light blue) are not unique sites. Sites that were inferred to be under significant positive selection along the Alethinophidian snake branch (by PAML branch-site analyses) are indicated with a plus sign. The relative order of replacements along each single branch has no meaning. These same sites are shown as spacefill representations in the structure, and are labeled and shaded with the same color code as the mark on the tree. One channel is shown per subfigure (A = channel D, B = channel H, and C = channel K). The three proton channels D, H, and K are shown in transparent purple, green, or magenta volumetric representation, respectively. The entire CO1 structure is shown as a transparent grey ribbon structure, heme groups are shown as spacefill representations colored by element, and the magnesium and copper atoms are colored magenta and green, respectively.

To further investigate the functional ramifications of evolutionary change in snake COI, we dissected the details of evolutionary modifications in each channel individually (Figure 3) and inferred the impact of replacements based on existing hypotheses concerning CO function. Proton channel D is thought to be the only channel that is capable of both delivering protons to the reaction center for the reduction of oxygen and pumping protons across the membrane to drive ATP synthesis [18], [22], [23]. Of the 14 residues that comprise the D channel, all but one are completely conserved outside of squamate reptiles (Table S5). Nevertheless, replacements in channel D at the base of snake evolution seem likely to have reduced (or eliminated) its proton transport capacity, based on the functional models of the bovine CO [18], [22], [23]. Replacements S108A and T146A exchanged conserved polar residues with alanine, interrupting an integrated chain of hydrogen bonds critical to proton transfer (Figure 3A; Tables S5–S6). Tsukihara *et al.* [18] suggested that individual substitutions in the proton channels might be tolerated due to contributions from water molecules, but the combined effect of these two replacements alone would be substantial, creating a large polarity gap in the network around intervening residue S149.

Although not immediately adjacent to the bovine channel D, the paired unique replacement A26S (Table 2) may contribute some electron density to the channel in place of the lost S108 (Figure 3A); both these sites appear to have reverted to their original states in the scolecophidian *Typhlops*, along with a clustered set of three replacements at the proton entrance to channel D (Figure 3A). Along the branches leading to the scolecophidian *Leptotyphlops* and to the Alethinophidia, replacements surrounding the above mentioned sites occur in the same three residues (114, 141, and 155); two of these (A114S and A141T) may also supplement some of the electron density lost by S108A and T146A (Figure 3A). We note that there are plausible reconstructions other than those presented in Figure 3A, but all would result in the same extraordinary number of convergent or reversion mutations. In a further surprising demonstration of apparent functional convergence, the amphisbaenian lizard *Rhineura*, a distantly related legless tubular squamate, also has S108A (but not A26S), A114S, and A141P (Tables S5) in common with snakes. Although *Rhineura* has an intact T146, it has a unique S115G replacement likely to similarly reduce proton channel capacity (in combination with S108A). In summary, it appears that amino acid replacements in ancestral snakes at highly conserved sites in channel D are not compatible with this channel's function as predicted in the bovine COI protein. The proton transport capacity of snake channel D may therefore have substantially decreased in the ancestral lineage leading to all snakes, but regained some capacity in all descendant lineages due to multiple reversion, convergent or distinctive replacements, and apparent structural functional convergent changes (Figure 3A).

Channel H is thought to be exclusively involved in pumping protons across the membrane to contribute to the proton concentration gradient [18], [21]–[23], driven by indirect coupling to the reduction reaction. Like channel D, channel H has experienced amino acid replacements early in snake evolution that are incompatible with the putative proton transfer pathway in the cow COI. There is no evidence to suggest, however, that subsequent replacements would have improved channel H capacity. The replacement Y443F along the branch leading to the Alethinophidia is unique, replacing a polar tyrosine residue in channel H with a non-polar phenylalanine (Figure 3B; Table S7); site-specific selection analyses also suggest strong positive selection associated with this substitution (Figure 3B; Table S3). Tyrosine 443 is thought to block the backflow of protons from the channel H exit, functioning as a proton flow regulator depending on the state of hydrogen bonding with residue N451 [18], [20]–[22]; a phenylalanine at site 443 would not fill this role. In addition, H413Q, which occurred in an ancestral squamate lineage, may also reduce channel H capacity due to the ∼9 Å gaps separating site 413 from other sites in the channel (Figure 3B). Another channel H replacement, Q407P, occurs only in *Leptotyphlops* and may disrupt the capacity of the channel (Figure 3B). There was a remarkable amount of convergent and reversionary changes of non-polar amino acid replacement among snakes in and around channel H (Figure 3B; Tables S7–S8); I453, for example, apparently experienced six replacements of isoleucine in different snake lineages (Figure 3B).

Evidence for the utilization of channel H is controversial, and its functionality has been supported only in vertebrates [21], [22]. A recently proposed alternative routing of channel H is slightly closer to the COI interior [21], [22], but this alternate conduit also appears to be eliminated by a unique tyrosine to phenylalanine replacement on the branch leading to the Alethinophidia (Y54F; Figure S17). Two other unique residues with non-polar replacements, L35I and I37M, are in contact with residue 54 (Figure S17, see also Figure 3B). A further unique residue on the branch leading to all snakes occurs near the exit of the alternative channel H (Y447F); a total of three unique tyrosine to phenylanaline replacements clustered around both proposed channel H exits (Figures 3B and S17) appear to eliminate any potential for proton pumping functionality in either proposed H channel, especially in the Alethinophidia. The thorough apparent disruption of this channel in snakes supports previous hypotheses that most vertebrates utilize the H channel [21], [22], while suggesting that snakes have reduced (or eliminated) channel H proton transfer capacity, thereby focusing proton-pumping capacity on channel D.

Channel K is closer to the COI reaction center (heme a3/Cu_B_) than the other proton channels, has more positively charged residues, and is thought to deliver protons exclusively for the reduction of oxygen to water and to not pump protons for ATP synthesis [18], [21]–[23]. Unlike the other two channels, there is no evidence consistent with channel K proton transfer capacity being decreased in snakes. On the contrary, increases in polarity and charge, and multiple lines of evidence for positive selection and coevolution are more compatible with the hypothesis that its capacity was selectively enhanced. Two unique replacements (E266N and H256S) occurred in the Alethinophidia, both of which appear to have evolved under significant positive selection (Figure 3C; Tables S3). Three other replacements (S/T489K on the branch leading to snakes and N491H and T488K on the branch leading to the Alethinophidia) radically altered the physicochemical environment by adding positive charge density at the proton entrance to the channel. Incredibly, two positively charged residues are also added at the entrance to this channel in *Rhineura*, but at a different combination of sites (S/T489H and T490K, along with N491G in the same region; Tables S10, S13). The precise functional effects of these changes are unclear, however this use of positively-charged amino acids at sites 488–491 (along with coevolved structural adjustments) could conceivably improve the water hydrogen-bonding network and thus the proton flux from the mitochondrial matrix into the channel, or modify the regulation of proton flow. Interestingly, sites 491 and 492 both also change but to different amino acids in both branches of the Scolecophidia; additionally site 491 changes in *Leptotyhplops* (Figure 3C). The proton conducting capacity of channel K appears to have been either maintained or possibly improved in various snake lineages, particularly in the Alethinophidia. Remarkably, similar and otherwise extremely rare replacements in the amphisbaenian lizard *Rhineura* recapitulate the apparent functional convergence between this lizard and snakes observed in the D channel.

Adaptive evolution and coevolution in COI early in snake evolution appear to have redesigned core functions. In particular, the roles of the various amino acid residues and channels in proton transport, coupling of proton transport to oxygen reduction, and regulation of these processes appear to have been reorganized. Although the structural and functional evidence is best in COI, there is also compelling evidence for adaptive evolution in other mitochondrial proteins early in snake evolution. Evidence from the distribution and number of unique amino acid replacements (Tables 1), the elevated *dN*/*dS* for the entire mitochondrial proteome (Figure 1A, Figure S3), site-specific selection analyses (Table 2), as well as nucleotide dynamics [5] collectively suggest that most snake mitochondrial proteins have experienced extraordinary levels of functional adaptive change, similar to those we have documented in COI early in snake evolution. Snake mitochondrial function and oxidative metabolism appear to be exceptional system-wide, implying that snakes are an excellent model system for further metabolic research.

Is there anything about snakes that makes them predisposed to adaptive pressure on metabolic function? The evolutionary origin of snakes involved extensive morphological and physiological adaptations to a subterranean (fossorial) lifestyle, including limb loss, the functional loss of one lung, and trunk and lung elongation [8], [26]; these adaptations have also occurred in *Rhineura*. Shared aspects of ecology and morphology between *Rhineura* and early snakes, together with observations that some extremely rare replacements at core functional residues in COI occurred in apparently functionally convergent patterns in both lineages, may indicate a causal link between some organismal and molecular patterns of convergence. This, along with evidence for less extensive adaptive bursts in the mitochondrial proteins of primates and other vertebrates [27]–[31], suggests that adaptation of core mitochondrial proteins may be a key component of major macroevolutionary shifts in organismal physiology and life history.

At the base of the Alethinophidia there was a second functionally dramatic adaptive shift. This lineage came to live above ground and evolved a suite of radical adaptations that center on the consumption of extremely large prey relative to their body size. These include substantial increases in body size and musculature, a highly kinetic skull, and the evolution of a diversity of highly toxic venom proteins [7], [8], [32]. They also evolved the ability to drastically remodel their organs and physiology [10], [11] while enduring metabolic and oxygen consumption rate fluctuations that are among the most extreme known in vertebrates [12]. These traits were later enhanced to various degrees in different descendant lineages, and the particularly successful “advanced snakes” diversified and radiated into one of the most speciose groups of vertebrates. It is, therefore, not entirely surprising that snakes appear to have experienced the most extreme adaptive evolutionary event yet observed in vertebrate mitochondrial proteins.

Adaptations for metabolic efficiency, extreme metabolic and physiological regulation, and extensive fluctuations in oxidative metabolism have made alethinophidian snakes an important model for physiological and metabolic research [10]–[13], and the adaptation of COI seems certain to be related in some way to their unique metabolism and physiology. By apparently changing the functional roles of the three proton channels, as well as altering the flow of oxygen and electrons, snakes may have reorganized the coupling and regulation of the reduction and proton pumping functions of CO. Our data strongly suggest the hypothesis that COI, and possibly other mitochondrial proteins and aerobic metabolism on the whole, have been evolutionary reorganized in snakes, and further biochemical or biophysical work should be done to verify this and to understand the precise functional nature of this putative process of adaptive reorganization. Despite intense study, many crucial details concerning the mechanism of this coupled reaction are still largely unknown and disputed [21], [22], primarily because of the difficulty of experimentally manipulating the large membrane-imbedded CO polypeptide. Snake CO may represent an invaluable evolutionary resource as a hypothesis-generating model of alternative CO function, and thus provide new insight into the poorly known and debated features of vertebrate CO function.

The evidence for uniquely strong and broad-scale adaptive shifts in snake metabolic proteins, together with previous evidence for major adaptive shifts in snake metabolism [11], [12], venom genes [32]–[34], mitochondrial genome architecture [5], physiology [10], [11], and morphology [7], [8], suggest that adaptive evolution and functional innovation may broadly characterize snake evolution. The identification of other components of snake genomes that demonstrate coordinated adaptive phenomena would provide intriguing insight into the coevolution and function of vertebrate metabolism, physiology, and ecology, and may yield unparalleled insight uniting the processes of microevolution and macroevolution.

## Materials and Methods

### Mitochondrial genome sequencing

The mitochondrial genomes of the blind snake *Typhlops reticulatus* and the lizards *Anolis carolinensis*, *Ophisaurus attenuatus*, and *Varanus salvator* were sequenced for this study (Table S1.1) to increase sampling at the base of snake evolution. For each species, DNA was extracted from frozen (80°C) liver tissue using a High Pure PCR Template Preparation Kit (Roche). Two 500 bp fragments, located in the 12sRNA/16sRNA and COIII genes respectively, were amplified using degenerate primers (Table S.2) [35]. New specific primers targeted to these two sequenced regions were then designed for each species. The whole mitochondrial genome of each species was amplified in two overlapping pieces, approximately 8 kb and 9 kb, each via species-specific primers (Table S1.3). Using the Roche Expand Long Template PCR kit, the 9 kb fragment was amplified. These two long PCR products were purified via agarose gel electrophoresis followed by adding GELase enzyme to excised fragments. Following a primer walking strategy, several internal fragments were amplified from each long PCR product and directly sequenced with internal primers. In addition to direct sequencing of PCR products, the control regions of *T. reticulatus*, *P. regius*, and *V. salvator* were cloned into the TOPO XL vector (Invitrogen) and sequenced. DNA sequencing reactions were performed using the ABI BigDye sequencing system. Sequencing reactions were purified on DyeEx columns (Qiagen), and the DNA sequence was determined using an ABI 3700 automated sequencer.

### Mitochondrial genome annotation

Most tRNAs in the raw genome sequences were detected using tRNAscan [36], followed by manual verification. The tRNAs not identified by tRNAscan were identified by their position in the genome and folded manually based on homology. The tRNAs were then used to identify approximate boundaries of protein coding genes, control region, and ribosomal RNAs. Final boundaries of protein coding genes were set based on position of the most plausible first start and last stop codons in each region, including non-canonical signal codons known to exist in vertebrate mitochondrial genomes [37]. Proteins were translated to their amino acid sequence, and all amino acid and DNA sequences were compared to the corresponding genes or regions from published mitochondrial genomes to verify the annotation.

### Alignment

In combination with the new snake and lizard mtDNAs, the mitochondrial genome sequence dataset included a broad diversity of available snake and lizard mitochondrial genomes (Table S2; full alignment shown in Figure S18) see online supplementary appendix of the alignment). In addition to squamate sampling, we included mitochondrial genomes from a diversity of tetrapod species for comparative purposes; this included heavy sampling of birds, mammals (mostly primates), crocodilians, and amphibian outgroups (Table S2). We limited our sampling of mammalian mtDNAs almost exclusively to primates (and *Bos taurus*) because we were particularly interested in obtaining precise comparative estimates of mutation rates and selection pressures that may otherwise become unreliable when sampling is overly sparse. Also, focused sampling of primates was incorporated to keep the total number of sequences low enough to facilitate complex likelihood analyses (which would otherwise be computationally unfeasible), and to facilitate comparisons in rates and patterns of selection between snakes and primates, e.g. [16]. We included *Bos* as a critical reference sequence because the crystal structure of the *Bos* CO protein is well studied [17], [18], [22]. Sequences of all 13 mitochondrial protein-coding genes were aligned using ClustalX [38], followed by manual adjustment. Protein-coding genes were first aligned at the amino acid level, and then the nucleotide sequences were aligned according to the corresponding amino acid alignment.

### Mitochondrial genome phylogenetic analyses

The phylogeny among all included tetrapod mitochondrial genomes was inferred using the concatenated nucleotide sequence of all 13 protein-coding genes by Bayesian Marcov-chain Monte Carlo analyses, conducted in MrBayes v3.1 [39]. This analysis incorporated a complex partitioned model of nucleotide substitution that allowed 39 independent partition-specific models to be used to jointly estimate the phylogeny. Each codon position per protein-coding gene was assigned its own partition and partition-specific models of evolution were determined using MrModeltest (http://people.scs.fsu.edu/nylander/) using AIC. In these partitioned Bayesian phylogeny analyses, all model parameters were unlinked and partition-specific evolutionary rates were allowed to vary.

Three independent runs of MrBayes, incorporating the above partitioned model were run to confirm convergence. Analyses were conducted with default settings for prior distributions, and each was run for 7×10^6^ generations. The burn-in period was determined by visual assessment of stationarity, the average standard deviation of bipartition probabilities among runs, convergence of likelihood values between runs, and apparent stationarity of model parameter estimates. Although burn-in appeared to occur by approximately 2×10^6^, we conservatively used the last 3×10^6^ generations of each run to estimate the posterior distribution for the phylogeny.

### Mitochondrial plus nuclear gene estimates of squamate phylogeny

**P**hylogenetic analyses based exclusively on mitochondrial genes inferred relationships among squamates that represented a substantial departure from recent estimates based on nuclear gene sequences [32], [40], [41]. Given this observation, and the extreme nucleotide dynamics already identified at the base of snake phylogeny [5] and the substantial convergence noted in this study between snakes and other squamate groups (both or which may substantially perturb phylogeny estimates), we separately estimated the phylogeny among included squamate taxa using a combined mitochondrial and nuclear gene dataset. We assembled a nucleotide dataset including the 13 protein-coding mitochondrial genes from all the squamate species included (plus a small number of representative tetrapod outgroups), together with sequences of two nuclear gene sequences obtained from Genbank: c-mos (oocyte maturation factor) and Rag-1 (recombinase activating gene 1). Genbank accession numbers and associated data for the nuclear gene data set are provided in Table S3. This combined nuclear and mitochondrial gene dataset was analyzed in MrBayes similar to the larger mitochondrial dataset above with partition-specific models for all codon positions of each protein-coding gene being allocated an independent model according to AIC criteria. This analysis was also conducted in triplicate for 5×10^6^ generations per run. Stationarity was apparently reached by approximately 10^6^ generations, and the last 3×10^6^ generations from each independent run were (conservatively) used to assemble the phylogeny posterior distribution.

### Combining topologies for the tetrapod tree

Our estimates of relationships among squamates were different based on the phylogeny estimates from the two datasets described above. Also, our phylogeny estimate for relationships among squamate species based on the combined nuclear and mitochondrial gene analyses were most consistent with other recent estimates of squamate phylogeny [32], [40], [41]. To take advantage of the best available comparative framework, we merged these two topology estimates by constraining the more extensive mitochondrial genome-based estimate of tetrapod relationships to reflect the relationships among squamate species inferred from the nuclear plus mitochondrial gene analyses. This topology was used throughout the study as the primary basis of phylogeny-based analyses, although we do present alternative analytical results based on the mitochondrial gene topology. It is noteworthy that since a majority of important evolutionary changes we focus on occur uniquely in the common ancestral lineage leading to snakes, or within the snake tree, the resolution of the relationships outside of snakes has very little bearing on our core analytical inferences.

### Traditional estimation of non-synonymous to synonymous rates along branches

We used two methods to estimate the ratio of non-synonymous to synonymous rates along branches. The first is based on fitting of a codon model of evolution, together with a GTR matrix of nucleotide rates within a maximum likelihood framework, in the program HyPhy [42]. In Hyphy, we specified the topology and conducted a standard codon model of selection analysis (using recommended settings). We used the standard codon selection analysis that performs the codon-based positive selection tests using the models of [43], under the packaged batch file *dNdS Rate Analysis.bf*. This codon model analysis jointly optimizes rate parameters and branch lengths using the MG94xREV model, a combination of the MG94 model [44] with 5 additional parameters for the time reversible nucleotide substitution process (i.e. the GTR matrix). We used the lineage dual variable rates model in which *dS* and *dN* are drawn from a bivariate normal distribution (with independent components), plus each lineage has an adjustment factor for the E[dN]/E[dS]). Independent general discrete distributions were initiated with random starting parameters and 4 rate classes each for synonymous and non-synonymous rates. Multiple runs of these analyses were conducted to confirm the convergence of independent estimates, and each run produced essentially identical results. To confirm the qualitative conclusions under alternative estimates of phylogeny, we used the primary merged topology (Figure S3), in addition to the alternative mitochondrial gene-based topology (Figure S1) as input for HyPhy *dN*/*dS* analyses.

### Transversion-based estimates of protein evolutionary rates

To obtain estimates of synonymous and non-synonymous rates of change that were independent of transition substitution biases, we developed a Markov-chain Monte Carlo method to estimate the number of different types (e.g., synonymous or non-synonymous) of transversion substitutions along each branch of the phylogenetic tree (in Figure S3). We used this method to estimate: the number of transversions at 4-fold redundant 3^rd^ codon positions (*dS*_TV4X_) indicating a neutral rate of transversions, and the number of transversions at 1^st^ and 2^nd^ codon positions (*dN*_TV12_) indicating the number of transversions that result in a change in the encoded amino acid. Transversions at the first two codon positions, and especially at the 2^nd^ codon position, result in predominantly radical amino acid replacements compared to transition substitutions at these positions. For this reason, the *dN*_TV12_ estimate provides a conservative indicator of the non-synonymous rate that is heavily influenced by amino acid replacements that result in radical changes in the physio-chemical attributes of residues.

### Estimation of amino acid replacements per branch

To estimate the correlation between our transversion-based estimates of non-synonymous change (*dN*_TV12_) with rates of amino acid replacement, we estimated the number of amino acid replacements per branch in the program PAML [45] using the *codeml* module, using the fixed topology from Figure S3. This analysis provides a maximum likelihood estimate of branch lengths along this specified topology based on the estimated number of amino acid replacements per site.

### Definition and identification of “unique sites”

The “unique sites” were defined as those that were completely conserved, except in alethinophidian snakes, in our initial tetrapod data set. Subsequently, several newly available mitochondrial genomes were added: the amphisbaenian lizards (including *Rhineura*), *Anolis*, *Shinosaurus*, *Crocodylus*, and *Varanus* species. Although these are highly conserved sites, it is expected that as more taxa are added the “unique” status of some sites that change in snakes may change, as noted for amphisbaenian lizards.

### Structural modeling and identification of COI proton channels

The crystallized Bovine heart CO protein (2OCC.pdb) from *Bos taurus* [18] was used in structural mapping of amino acid replacements in CO. Throughout discussion of COI evolution and structure, we refer to positions based on the *Bos taurus* COI sequence used to estimate the crystal structure of CO [17], [18]. Based on this *Bos* sequence and structure the three putative proton channels within COI following [17], [18]. The alternative H-channel pathway discussed in the text is based on [21].

### Phylogenetic mapping of amino acid substitutions

MacClade v.4 was used to estimate where on the tree particular amino acid changes had occurred (i.e., on what branch). In some cases either the branch or the exact amino acids being exchanged were ambiguous, and these instances are indicated where relevant.

### Site-specific detection of selection

To identify specific sites in COI that appear to have been under notable amounts of site-specific selection for amino acid replacements, we used the branch-site model (referred to in the PAML documentation as ‘the modified Model A’, also M2a) in PAML v.3.15 [45], [46]. We used this approach to detect selective pressures on sites of the COI gene specifically along the branch leading to all snakes and the branch leading to the Alethinophidia, since these two branches appear to have outstanding numbers of unique substitutions and extreme ratios of *dN*/*dS*. Specifically, we tested the significance of allowing some *dN*/*dS* ratios (ω) at sites and branches to be >1. To test this, we constructed “test 2” which is a likelihood ratio test (with degrees of freedom equal to ∼1) comparing model M2a with ω_2_ = 1 (null) versus the same model with ω_2_≥1 to be estimated by the data for the branch of interest. The selection detection process was repeated three times to avoid local minima as suggested by the author. Details of this analysis are given in the online supplement (Supplementary Text S1).

### Analyses of coevolution

Coevolution was analyzed in a likelihood-based framework using LnLCorr [47]. To increase detection power, residues at each site were segregated into two groups (states) with respect to their hydrophobicity, polarity, or side chain volume. This method is described and justified in detail elsewhere [19], [47]. In brief, it is a tree-based likelihood method that tests all possible pairs of residues within a protein for evidence of highly correlated amino acid replacement. The dataset and methods for coevolutionary analyses are essentially identical to [19], and here we report only the salient points of the methodology and the differences between this analysis and previous analyses. This also allows direct comparison of analysis of the same dataset with and without snake mitochondrial genomes included.

In a previous study [19] we had analyzed coevolutionary interactions using this approach across a large collection of vertebrate COI sequences (N = 231), but excluded snakes because preliminary analyses suggested snake mitochondrial proteins exhibited excessive coevolutionary signal compared to the rest of the vertebrates. Here, we essentially repeated that analysis but with the eleven snake COI sequences included, and compare the coevolutionary signal detected by this analysis to demonstrate the relative increase in coevolutionary signal attributable to the addition of the snake proteins. The final mitochondrial protein dataset used for coevolutionary analyses here contained 242 species of vertebrates (with the snakes included), ranging from cartilaginous fishes to tetrapods (including snakes), see [19]. The crystal structure of bovine heart mitochondrial CO (PDB ID: 2OCC) [18] was obtained from the Protein Data Bank and used to estimate structural distances among residues among COI residues. The distance between two residues of a pair was defined as the distance between their Cá atoms in the monomer unit of the bovine crystal structure.

Two alternative models of evolutionary amino acid replacement, an independent (null) model and a dependent (alternative coevolution) model are compared. The dependent (coevolution) model has one more parameter than the independent (null) model, and the independent model is nested within the dependent model. The likelihoods were calculated via a pruning algorithm, and an MCMC algorithm was implemented to traverse the parameter surfaces and locate the maxima. We conducted 10,000 iterations for each chain, although equilibrium was reached in less than 2000 iterations. A likelihood ratio statistic was used to evaluate the significance of coevolution between sites. It has been previously shown that this statistic cannot be assumed to have a χ^2^ distribution under the null (independent) model [47]. Therefore, to obtain more accurate distribution estimators, we performed parametric bootstrapping by simulating datasets using the same phylogenetic tree and the maximum likelihood parameter estimates (MLEs) from the independent model [47].

Since thousands of comparisons were performed in each analysis, these probability values are not good estimators of the probability that each pair of sites coevolved (i.e., multiple comparisons issues have to be addressed). We therefore considered the pairs with LRs beyond the 5% probability cutoff to be a set of hypothetical coevolving pairs, and compared the observed percentage of coevolving pairs (the “coevolving percentage”) to the percentage of false “coevolving pairs” that would have been expected even if no coevolution had actually occurred, as in [19] and [47].

## Supporting Information

Figure S1 The phylogeny estimated from all 13 protein-coding genes from of all 65 mitochondrial genomes used in this study (Table S2), estimated using Bayesian partitioned model analyses (with 39 partitions).
(0.08 MB PDF)

Figure S2 Phylogeny based on the combined mitochondrial and nuclear (c-mos and Rag-1) gene alignment estimated using partitioned-model Bayesian analyses.
(0.08 MB PDF)

Figure S3 Results of traditional dN/dS estimates for the entire set of 13 mitochondrial protein-coding genes with branches colored based on dN/dS ratios.
(0.09 MB PDF)

Figure S4 Results of traditional dN/dS estimates for the COI gene using the Figure S3 phylogeny with branches colored based on dN/dS ratios.
(0.08 MB PDF)

Figure S5 Results of traditional dN/dS estimates for the CytB gene using the Figure S3 phylogeny with branches colored based on dN/dS ratios.
(0.08 MB PDF)

Figure S6 Results of traditional dN/dS estimates for all 13 protein-coding mitochondrial genes using the alternative (mitochondrial gene-based) topology from Figure S1, with branches colored based on dN/dS ratios.
(0.09 MB PDF)

Figure S7 Results of traditional dN/dS estimates for the COI gene using the alternative (mitochondrial gene-based) topology from Figure S1, with branches colored based on dN/dS ratios.
(0.09 MB PDF)

Figure S8 Results of traditional dN/dS estimates for the CytB gene using the alternative (mitochondrial gene-based) topology from Figure S1, with branches colored based on dN/dS ratios.
(0.09 MB PDF)

Figure S9 Correlation between the number of transversion substitutions at 4-fold redundant sites (TV4X) in all mitochondrial proteins versus dSTV4X COI.
(0.06 MB PDF)

Figure S10 Estimated amino acid replacements (proportion of replacements per sequence) versus transversion substitutions at 1st and 2nd codon positions (dNTV12).
(0.07 MB PDF)

Figure S11 Conservative transversion-based approximations of the relative rates of non-synonymous to synonymous substitution (dNTV12 / dSTV4x) rates for the mitochondrial Cytochrome B (CytB) gene suggest that rates of amino acid replacement along br
(0.12 MB PDF)

Figure S12 Three dimensional view of the ribbon structure of the 13 subunits of the monomer of CO.
(0.39 MB PDF)

Figure S13 Two different three dimensional views of the ribbon structure of cytochrome C oxidase subunit I (COI) with major functional regions and features illustrated.
(1.13 MB PDF)

Figure S14 The three proposed proton transfer channels in COI.
(0.50 MB PDF)

Figure S15 Three-dimensional views of the three proton channels within the ribbon structure of cytochrome C oxidase subunit 1 (COI; based on the cow COI structure).
(0.96 MB PDF)

Figure S16 Locations of unique substitutions projected over the location of the reaction center and proton transfer channels.
(0.67 MB PDF)

Figure S17 Three-dimensional views of the alternative pathways of proton channel H within the ribbon structure of cytochrome C oxidase subunit 1 (COI; based on the cow COI structure).
(1.24 MB PDF)

Figure S18 Nucleotide Alignment
(7.64 MB PDF)

Table S1 Laboratory and specimen voucher information for mitochondrial genomes sequenced in this study.
(0.09 MB PDF)

Table S2 Complete mitochondrial genomes used in this study, and associated Genbank accession numbers.
(0.08 MB PDF)

Table S3 Sites that have undergone positive selection in COI along the branches leading to all snakes and that leading to the Alethinophidia, based on the branch-site model of PAML.
(0.06 MB PDF)

Table S4 Results of likelihood-based analysis of coevolution among protein residues within the COI protein.
(0.09 MB PDF)

Table S5 Conservation of residues in proton transfer channel D across the 65 taxon dataset used.
(0.08 MB PDF)

Table S6 Conservation of residues in proton transfer channel H across the 65 taxon dataset used.
(0.08 MB PDF)

Table S7 Conservation of residues in proton transfer channel K across the 65 taxon dataset used.
(0.08 MB PDF)

Table S8 Conservation of residues surrounding channel D across the 65 taxon dataset used.
(0.13 MB PDF)

Table S9 Conservation of residues surrounding channel H across the 65 taxon dataset used.
(0.13 MB PDF)

Table S10 Conservation of residues surrounding channel K across the 65 taxon dataset used.
(0.13 MB PDF)

Text S1 Detailed results of branch-site analyses of positive selection.
(0.05 MB PDF)
