# Supplementary material for: Adaptive Evolution and Functional Redesign of Core Metabolic Proteins in Snakes
Source: PLoS One. 2008 May 21;3(5):e2201. doi: 10.1371/journal.pone.0002201 (PMC2376058; doi:10.1371/journal.pone.0002201)
Supplement: Figure S1 — The phylogeny estimated from all 13 protein-coding genes from of all 65 mitochondrial genomes used in this study (Table S2), estimated using Bayesian partitioned model analyses (with 39 partitions). (0.08 MB PDF) [file pone.0002201.s001.pdf]

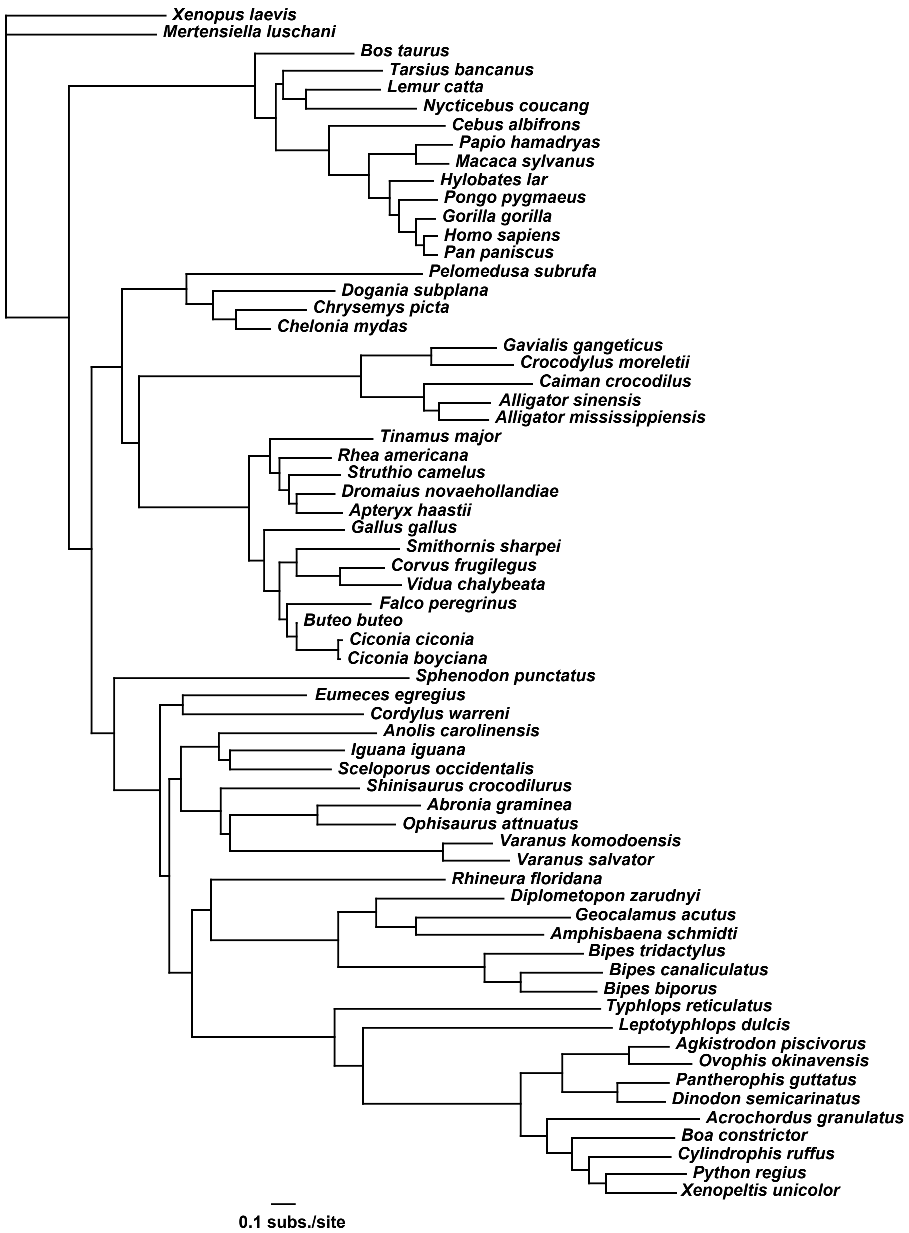

Figure S1. The phylogeny estimated from all 13 protein-coding genes from of all 65 mitochondrial genomes used in this study (Table S2), estimated using Bayesian mixed partitioned analyses (with 39 partitions). ***NOTE:*** All nodes received 100% Bayesian posterior probability support.
