# Supplementary material for: Adaptive Evolution and Functional Redesign of Core Metabolic Proteins in Snakes
Source: PLoS One. 2008 May 21;3(5):e2201. doi: 10.1371/journal.pone.0002201 (PMC2376058; doi:10.1371/journal.pone.0002201)
Supplement: Figure S2 — Phylogeny based on the combined mitochondrial and nuclear (c-mos and Rag-1) gene alignment estimated using partitioned-model Bayesian analyses. (0.08 MB PDF) [file pone.0002201.s002.pdf]

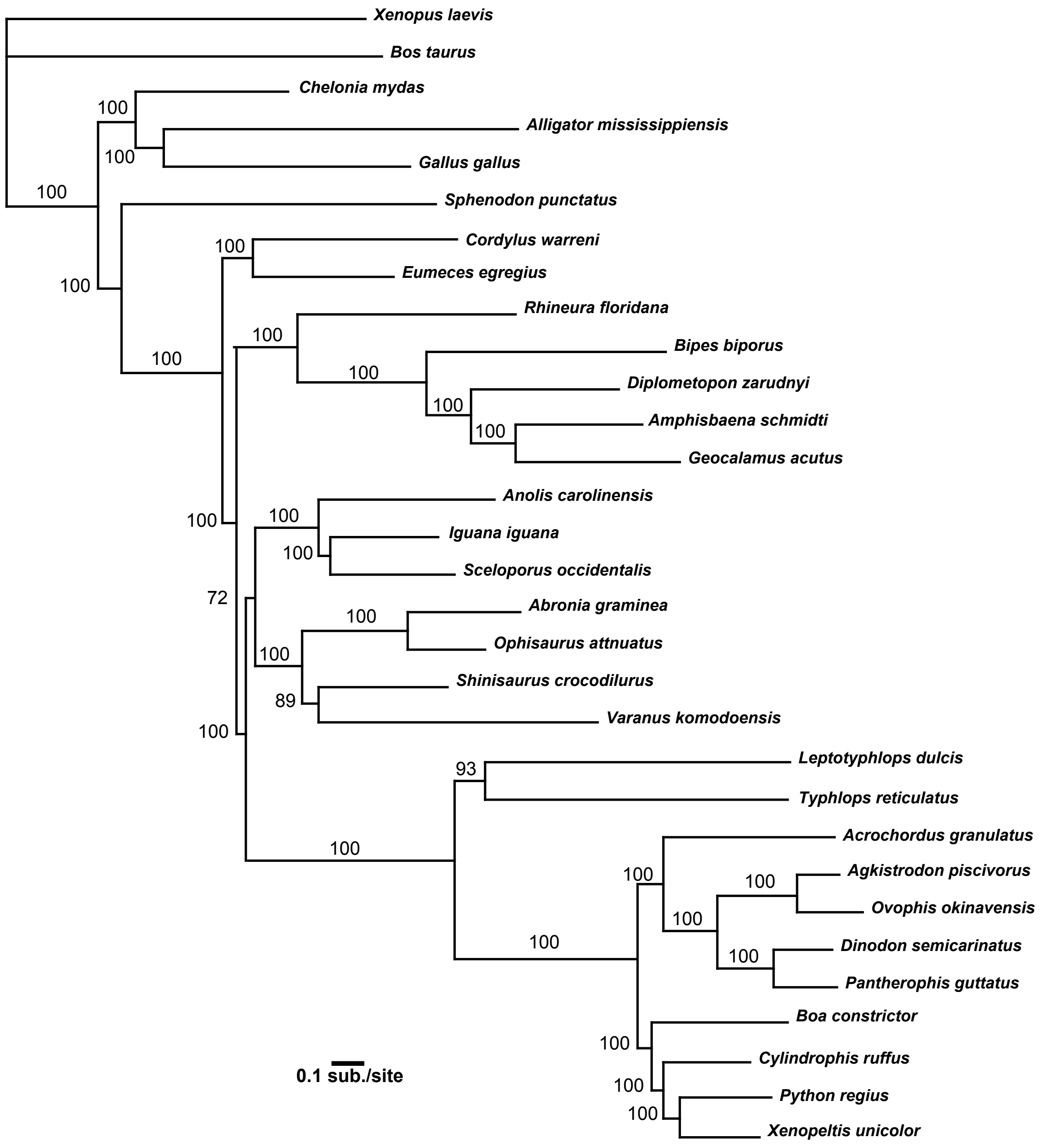

Figure S2. Phylogeny based on the combined mitochondrial and nuclear (c-mos and Rag-1) gene alignment estimated using partitioned-model Bayesian analyses. Bayesian posterior probability values for nodal support are provided above or adjacent to each node. Taxon sampling for this dataset is a subsample of the larger tetrapod mtDNA dataset (Fig. S1) and focuses on relationships among squamate species. This topology for relationships among squamates was used to constrain relationships among the tetrapod mtDNAs (Fig. S3).
