# Supplementary material for: Adaptive Evolution and Functional Redesign of Core Metabolic Proteins in Snakes
Source: PLoS One. 2008 May 21;3(5):e2201. doi: 10.1371/journal.pone.0002201 (PMC2376058; doi:10.1371/journal.pone.0002201)
Supplement: Figure S5 — Results of traditional dN/dS estimates for the CytB gene using the Figure S3 phylogeny with branches colored based on dN/dS ratios. (0.08 MB PDF) [file pone.0002201.s005.pdf]

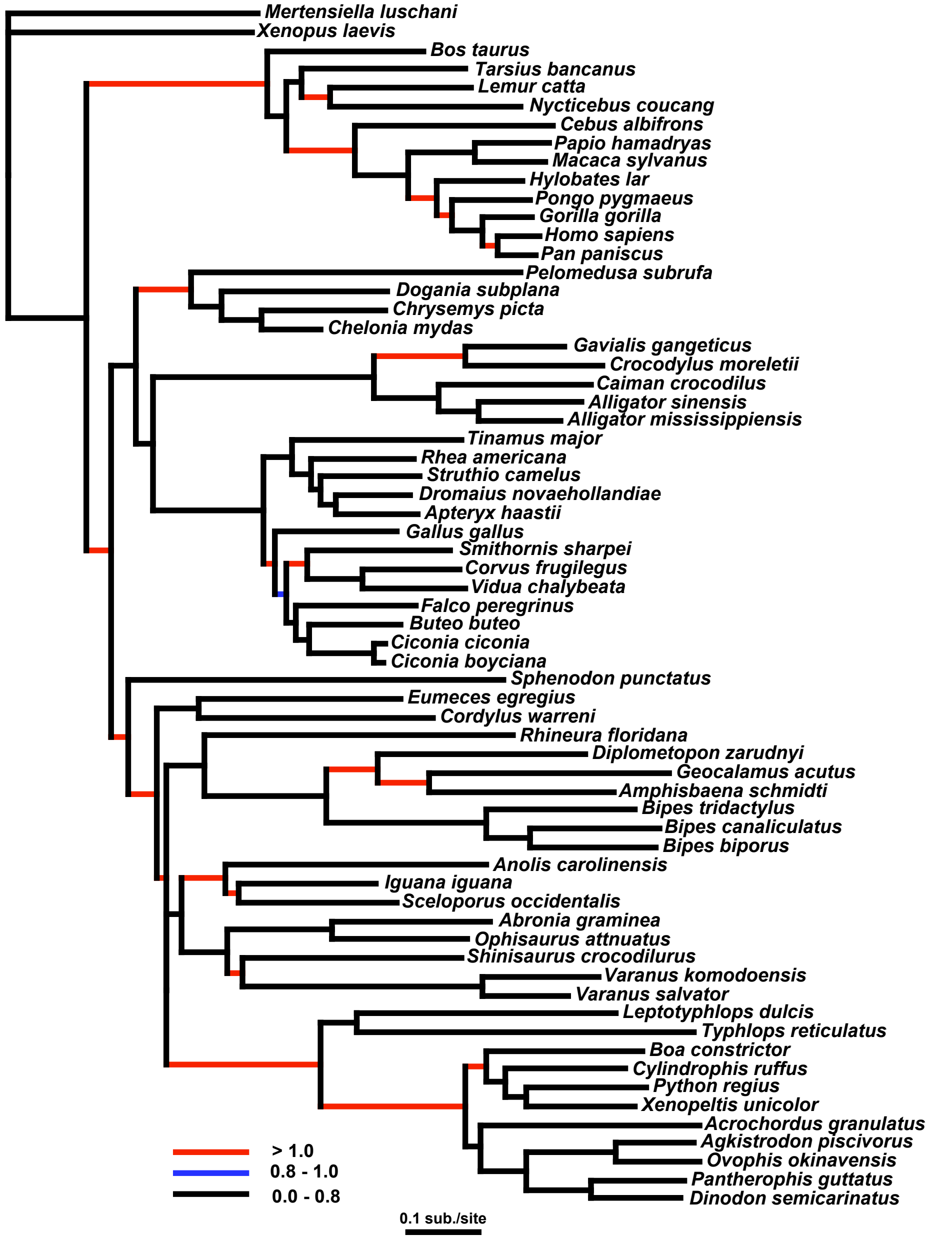

Figure S5. Results of traditional *dN/dS* estimates for the CytB gene using the Fig. S3 phylogeny with branches colored based on *dN/dS* ratios. Estimates of *dN/dS* are from codon model-based analyses conducted HyPhy (See Supplementary Methods).
