# Supplementary material for: Adaptive Evolution and Functional Redesign of Core Metabolic Proteins in Snakes
Source: PLoS One. 2008 May 21;3(5):e2201. doi: 10.1371/journal.pone.0002201 (PMC2376058; doi:10.1371/journal.pone.0002201)
Supplement: Figure S8 — Results of traditional dN/dS estimates for the CytB gene using the alternative (mitochondrial gene-based) topology from Figure S1, with branches colored based on dN/dS ratios. (0.09 MB PDF) [file pone.0002201.s008.pdf]

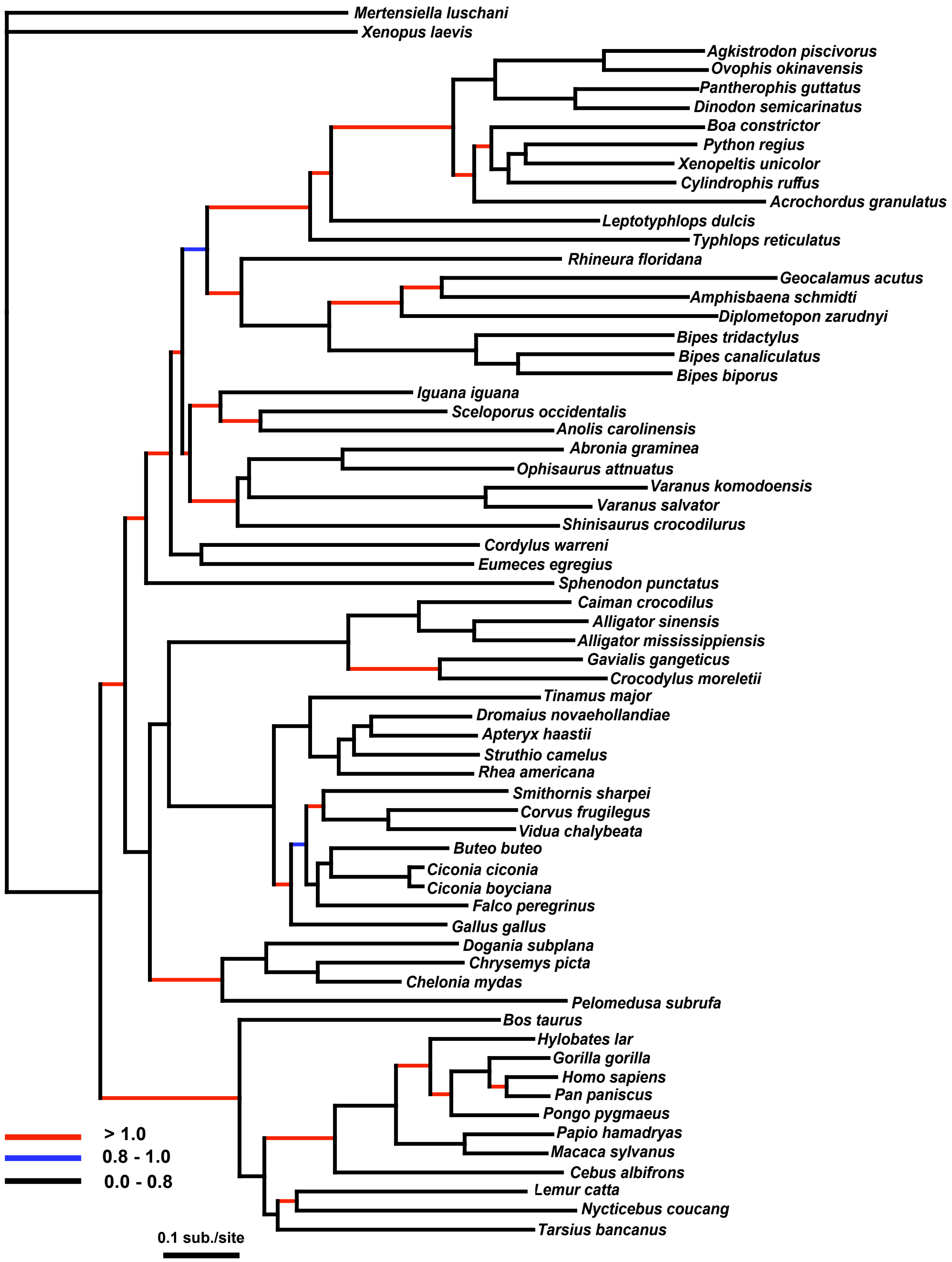

Figure S8. Results of traditional *dN/dS* estimates for the CytB gene using the alternative (mitochondrial gene-based) topology from Fig. S1, with branches colored based on *dN/dS* ratios. Branch lengths are based on all mitochondrial protein-coding genes, optimized on this topology in PAUP* under a GTRΓI model. Estimates of *dN/dS* are from codon model-based analyses conducted HyPhy (See Supplementary Methods).
