# Supplementary material for: Adaptive Evolution and Functional Redesign of Core Metabolic Proteins in Snakes
Source: PLoS One. 2008 May 21;3(5):e2201. doi: 10.1371/journal.pone.0002201 (PMC2376058; doi:10.1371/journal.pone.0002201)
Supplement: Figure S9 — Correlation between the number of transversion substitutions at 4-fold redundant sites (TV4X) in all mitochondrial proteins versus dSTV4X COI. (0.06 MB PDF) [file pone.0002201.s009.pdf]

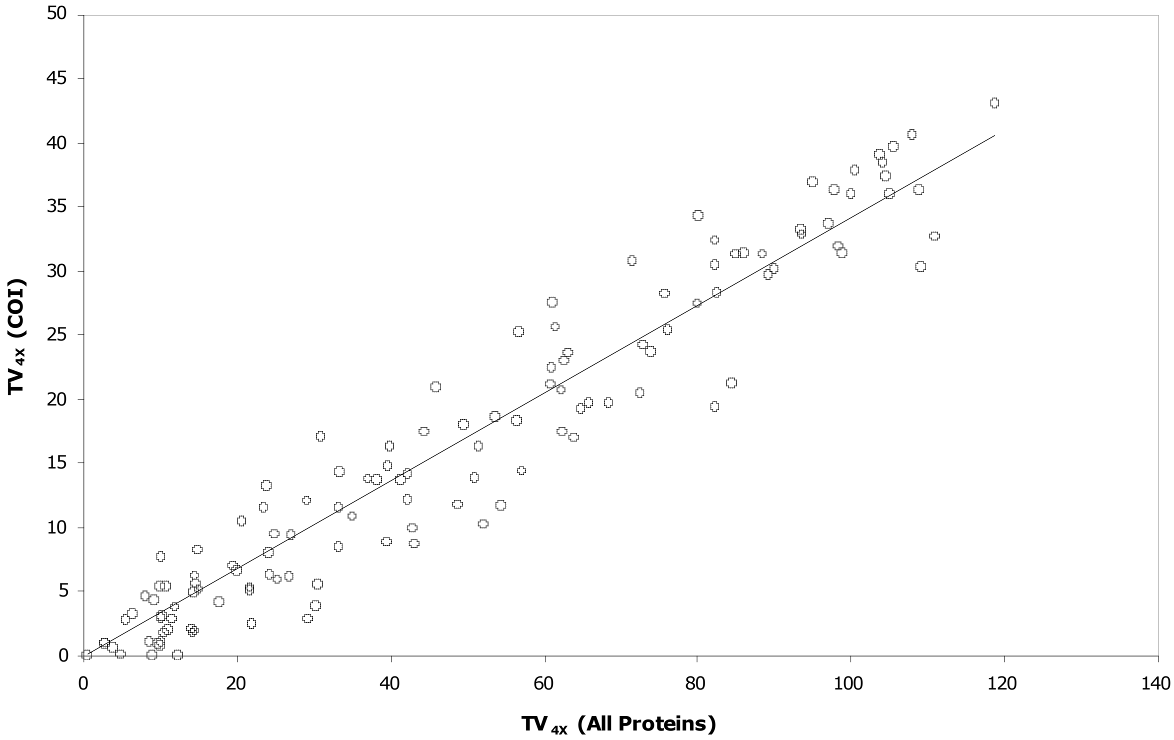

Figure S9. Correlation between the number of transversion substitutions at 4-fold redundant sites (TV_{4X}) in all mitochondrial proteins versus dS_{TV4X} COI. Each point represents a branch of the tree. Best-fit line: $y = 0.3412X$, $r^2 = 0.9288$, $p < 0.001$.
