# Supplementary material for: Adaptive Evolution and Functional Redesign of Core Metabolic Proteins in Snakes
Source: PLoS One. 2008 May 21;3(5):e2201. doi: 10.1371/journal.pone.0002201 (PMC2376058; doi:10.1371/journal.pone.0002201)
Supplement: Figure S10 — Estimated amino acid replacements (proportion of replacements per sequence) versus transversion substitutions at 1st and 2nd codon positions (dNTV12). (0.07 MB PDF) [file pone.0002201.s010.pdf]

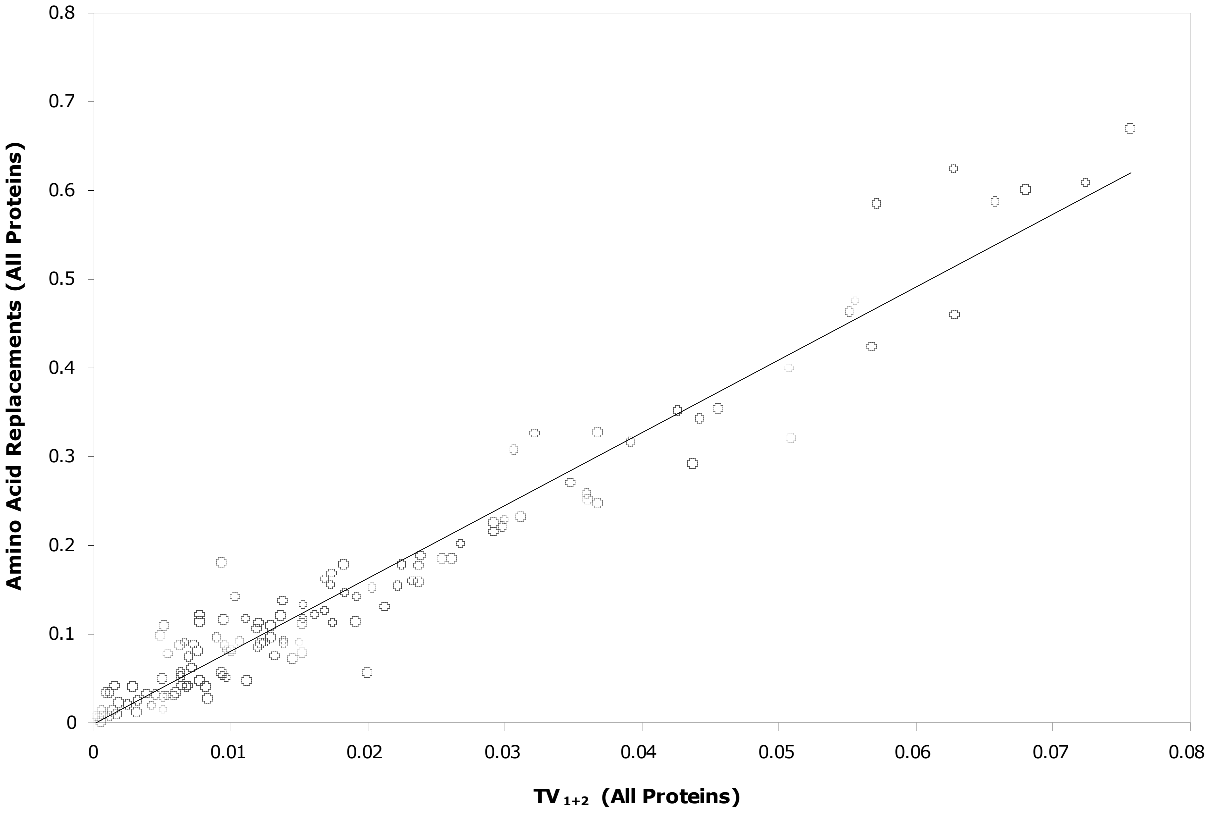

Figure S10. Estimated amino acid replacements (proportion of replacements per sequence) versus transversion substitutions at 1st and 2nd codon positions (dN_{TV12}). Values are given per site for all mitochondrial protein-coding genes. The number of amino acid replacements were estimated using PAML (see Methods). Best-fit line: $y = 8.1932x$, $r^2 = 0.9456$, $p < 0.001$.
