# Supplementary material for: Adaptive Evolution and Functional Redesign of Core Metabolic Proteins in Snakes
Source: PLoS One. 2008 May 21;3(5):e2201. doi: 10.1371/journal.pone.0002201 (PMC2376058; doi:10.1371/journal.pone.0002201)
Supplement: Figure S11 — Conservative transversion-based approximations of the relative rates of non-synonymous to synonymous substitution (dNTV12 / dSTV4x) rates for the mitochondrial Cytochrome B (CytB) gene suggest that rates of amino acid replacement along br (0.12 MB PDF) [file pone.0002201.s011.pdf]

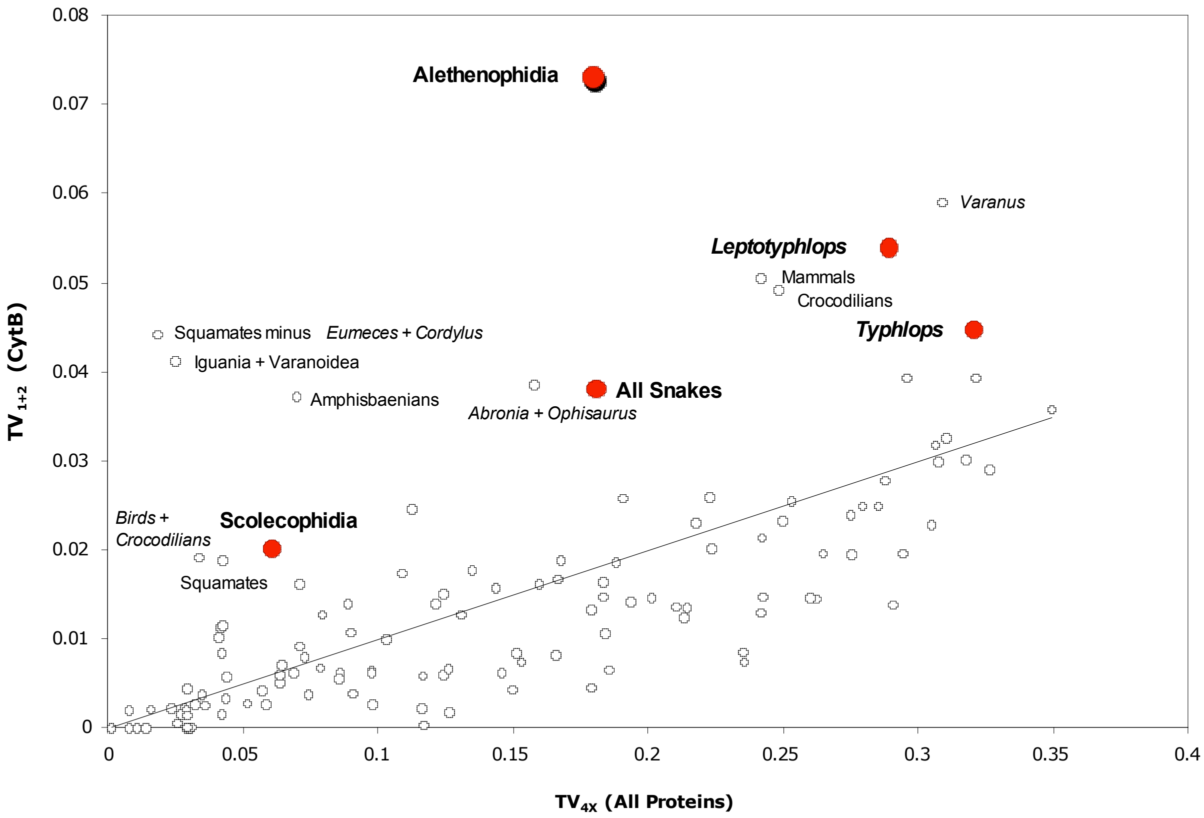

Figure S11. Conservative transversion-based approximations of the relative rates of non-synonymous to synonymous substitution (dN_{TV12} / dS_{TV4x}) rates for the mitochondrial Cytochrome B (CytB) gene suggest that rates of amino acid replacement along branches early in snake evolution (red circles) have been extremely accelerated, especially the branch leading to the Alethinophidia. Several very long branches that occur at the extreme right (e.g., *Varanus*) have high rates of dN_{TV12} / dS_{TV4x} possibly indicating accelerated amino acid change or erroneous underestimation of synonymous transversions due to saturation; estimates at this extreme end of the graph should be interpreted with caution.
