# Supplementary material for: Adaptive Evolution and Functional Redesign of Core Metabolic Proteins in Snakes
Source: PLoS One. 2008 May 21;3(5):e2201. doi: 10.1371/journal.pone.0002201 (PMC2376058; doi:10.1371/journal.pone.0002201)
Supplement: Figure S12 — Three dimensional view of the ribbon structure of the 13 subunits of the monomer of CO. (0.39 MB PDF) [file pone.0002201.s012.pdf]

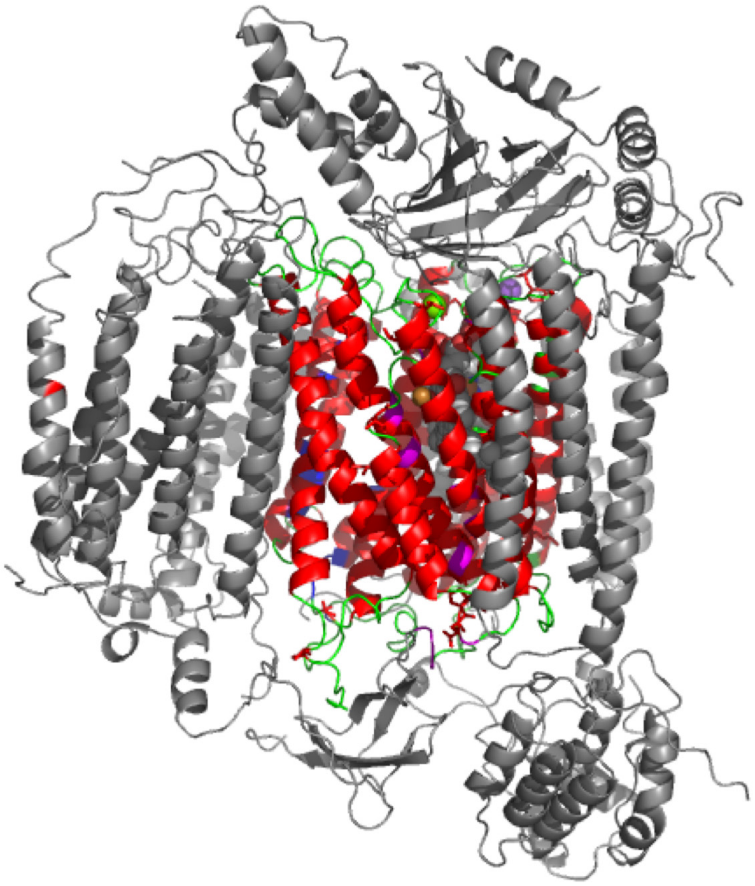

Figure S12. Three dimensional view of the ribbon structure of the 13 subunits of the monomer of CO. The COI subunit (in red) resides at the core of the CO polypeptide complex and is surrounded by the other 12 subunits (in grey).
