# Supplementary material for: Adaptive Evolution and Functional Redesign of Core Metabolic Proteins in Snakes
Source: PLoS One. 2008 May 21;3(5):e2201. doi: 10.1371/journal.pone.0002201 (PMC2376058; doi:10.1371/journal.pone.0002201)
Supplement: Figure S13 — Two different three dimensional views of the ribbon structure of cytochrome C oxidase subunit I (COI) with major functional regions and features illustrated. (1.13 MB PDF) [file pone.0002201.s013.pdf]

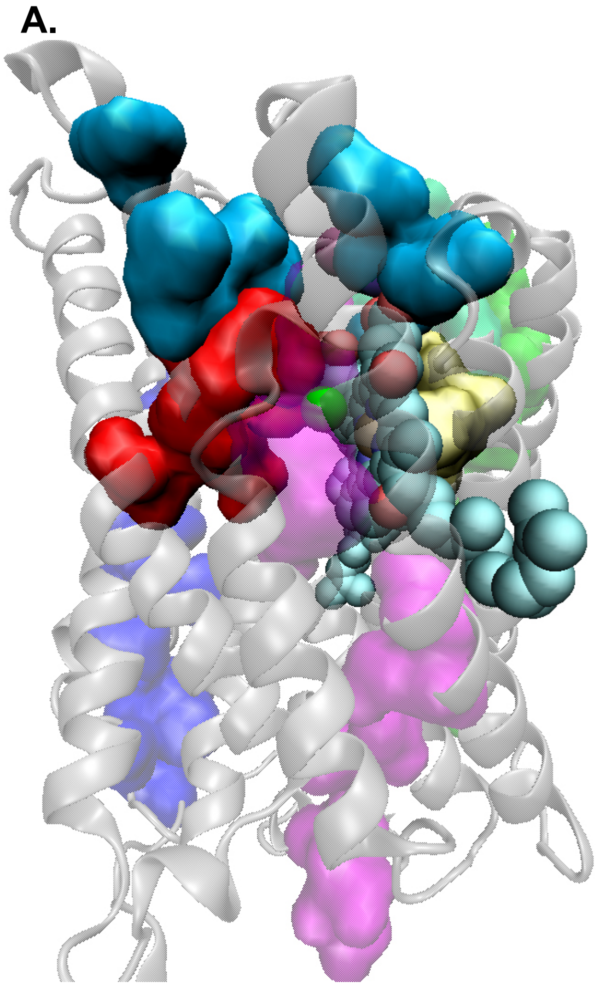

Figure S13. Two different three dimensional views of the ribbon structure of cytochrome C oxidase subunit I (COI) with major functional regions and features illustrated. Figure A is the approximately the front of the molecule and Figure B is approximately the back of the molecule. The two heme groups are indicated with molecular spacefill shading (predominantly light blue and red) at the center of the protein. Spacefill representations of the residues that form the three proton channels are indicated in transparent blue (channel D), transparent majenta (channel K) and transparent green (channel H; note that the main conduit for channel H is shown in light green, the alternative route in darker green). Other channels are shown in solid spacefill, including the oxygen channel (red), the electron channel (yellow) and water channels (light blue).
