# Supplementary material for: Adaptive Evolution and Functional Redesign of Core Metabolic Proteins in Snakes
Source: PLoS One. 2008 May 21;3(5):e2201. doi: 10.1371/journal.pone.0002201 (PMC2376058; doi:10.1371/journal.pone.0002201)
Supplement: Figure S14 — The three proposed proton transfer channels in COI. (0.50 MB PDF) [file pone.0002201.s014.pdf]

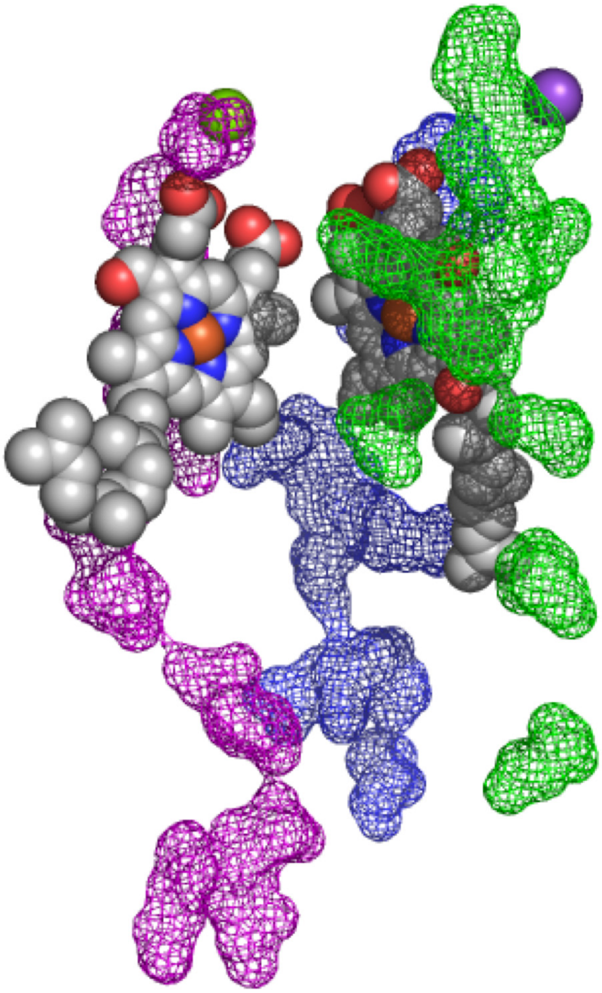

Figure S14. The three proposed proton transfer channels in COI. Channels are expressed by the electron density of amino acids assembling each channel. Channel identification is as follows: channel D (blue), channel H (green), and channel K (magenta).
