# Supplementary material for: Adaptive Evolution and Functional Redesign of Core Metabolic Proteins in Snakes
Source: PLoS One. 2008 May 21;3(5):e2201. doi: 10.1371/journal.pone.0002201 (PMC2376058; doi:10.1371/journal.pone.0002201)
Supplement: Figure S15 — Three-dimensional views of the three proton channels within the ribbon structure of cytochrome C oxidase subunit 1 (COI; based on the cow COI structure). (0.96 MB PDF) [file pone.0002201.s015.pdf]

A.

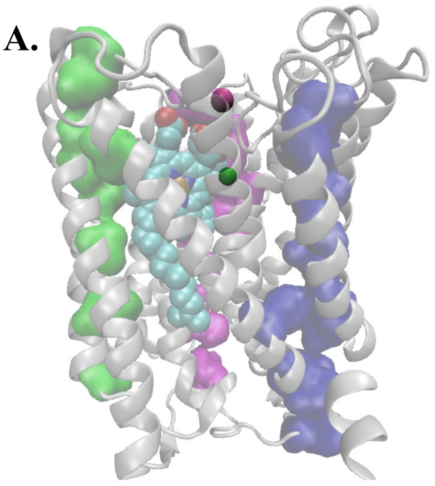

D.

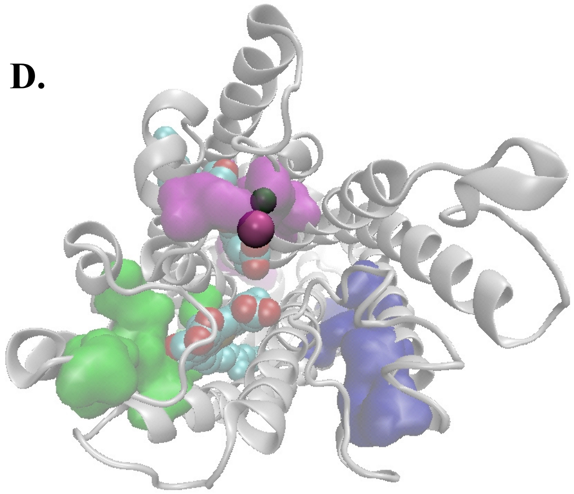

B.

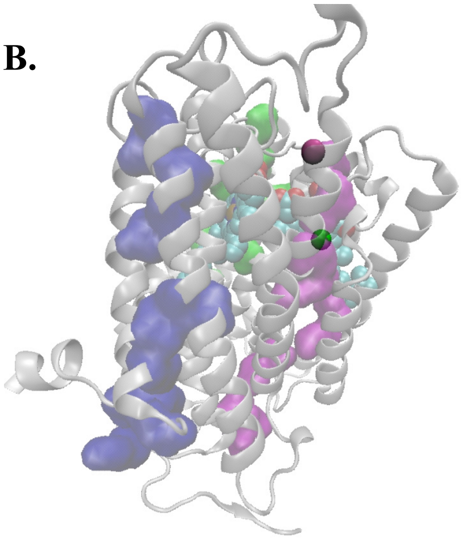

E.

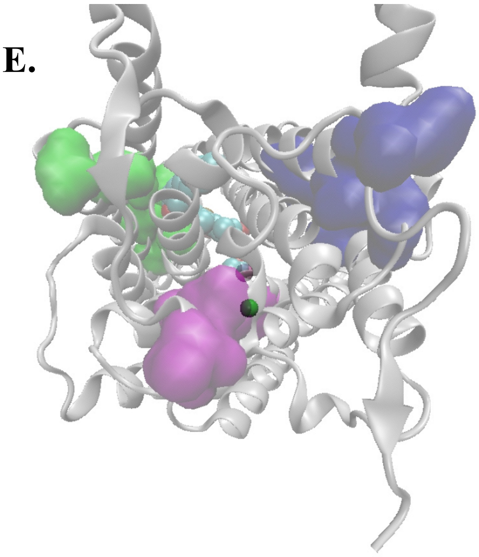

C.

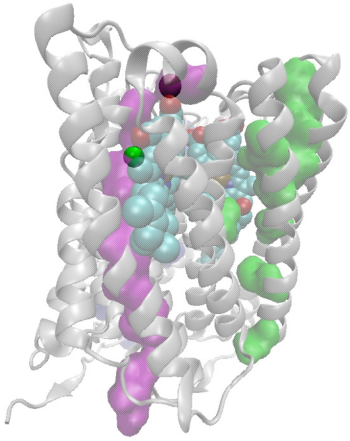

**Figure S15.** Three-dimensional views of the three proton channels within the ribbon structure of cytochrome C oxidase subunit 1 (COI; based on the cow COI structure). The proton channels are shown in blue (channel D), green (channel H), and magenta (channel K) space-fill; heme groups are shown in light blue and red, the sodium and magnesium ions are shown with pink and green balls, respectively. (A) channels H and D, (B) channels D and K, (C) channels K and H, (D) top view, (E) bottom view.
