# Supplementary material for: Adaptive Evolution and Functional Redesign of Core Metabolic Proteins in Snakes
Source: PLoS One. 2008 May 21;3(5):e2201. doi: 10.1371/journal.pone.0002201 (PMC2376058; doi:10.1371/journal.pone.0002201)
Supplement: Figure S16 — Locations of unique substitutions projected over the location of the reaction center and proton transfer channels. (0.67 MB PDF) [file pone.0002201.s016.pdf]

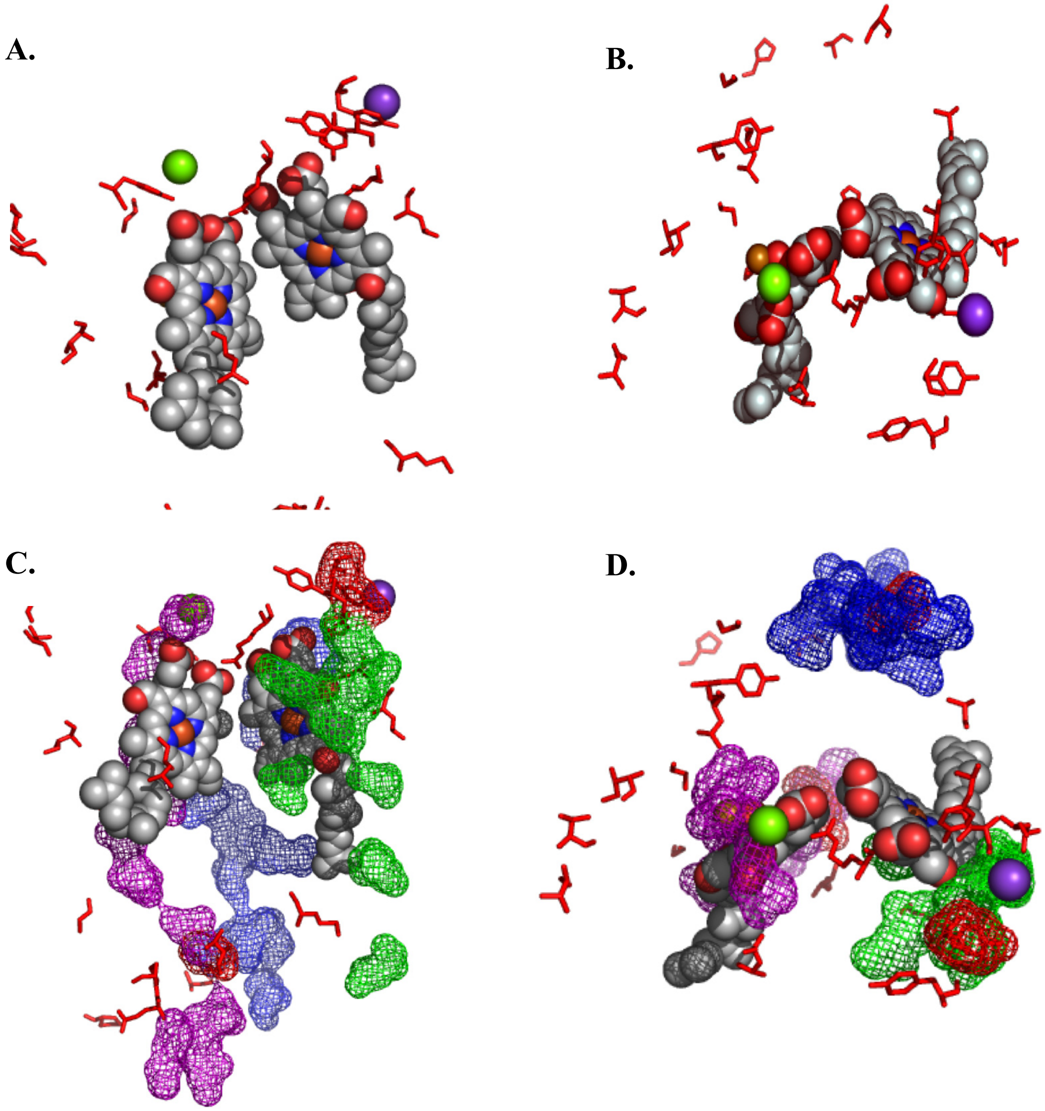

Figure S16. Locations of unique substitutions projected over the location of the reaction center and proton transfer channels. The bi-heme reaction center of COI is shown from the side (A) and top (B), and is also shown with the proposed proton transfer channels from the side-view (C) and top-view (D). Red colored amino acid side chain structure (sticks) are where unique substitutions occurred. Proton transfer channels (C, D) are expressed by electron density of the amino acids assembling the channels; Feature colors are as follows: channel D (blue), channel H (green), channel K (magenta), magnesium ion (green ball), and sodium ion (magenta ball).
