# Supplementary material for: Adaptive Evolution and Functional Redesign of Core Metabolic Proteins in Snakes
Source: PLoS One. 2008 May 21;3(5):e2201. doi: 10.1371/journal.pone.0002201 (PMC2376058; doi:10.1371/journal.pone.0002201)
Supplement: Figure S17 — Three-dimensional views of the alternative pathways of proton channel H within the ribbon structure of cytochrome C oxidase subunit 1 (COI; based on the cow COI structure). (1.24 MB PDF) [file pone.0002201.s017.pdf]

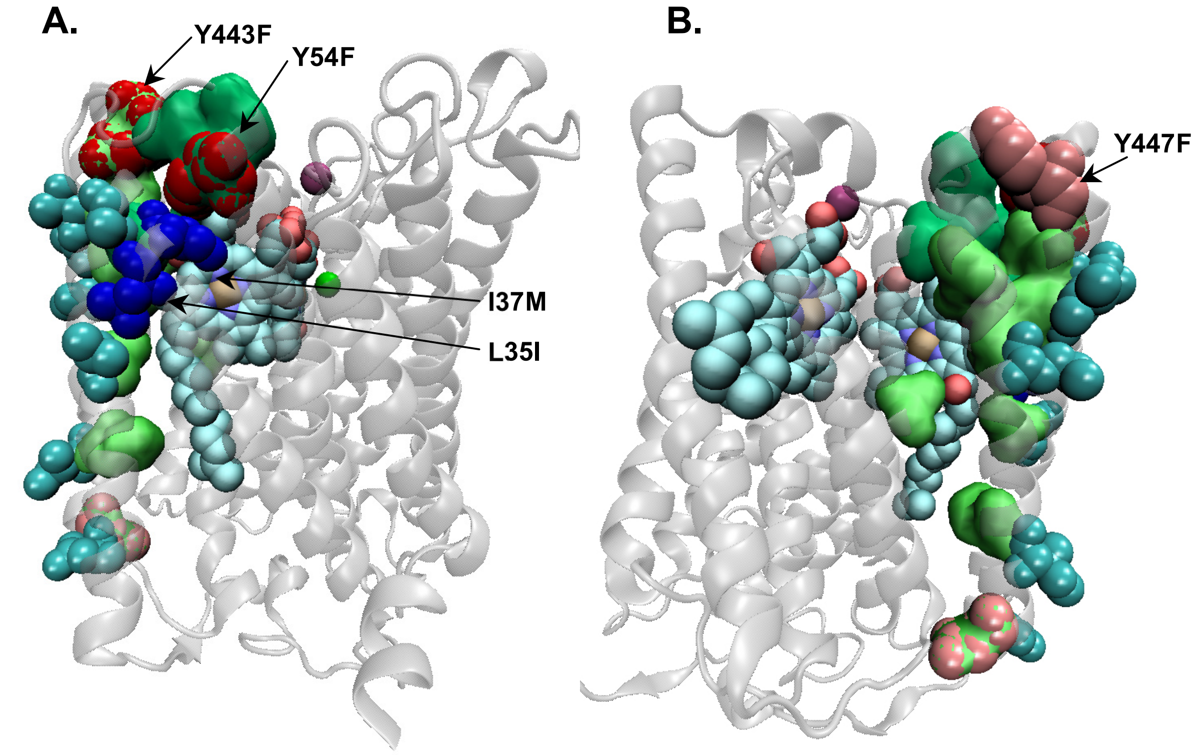

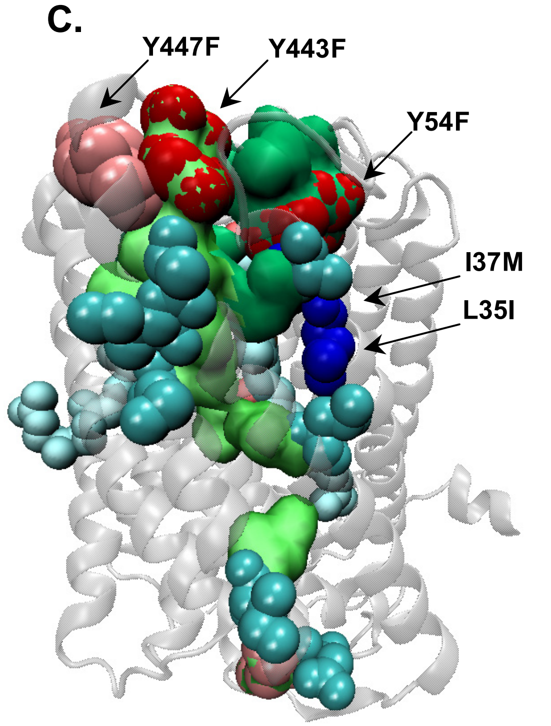

Figure S17. Three-dimensional views of the alternative pathways of proton channel H within the ribbon structure of cytochrome C oxidase subunit 1 (COI; based on the cow COI structure). The traditional channel H pathway is light green (channel H), and alternative channel H pathway is shown in dark green space-fill; heme groups are shown in light blue and red, the sodium and magnesium ions are shown with magenta and green balls, respectively. Three front (A), back (B) and side (C) perspective views are shown. Important amino acid replacements that are discussed in the text are labeled. Residues that have been replaced in snakes are shown in spacefill representations following the color scheme in Fig. 3 (red = unique in channel, pink = non unique within channel, dark blue = unique adjacent to channel, light blue = non unique adjacent to channel). See table S6 for details of the amino acid replacements at these unique sites.
