# Supplementary material for: Adaptive Evolution and Functional Redesign of Core Metabolic Proteins in Snakes
Source: PLoS One. 2008 May 21;3(5):e2201. doi: 10.1371/journal.pone.0002201 (PMC2376058; doi:10.1371/journal.pone.0002201)
Supplement: Figure S18 — Nucleotide Alignment (7.64 MB PDF) [file pone.0002201.s018.pdf]

Online Alignment Supplement – Nucleic acid alignment of 13 mitochondrial protein-coding genes used for all analyses.

Coordinates for genes are as follows:

Gene	Start Position	End Position
ATP6	1	729
ATP8	730	999
COX1	1000	2613
COX2	2614	3321
COX3	3322	4164
CYTB	4165	5403
ND1	5404	6402
ND2	6403	7497
ND3	7498	7890
ND4	7891	9339
ND4L	9340	9642
ND5	9643	11676
ND6	11677	12270

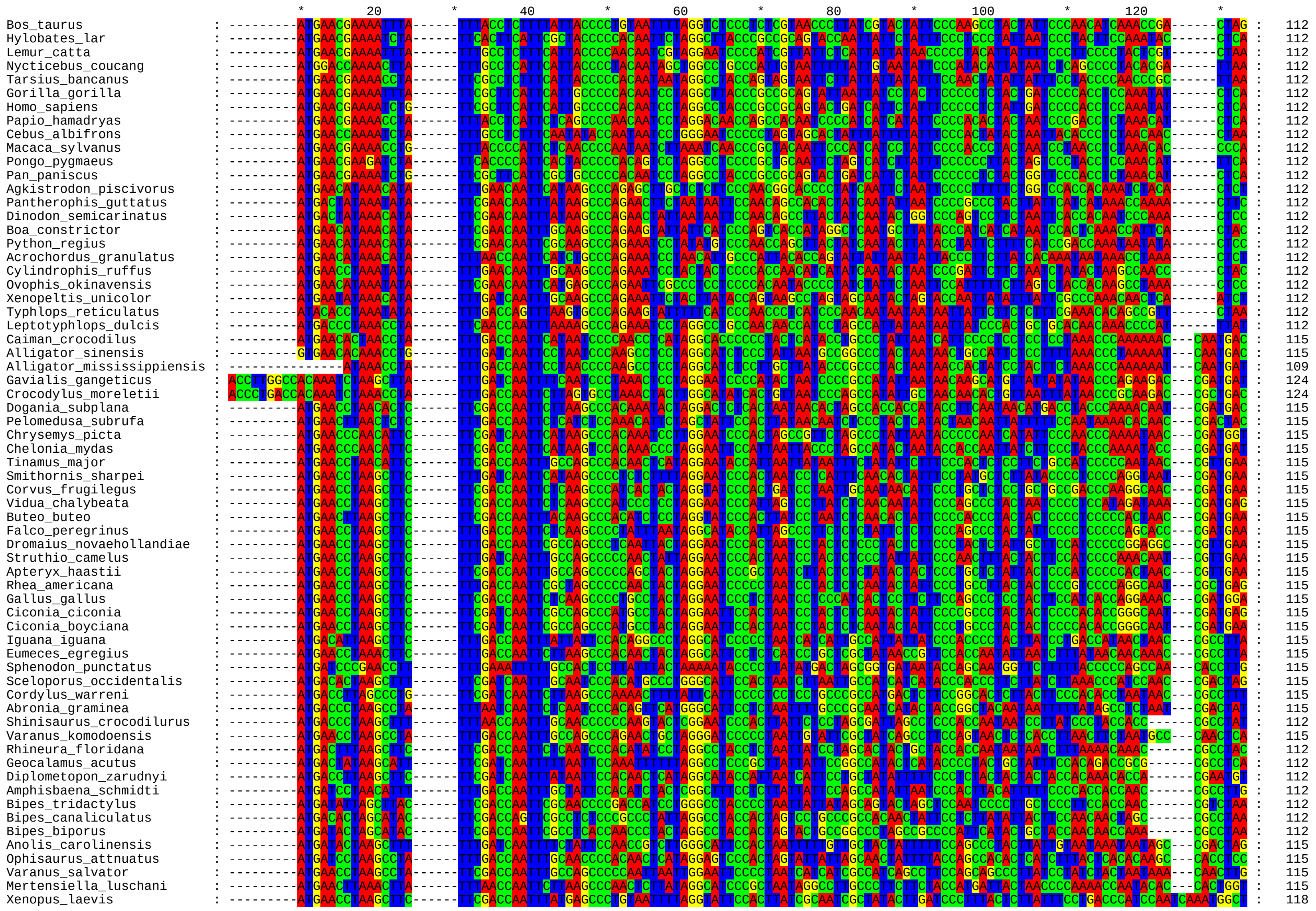
Bos_taurus 112
Hylobates_lar 112
Lemur_catta 112
Nycticebus_coucang 112
Tarsius_bancanus 112
Gorilla_gorilla 112
Homo_sapiens 112
Papio_hamadryas 112
Cebus_albifrons 112
Macaca_sylvanus 112
Pongo_pygmaeus 112
Pan_paniscus 112
Agkistrodon_piscivorus 112
Pantherophis_guttatus 112
Dinodon_semicarinatus 112
Boa_constrictor 112
Python_regius 112
Acrochordus_granulatus 112
Cylindrophis_ruffus 112
Ovophis_okinavensis 112
Xenopeltis_unicolor 112
Typhlops_reticulatus 112
Leptotyphlops_dulcis 112
Caiman_crocodilus 115
Alligator_sinensis 115
Alligator_mississippiensis 109
Gavialis_gangeticus 124
Crocodylus_moreletii 124
Dogania_subplana 115
Pelomedusa_subrufa 115
Chrysemys_picta 115
Chelonia_mydas 115
Tinamus_major 115
Smithornis_sharpei 115
Corvus_frugilegus 115
Vidua_chalybeata 115
Buteo_buteo 115
Falco_peregrinus 115
Dromaius_novaehollandiae 115
Struthio_camelus 115
Apteryx_haastii 115
Rhea_americana 115
Gallus_gallus 115
Ciconia_ciconia 115
Ciconia_boyciana 115
Iguana_iguana 115
Eumeces_egregius 115
Sphenodon_punctatus 115
Sceloporus_occidentalis 115
Cordylus_warreni 115
Abronia_graminea 115
Shinisaurus_crocodilurus 112
Varanus_komodoensis 115
Rhineura_floridana 112
Geocalamus_acutus 112
Diplometopon_zarudnyi 112
Amphisbaena_schmidti 112
Bipes_tridactylus 112
Bipes_canaliculatus 112
Bipes_biporus 112
Anolis_carolinensis 115
Ophisaurus_attnuatus 115
Varanus_salvator 115
Mertensiella_luschani 115
Xenopus_laevis 118

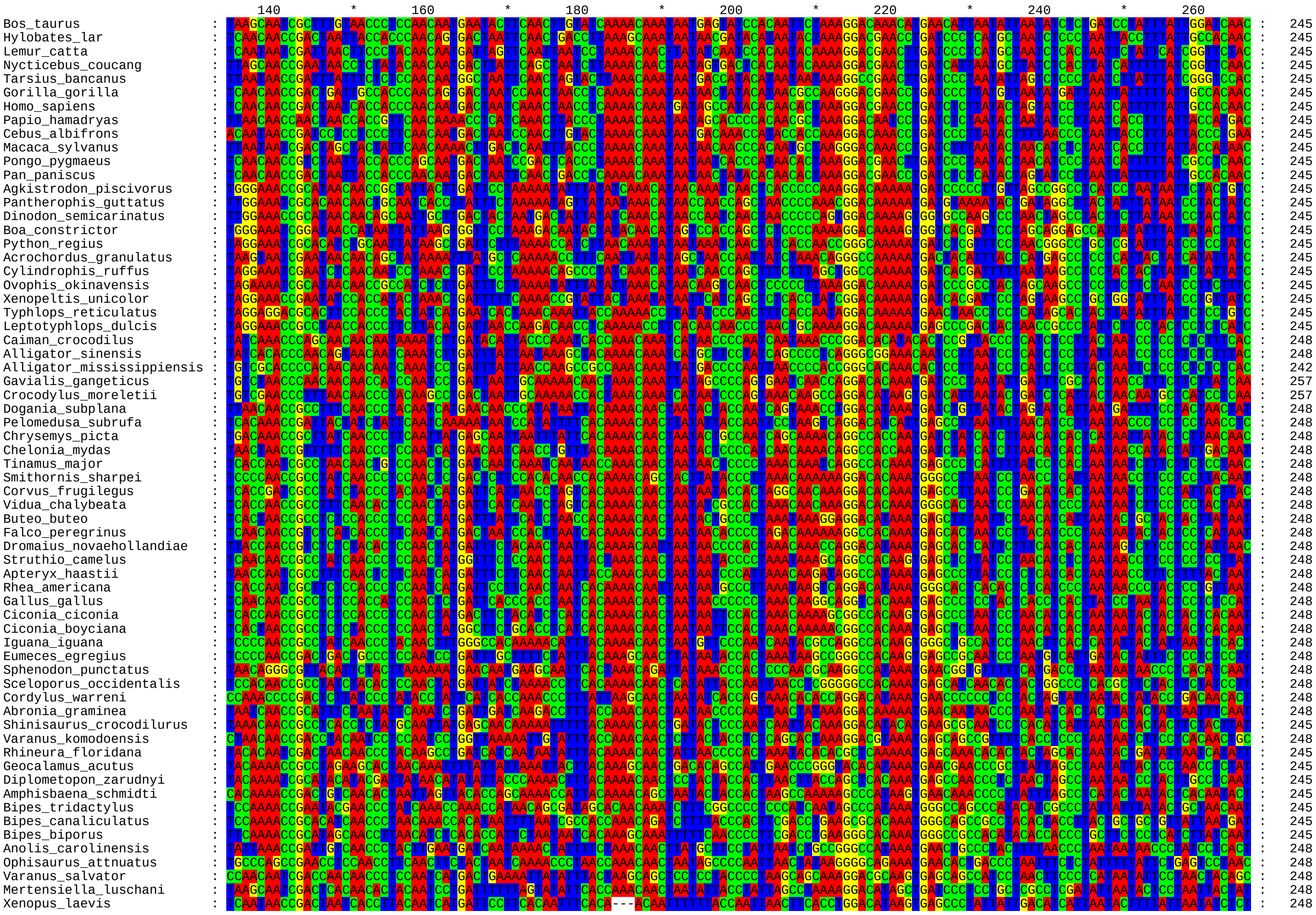
140 * 160 * 180 * 200 * 220 * 240 * 260
Bos_taurus : 245
Hylobates_lar : 245
Lemur_catta : 245
Nycticebus_coucang : 245
Tarsius_bancanus : 245
Gorilla_gorilla : 245
Homo_sapiens : 245
Papio_hamadryas : 245
Cebus_albifrons : 245
Macaca_sylvanus : 245
Pongo_pygmaeus : 245
Pan_paniscus : 245
Agkistrodon_piscivorus : 245
Pantherophis_guttatus : 245
Dinodon_semicarinatus : 245
Boa_constrictor : 245
Python_regius : 245
Acrochordus_granulatus : 245
Cylindrophis_ruffus : 245
Ovophis_okinavensis : 245
Xenopeltis_unicolor : 245
Typhlops_reticulatus : 245
Leptotyphlops_dulcis : 245
Caiman_crocodilus : 248
Alligator_sinensis : 248
Alligator_mississippiensis : 242
Gavialis_gangeticus : 257
Crocodylus_moreletii : 257
Dogania_subplana : 248
Pelomedusa_subrufa : 248
Chrysemys_picta : 248
Chelonia_mydas : 248
Tinamus_major : 248
Smithornis_sharpei : 248
Corvus_frugilegus : 248
Vidua_chalybeata : 248
Buteo_buteo : 248
Falco_peregrinus : 248
Dromaius_novaehollandiae : 248
Struthio_camelus : 248
Apteryx_haastii : 248
Rhea_americana : 248
Gallus_gallus : 248
Ciconia_ciconia : 248
Ciconia_boyciana : 248
Iguana_iguana : 248
Eumeces_egregius : 248
Sphenodon_punctatus : 248
Sceloporus_occidentalis : 248
Cordylus_warreni : 248
Abronia_graminea : 248
Shinisaurus_crocodilurus : 245
Varanus_komodoensis : 248
Rhineura_floridana : 245
Geocalamus_acutus : 245
Diplometopon_zarudnyi : 245
Amphisbaena_schmidti : 245
Bipes_tridactylus : 245
Bipes_canaliculatus : 245
Bipes_biporus : 245
Anolis_carolinensis : 248
Ophisaurus_attnuatus : 248
Varanus_salvator : 248
Mertensiella_luschani : 248
Xenopus_laevis : 248

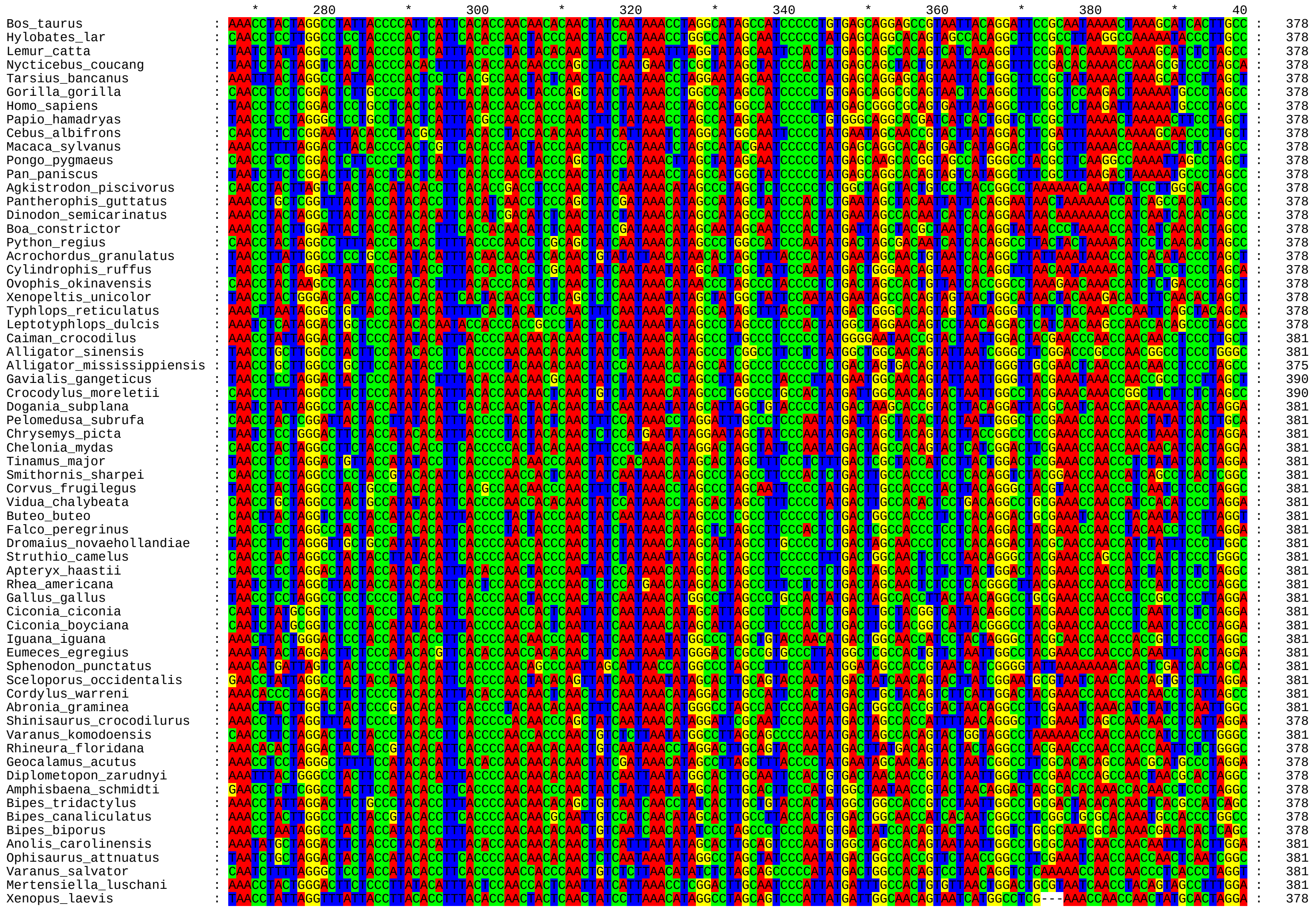
Bos_taurus
Hylobates_lar
Lemur_catta
Nycticebus_coucang
Tarsius_bancanus
Gorilla_gorilla
Homo_sapiens
Papio_hamadryas
Cebus_albifrons
Macaca_sylvanus
Pongo_pygmaeus
Pan_paniscus
Agkistrodon_piscivorus
Pantherophis_guttatus
Dinodon_semicarinatus
Boa_constrictor
Python_regius
Acrochordus_granulatus
Cylindrophis_ruffus
Ovophis_okinavensis
Xenopeltis_unicolor
Typhlops_reticulatus
Leptotyphlops_dulcis
Caiman_crocodilus
Alligator_sinensis
Alligator_mississippiensis
Gavialis_gangeticus
Crocodylus_moreletii
Dogania_subplana
Pelomedusa_subrufa
Chrysemys_picta
Chelonia_mydas
Tinamus_major
Smithornis_sharpei
Corvus_frugilegus
Vidua_chalybeata
Buteo_buteo
Falco_peregrinus
Dromaius_novaehollandiae
Struthio_camelus
Apteryx_haastii
Rhea_americana
Gallus_gallus
Ciconia_ciconia
Ciconia_boyciana
Iguana_iguana
Eumeces_egregius
Sphenodon_punctatus
Sceloporus_occidentalis
Cordylus_warreni
Abronia_graminea
Shinisaurus_crocodilurus
Varanus_komodoensis
Rhineura_floridana
Geocalamus_acutus
Diplometopon_zarudnyi
Amphisbaena_schmidti
Bipes_tridactylus
Bipes_canaliculatus
Bipes_biporus
Anolis_carolinensis
Ophisaurus_attnuatus
Varanus_salvator
Mertensiella_luschani
Xenopus_laevis

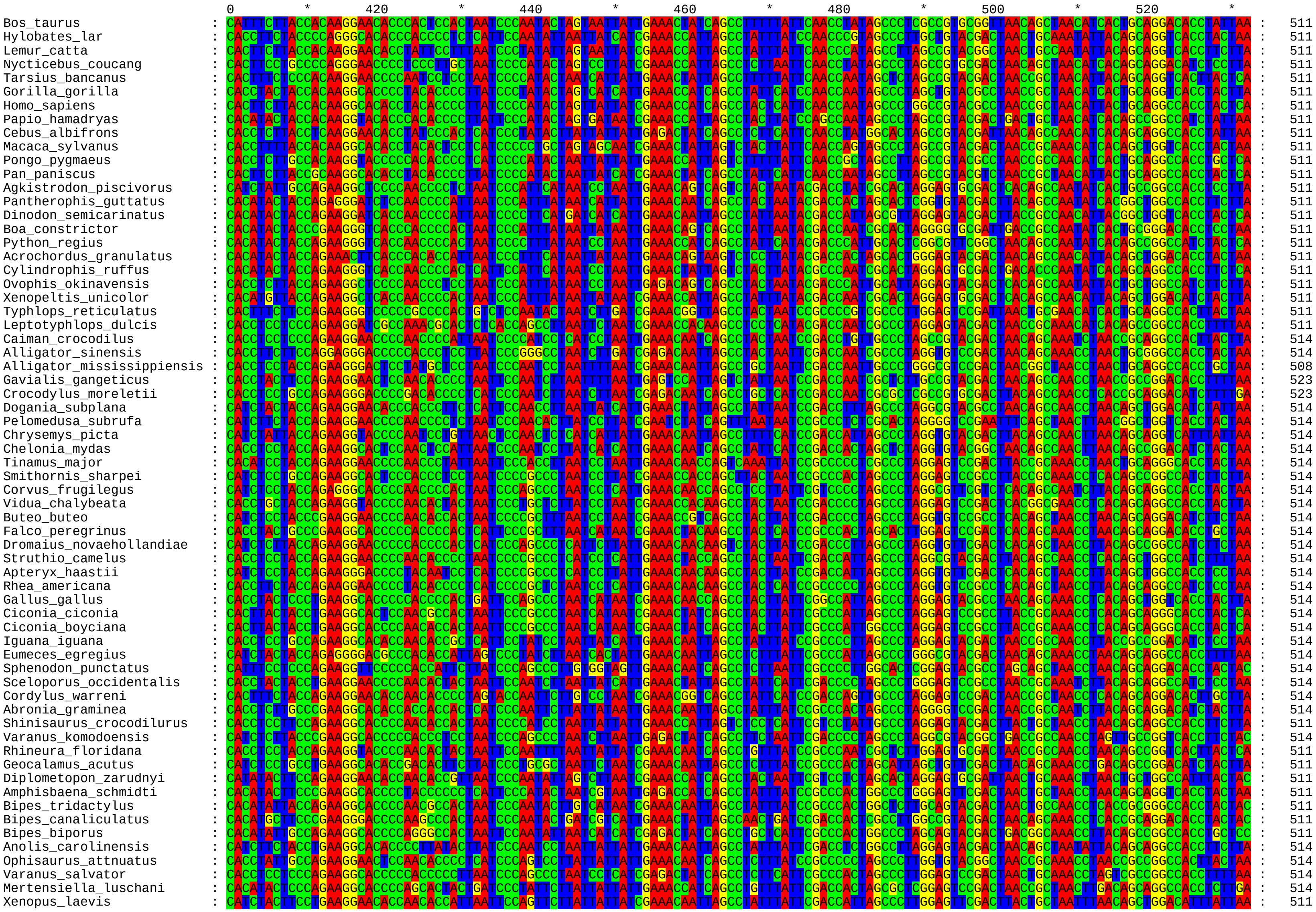

0 * 420 * 440 * 460 * 480 * 500 * 520 *
Bos_taurus : 511
Hylobates_lar : 511
Lemur_catta : 511
Nycticebus_coucang : 511
Tarsius_bancanus : 511
Gorilla_gorilla : 511
Homo_sapiens : 511
Papio_hamadryas : 511
Cebus_albifrons : 511
Macaca_sylvanus : 511
Pongo_pygmaeus : 511
Pan_paniscus : 511
Agkistrodon_piscivorus : 511
Pantherophis_guttatus : 511
Dinodon_semicarinatus : 511
Boa_constrictor : 511
Python_regius : 511
Acrochordus_granulatus : 511
Cylindrophis_ruffus : 511
Ovophis_okinavensis : 511
Xenopeltis_unicolor : 511
Typhlops_reticulatus : 511
Leptotyphlops_dulcis : 511
Caiman_crocodilus : 514
Alligator_sinensis : 514
Alligator_mississippiensis : 508
Gavialis_gangeticus : 523
Crocodylus_moreletii : 523
Dogania_subplana : 514
Pelomedusa_subrufa : 514
Chrysemys_picta : 514
Chelonia_mydas : 514
Tinamus_major : 514
Smithornis_sharpei : 514
Corvus_frugilegus : 514
Vidua_chalybeata : 514
Buteo_buteo : 514
Falco_peregrinus : 514
Dromaius_novaehollandiae : 514
Struthio_camelus : 514
Apteryx_haastii : 514
Rhea_americana : 514
Gallus_gallus : 514
Ciconia_ciconia : 514
Ciconia_boyciana : 514
Iguana_iguana : 514
Eumeces_egregius : 514
Sphenodon_punctatus : 514
Sceloporus_occidentalis : 514
Cordylus_warreni : 514
Abronia_graminea : 514
Shinisaurus_crocodilurus : 511
Varanus_komodoensis : 514
Rhineura_floridana : 511
Geocalamus_acutus : 511
Diplometopon_zarudnyi : 511
Amphisbaena_schmidti : 511
Bipes_tridactylus : 511
Bipes_canaliculatus : 511
Bipes_biporus : 511
Anolis_carolinensis : 514
Ophisaurus_attnuatus : 514
Varanus_salvator : 514
Mertensiella_luschani : 514
Xenopus_laevis : 511

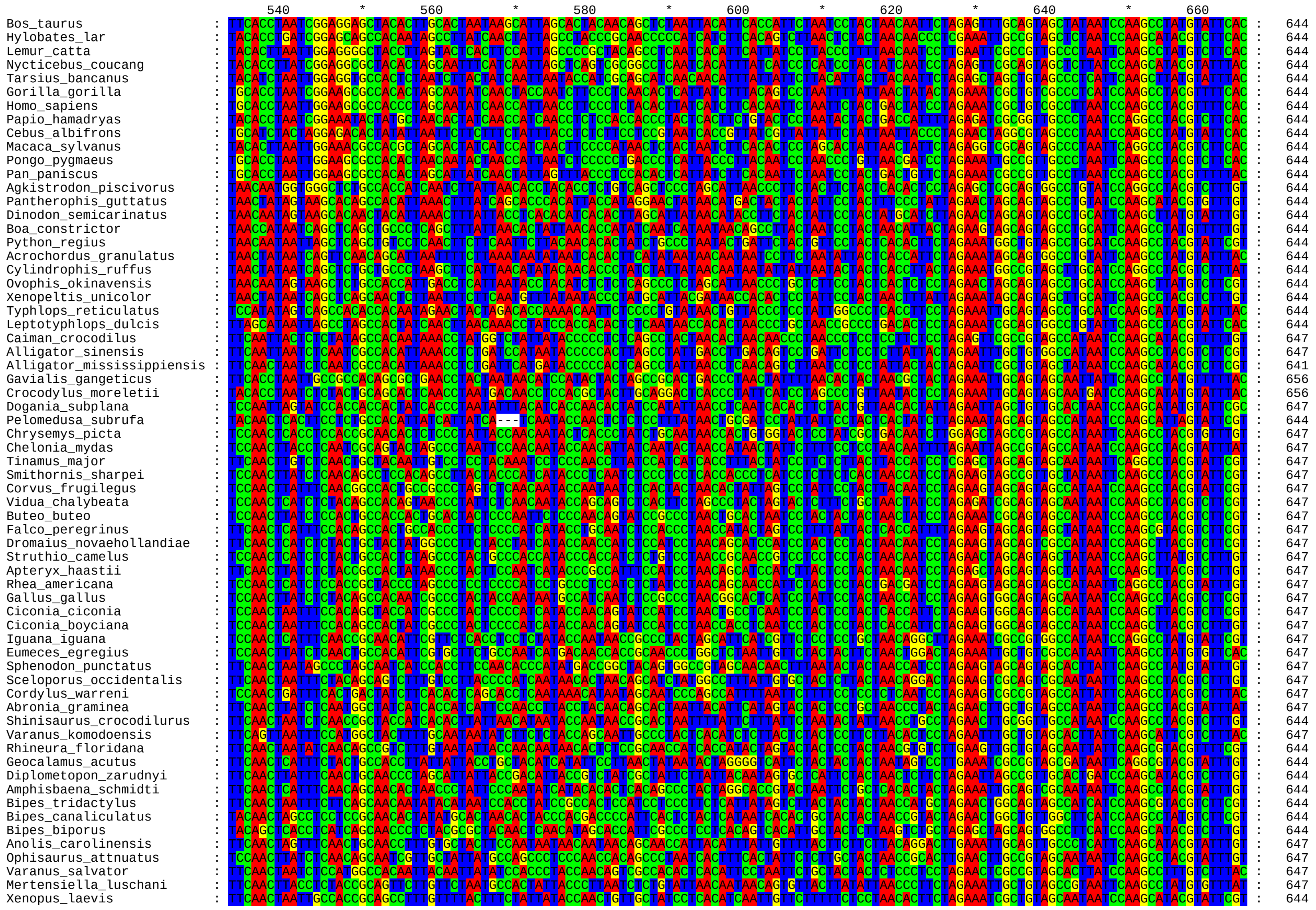
540 * 560 * 580 * 600 * 620 * 640 * 660
Bos_taurus : 644
Hylobates_lar : 644
Lemur_catta : 644
Nycticebus_coucang : 644
Tarsius_bancanus : 644
Gorilla_gorilla : 644
Homo_sapiens : 644
Papio_hamadryas : 644
Cebus_albifrons : 644
Macaca_sylvanus : 644
Pongo_pygmaeus : 644
Pan_paniscus : 644
Agkistrodon_piscivorus : 644
Pantherophis_guttatus : 644
Dinodon_semicarinatus : 644
Boa_constrictor : 644
Python_regius : 644
Acrochordus_granulatus : 644
Cylindrophis_ruffus : 644
Ovophis_okinavensis : 644
Xenopeltis_unicolor : 644
Typhlops_reticulatus : 644
Leptotyphlops_dulcis : 644
Caiman_crocodilus : 647
Alligator_sinensis : 647
Alligator_mississippiensis : 641
Gavialis_gangeticus : 656
Crocodylus_moreletii : 656
Dogania_subplana : 647
Pelomedusa_subrufa : 644
Chrysemys_picta : 647
Chelonia_mydas : 647
Tinamus_major : 647
Smithornis_sharpei : 647
Corvus_frugilegus : 647
Vidua_chalybeata : 647
Buteo_buteo : 647
Falco_peregrinus : 647
Dromaius_novaehollandiae : 647
Struthio_camelus : 647
Apteryx_haastii : 647
Rhea_americana : 647
Gallus_gallus : 647
Ciconia_ciconia : 647
Ciconia_boyciana : 647
Iguana_iguana : 647
Eumeces_egregius : 647
Sphenodon_punctatus : 647
Sceloporus_occidentalis : 647
Cordylus_warreni : 647
Abronia_graminea : 647
Shinisaurus_crocodilurus : 644
Varanus_komodoensis : 647
Rhineura_floridana : 644
Geocalamus_acutus : 644
Diplometopon_zarudnyi : 644
Amphisbaena_schmidti : 644
Bipes_tridactylus : 644
Bipes_canaliculatus : 644
Bipes_biporus : 644
Anolis_carolinensis : 647
Ophisaurus_attnuatus : 647
Varanus_salvator : 647
Mertensiella_luschani : 647
Xenopus_laevis : 644

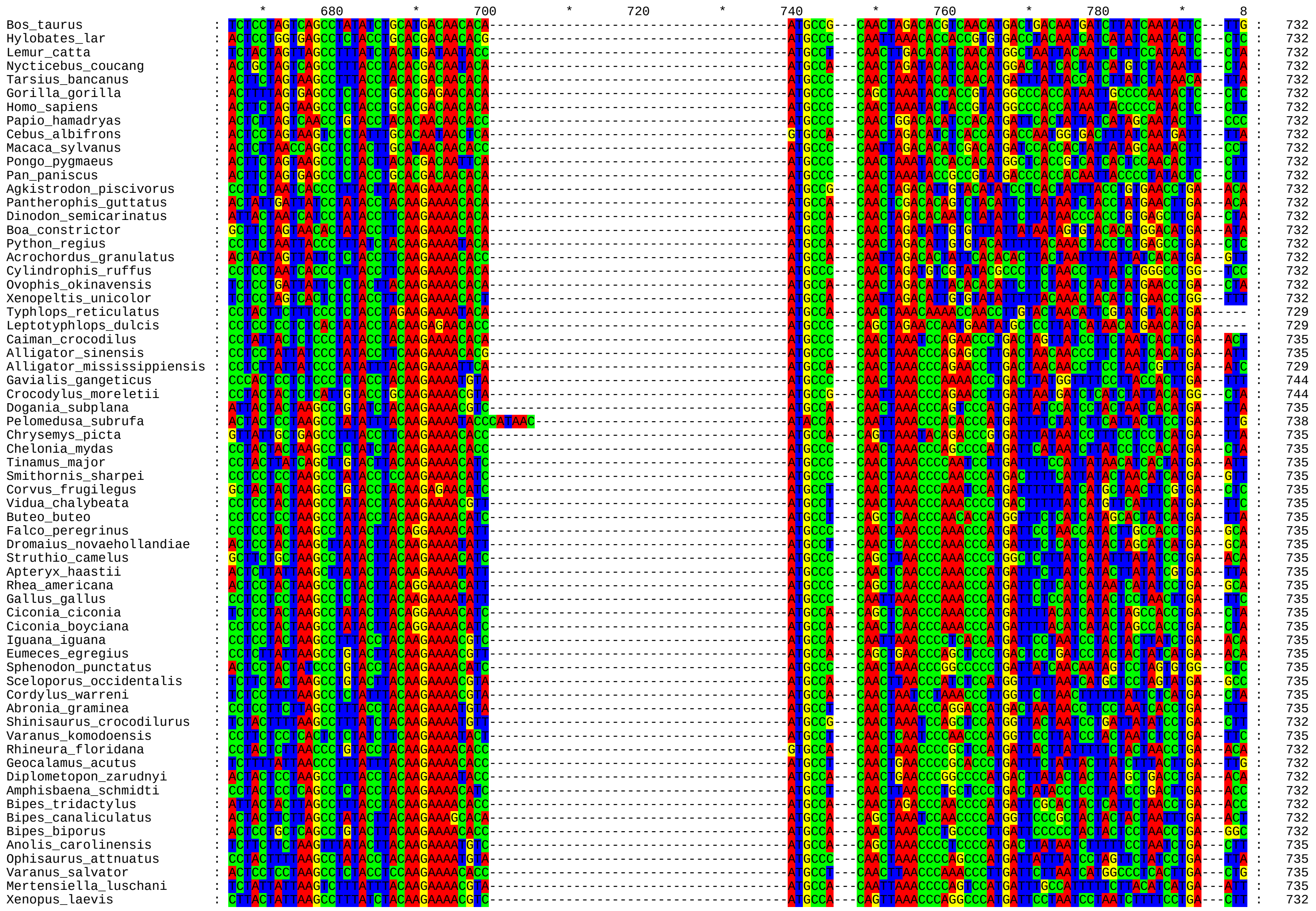
* 680 * 700 * 720 * 740 * 760 * 780 * 8
Bos_taurus : 732
Hylobates_lar : 732
Lemur_catta : 732
Nycticebus_coucang : 732
Tarsius_bancanus : 732
Gorilla_gorilla : 732
Homo_sapiens : 732
Papio_hamadryas : 732
Cebus_albifrons : 732
Macaca_sylvanus : 732
Pongo_pygmaeus : 732
Pan_paniscus : 732
Agkistrodon_piscivorus : 732
Pantherophis_guttatus : 732
Dinodon_semicarinatus : 732
Boa_constrictor : 732
Python_regius : 732
Acrochordus_granulatus : 732
Cylindrophis_ruffus : 732
Ovophis_okinavensis : 732
Xenopeltis_unicolor : 732
Typhlops_reticulatus : 729
Leptotyphlops_dulcis : 729
Caiman_crocodilus : 735
Alligator_sinensis : 735
Alligator_mississippiensis : 729
Gavialis_gangeticus : 744
Crocodylus_moreletii : 744
Dogania_subplana : 735
Pelomedusa_subrufa : 738
Chrysemys_picta : 735
Chelonia_mydas : 735
Tinamus_major : 735
Smithornis_sharpei : 735
Corvus_frugilegus : 735
Vidua_chalybeata : 735
Buteo_buteo : 735
Falco_peregrinus : 735
Dromaius_novaehollandiae : 735
Struthio_camelus : 735
Apteryx_haastii : 735
Rhea_americana : 735
Gallus_gallus : 735
Ciconia_ciconia : 735
Ciconia_boyciana : 735
Iguana_iguana : 735
Eumeces_egregius : 735
Sphenodon_punctatus : 735
Sceloporus_occidentalis : 735
Cordylus_warreni : 735
Abronia_graminea : 735
Shinisaurus_crocodilurus : 732
Varanus_komodoensis : 735
Rhineura_floridana : 732
Geocalamus_acutus : 732
Diplometopon_zarudnyi : 732
Amphisbaena_schmidti : 732
Bipes_tridactylus : 732
Bipes_canaliculatus : 732
Bipes_biporus : 732
Anolis_carolinensis : 735
Ophisaurus_attnuatus : 735
Varanus_salvator : 735
Mertensiella_luschani : 735
Xenopus_laevis : 732

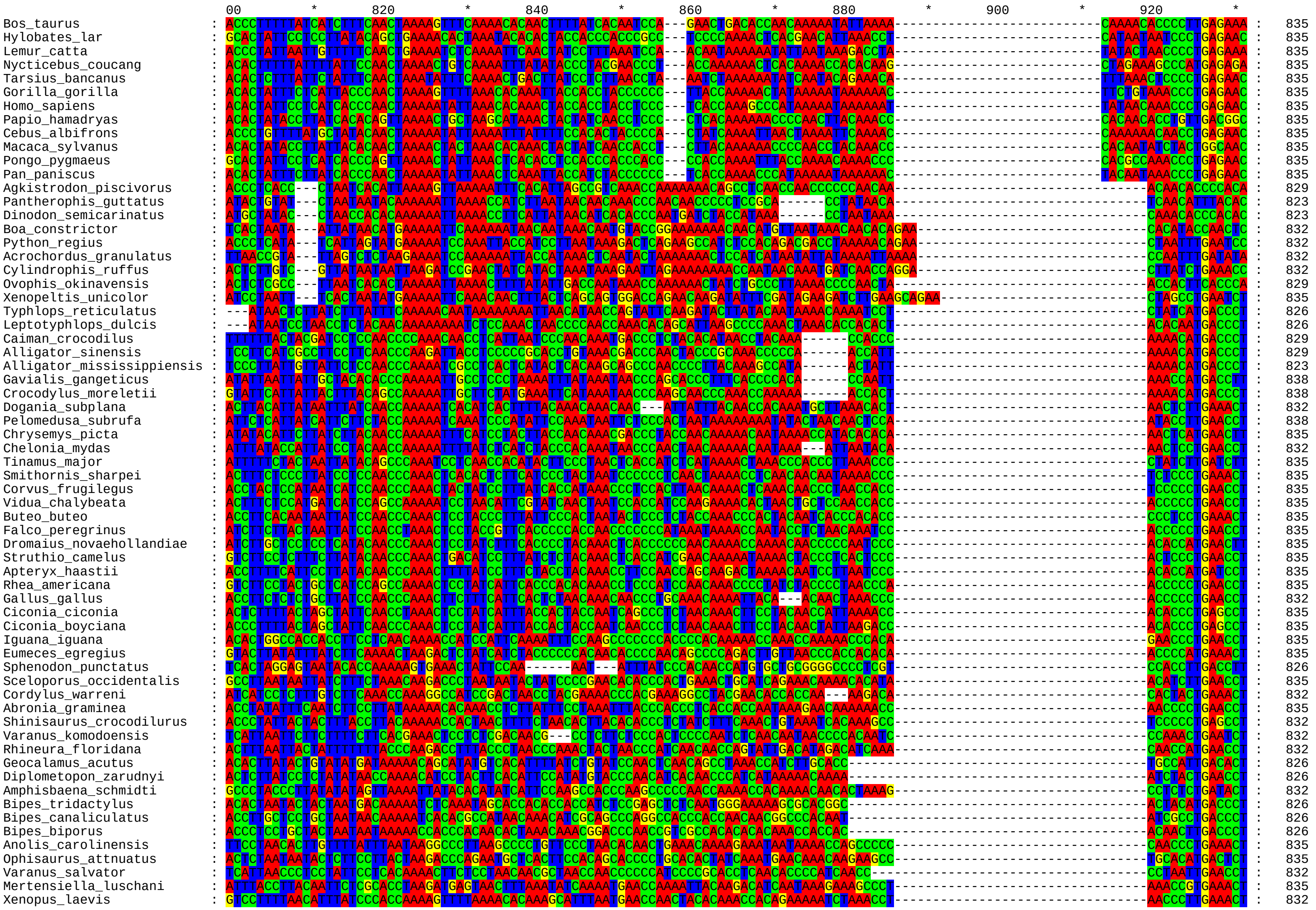
Bos_taurus 835
Hylobates_lar 835
Lemur_catta 835
Nycticebus_coucang 835
Tarsius_bancanus 835
Gorilla_gorilla 835
Homo_sapiens 835
Papio_hamadryas 835
Cebus_albifrons 835
Macaca_sylvanus 835
Pongo_pygmaeus 835
Pan_paniscus 835
Agkistrodon_piscivorus 829
Pantherophis_guttatus 823
Dinodon_semicarinatus 823
Boa_constrictor 832
Python_regius 832
Acrochordus_granulatus 832
Cylindrophis_ruffus 832
Ovophis_okinavensis 829
Xenopeltis_unicolor 835
Typhlops_reticulatus 826
Leptotyphlops_dulcis 826
Caiman_crocodilus 829
Alligator_sinensis 829
Alligator_mississippiensis 823
Gavialis_gangeticus 838
Crocodylus_moreletii 838
Dogania_subplana 832
Pelomedusa_subrufa 838
Chrysemys_picta 835
Chelonia_mydas 832
Tinamus_major 835
Smithornis_sharpei 835
Corvus_frugilegus 835
Vidua_chalybeata 835
Buteo_buteo 835
Falco_peregrinus 835
Dromaius_novaehollandiae 835
Struthio_camelus 835
Apteryx_haastii 835
Rhea_americana 835
Gallus_gallus 832
Ciconia_ciconia 835
Ciconia_boyciana 835
Iguana_iguana 835
Eumeces_egregius 835
Sphenodon_punctatus 826
Sceloporus_occidentalis 835
Cordylus_warreni 832
Abronia_graminea 835
Shinisaurus_crocodilurus 832
Varanus_komodoensis 832
Rhineura_floridana 832
Geocalamus_acutus 826
Diplometopon_zarudnyi 826
Amphisbaena_schmidti 832
Bipes_tridactylus 826
Bipes_canaliculatus 826
Bipes_biporus 826
Anolis_carolinensis 835
Ophisaurus_attnuatus 835
Varanus_salvator 832
Mertensiella_luschani 835
Xenopus_laevis 832

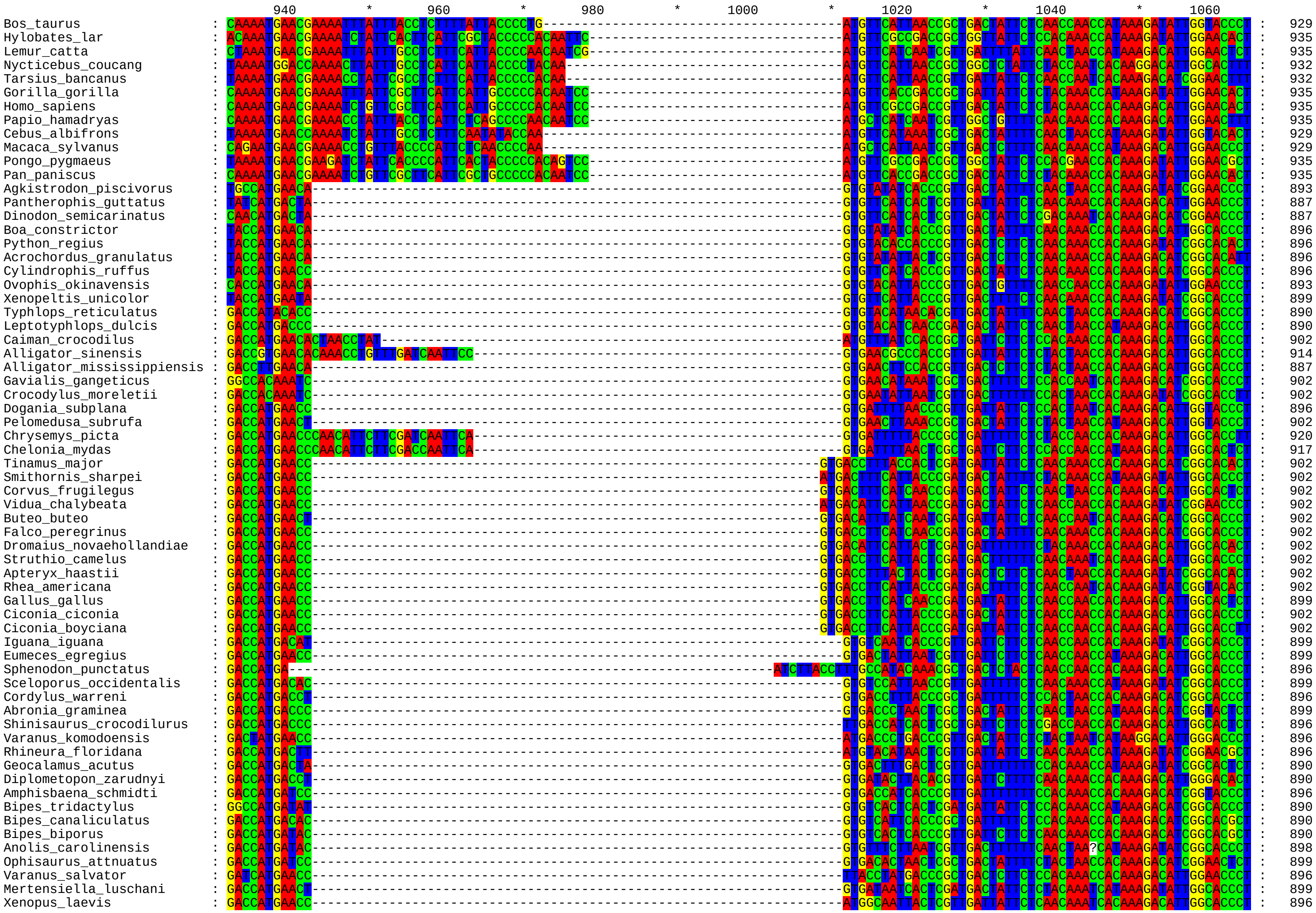

940
960
980
1000
1020
1040
1060
Bos_taurus 929
Hylobates_lar 935
Lemur_catta 935
Nycticebus_coucang 932
Tarsius_bancanus 932
Gorilla_gorilla 935
Homo_sapiens 935
Papio_hamadryas 935
Cebus_albifrons 929
Macaca_sylvanus 929
Pongo_pygmaeus 935
Pan_paniscus 935
Agkistrodon_piscivorus 893
Pantherophis_guttatus 887
Dinodon_semicarinatus 887
Boa_constrictor 896
Python_regius 896
Acrochordus_granulatus 896
Cylindrophis_ruffus 896
Ovophis_okinavensis 893
Xenopeltis_unicolor 899
Typhlops_reticulatus 890
Leptotyphlops_dulcis 890
Caiman_crocodilus 902
Alligator_sinensis 914
Alligator_mississippiensis 887
Gavialis_gangeticus 902
Crocodylus_moreletii 902
Dogania_subplana 896
Pelomedusa_subrufa 902
Chrysemys_picta 920
Chelonia_mydas 917
Tinamus_major 902
Smithornis_sharpei 902
Corvus_frugilegus 902
Vidua_chalybeata 902
Buteo_buteo 902
Falco_peregrinus 902
Dromaius_novaehollandiae 902
Struthio_camelus 902
Apteryx_haastii 902
Rhea_americana 902
Gallus_gallus 899
Ciconia_ciconia 902
Ciconia_boyciana 902
Iguana_iguana 899
Eumeces_egregius 899
Sphenodon_punctatus 896
Sceloporus_occidentalis 899
Cordylus_warreni 896
Abronia_graminea 899
Shinisaurus_crocodilurus 896
Varanus_komodoensis 896
Rhineura_floridana 896
Geocalamus_acutus 890
Diplometopon_zarudnyi 890
Amphisbaena_schmidti 896
Bipes_tridactylus 890
Bipes_canaliculatus 890
Bipes_biporus 890
Anolis_carolinensis 898
Ophisaurus_attnuatus 899
Varanus_salvator 896
Mertensiella_luschani 899
Xenopus_laevis 896

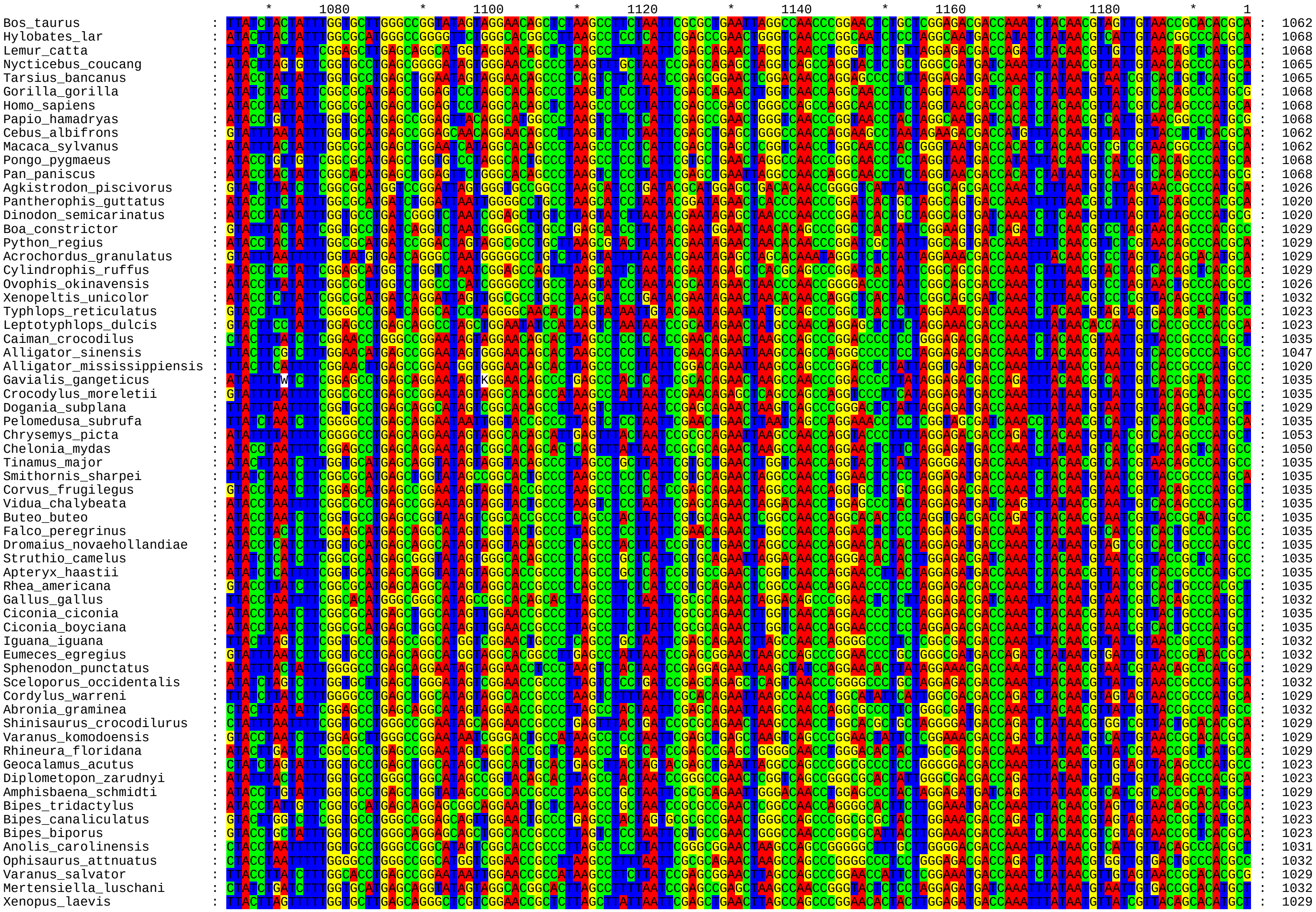

1080 1100 1120 1140 1160 1180
Bos_taurus 1062
Hylobates_lar 1068
Lemur_catta 1068
Nycticebus_coucang 1065
Tarsius_bancanus 1065
Gorilla_gorilla 1068
Homo_sapiens 1068
Papio_hamadryas 1068
Cebus_albifrons 1062
Macaca_sylvanus 1062
Pongo_pygmaeus 1068
Pan_paniscus 1068
Agkistrodon_piscivorus 1026
Pantherophis_guttatus 1020
Dinodon_semicarinatus 1020
Boa_constrictor 1029
Python_regius 1029
Acrochordus_granulatus 1029
Cylindrophis_ruffus 1029
Ovophis_okinavensis 1026
Xenopeltis_unicolor 1032
Typhlops_reticulatus 1023
Leptotyphlops_dulcis 1023
Caiman_crocodilus 1035
Alligator_sinensis 1047
Alligator_mississippiensis 1020
Gavialis_gangeticus 1035
Crocodylus_moreletii 1035
Dogania_subplana 1029
Pelomedusa_subrufa 1035
Chrysemys_picta 1053
Chelonia_mydas 1050
Tinamus_major 1035
Smithornis_sharpei 1035
Corvus_frugilegus 1035
Vidua_chalybeata 1035
Buteo_buteo 1035
Falco_peregrinus 1035
Dromaius_novaehollandiae 1035
Struthio_camelus 1035
Apteryx_haastii 1035
Rhea_americana 1035
Gallus_gallus 1032
Ciconia_ciconia 1035
Ciconia_boyciana 1035
Iguana_iguana 1032
Eumeces_egregius 1032
Sphenodon_punctatus 1029
Sceloporus_occidentalis 1032
Cordylus_warreni 1029
Abronia_graminea 1032
Shinisaurus_crocodilurus 1029
Varanus_komodoensis 1029
Rhineura_floridana 1029
Geocalamus_acutus 1023
Diplometopon_zarudnyi 1023
Amphisbaena_schmidti 1029
Bipes_tridactylus 1023
Bipes_canaliculatus 1023
Bipes_biporus 1023
Anolis_carolinensis 1031
Ophisaurus_attnuatus 1032
Varanus_salvator 1029
Mertensiella_luschani 1032
Xenopus_laevis 1029

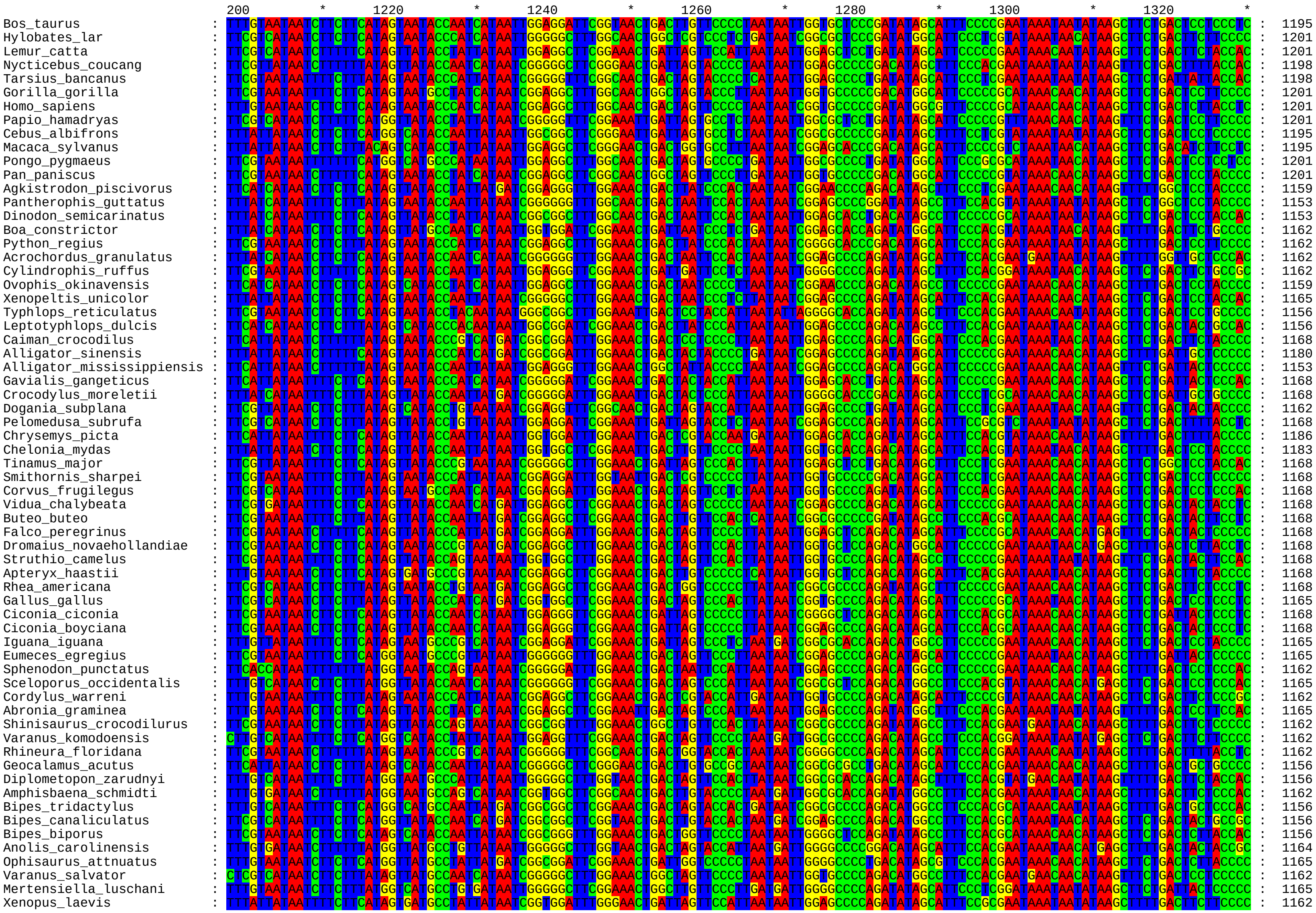

200 * 1220 * 1240 * 1260 * 1280 * 1300 * 1320 *
Bos_taurus : 1195
Hylobates_lar : 1201
Lemur_catta : 1201
Nycticebus_coucang : 1198
Tarsius_bancanus : 1198
Gorilla_gorilla : 1201
Homo_sapiens : 1201
Papio_hamadryas : 1201
Cebus_albifrons : 1195
Macaca_sylvanus : 1195
Pongo_pygmaeus : 1201
Pan_paniscus : 1201
Agkistrodon_piscivorus : 1159
Pantherophis_guttatus : 1153
Dinodon_semicarinatus : 1153
Boa_constrictor : 1162
Python_regius : 1162
Acrochordus_granulatus : 1162
Cylindrophis_ruffus : 1162
Ovophis_okinavensis : 1159
Xenopeltis_unicolor : 1165
Typhlops_reticulatus : 1156
Leptotyphlops_dulcis : 1156
Caiman_crocodilus : 1168
Alligator_sinensis : 1180
Alligator_mississippiensis : 1153
Gavialis_gangeticus : 1168
Crocodylus_moreletii : 1168
Dogania_subplana : 1162
Pelomedusa_subrufa : 1168
Chrysemys_picta : 1186
Chelonia_mydas : 1183
Tinamus_major : 1168
Smithornis_sharpei : 1168
Corvus_frugilegus : 1168
Vidua_chalybeata : 1168
Buteo_buteo : 1168
Falco_peregrinus : 1168
Dromaius_novaehollandiae : 1168
Struthio_camelus : 1168
Apteryx_haastii : 1168
Rhea_americana : 1168
Gallus_gallus : 1165
Ciconia_ciconia : 1168
Ciconia_boyciana : 1168
Iguana_iguana : 1165
Eumeces_egregius : 1165
Sphenodon_punctatus : 1162
Sceloporus_occidentalis : 1165
Cordylus_warreni : 1162
Abronia_graminea : 1165
Shinisaurus_crocodilurus : 1162
Varanus_komodoensis : 1162
Rhineura_floridana : 1162
Geocalamus_acutus : 1156
Diplometopon_zarudnyi : 1156
Amphisbaena_schmidti : 1162
Bipes_tridactylus : 1156
Bipes_canaliculatus : 1156
Bipes_biporus : 1156
Anolis_carolinensis : 1164
Ophisaurus_attnuatus : 1165
Varanus_salvator : 1162
Mertensiella_luschani : 1165
Xenopus_laevis : 1162

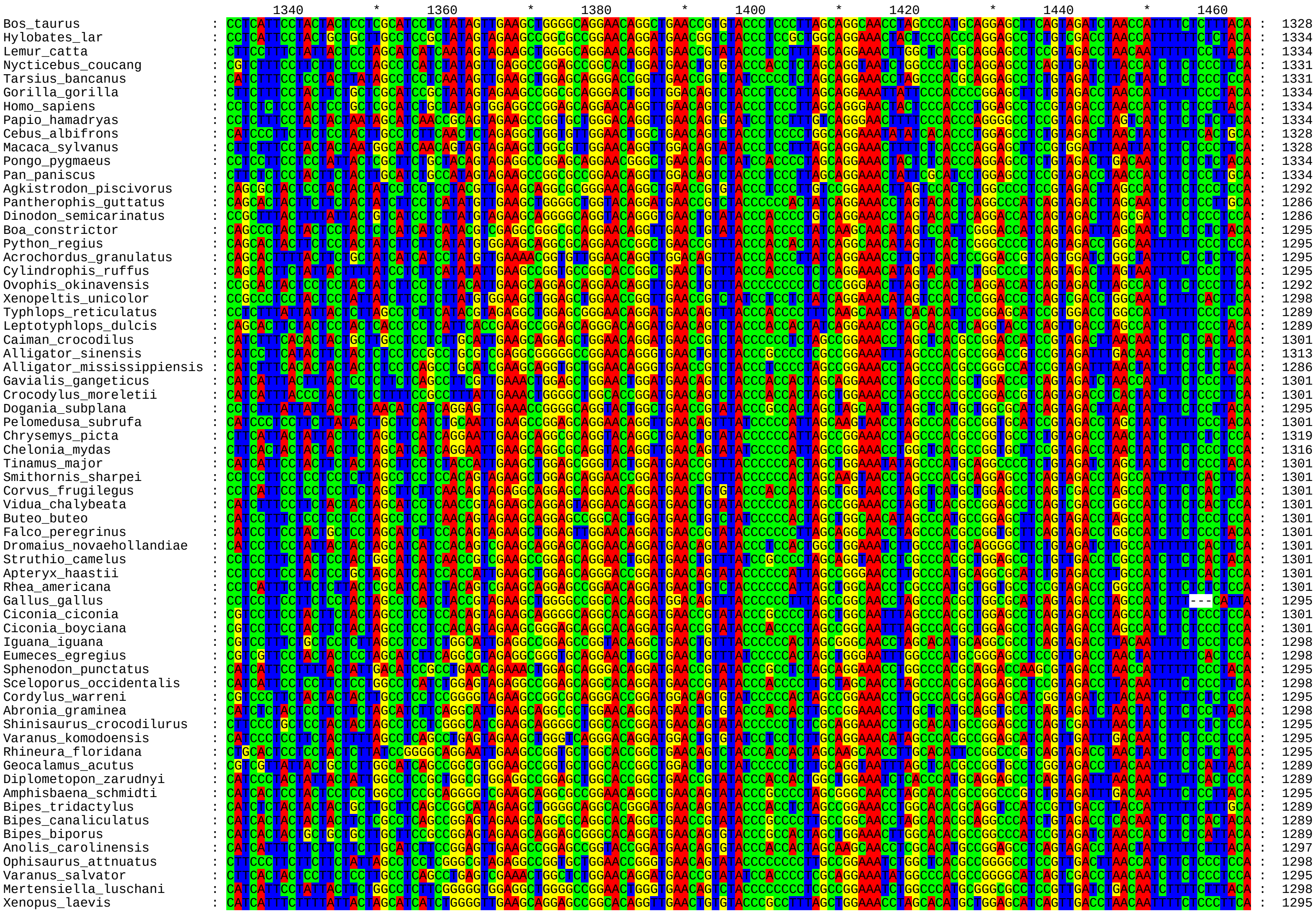
1340 * 1360 * 1380 * 1400 * 1420 * 1440 * 1460
Bos_taurus : 1328
Hylobates_lar : 1334
Lemur_catta : 1334
Nycticebus_coucang : 1331
Tarsius_bancanus : 1331
Gorilla_gorilla : 1334
Homo_sapiens : 1334
Papio_hamadryas : 1334
Cebus_albifrons : 1328
Macaca_sylvanus : 1328
Pongo_pygmaeus : 1334
Pan_paniscus : 1334
Agkistrodon_piscivorus : 1292
Pantherophis_guttatus : 1286
Dinodon_semicarinatus : 1286
Boa_constrictor : 1295
Python_regius : 1295
Acrochordus_granulatus : 1295
Cylindrophis_ruffus : 1295
Ovophis_okinavensis : 1292
Xenopeltis_unicolor : 1298
Typhlops_reticulatus : 1289
Leptotyphlops_dulcis : 1289
Caiman_crocodilus : 1301
Alligator_sinensis : 1313
Alligator_mississippiensis : 1286
Gavialis_gangeticus : 1301
Crocodylus_moreletii : 1301
Dogania_subplana : 1295
Pelomedusa_subrufa : 1301
Chrysemys_picta : 1319
Chelonia_mydas : 1316
Tinamus_major : 1301
Smithornis_sharpei : 1301
Corvus_frugilegus : 1301
Vidua_chalybeata : 1301
Buteo_buteo : 1301
Falco_peregrinus : 1301
Dromaius_novaehollandiae : 1301
Struthio_camelus : 1301
Apteryx_haastii : 1301
Rhea_americana : 1301
Gallus_gallus : 1295
Ciconia_ciconia : 1301
Ciconia_boyciana : 1301
Iguana_iguana : 1298
Eumeces_egregius : 1298
Sphenodon_punctatus : 1295
Sceloporus_occidentalis : 1298
Cordylus_warreni : 1295
Abronia_graminea : 1298
Shinisaurus_crocodilurus : 1295
Varanus_komodoensis : 1295
Rhineura_floridana : 1295
Geocalamus_acutus : 1289
Diplometopon_zarudnyi : 1289
Amphisbaena_schmidti : 1295
Bipes_tridactylus : 1289
Bipes_canaliculatus : 1289
Bipes_biporus : 1289
Anolis_carolinensis : 1297
Ophisaurus_attnuatus : 1298
Varanus_salvator : 1295
Mertensiella_luschani : 1298
Xenopus_laevis : 1295

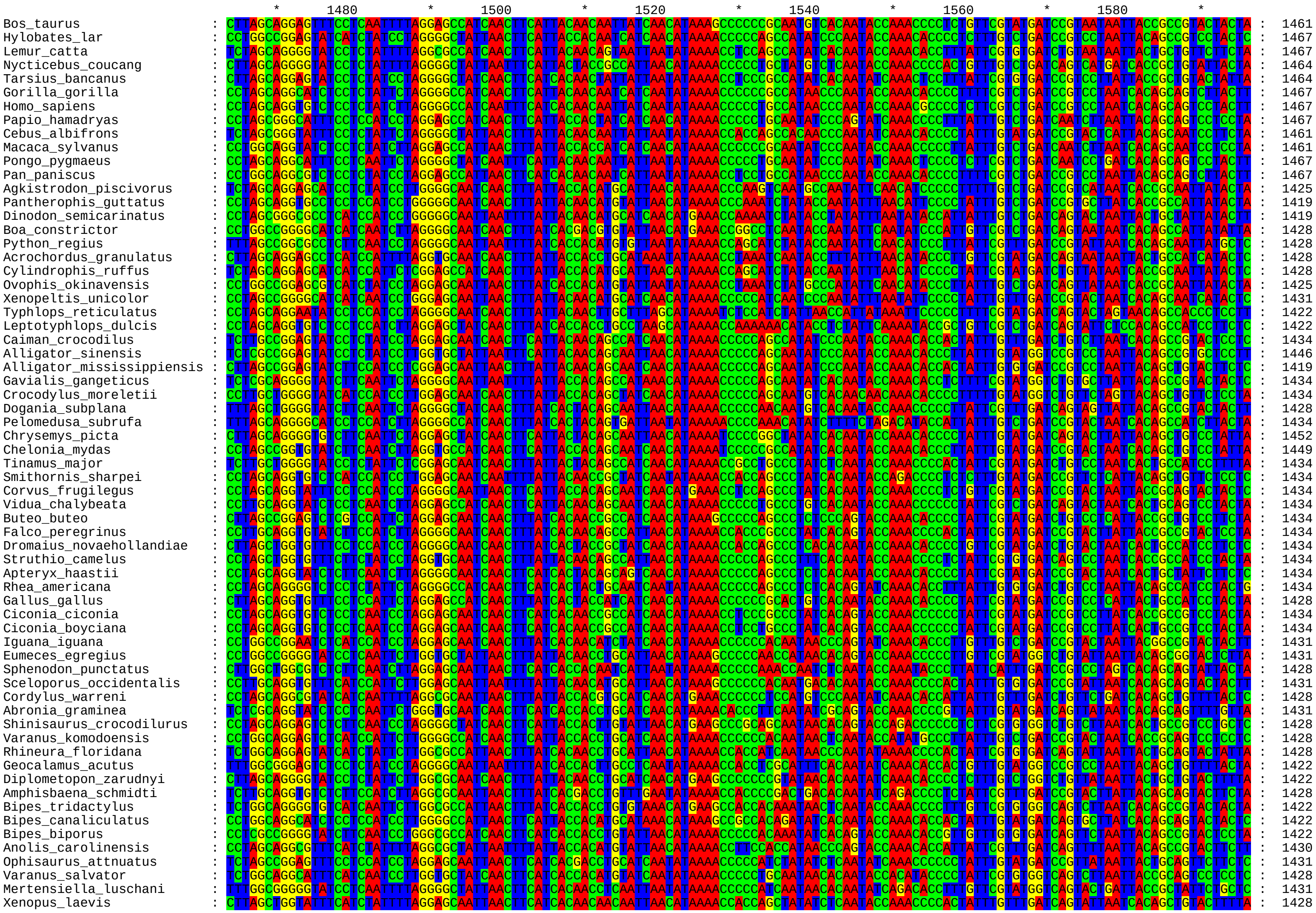

1480
1500
1520
1540
1560
1580
Bos_taurus 1461
Hylobates_lar 1467
Lemur_catta 1467
Nycticebus_coucang 1464
Tarsius_bancanus 1464
Gorilla_gorilla 1467
Homo_sapiens 1467
Papio_hamadryas 1467
Cebus_albifrons 1461
Macaca_sylvanus 1461
Pongo_pygmaeus 1467
Pan_paniscus 1467
Agkistrodon_piscivorus 1425
Pantherophis_guttatus 1419
Dinodon_semicarinatus 1419
Boa_constrictor 1428
Python_regius 1428
Acrochordus_granulatus 1428
Cylindrophis_ruffus 1428
Ovophis_okinavensis 1425
Xenopeltis_unicolor 1431
Typhlops_reticulatus 1422
Leptotyphlops_dulcis 1422
Caiman_crocodilus 1434
Alligator_sinensis 1446
Alligator_mississippiensis 1419
Gavialis_gangeticus 1434
Crocodylus_moreletii 1434
Dogania_subplana 1428
Pelomedusa_subrufa 1434
Chrysemys_picta 1452
Chelonia_mydas 1449
Tinamus_major 1434
Smithornis_sharpei 1434
Corvus_frugilegus 1434
Vidua_chalybeata 1434
Buteo_buteo 1434
Falco_peregrinus 1434
Dromaius_novaehollandiae 1434
Struthio_camelus 1434
Apteryx_haastii 1434
Rhea_americana 1434
Gallus_gallus 1428
Ciconia_ciconia 1434
Ciconia_boyciana 1434
Iguana_iguana 1431
Eumeces_egregius 1431
Sphenodon_punctatus 1428
Sceloporus_occidentalis 1431
Cordylus_warreni 1428
Abronia_graminea 1431
Shinisaurus_crocodilurus 1428
Varanus_komodoensis 1428
Rhineura_floridana 1428
Geocalamus_acutus 1422
Diplometopon_zarudnyi 1422
Amphisbaena_schmidti 1428
Bipes_tridactylus 1422
Bipes_canaliculatus 1422
Bipes_biporus 1422
Anolis_carolinensis 1430
Ophisaurus_attnuatus 1431
Varanus_salvator 1428
Mertensiella_luschani 1431
Xenopus_laevis 1428

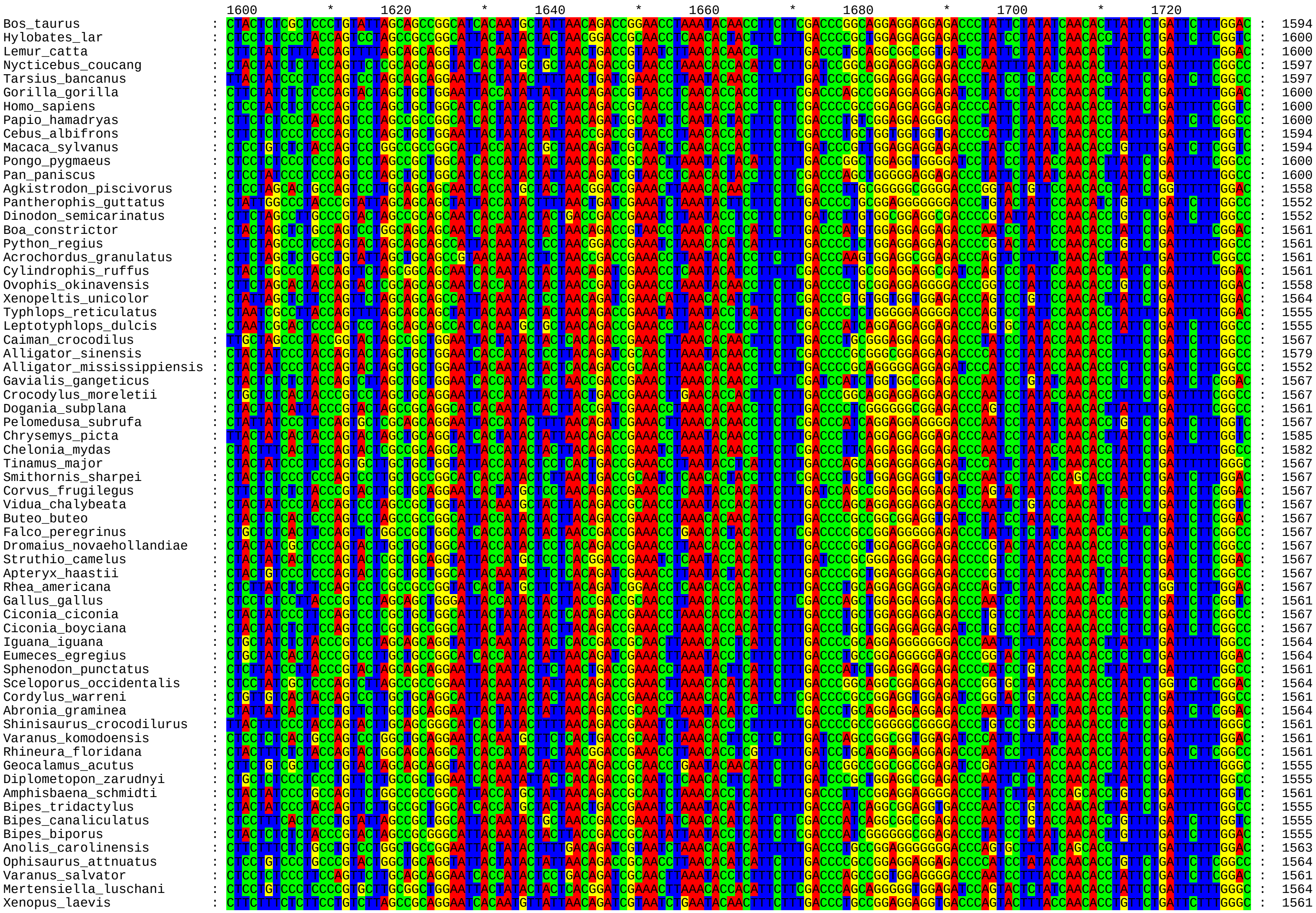
1600 * 1620 * 1640 * 1660 * 1680 * 1700 * 1720
Bos_taurus : 1594
Hylobates_lar : 1600
Lemur_catta : 1600
Nycticebus_coucang : 1597
Tarsius_bancanus : 1597
Gorilla_gorilla : 1600
Homo_sapiens : 1600
Papio_hamadryas : 1600
Cebus_albifrons : 1594
Macaca_sylvanus : 1594
Pongo_pygmaeus : 1600
Pan_paniscus : 1600
Agkistrodon_piscivorus : 1558
Pantherophis_guttatus : 1552
Dinodon_semicarinatus : 1552
Boa_constrictor : 1561
Python_regius : 1561
Acrochordus_granulatus : 1561
Cylindrophis_ruffus : 1561
Ovophis_okinavensis : 1558
Xenopeltis_unicolor : 1564
Typhlops_reticulatus : 1555
Leptotyphlops_dulcis : 1555
Caiman_crocodilus : 1567
Alligator_sinensis : 1579
Alligator_mississippiensis : 1552
Gavialis_gangeticus : 1567
Crocodylus_moreletii : 1567
Dogania_subplana : 1561
Pelomedusa_subrufa : 1567
Chrysemys_picta : 1585
Chelonia_mydas : 1582
Tinamus_major : 1567
Smithornis_sharpei : 1567
Corvus_frugilegus : 1567
Vidua_chalybeata : 1567
Buteo_buteo : 1567
Falco_peregrinus : 1567
Dromaius_novaehollandiae : 1567
Struthio_camelus : 1567
Apteryx_haastii : 1567
Rhea_americana : 1567
Gallus_gallus : 1561
Ciconia_ciconia : 1567
Ciconia_boyciana : 1567
Iguana_iguana : 1564
Eumeces_egregius : 1564
Sphenodon_punctatus : 1561
Sceloporus_occidentalis : 1564
Cordylus_warreni : 1561
Abronia_graminea : 1564
Shinisaurus_crocodilurus : 1561
Varanus_komodoensis : 1561
Rhineura_floridana : 1561
Geocalamus_acutus : 1555
Diplometopon_zarudnyi : 1555
Amphisbaena_schmidti : 1561
Bipes_tridactylus : 1555
Bipes_canaliculatus : 1555
Bipes_biporus : 1555
Anolis_carolinensis : 1563
Ophisaurus_attnuatus : 1564
Varanus_salvator : 1561
Mertensiella_luschani : 1564
Xenopus_laevis : 1561

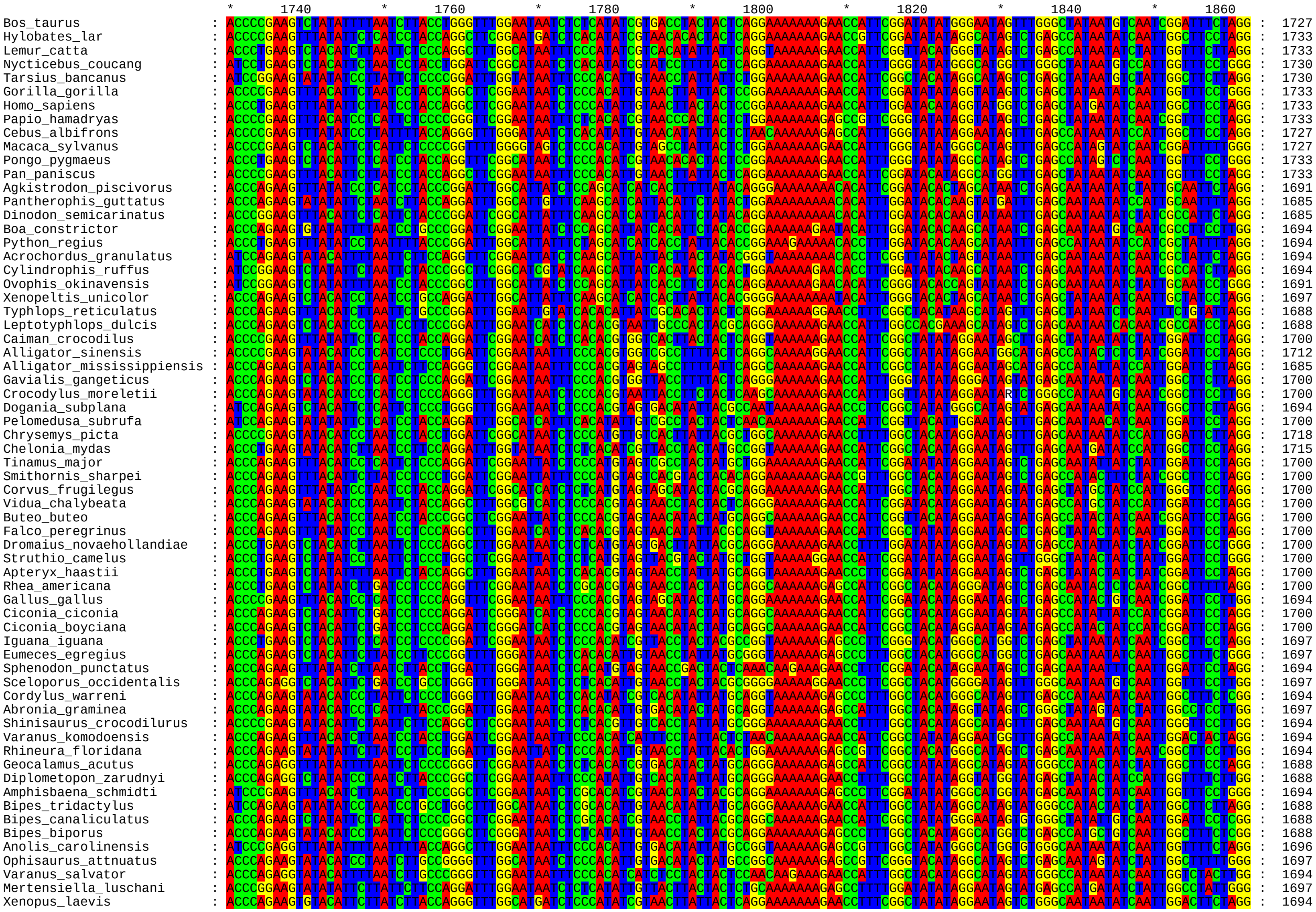
* 1740 * 1760 * 1780 * 1800 * 1820 * 1840 * 1860
Bos_taurus : 1727
Hylobates_lar : 1733
Lemur_catta : 1733
Nycticebus_coucang : 1730
Tarsius_bancanus : 1730
Gorilla_gorilla : 1733
Homo_sapiens : 1733
Papio_hamadryas : 1733
Cebus_albifrons : 1727
Macaca_sylvanus : 1727
Pongo_pygmaeus : 1733
Pan_paniscus : 1733
Agkistrodon_piscivorus : 1691
Pantherophis_guttatus : 1685
Dinodon_semicarinatus : 1685
Boa_constrictor : 1694
Python_regius : 1694
Acrochordus_granulatus : 1694
Cylindrophis_ruffus : 1694
Ovophis_okinavensis : 1691
Xenopeltis_unicolor : 1697
Typhlops_reticulatus : 1688
Leptotyphlops_dulcis : 1688
Caiman_crocodilus : 1700
Alligator_sinensis : 1712
Alligator_mississippiensis : 1685
Gavialis_gangeticus : 1700
Crocodylus_moreletii : 1700
Dogania_subplana : 1694
Pelomedusa_subrufa : 1700
Chrysemys_picta : 1718
Chelonia_mydas : 1715
Tinamus_major : 1700
Smithornis_sharpei : 1700
Corvus_frugilegus : 1700
Vidua_chalybeata : 1700
Buteo_buteo : 1700
Falco_peregrinus : 1700
Dromaius_novaehollandiae : 1700
Struthio_camelus : 1700
Apteryx_haastii : 1700
Rhea_americana : 1700
Gallus_gallus : 1694
Ciconia_ciconia : 1700
Ciconia_boyciana : 1700
Iguana_iguana : 1697
Eumeces_egregius : 1697
Sphenodon_punctatus : 1694
Sceloporus_occidentalis : 1697
Cordylus_warreni : 1694
Abronia_graminea : 1697
Shinisaurus_crocodilurus : 1694
Varanus_komodoensis : 1694
Rhineura_floridana : 1694
Geocalamus_acutus : 1688
Diplometopon_zarudnyi : 1688
Amphisbaena_schmidti : 1694
Bipes_tridactylus : 1688
Bipes_canaliculatus : 1688
Bipes_biporus : 1688
Anolis_carolinensis : 1696
Ophisaurus_attnuatus : 1697
Varanus_salvator : 1694
Mertensiella_luschani : 1697
Xenopus_laevis : 1694

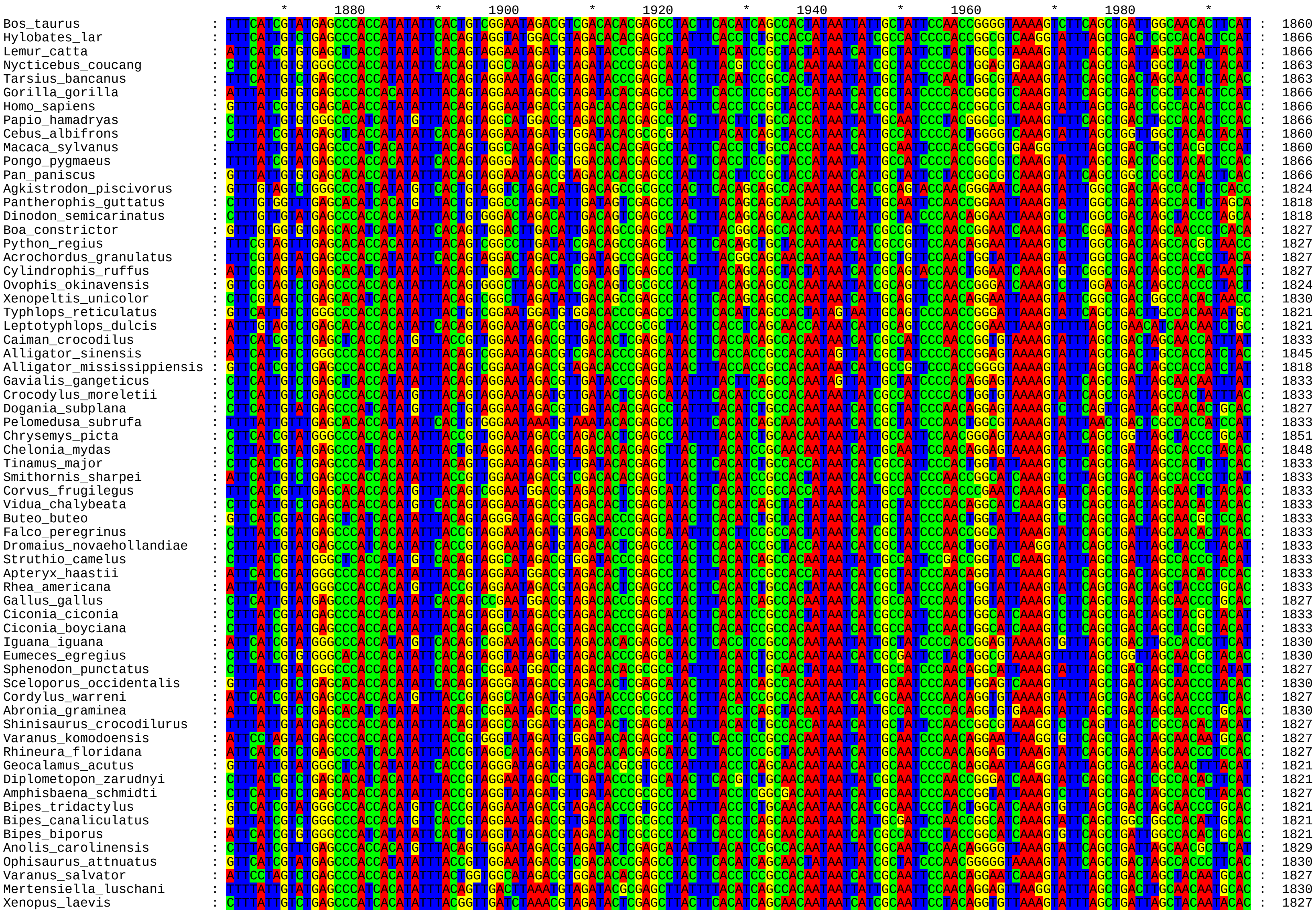
1880
1900
1920
1940
1960
1980
Bos_taurus 1860
Hylobates_lar 1866
Lemur_catta 1866
Nycticebus_coucang 1863
Tarsius_bancanus 1863
Gorilla_gorilla 1866
Homo_sapiens 1866
Papio_hamadryas 1866
Cebus_albifrons 1860
Macaca_sylvanus 1860
Pongo_pygmaeus 1866
Pan_paniscus 1866
Agkistrodon_piscivorus 1824
Pantherophis_guttatus 1818
Dinodon_semicarinatus 1818
Boa_constrictor 1827
Python_regius 1827
Acrochordus_granulatus 1827
Cylindrophis_ruffus 1827
Ovophis_okinavensis 1824
Xenopeltis_unicolor 1830
Typhlops_reticulatus 1821
Leptotyphlops_dulcis 1821
Caiman_crocodilus 1833
Alligator_sinensis 1845
Alligator_mississippiensis 1818
Gavialis_gangeticus 1833
Crocodylus_moreletii 1833
Dogania_subplana 1827
Pelomedusa_subrufa 1833
Chrysemys_picta 1851
Chelonia_mydas 1848
Tinamus_major 1833
Smithornis_sharpei 1833
Corvus_frugilegus 1833
Vidua_chalybeata 1833
Buteo_buteo 1833
Falco_peregrinus 1833
Dromaius_novaehollandiae 1833
Struthio_camelus 1833
Apteryx_haastii 1833
Rhea_americana 1833
Gallus_gallus 1827
Ciconia_ciconia 1833
Ciconia_boyciana 1833
Iguana_iguana 1830
Eumeces_egregius 1830
Sphenodon_punctatus 1827
Sceloporus_occidentalis 1830
Cordylus_warreni 1827
Abronia_graminea 1830
Shinisaurus_crocodilurus 1827
Varanus_komodoensis 1827
Rhineura_floridana 1827
Geocalamus_acutus 1821
Diplometopon_zarudnyi 1821
Amphisbaena_schmidti 1827
Bipes_tridactylus 1821
Bipes_canaliculatus 1821
Bipes_biporus 1821
Anolis_carolinensis 1829
Ophisaurus_attnuatus 1830
Varanus_salvator 1827
Mertensiella_luschani 1830
Xenopus_laevis 1827

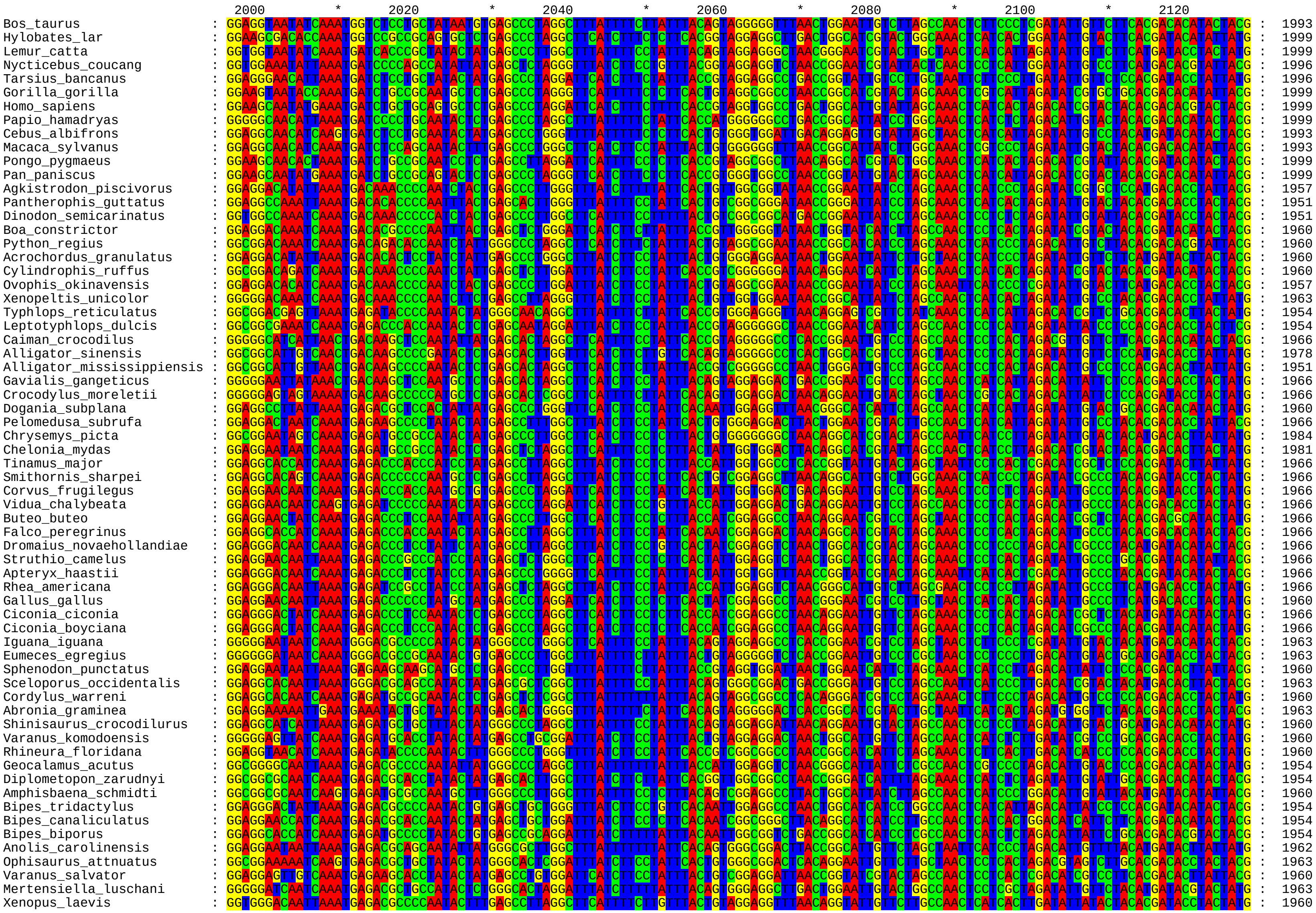
2000
2020
2040
2060
2080
2100
2120
Bos_taurus 1993
Hylobates_lar 1999
Lemur_catta 1999
Nycticebus_coucang 1996
Tarsius_bancanus 1996
Gorilla_gorilla 1999
Homo_sapiens 1999
Papio_hamadryas 1999
Cebus_albifrons 1993
Macaca_sylvanus 1993
Pongo_pygmaeus 1999
Pan_paniscus 1999
Agkistrodon_piscivorus 1957
Pantherophis_guttatus 1951
Dinodon_semicarinatus 1951
Boa_constrictor 1960
Python_regius 1960
Acrochordus_granulatus 1960
Cylindrophis_ruffus 1960
Ovophis_okinavensis 1957
Xenopeltis_unicolor 1963
Typhlops_reticulatus 1954
Leptotyphlops_dulcis 1954
Caiman_crocodilus 1966
Alligator_sinensis 1978
Alligator_mississippiensis 1951
Gavialis_gangeticus 1966
Crocodylus_moreletii 1966
Dogania_subplana 1960
Pelomedusa_subrufa 1966
Chrysemys_picta 1984
Chelonia_mydas 1981
Tinamus_major 1966
Smithornis_sharpei 1966
Corvus_frugilegus 1966
Vidua_chalybeata 1966
Buteo_buteo 1966
Falco_peregrinus 1966
Dromaius_novaehollandiae 1966
Struthio_camelus 1966
Apteryx_haastii 1966
Rhea_americana 1966
Gallus_gallus 1960
Ciconia_ciconia 1966
Ciconia_boyciana 1966
Iguana_iguana 1963
Eumeces_egregius 1963
Sphenodon_punctatus 1960
Sceloporus_occidentalis 1963
Cordylus_warreni 1960
Abronia_graminea 1963
Shinisaurus_crocodilurus 1960
Varanus_komodoensis 1960
Rhineura_floridana 1960
Geocalamus_acutus 1954
Diplometopon_zarudnyi 1954
Amphisbaena_schmidti 1960
Bipes_tridactylus 1954
Bipes_canaliculatus 1954
Bipes_biporus 1954
Anolis_carolinensis 1962
Ophisaurus_attnuatus 1963
Varanus_salvator 1960
Mertensiella_luschani 1963
Xenopus_laevis 1960

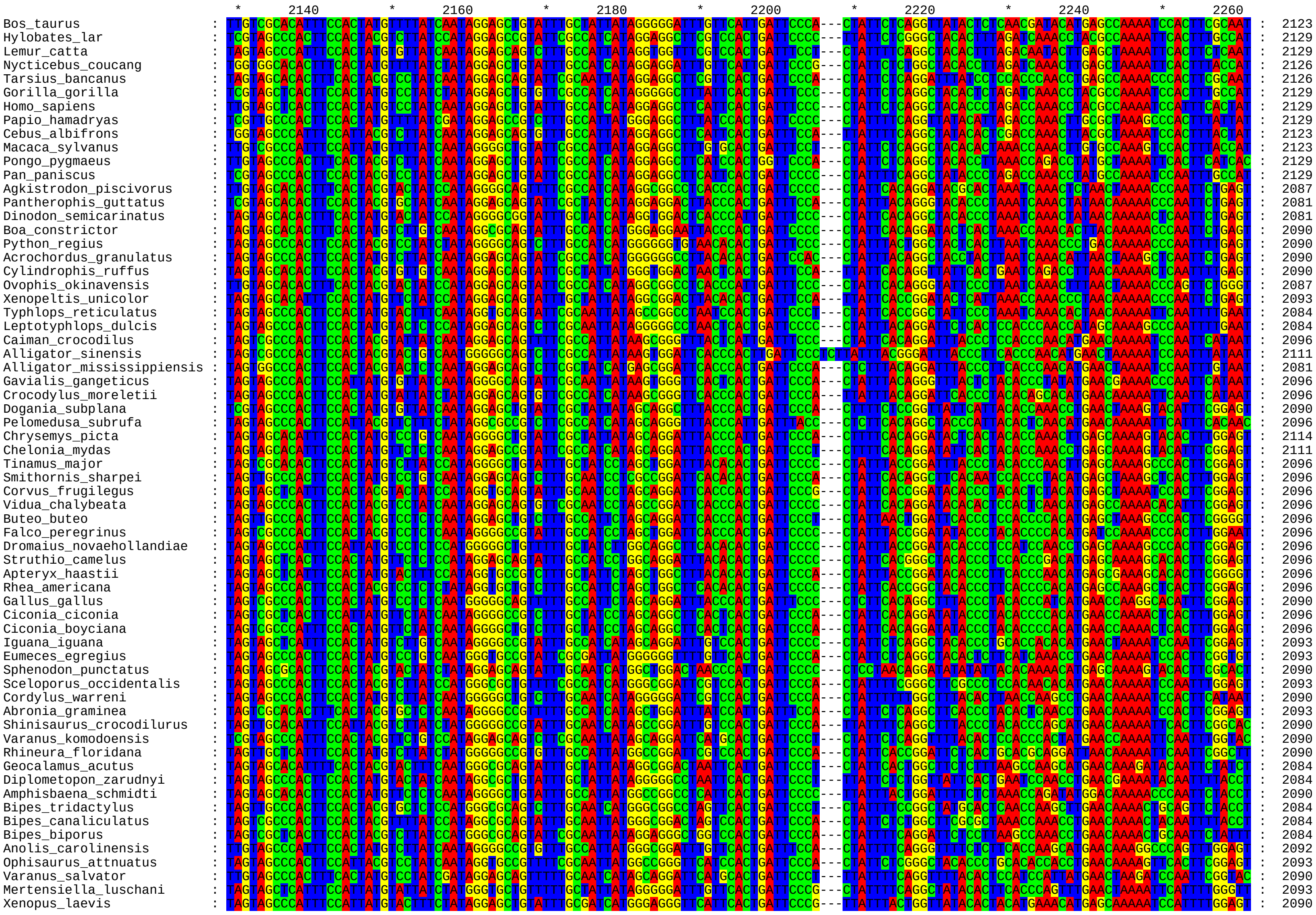

* 2140 * 2160 * 2180 * 2200 * 2220 * 2240 * 2260
Bos_taurus : 2123
Hylobates_lar : 2129
Lemur_catta : 2129
Nycticebus_coucang : 2126
Tarsius_bancanus : 2126
Gorilla_gorilla : 2129
Homo_sapiens : 2129
Papio_hamadryas : 2129
Cebus_albifrons : 2123
Macaca_sylvanus : 2123
Pongo_pygmaeus : 2129
Pan_paniscus : 2129
Agkistrodon_piscivorus : 2087
Pantherophis_guttatus : 2081
Dinodon_semicarinatus : 2081
Boa_constrictor : 2090
Python_regius : 2090
Acrochordus_granulatus : 2090
Cylindrophis_ruffus : 2090
Ovophis_okinavensis : 2087
Xenopeltis_unicolor : 2093
Typhlops_reticulatus : 2084
Leptotyphlops_dulcis : 2084
Caiman_crocodilus : 2096
Alligator_sinensis : 2111
Alligator_mississippiensis : 2081
Gavialis_gangeticus : 2096
Crocodylus_moreletii : 2096
Dogania_subplana : 2090
Pelomedusa_subrufa : 2096
Chrysemys_picta : 2114
Chelonia_mydas : 2111
Tinamus_major : 2096
Smithornis_sharpei : 2096
Corvus_frugilegus : 2096
Vidua_chalybeata : 2096
Buteo_buteo : 2096
Falco_peregrinus : 2096
Dromaius_novaehollandiae : 2096
Struthio_camelus : 2096
Apteryx_haastii : 2096
Rhea_americana : 2096
Gallus_gallus : 2090
Ciconia_ciconia : 2096
Ciconia_boyciana : 2096
Iguana_iguana : 2093
Eumeces_egregius : 2093
Sphenodon_punctatus : 2090
Sceloporus_occidentalis : 2093
Cordylus_warreni : 2090
Abronia_graminea : 2093
Shinisaurus_crocodilurus : 2090
Varanus_komodoensis : 2090
Rhineura_floridana : 2090
Geocalamus_acutus : 2084
Diplometopon_zarudnyi : 2084
Amphisbaena_schmidti : 2090
Bipes_tridactylus : 2084
Bipes_canaliculatus : 2084
Bipes_biporus : 2084
Anolis_carolinensis : 2092
Ophisaurus_attnuatus : 2093
Varanus_salvator : 2090
Mertensiella_luschani : 2093
Xenopus_laevis : 2090

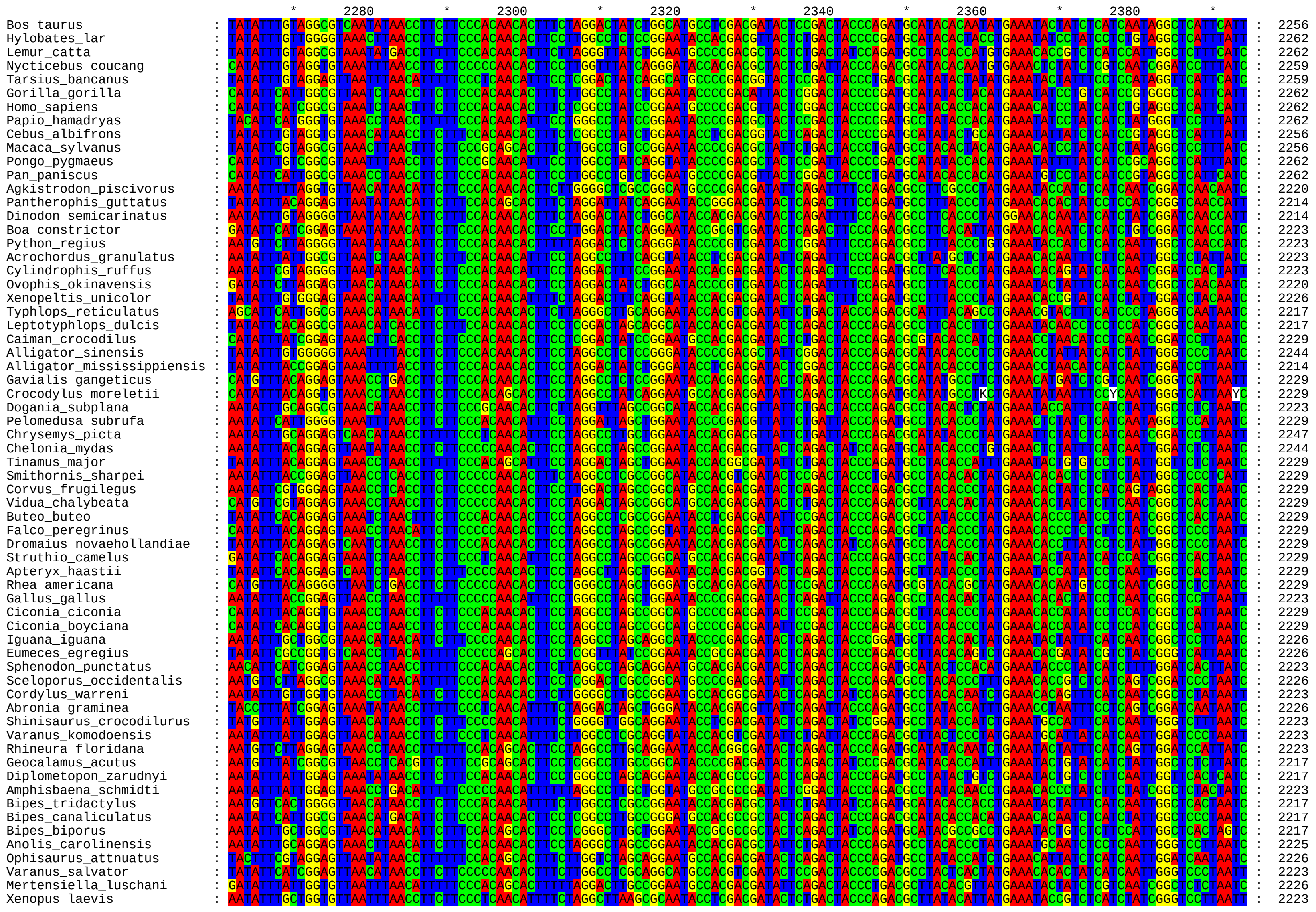
* 2280 * 2300 * 2320 * 2340 * 2360 * 2380 *
Bos_taurus : 2256
Hylobates_lar : 2262
Lemur_catta : 2262
Nycticebus_coucang : 2259
Tarsius_bancanus : 2259
Gorilla_gorilla : 2262
Homo_sapiens : 2262
Papio_hamadryas : 2262
Cebus_albifrons : 2256
Macaca_sylvanus : 2256
Pongo_pygmaeus : 2262
Pan_paniscus : 2262
Agkistrodon_piscivorus : 2220
Pantherophis_guttatus : 2214
Dinodon_semicarinatus : 2214
Boa_constrictor : 2223
Python_regius : 2223
Acrochordus_granulatus : 2223
Cylindrophis_ruffus : 2223
Ovophis_okinavensis : 2220
Xenopeltis_unicolor : 2226
Typhlops_reticulatus : 2217
Leptotyphlops_dulcis : 2217
Caiman_crocodilus : 2229
Alligator_sinensis : 2244
Alligator_mississippiensis : 2214
Gavialis_gangeticus : 2229
Crocodylus_moreletii : 2229
Dogania_subplana : 2223
Pelomedusa_subrufa : 2229
Chrysemys_picta : 2247
Chelonia_mydas : 2244
Tinamus_major : 2229
Smithornis_sharpei : 2229
Corvus_frugilegus : 2229
Vidua_chalybeata : 2229
Buteo_buteo : 2229
Falco_peregrinus : 2229
Dromaius_novaehollandiae : 2229
Struthio_camelus : 2229
Apteryx_haastii : 2229
Rhea_americana : 2229
Gallus_gallus : 2223
Ciconia_ciconia : 2229
Ciconia_boyciana : 2229
Iguana_iguana : 2226
Eumeces_egregius : 2226
Sphenodon_punctatus : 2223
Sceloporus_occidentalis : 2226
Cordylus_warreni : 2223
Abronia_graminea : 2226
Shinisaurus_crocodilurus : 2223
Varanus_komodoensis : 2223
Rhineura_floridana : 2223
Geocalamus_acutus : 2217
Diplometopon_zarudnyi : 2217
Amphisbaena_schmidti : 2223
Bipes_tridactylus : 2217
Bipes_canaliculatus : 2217
Bipes_biporus : 2217
Anolis_carolinensis : 2225
Ophisaurus_attnuatus : 2226
Varanus_salvator : 2223
Mertensiella_luschani : 2226
Xenopus_laevis : 2223

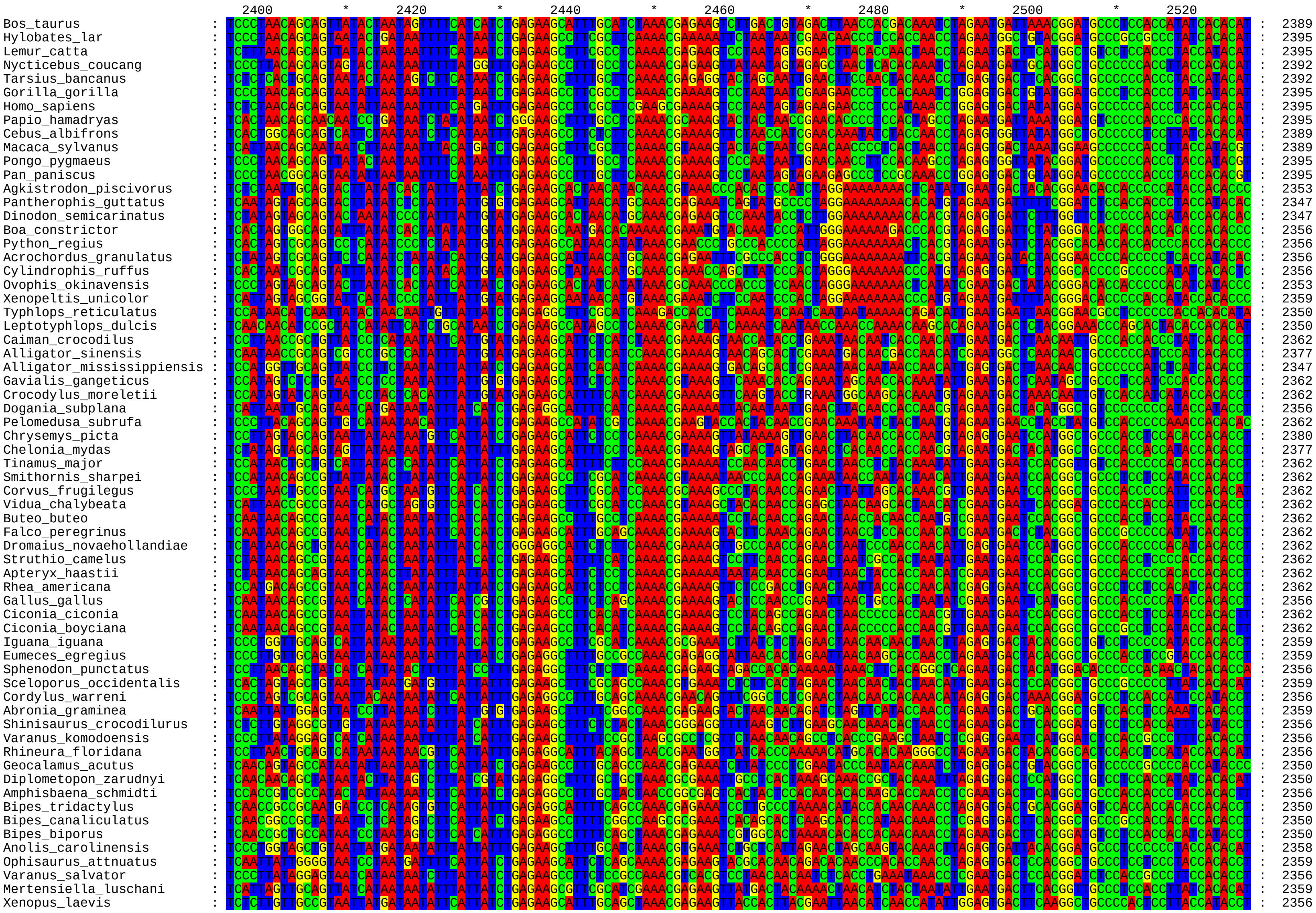
2400 * 2420 * 2440 * 2460 * 2480 * 2500 * 2520
Bos_taurus : 2389
Hylobates_lar : 2395
Lemur_catta : 2395
Nycticebus_coucang : 2392
Tarsius_bancanus : 2392
Gorilla_gorilla : 2395
Homo_sapiens : 2395
Papio_hamadryas : 2395
Cebus_albifrons : 2389
Macaca_sylvanus : 2389
Pongo_pygmaeus : 2395
Pan_paniscus : 2395
Agkistrodon_piscivorus : 2353
Pantherophis_guttatus : 2347
Dinodon_semicarinatus : 2347
Boa_constrictor : 2356
Python_regius : 2356
Acrochordus_granulatus : 2356
Cylindrophis_ruffus : 2356
Ovophis_okinavensis : 2353
Xenopeltis_unicolor : 2359
Typhlops_reticulatus : 2350
Leptotyphlops_dulcis : 2350
Caiman_crocodilus : 2362
Alligator_sinensis : 2377
Alligator_mississippiensis : 2347
Gavialis_gangeticus : 2362
Crocodylus_moreletii : 2362
Dogania_subplana : 2356
Pelomedusa_subrufa : 2362
Chrysemys_picta : 2380
Chelonia_mydas : 2377
Tinamus_major : 2362
Smithornis_sharpei : 2362
Corvus_frugilegus : 2362
Vidua_chalybeata : 2362
Buteo_buteo : 2362
Falco_peregrinus : 2362
Dromaius_novaehollandiae : 2362
Struthio_camelus : 2362
Apteryx_haastii : 2362
Rhea_americana : 2362
Gallus_gallus : 2356
Ciconia_ciconia : 2362
Ciconia_boyciana : 2362
Iguana_iguana : 2359
Eumeces_egregius : 2359
Sphenodon_punctatus : 2356
Sceloporus_occidentalis : 2359
Cordylus_warreni : 2356
Abronia_graminea : 2359
Shinisaurus_crocodilurus : 2356
Varanus_komodoensis : 2356
Rhineura_floridana : 2356
Geocalamus_acutus : 2350
Diplometopon_zarudnyi : 2350
Amphisbaena_schmidti : 2356
Bipes_tridactylus : 2350
Bipes_canaliculatus : 2350
Bipes_biporus : 2350
Anolis_carolinensis : 2358
Ophisaurus_attnuatus : 2359
Varanus_salvator : 2356
Mertensiella_luschani : 2359
Xenopus_laevis : 2356

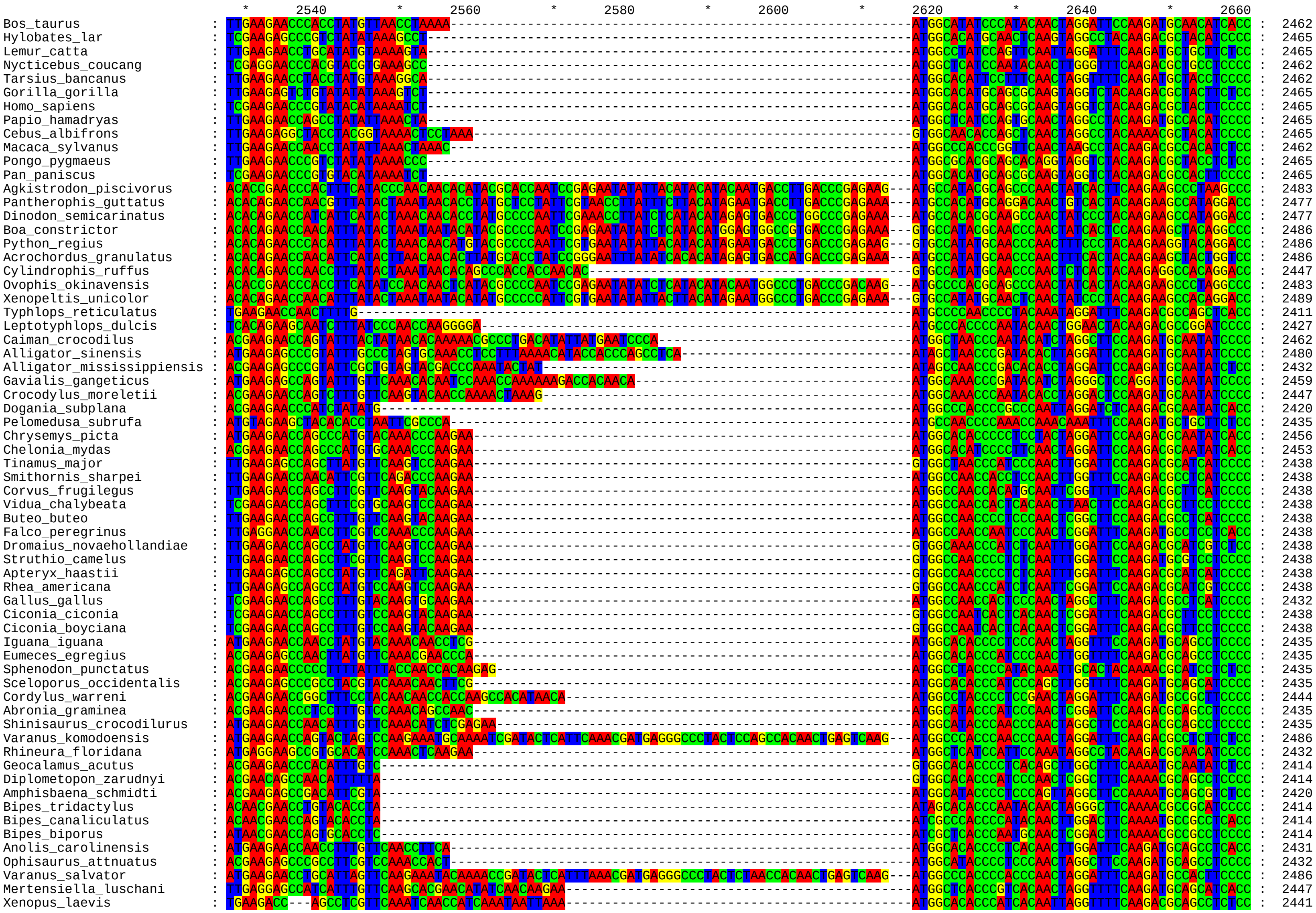

2540 2560 2580 2600 2620 2640 2660
Bos_taurus 2462
Hylobates_lar 2465
Lemur_catta 2465
Nycticebus_coucang 2462
Tarsius_bancanus 2462
Gorilla_gorilla 2465
Homo_sapiens 2465
Papio_hamadryas 2465
Cebus_albifrons 2465
Macaca_sylvanus 2462
Pongo_pygmaeus 2465
Pan_paniscus 2465
Agkistrodon_piscivorus 2483
Pantherophis_guttatus 2477
Dinodon_semicarinatus 2477
Boa_constrictor 2486
Python_regius 2486
Acrochordus_granulatus 2486
Cylindrophis_ruffus 2447
Ovophis_okinavensis 2483
Xenopeltis_unicolor 2489
Typhlops_reticulatus 2411
Leptotyphlops_dulcis 2427
Caiman_crocodilus 2462
Alligator_sinensis 2480
Alligator_mississippiensis 2432
Gavialis_gangeticus 2459
Crocodylus_moreletii 2447
Dogania_subplana 2420
Pelomedusa_subrufa 2435
Chrysemys_picta 2456
Chelonia_mydas 2453
Tinamus_major 2438
Smithornis_sharpei 2438
Corvus_frugilegus 2438
Vidua_chalybeata 2438
Buteo_buteo 2438
Falco_peregrinus 2438
Dromaius_novaehollandiae 2438
Struthio_camelus 2438
Apteryx_haastii 2438
Rhea_americana 2438
Gallus_gallus 2432
Ciconia_ciconia 2438
Ciconia_boyciana 2438
Iguana_iguana 2435
Eumeces_egregius 2435
Sphenodon_punctatus 2435
Sceloporus_occidentalis 2435
Cordylus_warreni 2444
Abronia_graminea 2435
Shinisaurus_crocodilurus 2435
Varanus_komodoensis 2486
Rhineura_floridana 2432
Geocalamus_acutus 2414
Diplometopon_zarudnyi 2414
Amphisbaena_schmidti 2420
Bipes_tridactylus 2414
Bipes_canaliculatus 2414
Bipes_biporus 2414
Anolis_carolinensis 2431
Ophisaurus_attnuatus 2432
Varanus_salvator 2486
Mertensiella_luschani 2447
Xenopus_laevis 2441

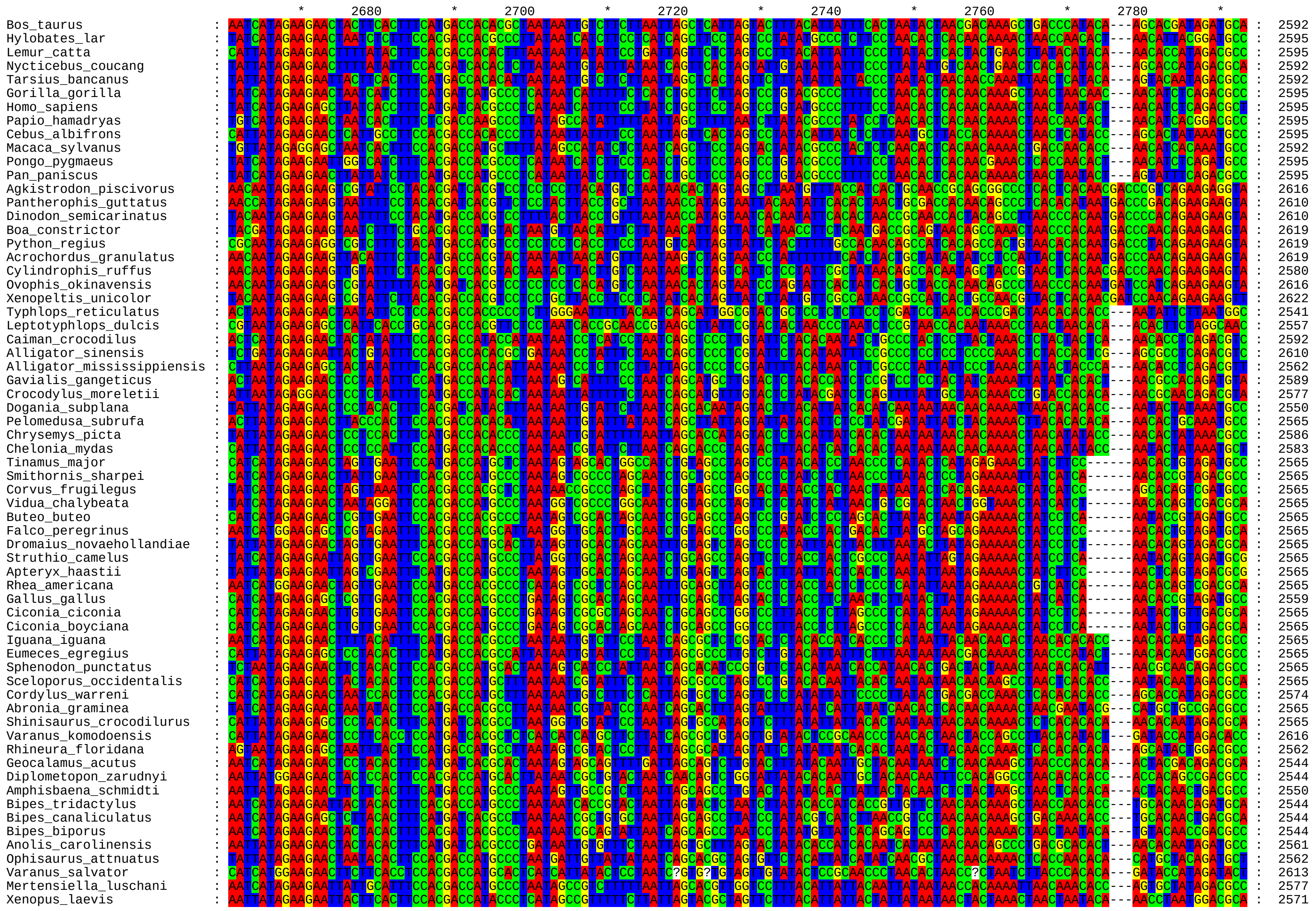
* 2680 * 2700 * 2720 * 2740 * 2760 * 2780 *
Bos_taurus : 2592
Hylobates_lar : 2595
Lemur_catta : 2595
Nycticebus_coucang : 2592
Tarsius_bancanus : 2592
Gorilla_gorilla : 2595
Homo_sapiens : 2595
Papio_hamadryas : 2595
Cebus_albifrons : 2595
Macaca_sylvanus : 2592
Pongo_pygmaeus : 2595
Pan_paniscus : 2595
Agkistrodon_piscivorus : 2616
Pantherophis_guttatus : 2610
Dinodon_semicarinatus : 2610
Boa_constrictor : 2619
Python_regius : 2619
Acrochordus_granulatus : 2619
Cylindrophis_ruffus : 2580
Ovophis_okinavensis : 2616
Xenopeltis_unicolor : 2622
Typhlops_reticulatus : 2541
Leptotyphlops_dulcis : 2557
Caiman_crocodilus : 2592
Alligator_sinensis : 2610
Alligator_mississippiensis : 2562
Gavialis_gangeticus : 2589
Crocodylus_moreletii : 2577
Dogania_subplana : 2550
Pelomedusa_subrufa : 2565
Chrysemys_picta : 2586
Chelonia_mydas : 2583
Tinamus_major : 2565
Smithornis_sharpei : 2565
Corvus_frugilegus : 2565
Vidua_chalybeata : 2565
Buteo_buteo : 2565
Falco_peregrinus : 2565
Dromaius_novaehollandiae : 2565
Struthio_camelus : 2565
Apteryx_haastii : 2565
Rhea_americana : 2565
Gallus_gallus : 2559
Ciconia_ciconia : 2565
Ciconia_boyciana : 2565
Iguana_iguana : 2565
Eumeces_egregius : 2565
Sphenodon_punctatus : 2565
Sceloporus_occidentalis : 2565
Cordylus_warreni : 2574
Abronia_graminea : 2565
Shinisaurus_crocodilurus : 2565
Varanus_komodoensis : 2616
Rhineura_floridana : 2562
Geocalamus_acutus : 2544
Diplometopon_zarudnyi : 2544
Amphisbaena_schmidti : 2550
Bipes_tridactylus : 2544
Bipes_canaliculatus : 2544
Bipes_biporus : 2544
Anolis_carolinensis : 2561
Ophisaurus_attnuatus : 2562
Varanus_salvator : 2613
Mertensiella_luschani : 2577
Xenopus_laevis : 2571

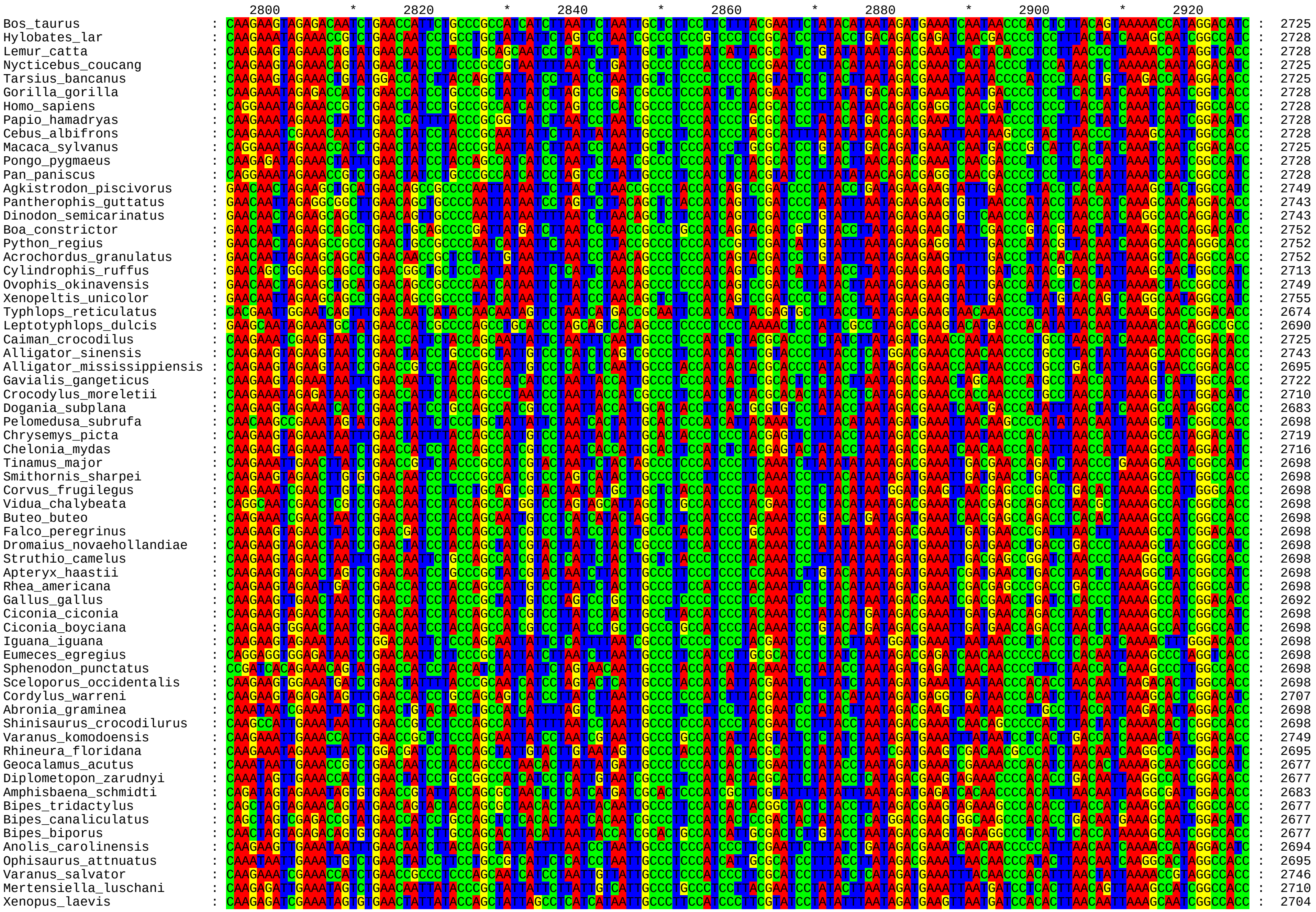
2800 * 2820 * 2840 * 2860 * 2880 * 2900 * 2920
Bos_taurus 2725
Hylobates_lar 2728
Lemur_catta 2728
Nycticebus_coucang 2725
Tarsius_bancanus 2725
Gorilla_gorilla 2728
Homo_sapiens 2728
Papio_hamadryas 2728
Cebus_albifrons 2728
Macaca_sylvanus 2725
Pongo_pygmaeus 2728
Pan_paniscus 2728
Agkistrodon_piscivorus 2749
Pantherophis_guttatus 2743
Dinodon_semicarinatus 2743
Boa_constrictor 2752
Python_regius 2752
Acrochordus_granulatus 2752
Cylindrophis_ruffus 2713
Ovophis_okinavensis 2749
Xenopeltis_unicolor 2755
Typhlops_reticulatus 2674
Leptotyphlops_dulcis 2690
Caiman_crocodilus 2725
Alligator_sinensis 2743
Alligator_mississippiensis 2695
Gavialis_gangeticus 2722
Crocodylus_moreletii 2710
Dogania_subplana 2683
Pelomedusa_subrufa 2698
Chrysemys_picta 2719
Chelonia_mydas 2716
Tinamus_major 2698
Smithornis_sharpei 2698
Corvus_frugilegus 2698
Vidua_chalybeata 2698
Buteo_buteo 2698
Falco_peregrinus 2698
Dromaius_novaehollandiae 2698
Struthio_camelus 2698
Apteryx_haastii 2698
Rhea_americana 2698
Gallus_gallus 2692
Ciconia_ciconia 2698
Ciconia_boyciana 2698
Iguana_iguana 2698
Eumeces_egregius 2698
Sphenodon_punctatus 2698
Sceloporus_occidentalis 2698
Cordylus_warreni 2707
Abronia_graminea 2698
Shinisaurus_crocodilurus 2698
Varanus_komodoensis 2749
Rhineura_floridana 2695
Geocalamus_acutus 2677
Diplometopon_zarudnyi 2677
Amphisbaena_schmidti 2683
Bipes_tridactylus 2677
Bipes_canaliculatus 2677
Bipes_biporus 2677
Anolis_carolinensis 2694
Ophisaurus_attnuatus 2695
Varanus_salvator 2746
Mertensiella_luschani 2710
Xenopus_laevis 2704

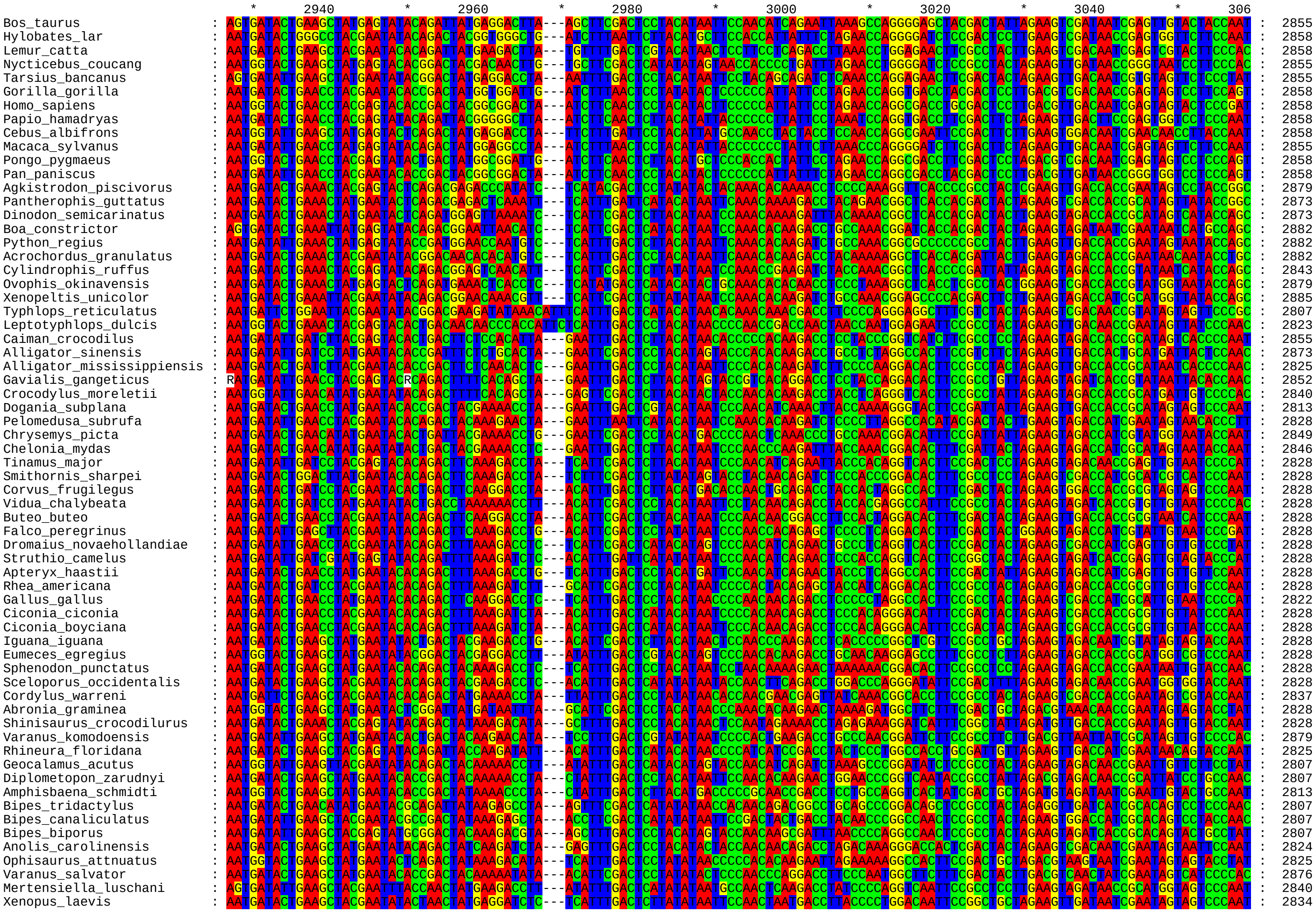

* 2940 * 2960 * 2980 * 3000 * 3020 * 3040 * 306
Bos_taurus : 2855
Hylobates_lar : 2858
Lemur_catta : 2858
Nycticebus_coucang : 2855
Tarsius_bancanus : 2855
Gorilla_gorilla : 2858
Homo_sapiens : 2858
Papio_hamadryas : 2858
Cebus_albifrons : 2858
Macaca_sylvanus : 2855
Pongo_pygmaeus : 2858
Pan_paniscus : 2858
Agkistrodon_piscivorus : 2879
Pantherophis_guttatus : 2873
Dinodon_semicarinatus : 2873
Boa_constrictor : 2882
Python_regius : 2882
Acrochordus_granulatus : 2882
Cylindrophis_ruffus : 2843
Ovophis_okinavensis : 2879
Xenopeltis_unicolor : 2885
Typhlops_reticulatus : 2807
Leptotyphlops_dulcis : 2823
Caiman_crocodilus : 2855
Alligator_sinensis : 2873
Alligator_mississippiensis : 2825
Gavialis_gangeticus : 2852
Crocodylus_moreletii : 2840
Dogania_subplana : 2813
Pelomedusa_subrufa : 2828
Chrysemys_picta : 2849
Chelonia_mydas : 2846
Tinamus_major : 2828
Smithornis_sharpei : 2828
Corvus_frugilegus : 2828
Vidua_chalybeata : 2828
Buteo_buteo : 2828
Falco_peregrinus : 2828
Dromaius_novaehollandiae : 2828
Struthio_camelus : 2828
Apteryx_haastii : 2828
Rhea_americana : 2828
Gallus_gallus : 2822
Ciconia_ciconia : 2828
Ciconia_boyciana : 2828
Iguana_iguana : 2828
Eumeces_egregius : 2828
Sphenodon_punctatus : 2828
Sceloporus_occidentalis : 2828
Cordylus_warreni : 2837
Abronia_graminea : 2828
Shinisaurus_crocodilurus : 2828
Varanus_komodoensis : 2879
Rhineura_floridana : 2825
Geocalamus_acutus : 2807
Diplometopon_zarudnyi : 2807
Amphisbaena_schmidti : 2813
Bipes_tridactylus : 2807
Bipes_canaliculatus : 2807
Bipes_biporus : 2807
Anolis_carolinensis : 2824
Ophisaurus_attnuatus : 2825
Varanus_salvator : 2876
Mertensiella_luschani : 2840
Xenopus_laevis : 2834

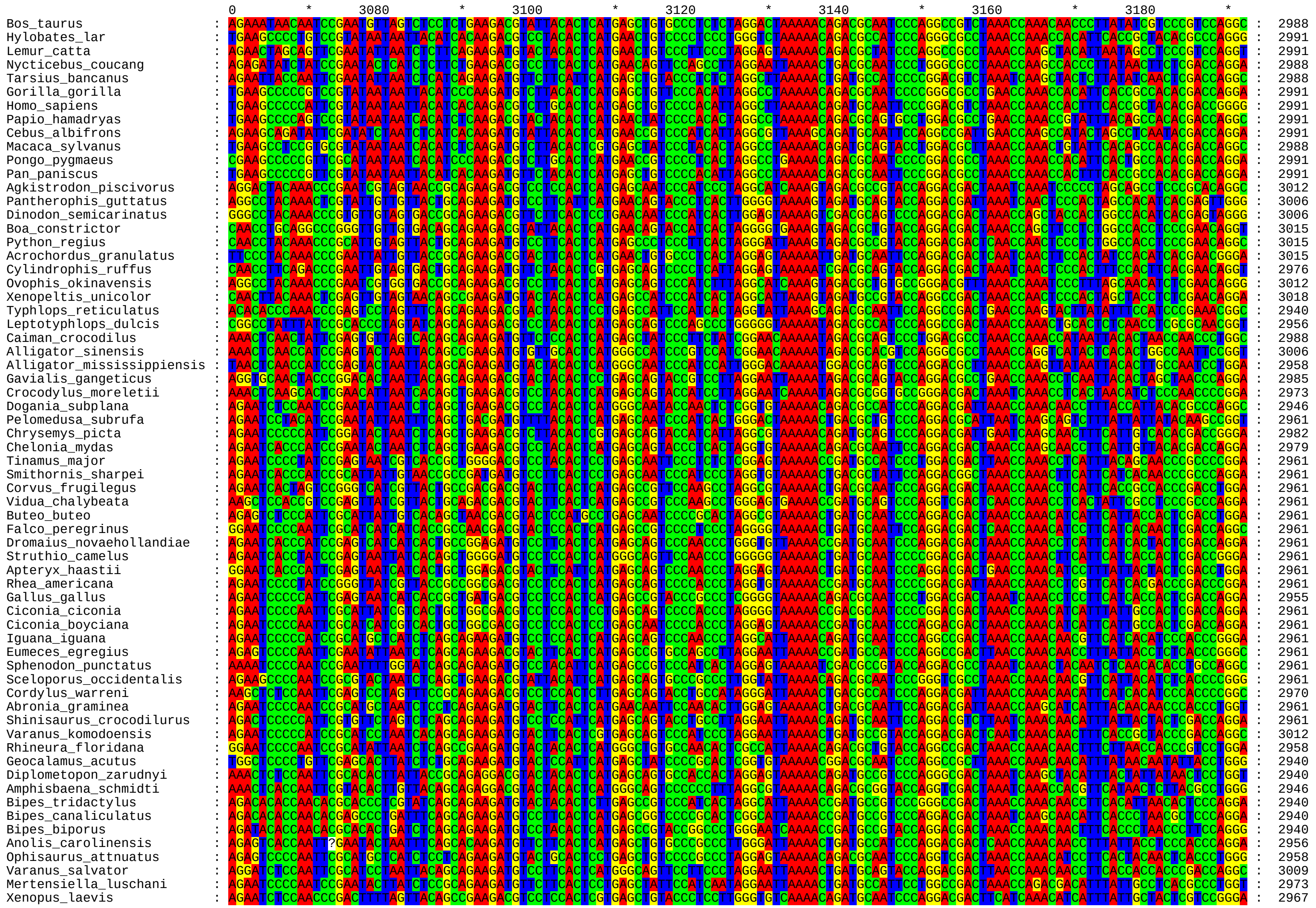
0 * 3080 * 3100 * 3120 * 3140 * 3160 * 3180 *
Bos_taurus : 2988
Hylobates_lar : 2991
Lemur_catta : 2991
Nycticebus_coucang : 2988
Tarsius_bancanus : 2988
Gorilla_gorilla : 2991
Homo_sapiens : 2991
Papio_hamadryas : 2991
Cebus_albifrons : 2991
Macaca_sylvanus : 2988
Pongo_pygmaeus : 2991
Pan_paniscus : 2991
Agkistrodon_piscivorus : 3012
Pantherophis_guttatus : 3006
Dinodon_semicarinatus : 3006
Boa_constrictor : 3015
Python_regius : 3015
Acrochordus_granulatus : 3015
Cylindrophis_ruffus : 2976
Ovophis_okinavensis : 3012
Xenopeltis_unicolor : 3018
Typhlops_reticulatus : 2940
Leptotyphlops_dulcis : 2956
Caiman_crocodilus : 2988
Alligator_sinensis : 3006
Alligator_mississippiensis : 2958
Gavialis_gangeticus : 2985
Crocodylus_moreletii : 2973
Dogania_subplana : 2946
Pelomedusa_subrufa : 2961
Chrysemys_picta : 2982
Chelonia_mydas : 2979
Tinamus_major : 2961
Smithornis_sharpei : 2961
Corvus_frugilegus : 2961
Vidua_chalybeata : 2961
Buteo_buteo : 2961
Falco_peregrinus : 2961
Dromaius_novaehollandiae : 2961
Struthio_camelus : 2961
Apteryx_haastii : 2961
Rhea_americana : 2961
Gallus_gallus : 2955
Ciconia_ciconia : 2961
Ciconia_boyciana : 2961
Iguana_iguana : 2961
Eumeces_egregius : 2961
Sphenodon_punctatus : 2961
Sceloporus_occidentalis : 2961
Cordylus_warreni : 2970
Abronia_graminea : 2961
Shinisaurus_crocodilurus : 2961
Varanus_komodoensis : 3012
Rhineura_floridana : 2958
Geocalamus_acutus : 2940
Diplometopon_zarudnyi : 2940
Amphisbaena_schmidti : 2946
Bipes_tridactylus : 2940
Bipes_canaliculatus : 2940
Bipes_biporus : 2940
Anolis_carolinensis : 2956
Ophisaurus_attnuatus : 2958
Varanus_salvator : 3009
Mertensiella_luschani : 2973
Xenopus_laevis : 2967

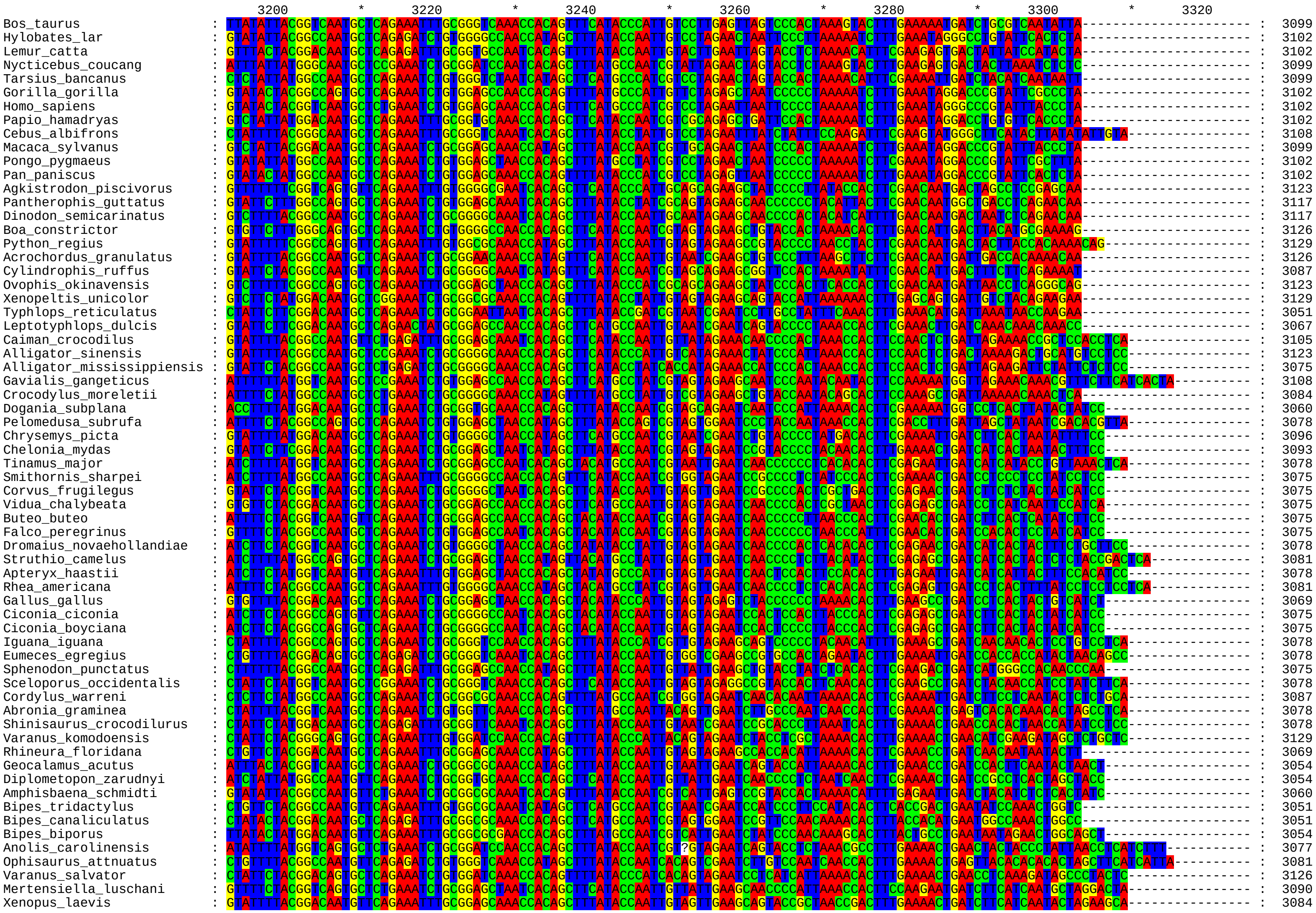
3200 * 3220 * 3240 * 3260 * 3280 * 3300 * 3320
Bos_taurus : 3099
Hylobates_lar : 3102
Lemur_catta : 3102
Nycticebus_coucang : 3099
Tarsius_bancanus : 3099
Gorilla_gorilla : 3102
Homo_sapiens : 3102
Papio_hamadryas : 3102
Cebus_albifrons : 3108
Macaca_sylvanus : 3099
Pongo_pygmaeus : 3102
Pan_paniscus : 3102
Agkistrodon_piscivorus : 3123
Pantherophis_guttatus : 3117
Dinodon_semicarinatus : 3117
Boa_constrictor : 3126
Python_regius : 3129
Acrochordus_granulatus : 3126
Cylindrophis_ruffus : 3087
Ovophis_okinavensis : 3123
Xenopeltis_unicolor : 3129
Typhlops_reticulatus : 3051
Leptotyphlops_dulcis : 3067
Caiman_crocodilus : 3105
Alligator_sinensis : 3123
Alligator_mississippiensis : 3075
Gavialis_gangeticus : 3108
Crocodylus_moreletii : 3084
Dogania_subplana : 3060
Pelomedusa_subrufa : 3078
Chrysemys_picta : 3096
Chelonia_mydas : 3093
Tinamus_major : 3078
Smithornis_sharpei : 3075
Corvus_frugilegus : 3075
Vidua_chalybeata : 3075
Buteo_buteo : 3075
Falco_peregrinus : 3075
Dromaius_novaehollandiae : 3078
Struthio_camelus : 3081
Apteryx_haastii : 3078
Rhea_americana : 3081
Gallus_gallus : 3069
Ciconia_ciconia : 3075
Ciconia_boyciana : 3075
Iguana_iguana : 3078
Eumeces_egregius : 3078
Sphenodon_punctatus : 3075
Sceloporus_occidentalis : 3078
Cordylus_warreni : 3087
Abronia_graminea : 3078
Shinisaurus_crocodilurus : 3078
Varanus_komodoensis : 3129
Rhineura_floridana : 3069
Geocalamus_acutus : 3054
Diplometopon_zarudnyi : 3054
Amphisbaena_schmidti : 3060
Bipes_tridactylus : 3051
Bipes_canaliculatus : 3051
Bipes_biporus : 3054
Anolis_carolinensis : 3077
Ophisaurus_attnuatus : 3081
Varanus_salvator : 3126
Mertensiella_luschani : 3090
Xenopus_laevis : 3084

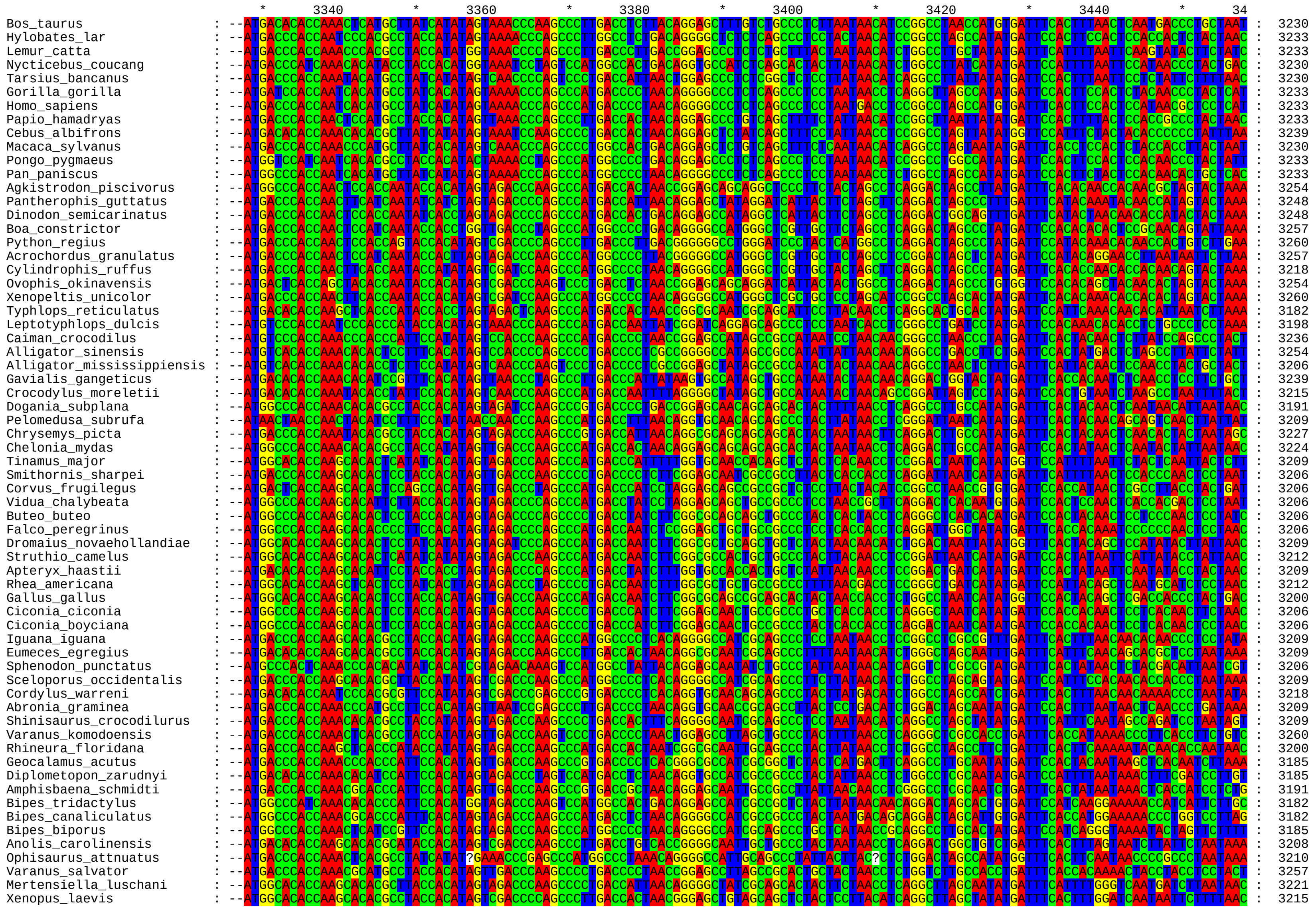
* 3340 * 3360 * 3380 * 3400 * 3420 * 3440 * 34
Bos_taurus : 3230
Hylobates_lar : 3233
Lemur_catta : 3233
Nycticebus_coucang : 3230
Tarsius_bancanus : 3230
Gorilla_gorilla : 3233
Homo_sapiens : 3233
Papio_hamadryas : 3233
Cebus_albifrons : 3239
Macaca_sylvanus : 3230
Pongo_pygmaeus : 3233
Pan_paniscus : 3233
Agkistrodon_piscivorus : 3254
Pantherophis_guttatus : 3248
Dinodon_semicarinatus : 3248
Boa_constrictor : 3257
Python_regius : 3260
Acrochordus_granulatus : 3257
Cylindrophis_ruffus : 3218
Ovophis_okinavensis : 3254
Xenopeltis_unicolor : 3260
Typhlops_reticulatus : 3182
Leptotyphlops_dulcis : 3198
Caiman_crocodilus : 3236
Alligator_sinensis : 3254
Alligator_mississippiensis : 3206
Gavialis_gangeticus : 3239
Crocodylus_moreletii : 3215
Dogania_subplana : 3191
Pelomedusa_subrufa : 3209
Chrysemys_picta : 3227
Chelonia_mydas : 3224
Tinamus_major : 3209
Smithornis_sharpei : 3206
Corvus_frugilegus : 3206
Vidua_chalybeata : 3206
Buteo_buteo : 3206
Falco_peregrinus : 3206
Dromaius_novaehollandiae : 3209
Struthio_camelus : 3212
Apteryx_haastii : 3209
Rhea_americana : 3212
Gallus_gallus : 3200
Ciconia_ciconia : 3206
Ciconia_boyciana : 3206
Iguana_iguana : 3209
Eumeces_egregius : 3209
Sphenodon_punctatus : 3206
Sceloporus_occidentalis : 3209
Cordylus_warreni : 3218
Abronia_graminea : 3209
Shinisaurus_crocodilurus : 3209
Varanus_komodoensis : 3260
Rhineura_floridana : 3200
Geocalamus_acutus : 3185
Diplometopon_zarudnyi : 3185
Amphisbaena_schmidti : 3191
Bipes_tridactylus : 3182
Bipes_canaliculatus : 3182
Bipes_biporus : 3185
Anolis_carolinensis : 3208
Ophisaurus_attnuatus : 3210
Varanus_salvator : 3257
Mertensiella_luschani : 3221
Xenopus_laevis : 3215

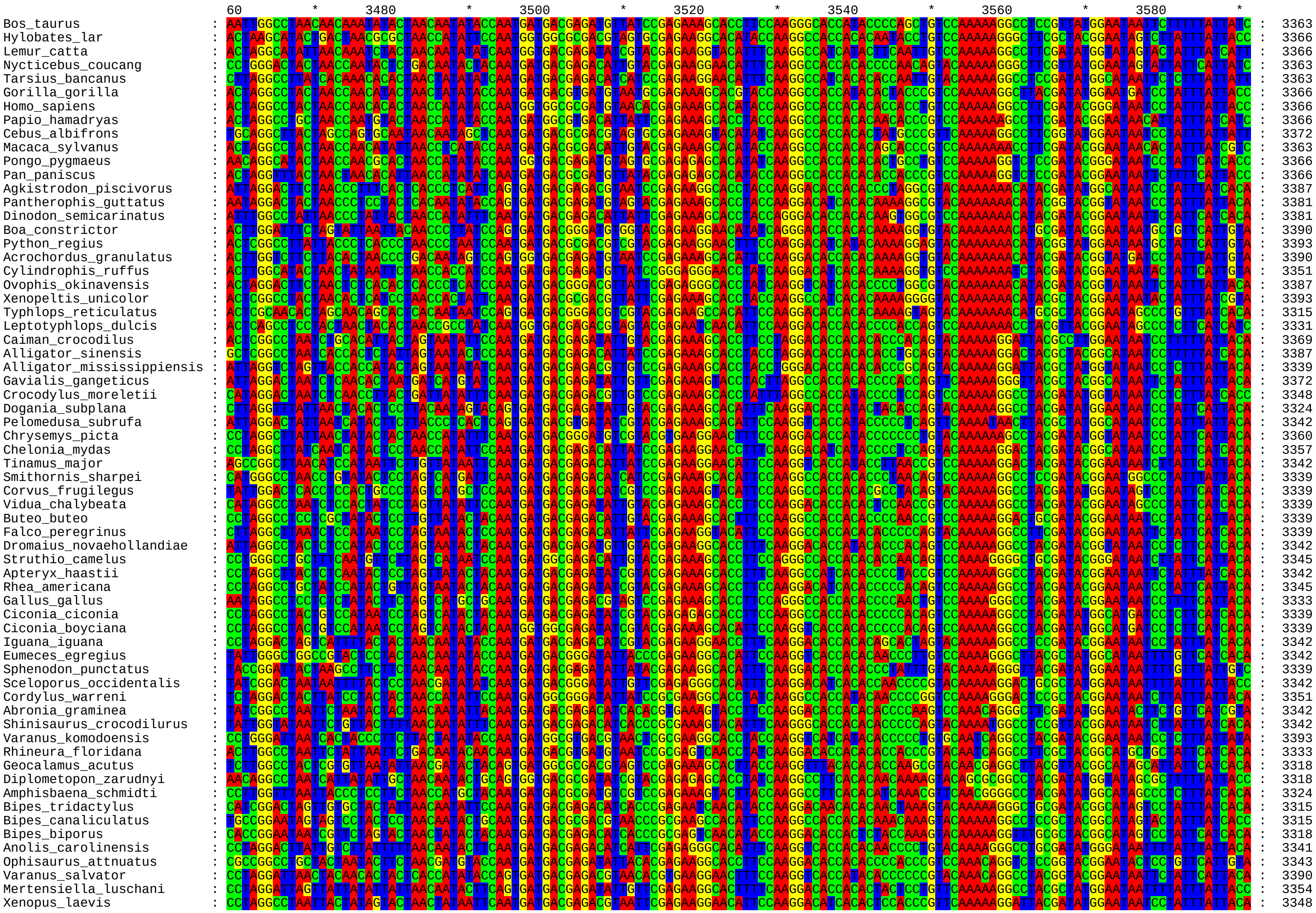
60 * 3480 * 3500 * 3520 * 3540 * 3560 * 3580 *
Bos_taurus : 3363
Hylobates_lar : 3366
Lemur_catta : 3366
Nycticebus_coucang : 3363
Tarsius_bancanus : 3363
Gorilla_gorilla : 3366
Homo_sapiens : 3366
Papio_hamadryas : 3366
Cebus_albifrons : 3372
Macaca_sylvanus : 3363
Pongo_pygmaeus : 3366
Pan_paniscus : 3366
Agkistrodon_piscivorus : 3387
Pantherophis_guttatus : 3381
Dinodon_semicarinatus : 3381
Boa_constrictor : 3390
Python_regius : 3393
Acrochordus_granulatus : 3390
Cylindrophis_ruffus : 3351
Ovophis_okinavensis : 3387
Xenopeltis_unicolor : 3393
Typhlops_reticulatus : 3315
Leptotyphlops_dulcis : 3331
Caiman_crocodilus : 3369
Alligator_sinensis : 3387
Alligator_mississippiensis : 3339
Gavialis_gangeticus : 3372
Crocodylus_moreletii : 3348
Dogania_subplana : 3324
Pelomedusa_subrufa : 3342
Chrysemys_picta : 3360
Chelonia_mydas : 3357
Tinamus_major : 3342
Smithornis_sharpei : 3339
Corvus_frugilegus : 3339
Vidua_chalybeata : 3339
Buteo_buteo : 3339
Falco_peregrinus : 3339
Dromaius_novaehollandiae : 3342
Struthio_camelus : 3345
Apteryx_haastii : 3342
Rhea_americana : 3345
Gallus_gallus : 3333
Ciconia_ciconia : 3339
Ciconia_boyciana : 3339
Iguana_iguana : 3342
Eumeces_egregius : 3342
Sphenodon_punctatus : 3339
Sceloporus_occidentalis : 3342
Cordylus_warreni : 3351
Abronia_graminea : 3342
Shinisaurus_crocodilurus : 3342
Varanus_komodoensis : 3393
Rhineura_floridana : 3333
Geocalamus_acutus : 3318
Diplometopon_zarudnyi : 3318
Amphisbaena_schmidti : 3324
Bipes_tridactylus : 3315
Bipes_canaliculatus : 3315
Bipes_biporus : 3318
Anolis_carolinensis : 3341
Ophisaurus_attnuatus : 3343
Varanus_salvator : 3390
Mertensiella_luschani : 3354
Xenopus_laevis : 3348

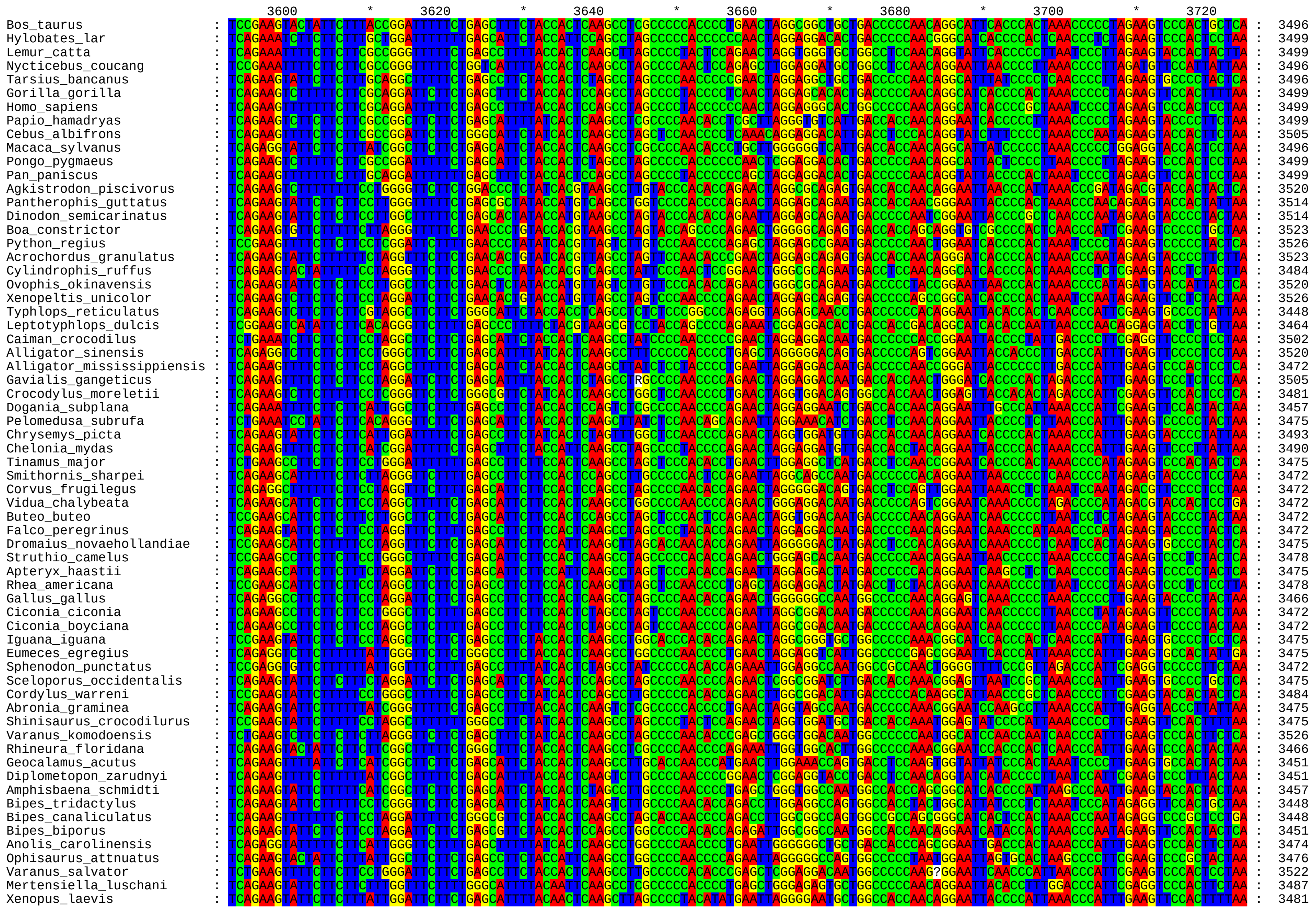
3600 * 3620 * 3640 * 3660 * 3680 * 3700 * 3720
Bos_taurus : 3496
Hylobates_lar : 3499
Lemur_catta : 3499
Nycticebus_coucang : 3496
Tarsius_bancanus : 3496
Gorilla_gorilla : 3499
Homo_sapiens : 3499
Papio_hamadryas : 3499
Cebus_albifrons : 3505
Macaca_sylvanus : 3496
Pongo_pygmaeus : 3499
Pan_paniscus : 3499
Agkistrodon_piscivorus : 3520
Pantherophis_guttatus : 3514
Dinodon_semicarinatus : 3514
Boa_constrictor : 3523
Python_regius : 3526
Acrochordus_granulatus : 3523
Cylindrophis_ruffus : 3484
Ovophis_okinavensis : 3520
Xenopeltis_unicolor : 3526
Typhlops_reticulatus : 3448
Leptotyphlops_dulcis : 3464
Caiman_crocodilus : 3502
Alligator_sinensis : 3520
Alligator_mississippiensis : 3472
Gavialis_gangeticus : 3505
Crocodylus_moreletii : 3481
Dogania_subplana : 3457
Pelomedusa_subrufa : 3475
Chrysemys_picta : 3493
Chelonia_mydas : 3490
Tinamus_major : 3475
Smithornis_sharpei : 3472
Corvus_frugilegus : 3472
Vidua_chalybeata : 3472
Buteo_buteo : 3472
Falco_peregrinus : 3472
Dromaius_novaehollandiae : 3475
Struthio_camelus : 3478
Apteryx_haastii : 3475
Rhea_americana : 3478
Gallus_gallus : 3466
Ciconia_ciconia : 3472
Ciconia_boyciana : 3472
Iguana_iguana : 3475
Eumeces_egregius : 3475
Sphenodon_punctatus : 3472
Sceloporus_occidentalis : 3475
Cordylus_warreni : 3484
Abronia_graminea : 3475
Shinisaurus_crocodilurus : 3475
Varanus_komodoensis : 3526
Rhineura_floridana : 3466
Geocalamus_acutus : 3451
Diplometopon_zarudnyi : 3451
Amphisbaena_schmidti : 3457
Bipes_tridactylus : 3448
Bipes_canaliculatus : 3448
Bipes_biporus : 3451
Anolis_carolinensis : 3474
Ophisaurus_attnuatus : 3476
Varanus_salvator : 3522
Mertensiella_luschani : 3487
Xenopus_laevis : 3481

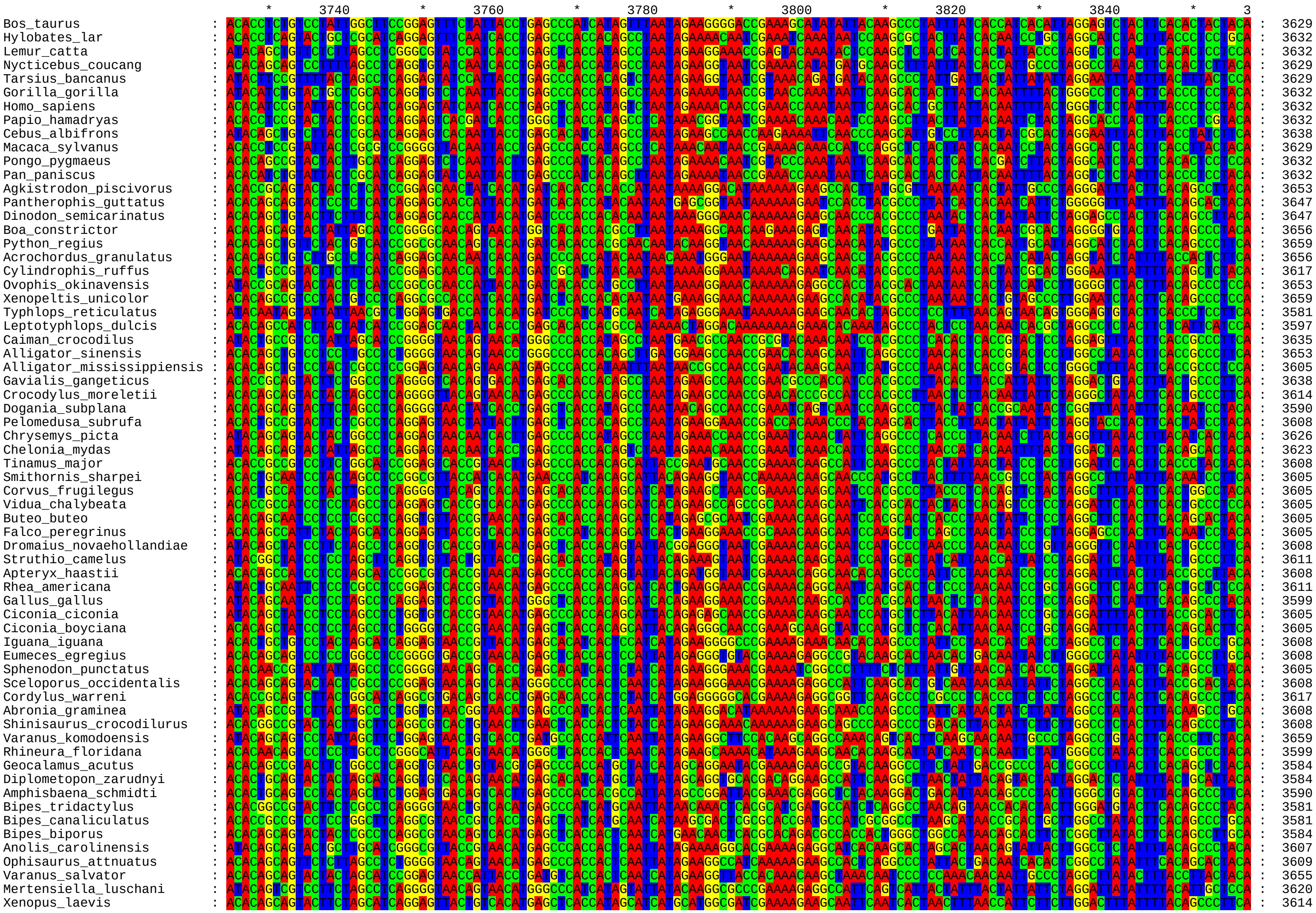
3740
3760
3780
3800
3820
3840
Bos_taurus : 3629
Hylobates_lar : 3632
Lemur_catta : 3632
Nycticebus_coucang : 3629
Tarsius_bancanus : 3629
Gorilla_gorilla : 3632
Homo_sapiens : 3632
Papio_hamadryas : 3632
Cebus_albifrons : 3638
Macaca_sylvanus : 3629
Pongo_pygmaeus : 3632
Pan_paniscus : 3632
Agkistrodon_piscivorus : 3653
Pantherophis_guttatus : 3647
Dinodon_semicarinatus : 3647
Boa_constrictor : 3656
Python_regius : 3659
Acrochordus_granulatus : 3656
Cylindrophis_ruffus : 3617
Ovophis_okinavensis : 3653
Xenopeltis_unicolor : 3659
Typhlops_reticulatus : 3581
Leptotyphlops_dulcis : 3597
Caiman_crocodilus : 3635
Alligator_sinensis : 3653
Alligator_mississippiensis : 3605
Gavialis_gangeticus : 3638
Crocodylus_moreletii : 3614
Dogania_subplana : 3590
Pelomedusa_subrufa : 3608
Chrysemys_picta : 3626
Chelonia_mydas : 3623
Tinamus_major : 3608
Smithornis_sharpei : 3605
Corvus_frugilegus : 3605
Vidua_chalybeata : 3605
Buteo_buteo : 3605
Falco_peregrinus : 3605
Dromaius_novaehollandiae : 3608
Struthio_camelus : 3611
Apteryx_haastii : 3608
Rhea_americana : 3611
Gallus_gallus : 3599
Ciconia_ciconia : 3605
Ciconia_boyciana : 3605
Iguana_iguana : 3608
Eumeces_egregius : 3608
Sphenodon_punctatus : 3605
Sceloporus_occidentalis : 3608
Cordylus_warreni : 3617
Abronia_graminea : 3608
Shinisaurus_crocodilurus : 3608
Varanus_komodoensis : 3659
Rhineura_floridana : 3599
Geocalamus_acutus : 3584
Diplometopon_zarudnyi : 3584
Amphisbaena_schmidti : 3590
Bipes_tridactylus : 3581
Bipes_canaliculatus : 3581
Bipes_biporus : 3584
Anolis_carolinensis : 3607
Ophisaurus_attnuatus : 3609
Varanus_salvator : 3655
Mertensiella_luschani : 3620
Xenopus_laevis : 3614

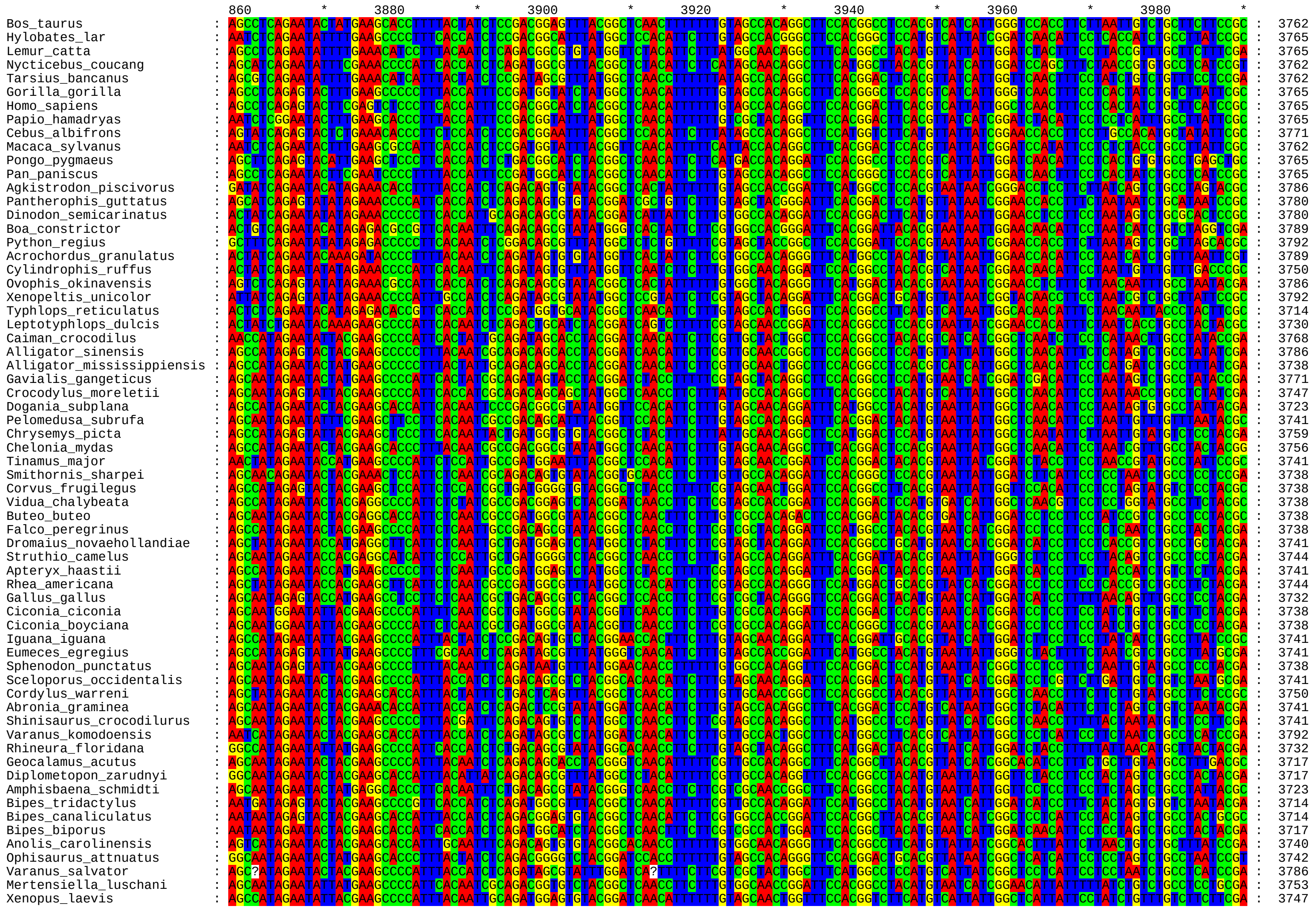

860 * 3880 * 3900 * 3920 * 3940 * 3960 * 3980 *
Bos_taurus : 3762
Hylobates_lar : 3765
Lemur_catta : 3765
Nycticebus_coucang : 3762
Tarsius_bancanus : 3762
Gorilla_gorilla : 3765
Homo_sapiens : 3765
Papio_hamadryas : 3765
Cebus_albifrons : 3771
Macaca_sylvanus : 3762
Pongo_pygmaeus : 3765
Pan_paniscus : 3765
Agkistrodon_piscivorus : 3786
Pantherophis_guttatus : 3780
Dinodon_semicarinatus : 3780
Boa_constrictor : 3789
Python_regius : 3792
Acrochordus_granulatus : 3789
Cylindrophis_ruffus : 3750
Ovophis_okinavensis : 3786
Xenopeltis_unicolor : 3792
Typhlops_reticulatus : 3714
Leptotyphlops_dulcis : 3730
Caiman_crocodilus : 3768
Alligator_sinensis : 3786
Alligator_mississippiensis : 3738
Gavialis_gangeticus : 3771
Crocodylus_moreletii : 3747
Dogania_subplana : 3723
Pelomedusa_subrufa : 3741
Chrysemys_picta : 3759
Chelonia_mydas : 3756
Tinamus_major : 3741
Smithornis_sharpei : 3738
Corvus_frugilegus : 3738
Vidua_chalybeata : 3738
Buteo_buteo : 3738
Falco_peregrinus : 3738
Dromaius_novaehollandiae : 3741
Struthio_camelus : 3744
Apteryx_haastii : 3741
Rhea_americana : 3744
Gallus_gallus : 3732
Ciconia_ciconia : 3738
Ciconia_boyciana : 3738
Iguana_iguana : 3741
Eumeces_egregius : 3741
Sphenodon_punctatus : 3738
Sceloporus_occidentalis : 3741
Cordylus_warreni : 3750
Abronia_graminea : 3741
Shinisaurus_crocodilurus : 3741
Varanus_komodoensis : 3792
Rhineura_floridana : 3732
Geocalamus_acutus : 3717
Diplometopon_zarudnyi : 3717
Amphisbaena_schmidti : 3723
Bipes_tridactylus : 3714
Bipes_canaliculatus : 3714
Bipes_biporus : 3717
Anolis_carolinensis : 3740
Ophisaurus_attnuatus : 3742
Varanus_salvator : 3786
Mertensiella_luschani : 3753
Xenopus_laevis : 3747

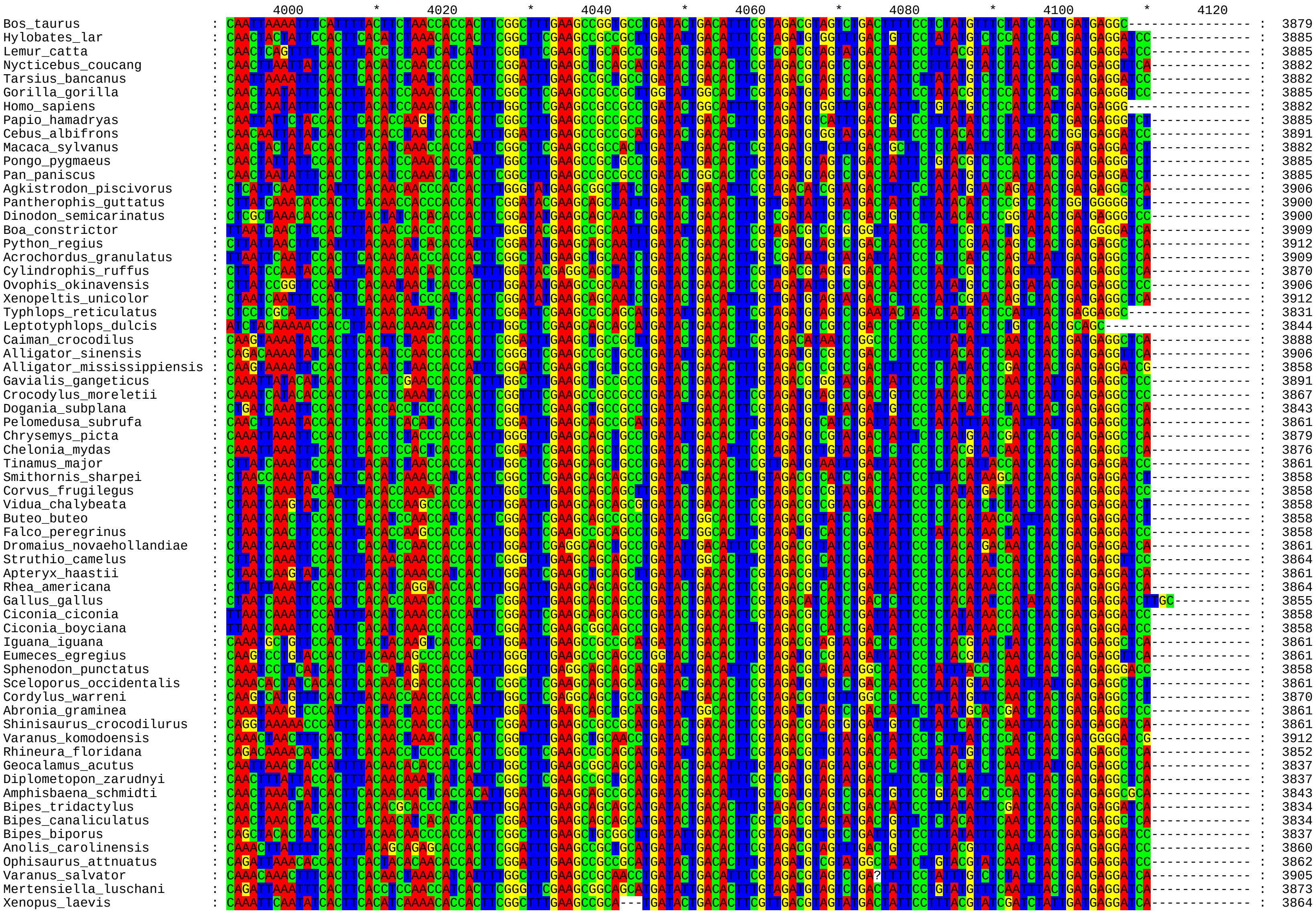
4000 * 4020 * 4040 * 4060 * 4080 * 4100 * 4120
Bos_taurus : 3879
Hylobates_lar : 3885
Lemur_catta : 3885
Nycticebus_coucang : 3882
Tarsius_bancanus : 3882
Gorilla_gorilla : 3885
Homo_sapiens : 3882
Papio_hamadryas : 3885
Cebus_albifrons : 3891
Macaca_sylvanus : 3882
Pongo_pygmaeus : 3885
Pan_paniscus : 3885
Agkistrodon_piscivorus : 3906
Pantherophis_guttatus : 3900
Dinodon_semicarinatus : 3900
Boa_constrictor : 3909
Python_regius : 3912
Acrochordus_granulatus : 3909
Cylindrophis_ruffus : 3870
Ovophis_okinavensis : 3906
Xenopeltis_unicolor : 3912
Typhlops_reticulatus : 3831
Leptotyphlops_dulcis : 3844
Caiman_crocodilus : 3888
Alligator_sinensis : 3906
Alligator_mississippiensis : 3858
Gavialis_gangeticus : 3891
Crocodylus_moreletii : 3867
Dogania_subplana : 3843
Pelomedusa_subrufa : 3861
Chrysemys_picta : 3879
Chelonia_mydas : 3876
Tinamus_major : 3861
Smithornis_sharpei : 3858
Corvus_frugilegus : 3858
Vidua_chalybeata : 3858
Buteo_buteo : 3858
Falco_peregrinus : 3858
Dromaius_novaehollandiae : 3861
Struthio_camelus : 3864
Apteryx_haastii : 3861
Rhea_americana : 3864
Gallus_gallus : 3855
Ciconia_ciconia : 3858
Ciconia_boyciana : 3858
Iguana_iguana : 3861
Eumeces_egregius : 3861
Sphenodon_punctatus : 3858
Sceloporus_occidentalis : 3861
Cordylus_warreni : 3870
Abronia_graminea : 3861
Shinisaurus_crocodilurus : 3861
Varanus_komodoensis : 3912
Rhineura_floridana : 3852
Geocalamus_acutus : 3837
Diplometopon_zarudnyi : 3837
Amphisbaena_schmidti : 3843
Bipes_tridactylus : 3834
Bipes_canaliculatus : 3834
Bipes_biporus : 3837
Anolis_carolinensis : 3860
Ophisaurus_attnuatus : 3862
Varanus_salvator : 3905
Mertensiella_luschani : 3873
Xenopus_laevis : 3864

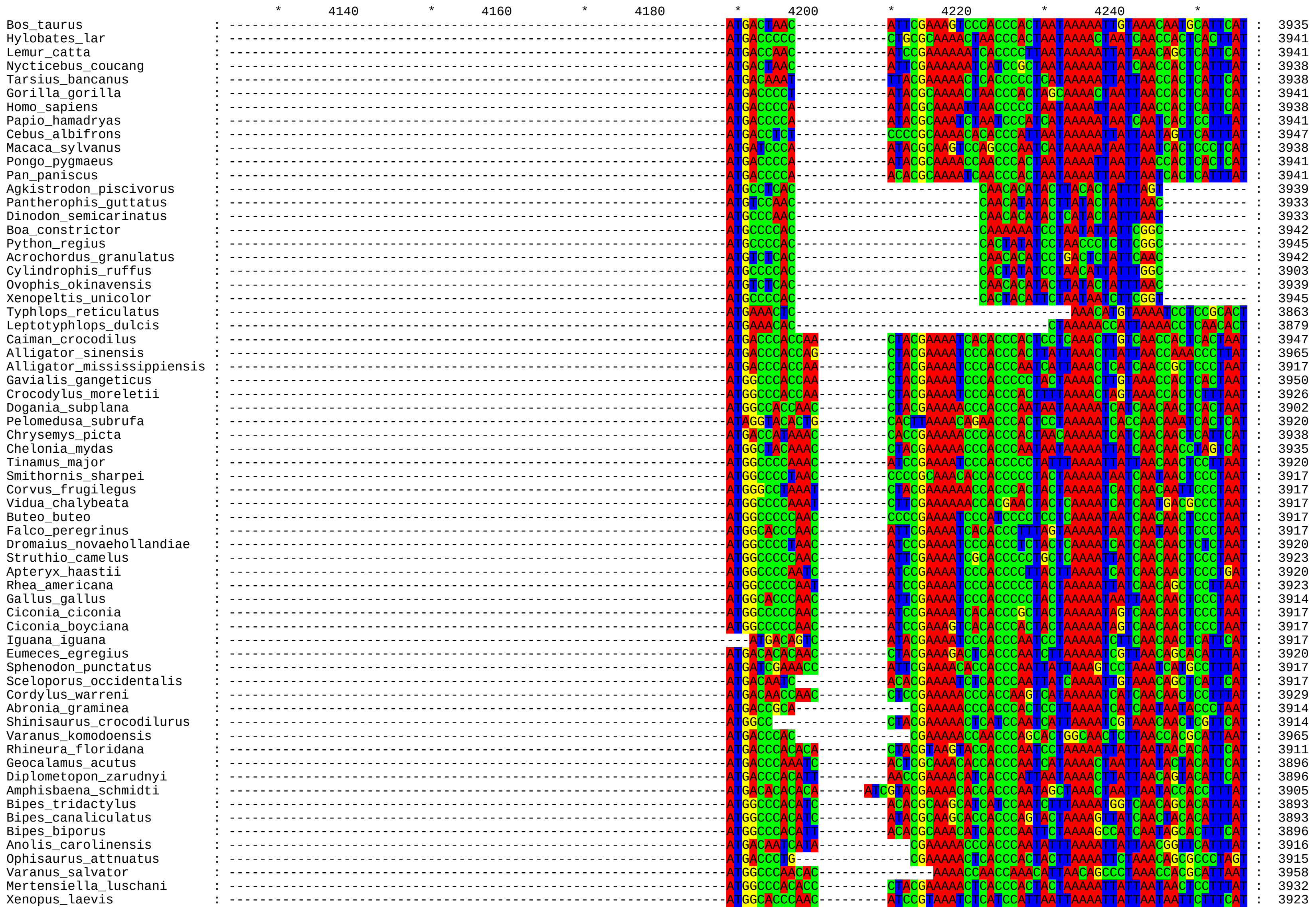
4140
4160
4180
4200
4220
4240
Bos_taurus : 3935
Hylobates_lar : 3941
Lemur_catta : 3941
Nycticebus_coucang : 3938
Tarsius_bancanus : 3938
Gorilla_gorilla : 3941
Homo_sapiens : 3938
Papio_hamadryas : 3941
Cebus_albifrons : 3947
Macaca_sylvanus : 3938
Pongo_pygmaeus : 3941
Pan_paniscus : 3941
Agkistrodon_piscivorus : 3939
Pantherophis_guttatus : 3933
Dinodon_semicarinatus : 3933
Boa_constrictor : 3942
Python_regius : 3945
Acrochordus_granulatus : 3942
Cylindrophis_ruffus : 3903
Ovophis_okinavensis : 3939
Xenopeltis_unicolor : 3945
Typhlops_reticulatus : 3863
Leptotyphlops_dulcis : 3879
Caiman_crocodilus : 3947
Alligator_sinensis : 3965
Alligator_mississippiensis : 3917
Gavialis_gangeticus : 3950
Crocodylus_moreletii : 3926
Dogania_subplana : 3902
Pelomedusa_subrufa : 3920
Chrysemys_picta : 3938
Chelonia_mydas : 3935
Tinamus_major : 3920
Smithornis_sharpei : 3917
Corvus_frugilegus : 3917
Vidua_chalybeata : 3917
Buteo_buteo : 3917
Falco_peregrinus : 3917
Dromaius_novaehollandiae : 3920
Struthio_camelus : 3923
Apteryx_haastii : 3920
Rhea_americana : 3923
Gallus_gallus : 3914
Ciconia_ciconia : 3917
Ciconia_boyciana : 3917
Iguana_iguana : 3917
Eumeces_egregius : 3920
Sphenodon_punctatus : 3917
Sceloporus_occidentalis : 3917
Cordylus_warreni : 3929
Abronia_graminea : 3914
Shinisaurus_crocodilurus : 3914
Varanus_komodoensis : 3965
Rhineura_floridana : 3911
Geocalamus_acutus : 3896
Diplometopon_zarudnyi : 3896
Amphisbaena_schmidti : 3905
Bipes_tridactylus : 3893
Bipes_canaliculatus : 3893
Bipes_biporus : 3896
Anolis_carolinensis : 3916
Ophisaurus_attnuatus : 3915
Varanus_salvator : 3958
Mertensiella_luschani : 3932
Xenopus_laevis : 3923

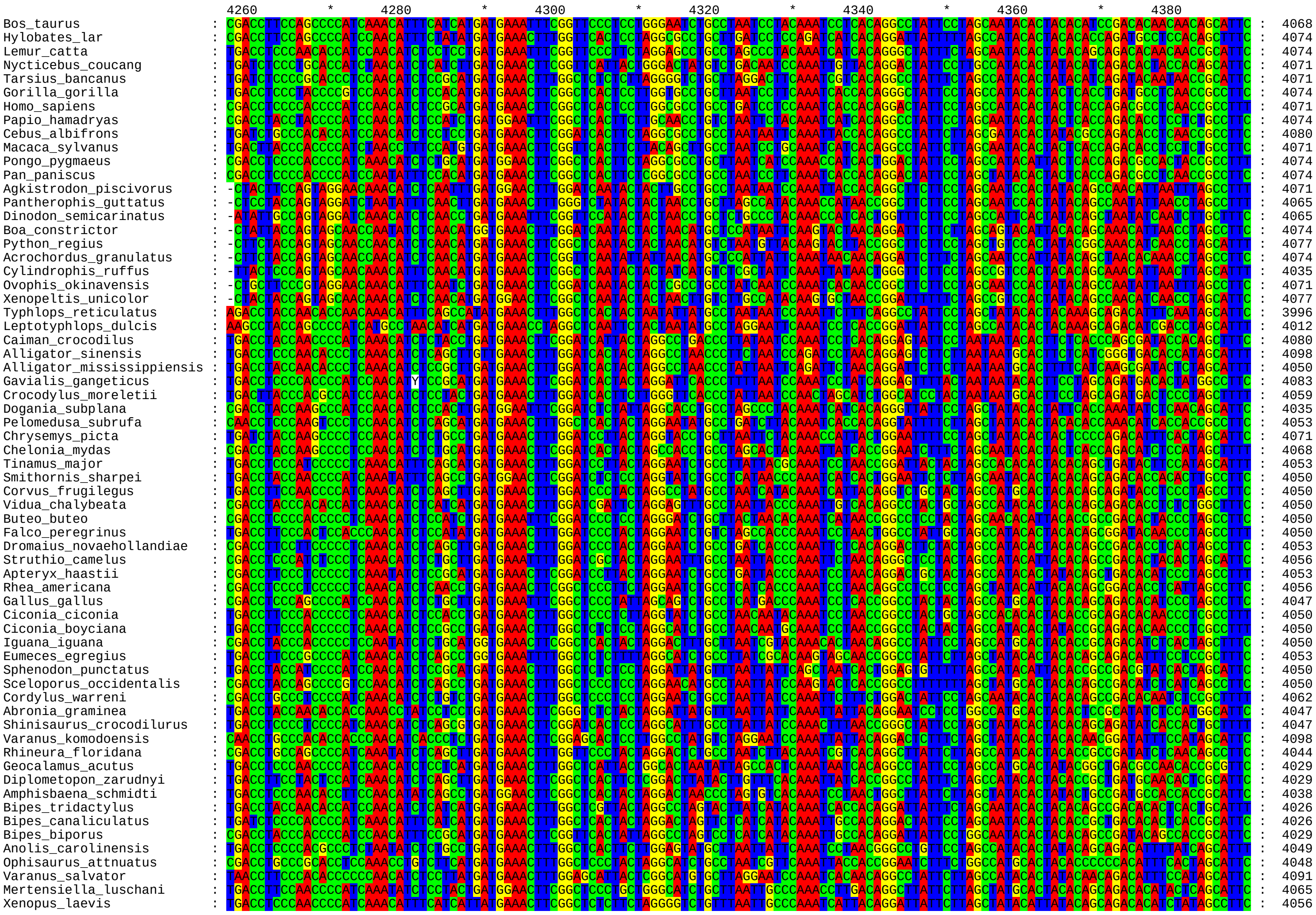
4260 * 4280 * 4300 * 4320 * 4340 * 4360 * 4380
Bos_taurus : 4068
Hylobates_lar : 4074
Lemur_catta : 4074
Nycticebus_coucang : 4071
Tarsius_bancanus : 4071
Gorilla_gorilla : 4074
Homo_sapiens : 4071
Papio_hamadryas : 4074
Cebus_albifrons : 4080
Macaca_sylvanus : 4071
Pongo_pygmaeus : 4074
Pan_paniscus : 4074
Agkistrodon_piscivorus : 4071
Pantherophis_guttatus : 4065
Dinodon_semicarinatus : 4065
Boa_constrictor : 4074
Python_regius : 4077
Acrochordus_granulatus : 4074
Cylindrophis_ruffus : 4035
Ovophis_okinavensis : 4071
Xenopeltis_unicolor : 4077
Typhlops_reticulatus : 3996
Leptotyphlops_dulcis : 4012
Caiman_crocodilus : 4080
Alligator_sinensis : 4098
Alligator_mississippiensis : 4050
Gavialis_gangeticus : 4083
Crocodylus_moreletii : 4059
Dogania_subplana : 4035
Pelomedusa_subrufa : 4053
Chrysemys_picta : 4071
Chelonia_mydas : 4068
Tinamus_major : 4053
Smithornis_sharpei : 4050
Corvus_frugilegus : 4050
Vidua_chalybeata : 4050
Buteo_buteo : 4050
Falco_peregrinus : 4050
Dromaius_novaehollandiae : 4053
Struthio_camelus : 4056
Apteryx_haastii : 4053
Rhea_americana : 4056
Gallus_gallus : 4047
Ciconia_ciconia : 4050
Ciconia_boyciana : 4050
Iguana_iguana : 4050
Eumeces_egregius : 4053
Sphenodon_punctatus : 4050
Sceloporus_occidentalis : 4050
Cordylus_warreni : 4062
Abronia_graminea : 4047
Shinisaurus_crocodilurus : 4047
Varanus_komodoensis : 4098
Rhineura_floridana : 4044
Geocalamus_acutus : 4029
Diplometopon_zarudnyi : 4029
Amphisbaena_schmidti : 4038
Bipes_tridactylus : 4026
Bipes_canaliculatus : 4026
Bipes_biporus : 4029
Anolis_carolinensis : 4049
Ophisaurus_attnuatus : 4048
Varanus_salvator : 4091
Mertensiella_luschani : 4065
Xenopus_laevis : 4056

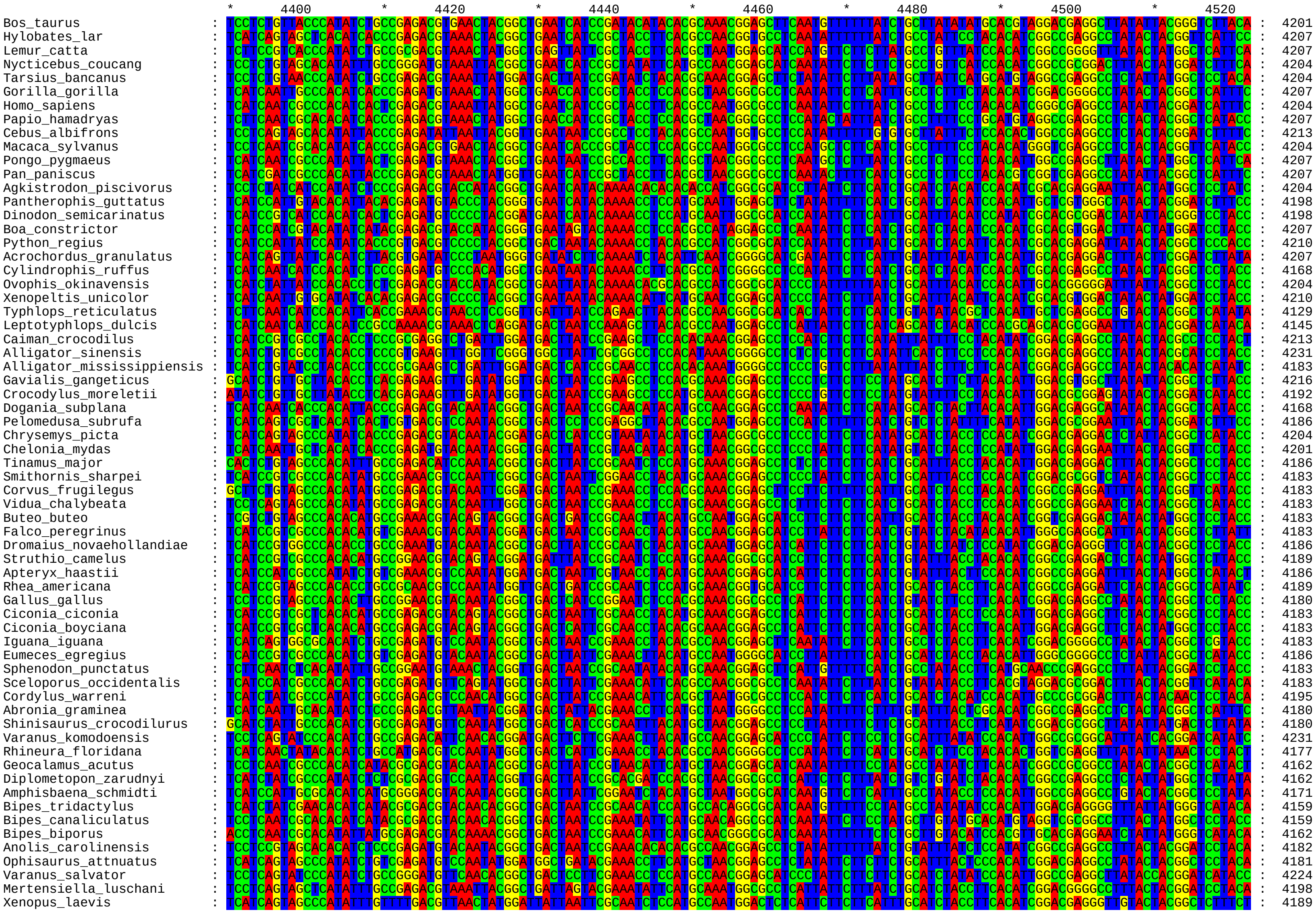

4400 4420 4440 4460 4480 4500 4520
Bos_taurus : 4201
Hylobates_lar : 4207
Lemur_catta : 4207
Nycticebus_coucang : 4204
Tarsius_bancanus : 4204
Gorilla_gorilla : 4207
Homo_sapiens : 4204
Papio_hamadryas : 4207
Cebus_albifrons : 4213
Macaca_sylvanus : 4204
Pongo_pygmaeus : 4207
Pan_paniscus : 4207
Agkistrodon_piscivorus : 4204
Pantherophis_guttatus : 4198
Dinodon_semicarinatus : 4198
Boa_constrictor : 4207
Python_regius : 4210
Acrochordus_granulatus : 4207
Cylindrophis_ruffus : 4168
Ovophis_okinavensis : 4204
Xenopeltis_unicolor : 4210
Typhlops_reticulatus : 4129
Leptotyphlops_dulcis : 4145
Caiman_crocodilus : 4213
Alligator_sinensis : 4231
Alligator_mississippiensis : 4183
Gavialis_gangeticus : 4216
Crocodylus_moreletii : 4192
Dogania_subplana : 4168
Pelomedusa_subrufa : 4186
Chrysemys_picta : 4204
Chelonia_mydas : 4201
Tinamus_major : 4186
Smithornis_sharpei : 4183
Corvus_frugilegus : 4183
Vidua_chalybeata : 4183
Buteo_buteo : 4183
Falco_peregrinus : 4183
Dromaius_novaehollandiae : 4186
Struthio_camelus : 4189
Apteryx_haastii : 4186
Rhea_americana : 4189
Gallus_gallus : 4180
Ciconia_ciconia : 4183
Ciconia_boyciana : 4183
Iguana_iguana : 4183
Eumeces_egregius : 4186
Sphenodon_punctatus : 4183
Sceloporus_occidentalis : 4183
Cordylus_warreni : 4195
Abronia_graminea : 4180
Shinisaurus_crocodilurus : 4180
Varanus_komodoensis : 4231
Rhineura_floridana : 4177
Geocalamus_acutus : 4162
Diplometopon_zarudnyi : 4162
Amphisbaena_schmidti : 4171
Bipes_tridactylus : 4159
Bipes_canaliculatus : 4159
Bipes_biporus : 4162
Anolis_carolinensis : 4182
Ophisaurus_attnuatus : 4181
Varanus_salvator : 4224
Mertensiella_luschani : 4198
Xenopus_laevis : 4189

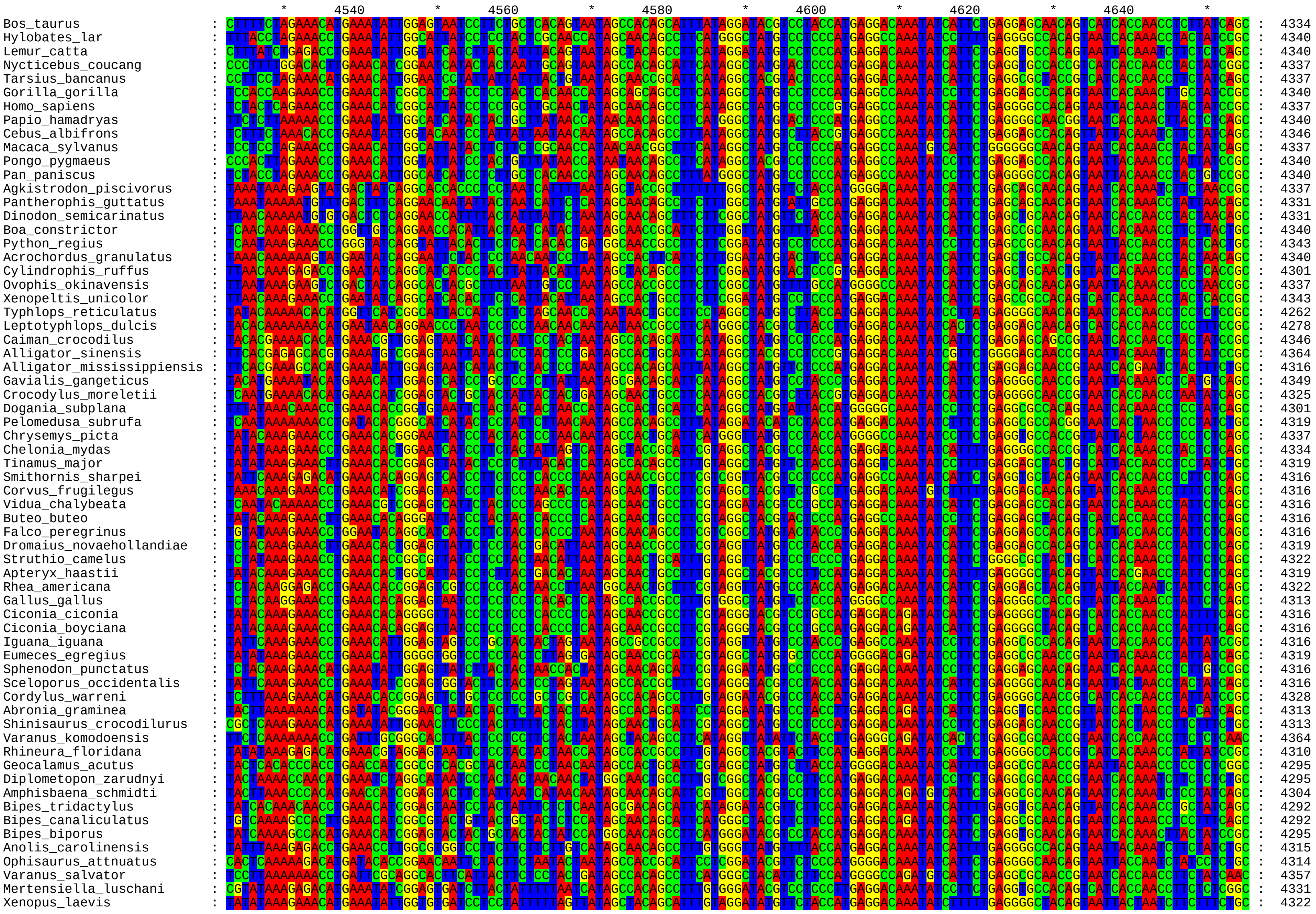
* 4540 * 4560 * 4580 * 4600 * 4620 * 4640 *
Bos_taurus : 4334
Hylobates_lar : 4340
Lemur_catta : 4340
Nycticebus_coucang : 4337
Tarsius_bancanus : 4337
Gorilla_gorilla : 4340
Homo_sapiens : 4337
Papio_hamadryas : 4340
Cebus_albifrons : 4346
Macaca_sylvanus : 4337
Pongo_pygmaeus : 4340
Pan_paniscus : 4340
Agkistrodon_piscivorus : 4337
Pantherophis_guttatus : 4331
Dinodon_semicarinatus : 4331
Boa_constrictor : 4340
Python_regius : 4343
Acrochordus_granulatus : 4340
Cylindrophis_ruffus : 4301
Ovophis_okinavensis : 4337
Xenopeltis_unicolor : 4343
Typhlops_reticulatus : 4262
Leptotyphlops_dulcis : 4278
Caiman_crocodilus : 4346
Alligator_sinensis : 4364
Alligator_mississippiensis : 4316
Gavialis_gangeticus : 4349
Crocodylus_moreletii : 4325
Dogania_subplana : 4301
Pelomedusa_subrufa : 4319
Chrysemys_picta : 4337
Chelonia_mydas : 4334
Tinamus_major : 4319
Smithornis_sharpei : 4316
Corvus_frugilegus : 4316
Vidua_chalybeata : 4316
Buteo_buteo : 4316
Falco_peregrinus : 4316
Dromaius_novaehollandiae : 4319
Struthio_camelus : 4322
Apteryx_haastii : 4319
Rhea_americana : 4322
Gallus_gallus : 4313
Ciconia_ciconia : 4316
Ciconia_boyciana : 4316
Iguana_iguana : 4316
Eumeces_egregius : 4319
Sphenodon_punctatus : 4316
Sceloporus_occidentalis : 4316
Cordylus_warreni : 4328
Abronia_graminea : 4313
Shinisaurus_crocodilurus : 4313
Varanus_komodoensis : 4364
Rhineura_floridana : 4310
Geocalamus_acutus : 4295
Diplometopon_zarudnyi : 4295
Amphisbaena_schmidti : 4304
Bipes_tridactylus : 4292
Bipes_canaliculatus : 4292
Bipes_biporus : 4295
Anolis_carolinensis : 4315
Ophisaurus_attnuatus : 4314
Varanus_salvator : 4357
Mertensiella_luschani : 4331
Xenopus_laevis : 4322

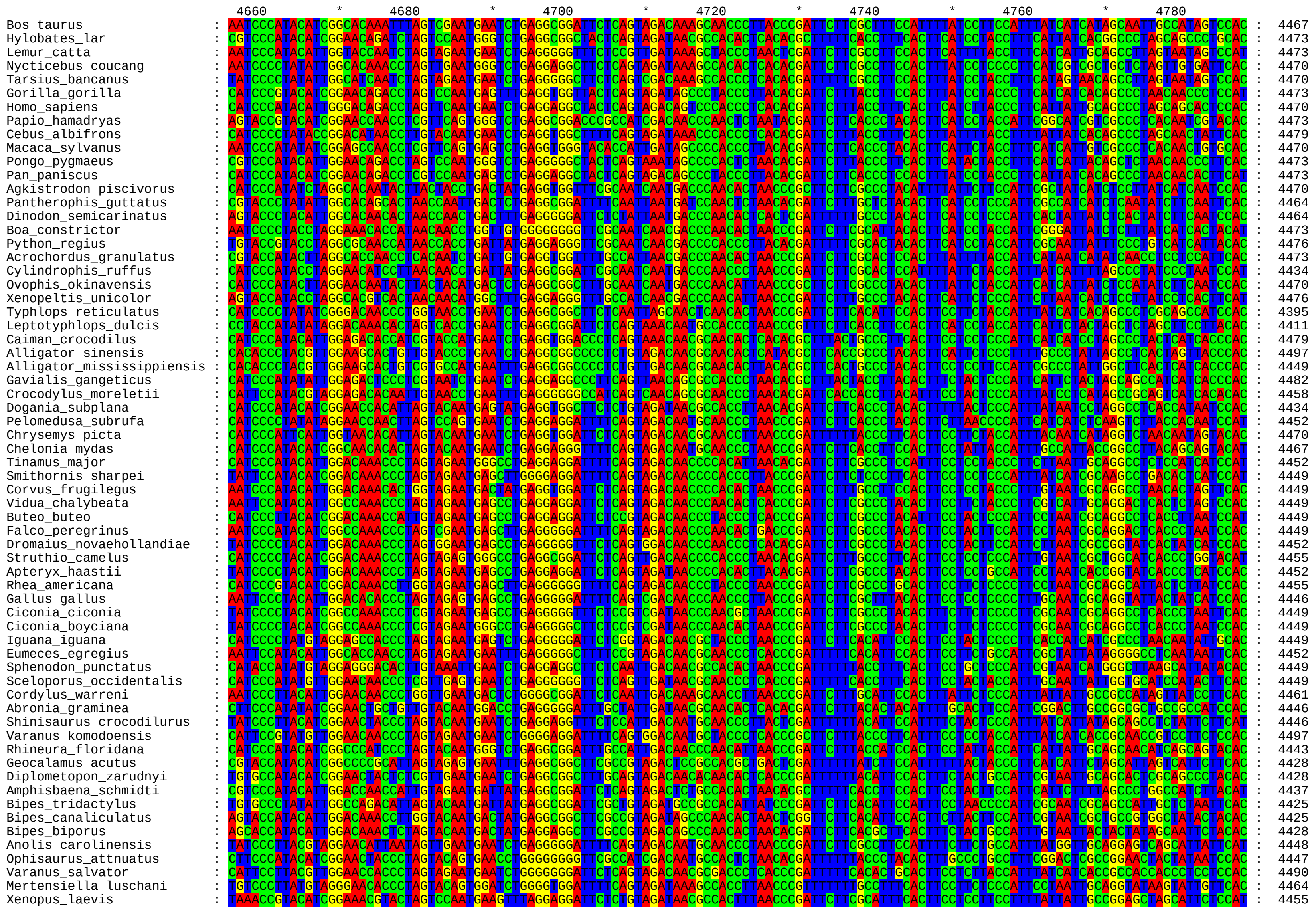
4660 * 4680 * 4700 * 4720 * 4740 * 4760 * 4780
Bos_taurus : 4467
Hylobates_lar : 4473
Lemur_catta : 4473
Nycticebus_coucang : 4470
Tarsius_bancanus : 4470
Gorilla_gorilla : 4473
Homo_sapiens : 4470
Papio_hamadryas : 4473
Cebus_albifrons : 4479
Macaca_sylvanus : 4470
Pongo_pygmaeus : 4473
Pan_paniscus : 4473
Agkistrodon_piscivorus : 4470
Pantherophis_guttatus : 4464
Dinodon_semicarinatus : 4464
Boa_constrictor : 4473
Python_regius : 4476
Acrochordus_granulatus : 4473
Cylindrophis_ruffus : 4434
Ovophis_okinavensis : 4470
Xenopeltis_unicolor : 4476
Typhlops_reticulatus : 4395
Leptotyphlops_dulcis : 4411
Caiman_crocodilus : 4479
Alligator_sinensis : 4497
Alligator_mississippiensis : 4449
Gavialis_gangeticus : 4482
Crocodylus_moreletii : 4458
Dogania_subplana : 4434
Pelomedusa_subrufa : 4452
Chrysemys_picta : 4470
Chelonia_mydas : 4467
Tinamus_major : 4452
Smithornis_sharpei : 4449
Corvus_frugilegus : 4449
Vidua_chalybeata : 4449
Buteo_buteo : 4449
Falco_peregrinus : 4449
Dromaius_novaehollandiae : 4452
Struthio_camelus : 4455
Apteryx_haastii : 4452
Rhea_americana : 4455
Gallus_gallus : 4446
Ciconia_ciconia : 4449
Ciconia_boyciana : 4449
Iguana_iguana : 4449
Eumeces_egregius : 4452
Sphenodon_punctatus : 4449
Sceloporus_occidentalis : 4449
Cordylus_warreni : 4461
Abronia_graminea : 4446
Shinisaurus_crocodilurus : 4446
Varanus_komodoensis : 4497
Rhineura_floridana : 4443
Geocalamus_acutus : 4428
Diplometopon_zarudnyi : 4428
Amphisbaena_schmidti : 4437
Bipes_tridactylus : 4425
Bipes_canaliculatus : 4425
Bipes_biporus : 4428
Anolis_carolinensis : 4448
Ophisaurus_attnuatus : 4447
Varanus_salvator : 4490
Mertensiella_luschani : 4464
Xenopus_laevis : 4455

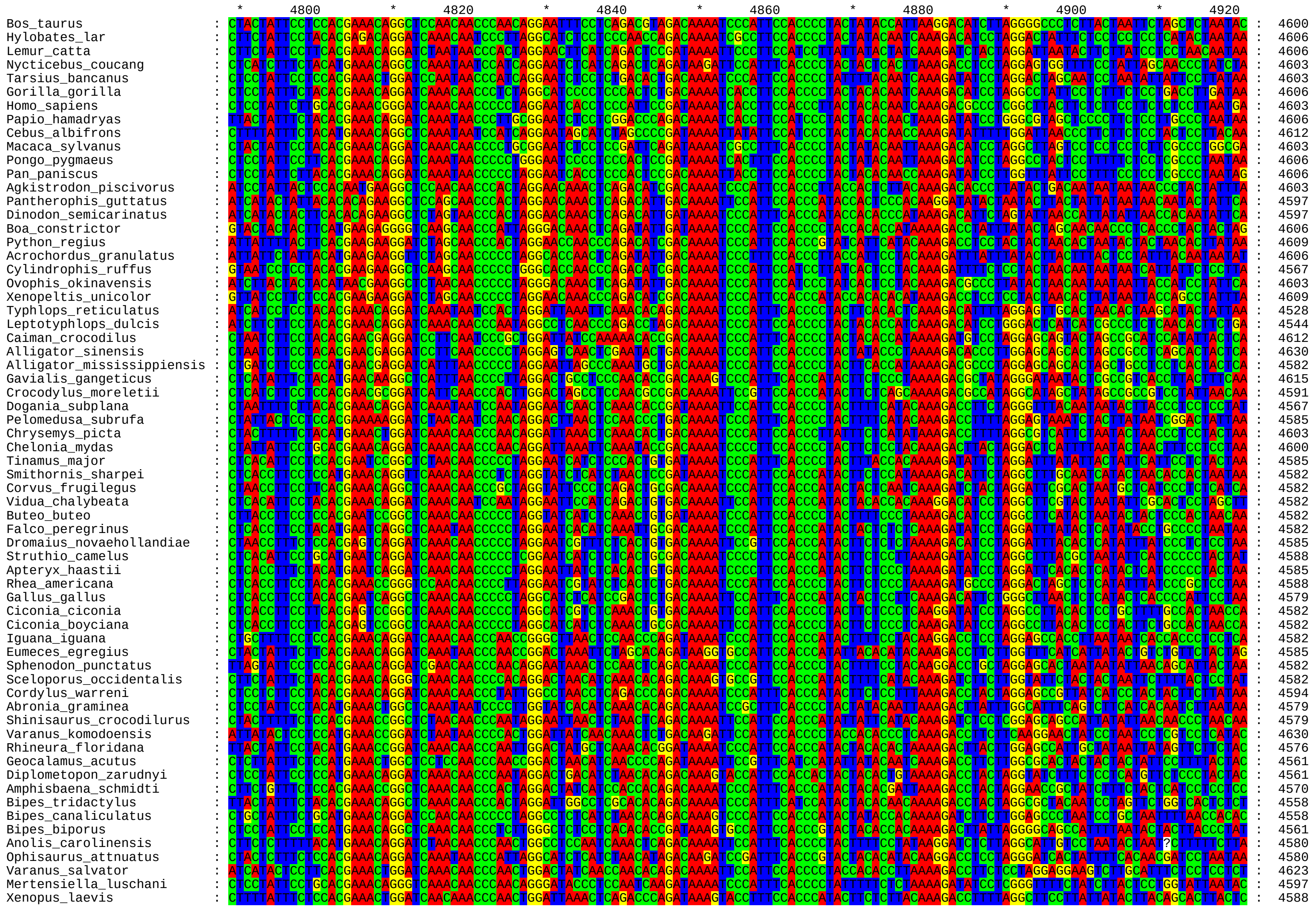
* 4800 * 4820 * 4840 * 4860 * 4880 * 4900 * 4920
Bos_taurus : 4600
Hylobates_lar : 4606
Lemur_catta : 4606
Nycticebus_coucang : 4603
Tarsius_bancanus : 4603
Gorilla_gorilla : 4606
Homo_sapiens : 4603
Papio_hamadryas : 4606
Cebus_albifrons : 4612
Macaca_sylvanus : 4603
Pongo_pygmaeus : 4606
Pan_paniscus : 4606
Agkistrodon_piscivorus : 4603
Pantherophis_guttatus : 4597
Dinodon_semicarinatus : 4597
Boa_constrictor : 4606
Python_regius : 4609
Acrochordus_granulatus : 4606
Cylindrophis_ruffus : 4567
Ovophis_okinavensis : 4603
Xenopeltis_unicolor : 4609
Typhlops_reticulatus : 4528
Leptotyphlops_dulcis : 4544
Caiman_crocodilus : 4612
Alligator_sinensis : 4630
Alligator_mississippiensis : 4582
Gavialis_gangeticus : 4615
Crocodylus_moreletii : 4591
Dogania_subplana : 4567
Pelomedusa_subrufa : 4585
Chrysemys_picta : 4603
Chelonia_mydas : 4600
Tinamus_major : 4585
Smithornis_sharpei : 4582
Corvus_frugilegus : 4582
Vidua_chalybeata : 4582
Buteo_buteo : 4582
Falco_peregrinus : 4582
Dromaius_novaehollandiae : 4585
Struthio_camelus : 4588
Apteryx_haastii : 4585
Rhea_americana : 4588
Gallus_gallus : 4579
Ciconia_ciconia : 4582
Ciconia_boyciana : 4582
Iguana_iguana : 4582
Eumeces_egregius : 4585
Sphenodon_punctatus : 4582
Sceloporus_occidentalis : 4582
Cordylus_warreni : 4594
Abronia_graminea : 4579
Shinisaurus_crocodilurus : 4579
Varanus_komodoensis : 4630
Rhineura_floridana : 4576
Geocalamus_acutus : 4561
Diplometopon_zarudnyi : 4561
Amphisbaena_schmidti : 4570
Bipes_tridactylus : 4558
Bipes_canaliculatus : 4558
Bipes_biporus : 4561
Anolis_carolinensis : 4580
Ophisaurus_attnuatus : 4580
Varanus_salvator : 4623
Mertensiella_luschani : 4597
Xenopus_laevis : 4588

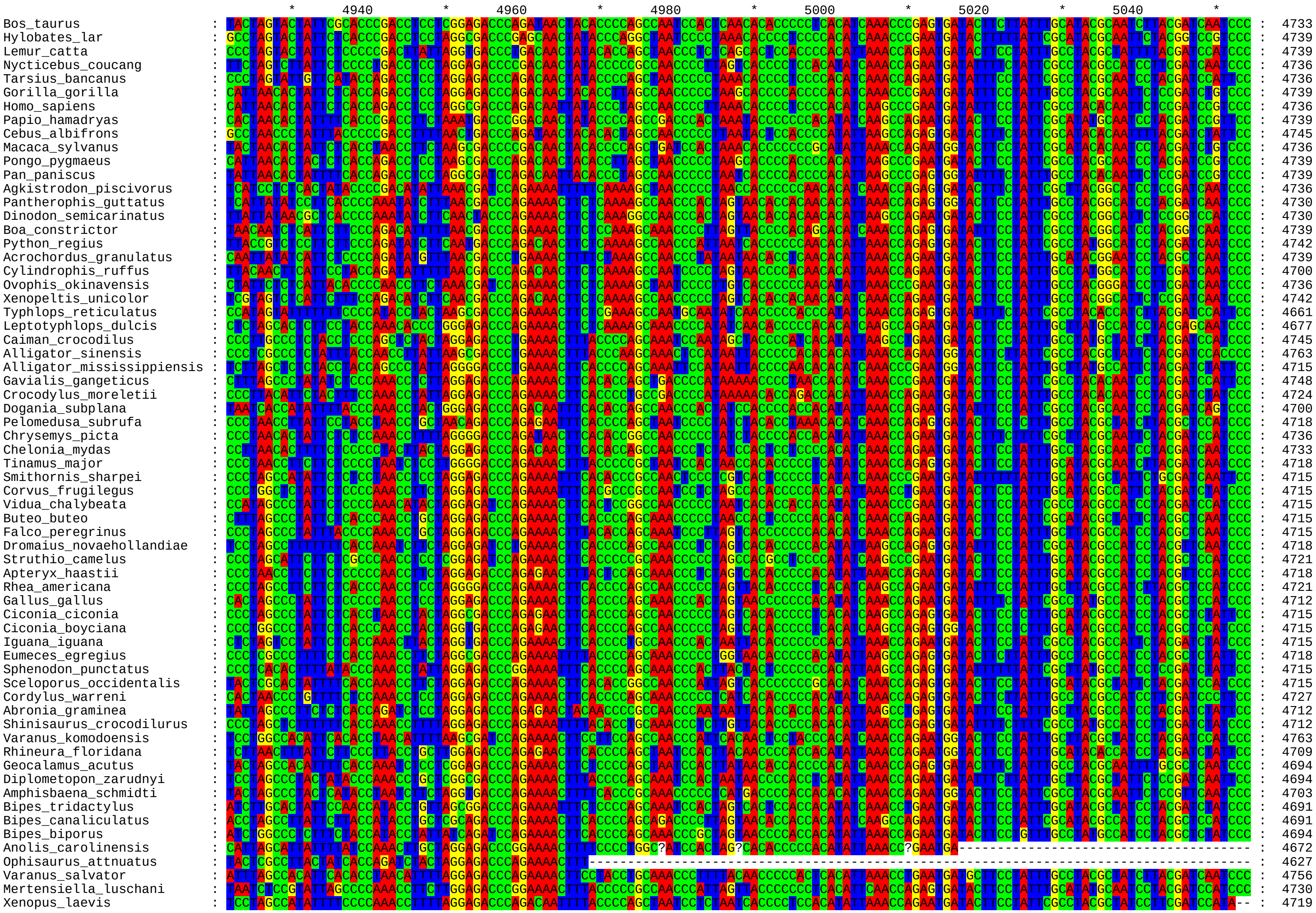
* 4940 * 4960 * 4980 * 5000 * 5020 * 5040 *
Bos_taurus : 4733
Hylobates_lar : 4739
Lemur_catta : 4739
Nycticebus_coucang : 4736
Tarsius_bancanus : 4736
Gorilla_gorilla : 4739
Homo_sapiens : 4736
Papio_hamadryas : 4739
Cebus_albifrons : 4745
Macaca_sylvanus : 4736
Pongo_pygmaeus : 4739
Pan_paniscus : 4739
Agkistrodon_piscivorus : 4736
Pantherophis_guttatus : 4730
Dinodon_semicarinatus : 4730
Boa_constrictor : 4739
Python_regius : 4742
Acrochordus_granulatus : 4739
Cylindrophis_ruffus : 4700
Ovophis_okinavensis : 4736
Xenopeltis_unicolor : 4742
Typhlops_reticulatus : 4661
Leptotyphlops_dulcis : 4677
Caiman_crocodilus : 4745
Alligator_sinensis : 4763
Alligator_mississippiensis : 4715
Gavialis_gangeticus : 4748
Crocodylus_moreletii : 4724
Dogania_subplana : 4700
Pelomedusa_subrufa : 4718
Chrysemys_picta : 4736
Chelonia_mydas : 4733
Tinamus_major : 4718
Smithornis_sharpei : 4715
Corvus_frugilegus : 4715
Vidua_chalybeata : 4715
Buteo_buteo : 4715
Falco_peregrinus : 4715
Dromaius_novaehollandiae : 4718
Struthio_camelus : 4721
Apteryx_haastii : 4718
Rhea_americana : 4721
Gallus_gallus : 4712
Ciconia_ciconia : 4715
Ciconia_boyciana : 4715
Iguana_iguana : 4715
Eumeces_egregius : 4718
Sphenodon_punctatus : 4715
Sceloporus_occidentalis : 4715
Cordylus_warreni : 4727
Abronia_graminea : 4712
Shinisaurus_crocodilurus : 4712
Varanus_komodoensis : 4763
Rhineura_floridana : 4709
Geocalamus_acutus : 4694
Diplometopon_zarudnyi : 4694
Amphisbaena_schmidti : 4703
Bipes_tridactylus : 4691
Bipes_canaliculatus : 4691
Bipes_biporus : 4694
Anolis_carolinensis : 4672
Ophisaurus_attnuatus : 4627
Varanus_salvator : 4756
Mertensiella_luschani : 4730
Xenopus_laevis : 4719

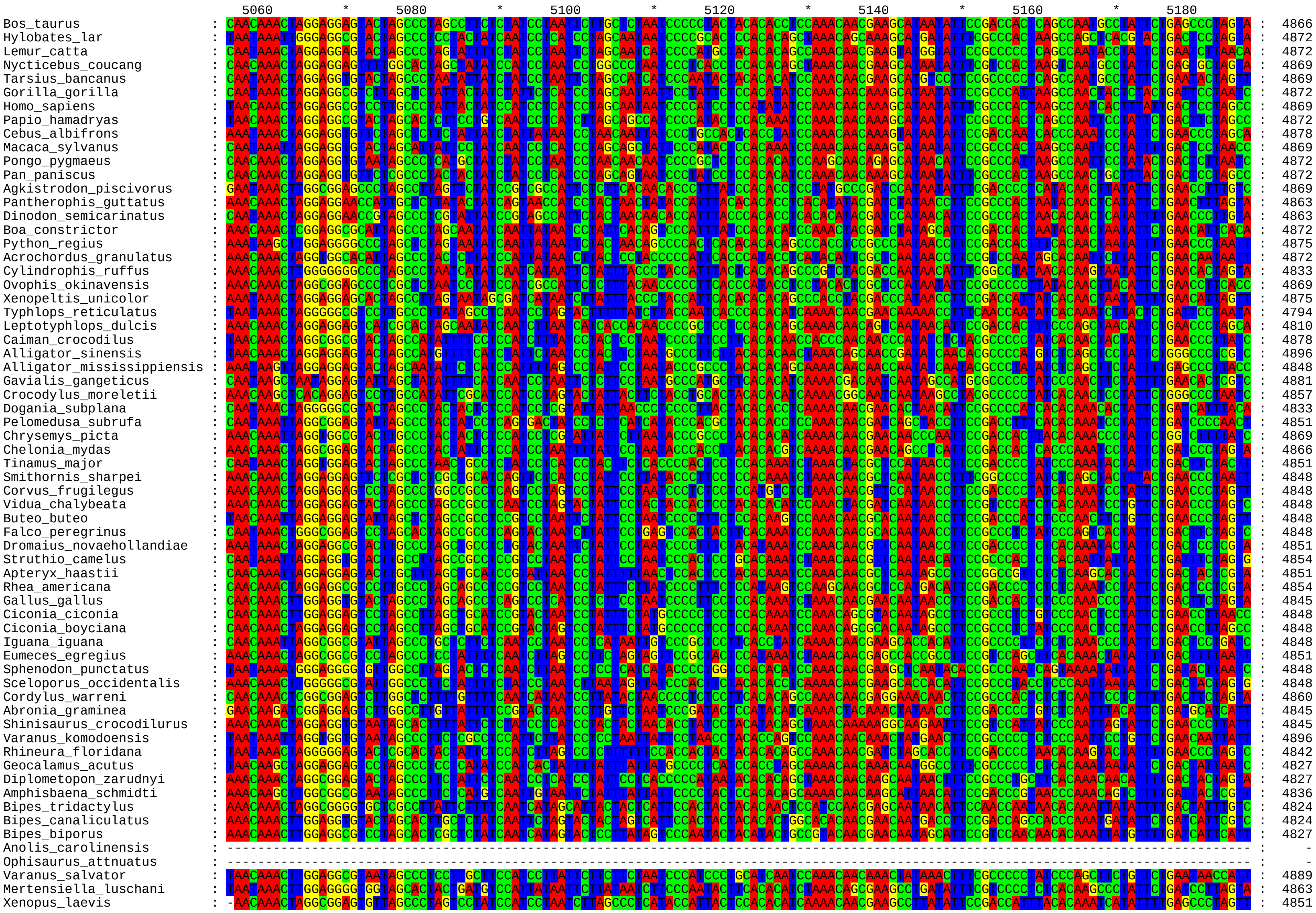
5060 * 5080 * 5100 * 5120 * 5140 * 5160 * 5180
Bos_taurus : 4866
Hylobates_lar : 4872
Lemur_catta : 4872
Nycticebus_coucang : 4869
Tarsius_bancanus : 4869
Gorilla_gorilla : 4872
Homo_sapiens : 4869
Papio_hamadryas : 4872
Cebus_albifrons : 4878
Macaca_sylvanus : 4869
Pongo_pygmaeus : 4872
Pan_paniscus : 4872
Agkistrodon_piscivorus : 4869
Pantherophis_guttatus : 4863
Dinodon_semicarinatus : 4863
Boa_constrictor : 4872
Python_regius : 4875
Acrochordus_granulatus : 4872
Cylindrophis_ruffus : 4833
Ovophis_okinavensis : 4869
Xenopeltis_unicolor : 4875
Typhlops_reticulatus : 4794
Leptotyphlops_dulcis : 4810
Caiman_crocodilus : 4878
Alligator_sinensis : 4896
Alligator_mississippiensis : 4848
Gavialis_gangeticus : 4881
Crocodylus_moreletii : 4857
Dogania_subplana : 4833
Pelomedusa_subrufa : 4851
Chrysemys_picta : 4869
Chelonia_mydas : 4866
Tinamus_major : 4851
Smithornis_sharpei : 4848
Corvus_frugilegus : 4848
Vidua_chalybeata : 4848
Buteo_buteo : 4848
Falco_peregrinus : 4848
Dromaius_novaehollandiae : 4851
Struthio_camelus : 4854
Apteryx_haastii : 4851
Rhea_americana : 4854
Gallus_gallus : 4845
Ciconia_ciconia : 4848
Ciconia_boyciana : 4848
Iguana_iguana : 4848
Eumeces_egregius : 4851
Sphenodon_punctatus : 4848
Sceloporus_occidentalis : 4848
Cordylus_warreni : 4860
Abronia_graminea : 4845
Shinisaurus_crocodilurus : 4845
Varanus_komodoensis : 4896
Rhineura_floridana : 4842
Geocalamus_acutus : 4827
Diplometopon_zarudnyi : 4827
Amphisbaena_schmidti : 4836
Bipes_tridactylus : 4824
Bipes_canaliculatus : 4824
Bipes_biporus : 4827
Anolis_carolinensis : -
Ophisaurus_attnuatus : -
Varanus_salvator : 4889
Mertensiella_luschani : 4863
Xenopus_laevis : 4851

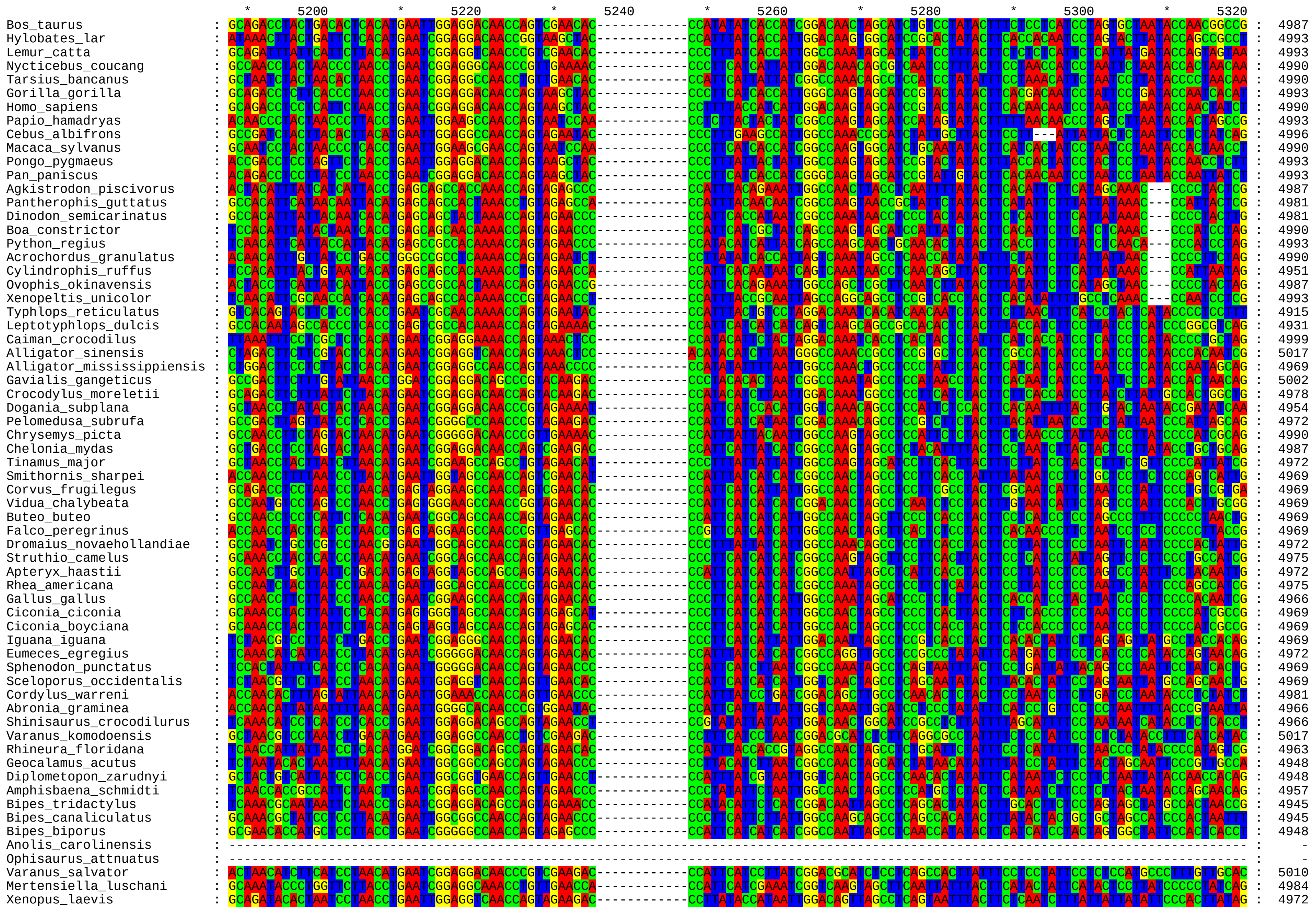
* 5200 * 5220 * 5240 * 5260 * 5280 * 5300 * 5320
Bos_taurus : 4987
Hylobates_lar : 4993
Lemur_catta : 4993
Nycticebus_coucang : 4990
Tarsius_bancanus : 4990
Gorilla_gorilla : 4993
Homo_sapiens : 4990
Papio_hamadryas : 4993
Cebus_albifrons : 4996
Macaca_sylvanus : 4990
Pongo_pygmaeus : 4993
Pan_paniscus : 4993
Agkistrodon_piscivorus : 4987
Pantherophis_guttatus : 4981
Dinodon_semicarinatus : 4981
Boa_constrictor : 4990
Python_regius : 4993
Acrochordus_granulatus : 4990
Cylindrophis_ruffus : 4951
Ovophis_okinavensis : 4987
Xenopeltis_unicolor : 4993
Typhlops_reticulatus : 4915
Leptotyphlops_dulcis : 4931
Caiman_crocodilus : 4999
Alligator_sinensis : 5017
Alligator_mississippiensis : 4969
Gavialis_gangeticus : 5002
Crocodylus_moreletii : 4978
Dogania_subplana : 4954
Pelomedusa_subrufa : 4972
Chrysemys_picta : 4990
Chelonia_mydas : 4987
Tinamus_major : 4972
Smithornis_sharpei : 4969
Corvus_frugilegus : 4969
Vidua_chalybeata : 4969
Buteo_buteo : 4969
Falco_peregrinus : 4969
Dromaius_novaehollandiae : 4972
Struthio_camelus : 4975
Apteryx_haastii : 4972
Rhea_americana : 4975
Gallus_gallus : 4966
Ciconia_ciconia : 4969
Ciconia_boyciana : 4969
Iguana_iguana : 4969
Eumeces_egregius : 4972
Sphenodon_punctatus : 4969
Sceloporus_occidentalis : 4969
Cordylus_warreni : 4981
Abronia_graminea : 4966
Shinisaurus_crocodilurus : 4966
Varanus_komodoensis : 5017
Rhineura_floridana : 4963
Geocalamus_acutus : 4948
Diplometopon_zarudnyi : 4948
Amphisbaena_schmidti : 4957
Bipes_tridactylus : 4945
Bipes_canaliculatus : 4945
Bipes_biporus : 4948
Anolis_carolinensis : -
Ophisaurus_attnuatus : -
Varanus_salvator : 5010
Mertensiella_luschani : 4984
Xenopus_laevis : 4972

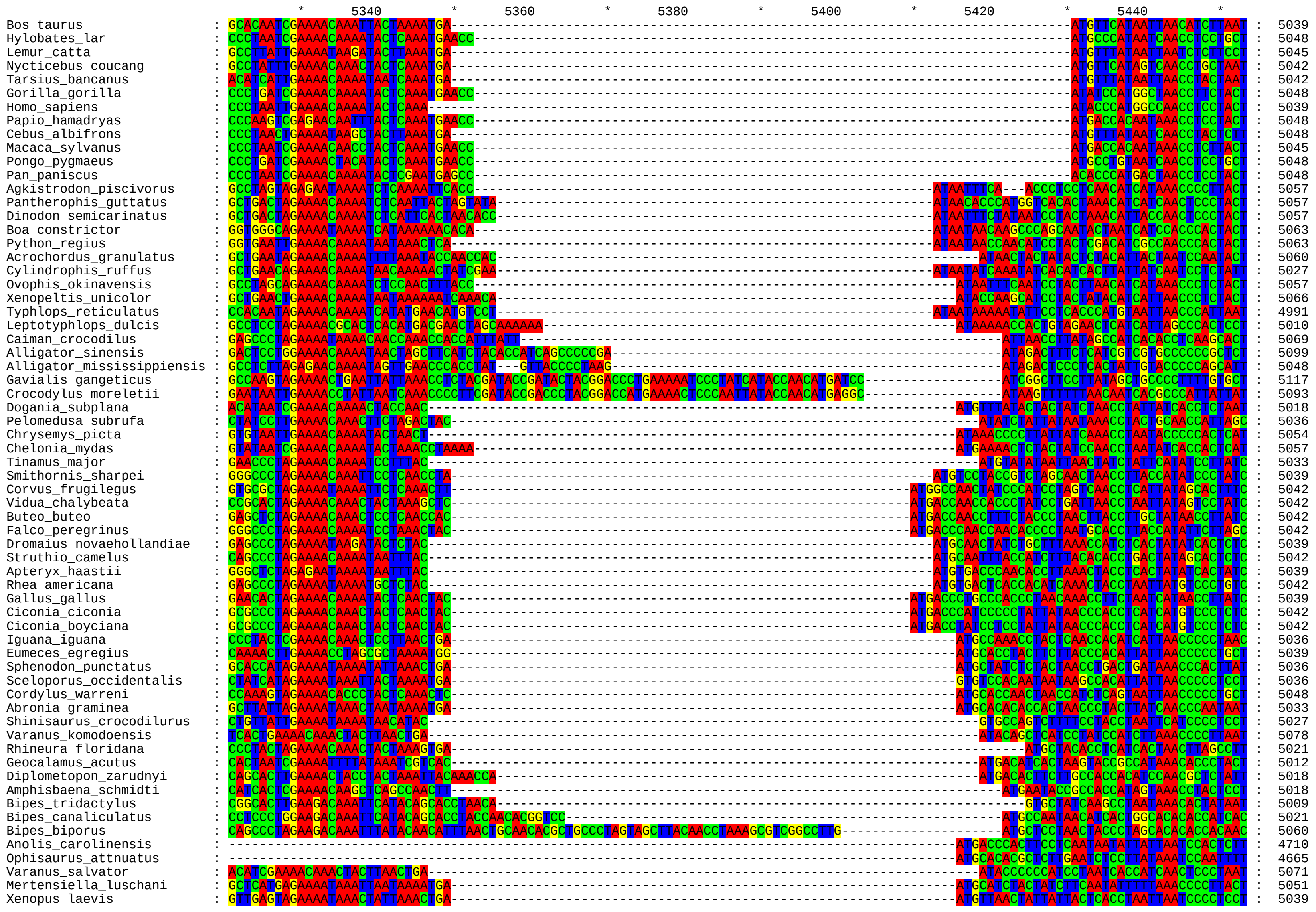

* 5340 * 5360 * 5380 * 5400 * 5420 * 5440 *
Bos_taurus : 5039
Hylobates_lar : 5048
Lemur_catta : 5045
Nycticebus_coucang : 5042
Tarsius_bancanus : 5042
Gorilla_gorilla : 5048
Homo_sapiens : 5039
Papio_hamadryas : 5048
Cebus_albifrons : 5048
Macaca_sylvanus : 5045
Pongo_pygmaeus : 5048
Pan_paniscus : 5048
Agkistrodon_piscivorus : 5057
Pantherophis_guttatus : 5057
Dinodon_semicarinatus : 5057
Boa_constrictor : 5063
Python_regius : 5063
Acrochordus_granulatus : 5060
Cylindrophis_ruffus : 5027
Ovophis_okinavensis : 5057
Xenopeltis_unicolor : 5066
Typhlops_reticulatus : 4991
Leptotyphlops_dulcis : 5010
Caiman_crocodilus : 5069
Alligator_sinensis : 5099
Alligator_mississippiensis : 5048
Gavialis_gangeticus : 5117
Crocodylus_moreletii : 5093
Dogania_subplana : 5018
Pelomedusa_subrufa : 5036
Chrysemys_picta : 5054
Chelonia_mydas : 5057
Tinamus_major : 5033
Smithornis_sharpei : 5039
Corvus_frugilegus : 5042
Vidua_chalybeata : 5042
Buteo_buteo : 5042
Falco_peregrinus : 5042
Dromaius_novaehollandiae : 5039
Struthio_camelus : 5042
Apteryx_haastii : 5039
Rhea_americana : 5042
Gallus_gallus : 5039
Ciconia_ciconia : 5042
Ciconia_boyciana : 5042
Iguana_iguana : 5036
Eumeces_egregius : 5039
Sphenodon_punctatus : 5036
Sceloporus_occidentalis : 5036
Cordylus_warreni : 5048
Abronia_graminea : 5033
Shinisaurus_crocodilurus : 5027
Varanus_komodoensis : 5078
Rhineura_floridana : 5021
Geocalamus_acutus : 5012
Diplometopon_zarudnyi : 5018
Amphisbaena_schmidti : 5018
Bipes_tridactylus : 5009
Bipes_canaliculatus : 5021
Bipes_biporus : 5060
Anolis_carolinensis : 4710
Ophisaurus_attnuatus : 4665
Varanus_salvator : 5071
Mertensiella_luschani : 5051
Xenopus_laevis : 5039

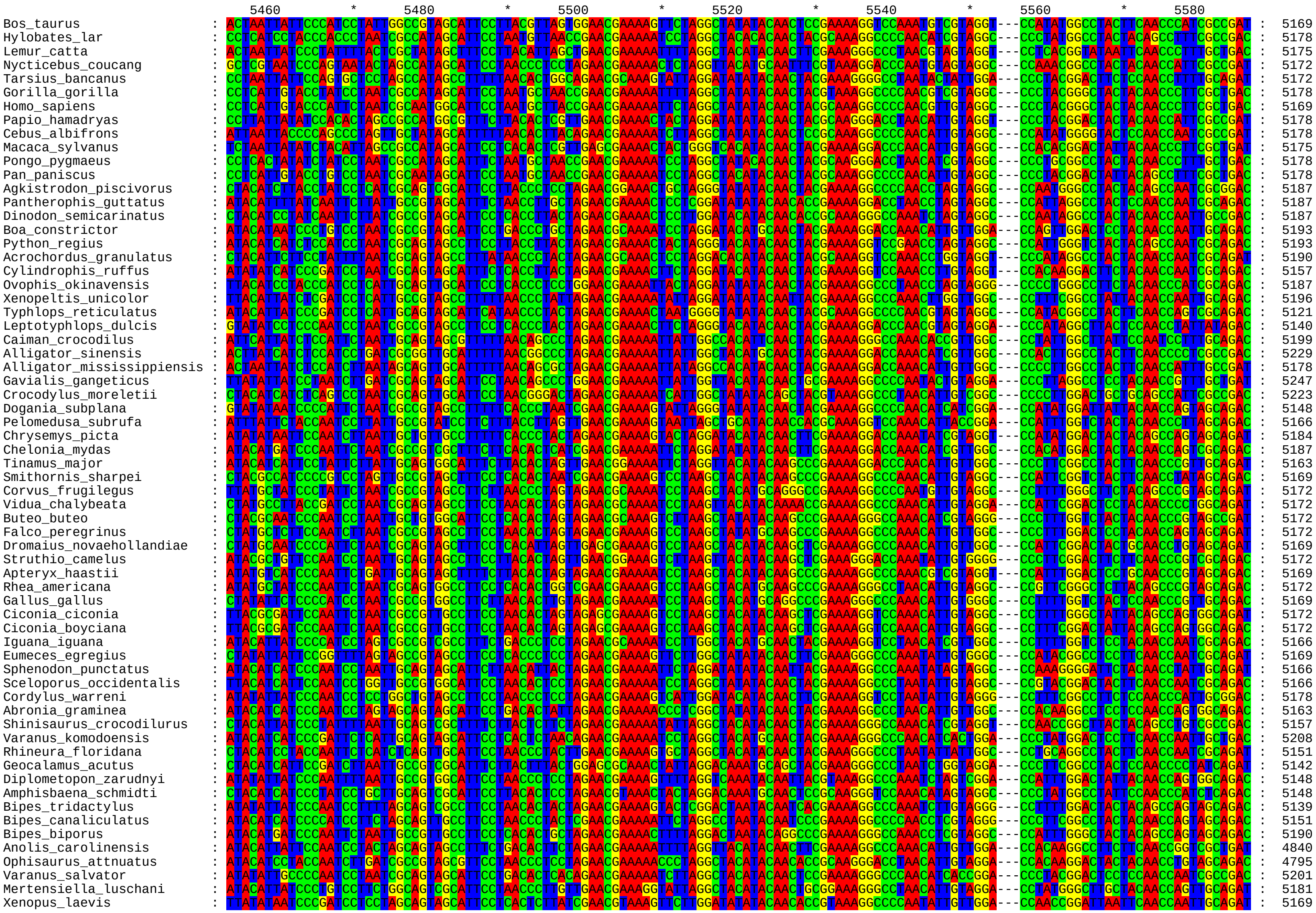
5460 * 5480 * 5500 * 5520 * 5540 * 5560 * 5580
Bos_taurus : 5169
Hylobates_lar : 5178
Lemur_catta : 5175
Nycticebus_coucang : 5172
Tarsius_bancanus : 5172
Gorilla_gorilla : 5178
Homo_sapiens : 5169
Papio_hamadryas : 5178
Cebus_albifrons : 5178
Macaca_sylvanus : 5175
Pongo_pygmaeus : 5178
Pan_paniscus : 5178
Agkistrodon_piscivorus : 5187
Pantherophis_guttatus : 5187
Dinodon_semicarinatus : 5187
Boa_constrictor : 5193
Python_regius : 5193
Acrochordus_granulatus : 5190
Cylindrophis_ruffus : 5157
Ovophis_okinavensis : 5187
Xenopeltis_unicolor : 5196
Typhlops_reticulatus : 5121
Leptotyphlops_dulcis : 5140
Caiman_crocodilus : 5199
Alligator_sinensis : 5229
Alligator_mississippiensis : 5178
Gavialis_gangeticus : 5247
Crocodylus_moreletii : 5223
Dogania_subplana : 5148
Pelomedusa_subrufa : 5166
Chrysemys_picta : 5184
Chelonia_mydas : 5187
Tinamus_major : 5163
Smithornis_sharpei : 5169
Corvus_frugilegus : 5172
Vidua_chalybeata : 5172
Buteo_buteo : 5172
Falco_peregrinus : 5172
Dromaius_novaehollandiae : 5169
Struthio_camelus : 5172
Apteryx_haastii : 5169
Rhea_americana : 5172
Gallus_gallus : 5169
Ciconia_ciconia : 5172
Ciconia_boyciana : 5172
Iguana_iguana : 5166
Eumeces_egregius : 5169
Sphenodon_punctatus : 5166
Sceloporus_occidentalis : 5166
Cordylus_warreni : 5178
Abronia_graminea : 5163
Shinisaurus_crocodilurus : 5157
Varanus_komodoensis : 5208
Rhineura_floridana : 5151
Geocalamus_acutus : 5142
Diplometopon_zarudnyi : 5148
Amphisbaena_schmidti : 5148
Bipes_tridactylus : 5139
Bipes_canaliculatus : 5151
Bipes_biporus : 5190
Anolis_carolinensis : 4840
Ophisaurus_attnuatus : 4795
Varanus_salvator : 5201
Mertensiella_luschani : 5181
Xenopus_laevis : 5169

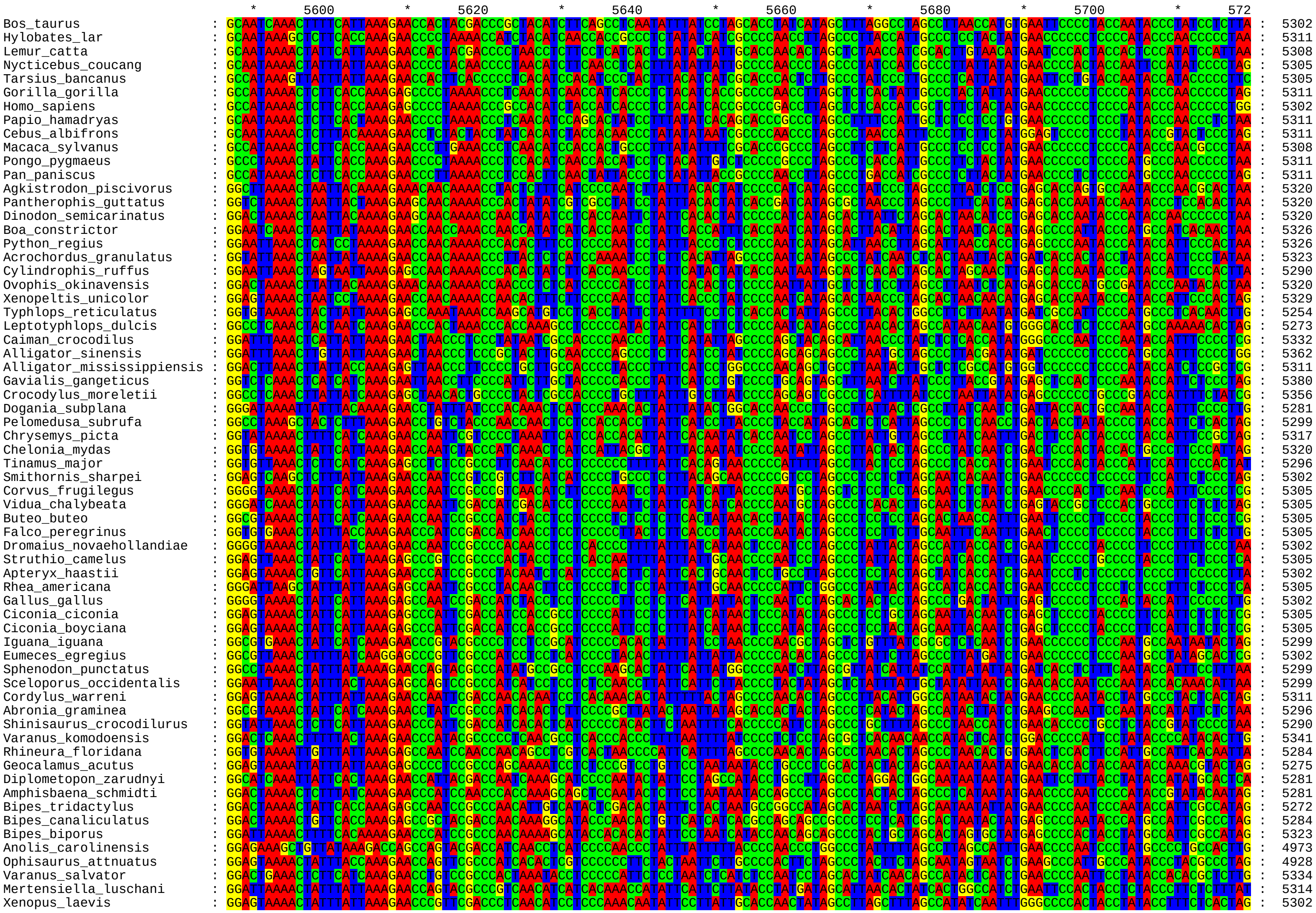
* 5600 * 5620 * 5640 * 5660 * 5680 * 5700 * 572
Bos_taurus 5302
Hylobates_lar 5311
Lemur_catta 5308
Nycticebus_coucang 5305
Tarsius_bancanus 5305
Gorilla_gorilla 5311
Homo_sapiens 5302
Papio_hamadryas 5311
Cebus_albifrons 5311
Macaca_sylvanus 5308
Pongo_pygmaeus 5311
Pan_paniscus 5311
Agkistrodon_piscivorus 5320
Pantherophis_guttatus 5320
Dinodon_semicarinatus 5320
Boa_constrictor 5326
Python_regius 5326
Acrochordus_granulatus 5323
Cylindrophis_ruffus 5290
Ovophis_okinavensis 5320
Xenopeltis_unicolor 5329
Typhlops_reticulatus 5254
Leptotyphlops_dulcis 5273
Caiman_crocodilus 5332
Alligator_sinensis 5362
Alligator_mississippiensis 5311
Gavialis_gangeticus 5380
Crocodylus_moreletii 5356
Dogania_subplana 5281
Pelomedusa_subrufa 5299
Chrysemys_picta 5317
Chelonia_mydas 5320
Tinamus_major 5296
Smithornis_sharpei 5302
Corvus_frugilegus 5305
Vidua_chalybeata 5305
Buteo_buteo 5305
Falco_peregrinus 5305
Dromaius_novaehollandiae 5302
Struthio_camelus 5305
Apteryx_haastii 5302
Rhea_americana 5305
Gallus_gallus 5302
Ciconia_ciconia 5305
Ciconia_boyciana 5305
Iguana_iguana 5299
Eumeces_egregius 5302
Sphenodon_punctatus 5299
Sceloporus_occidentalis 5299
Cordylus_warreni 5311
Abronia_graminea 5296
Shinisaurus_crocodilurus 5290
Varanus_komodoensis 5341
Rhineura_floridana 5284
Geocalamus_acutus 5275
Diplometopon_zarudnyi 5281
Amphisbaena_schmidti 5281
Bipes_tridactylus 5272
Bipes_canaliculatus 5284
Bipes_biporus 5323
Anolis_carolinensis 4973
Ophisaurus_attnuatus 4928
Varanus_salvator 5334
Mertensiella_luschani 5314
Xenopus_laevis 5302

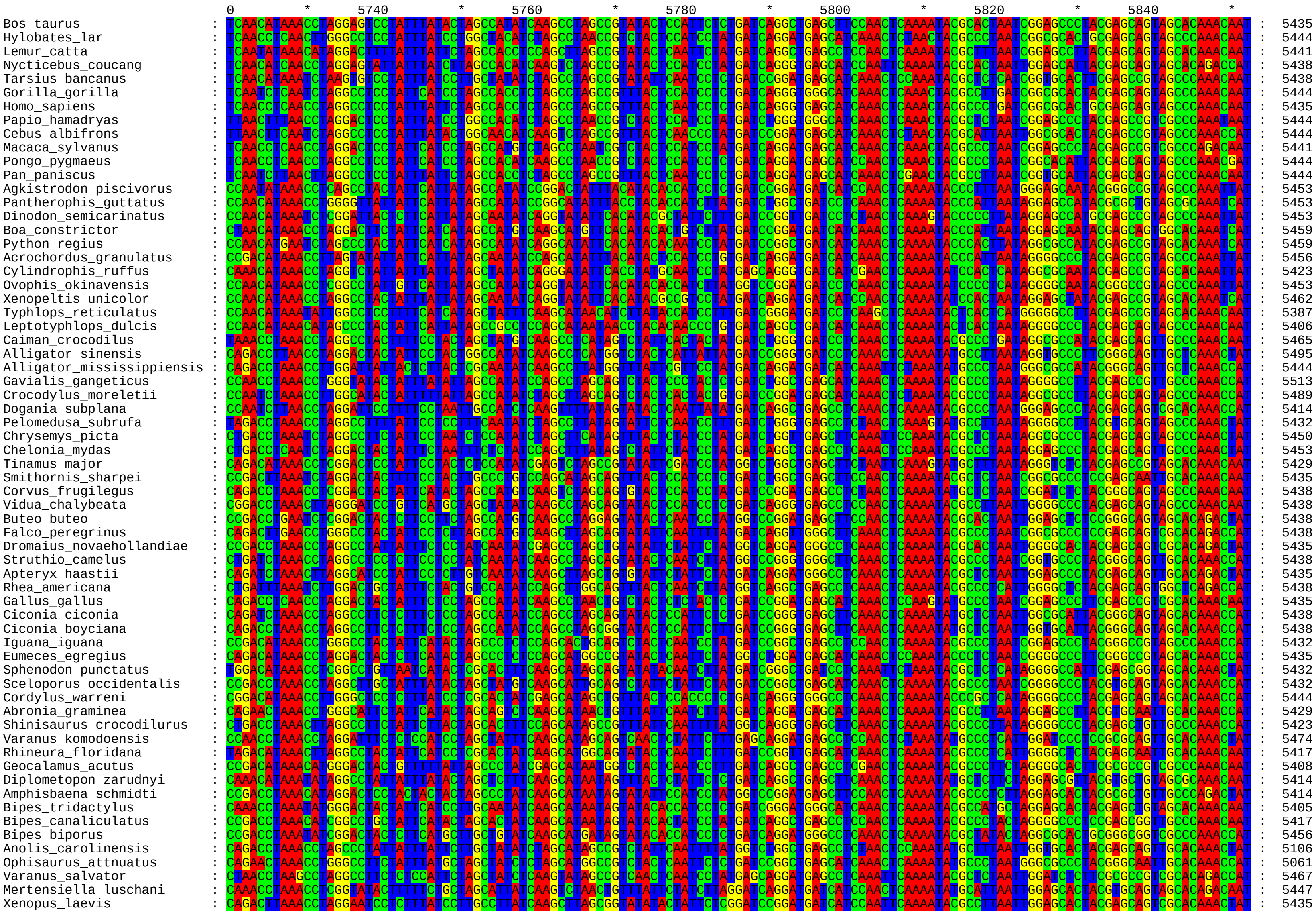

0 * 5740 * 5760 * 5780 * 5800 * 5820 * 5840 *
Bos_taurus : 5435
Hylobates_lar : 5444
Lemur_catta : 5441
Nycticebus_coucang : 5438
Tarsius_bancanus : 5438
Gorilla_gorilla : 5444
Homo_sapiens : 5435
Papio_hamadryas : 5444
Cebus_albifrons : 5444
Macaca_sylvanus : 5441
Pongo_pygmaeus : 5444
Pan_paniscus : 5444
Agkistrodon_piscivorus : 5453
Pantherophis_guttatus : 5453
Dinodon_semicarinatus : 5453
Boa_constrictor : 5459
Python_regius : 5459
Acrochordus_granulatus : 5456
Cylindrophis_ruffus : 5423
Ovophis_okinavensis : 5453
Xenopeltis_unicolor : 5462
Typhlops_reticulatus : 5387
Leptotyphlops_dulcis : 5406
Caiman_crocodilus : 5465
Alligator_sinensis : 5495
Alligator_mississippiensis : 5444
Gavialis_gangeticus : 5513
Crocodylus_moreletii : 5489
Dogania_subplana : 5414
Pelomedusa_subrufa : 5432
Chrysemys_picta : 5450
Chelonia_mydas : 5453
Tinamus_major : 5429
Smithornis_sharpei : 5435
Corvus_frugilegus : 5438
Vidua_chalybeata : 5438
Buteo_buteo : 5438
Falco_peregrinus : 5438
Dromaius_novaehollandiae : 5435
Struthio_camelus : 5438
Apteryx_haastii : 5435
Rhea_americana : 5438
Gallus_gallus : 5435
Ciconia_ciconia : 5438
Ciconia_boyciana : 5438
Iguana_iguana : 5432
Eumeces_egregius : 5435
Sphenodon_punctatus : 5432
Sceloporus_occidentalis : 5432
Cordylus_warreni : 5444
Abronia_graminea : 5429
Shinisaurus_crocodilurus : 5423
Varanus_komodoensis : 5474
Rhineura_floridana : 5417
Geocalamus_acutus : 5408
Diplometopon_zarudnyi : 5414
Amphisbaena_schmidti : 5414
Bipes_tridactylus : 5405
Bipes_canaliculatus : 5417
Bipes_biporus : 5456
Anolis_carolinensis : 5106
Ophisaurus_attnuatus : 5061
Varanus_salvator : 5467
Mertensiella_luschani : 5447
Xenopus_laevis : 5435

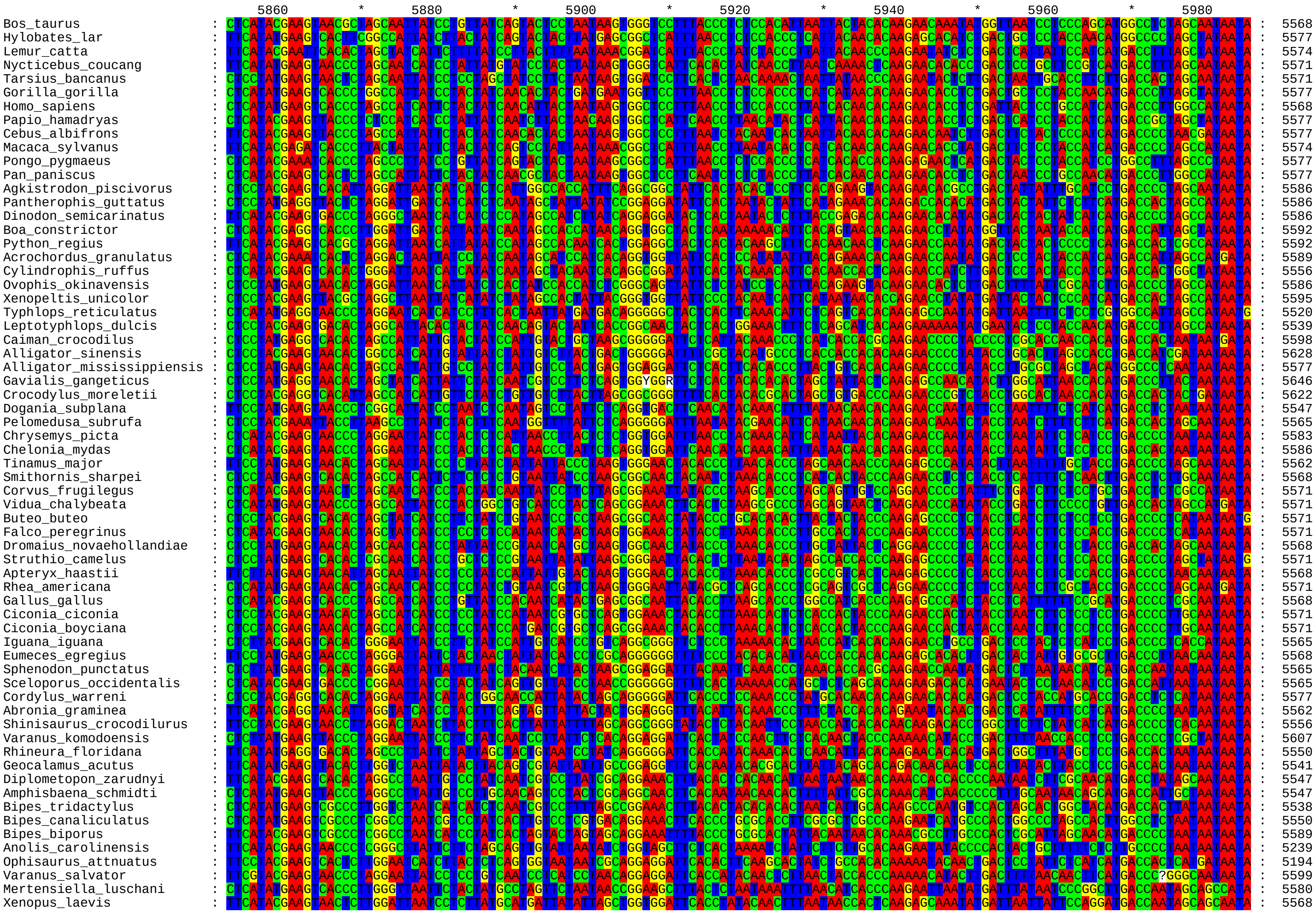
5860 * 5880 * 5900 * 5920 * 5940 * 5960 * 5980
Bos_taurus : 5568
Hylobates_lar : 5577
Lemur_catta : 5574
Nycticebus_coucang : 5571
Tarsius_bancanus : 5571
Gorilla_gorilla : 5577
Homo_sapiens : 5568
Papio_hamadryas : 5577
Cebus_albifrons : 5577
Macaca_sylvanus : 5574
Pongo_pygmaeus : 5577
Pan_paniscus : 5577
Agkistrodon_piscivorus : 5586
Pantherophis_guttatus : 5586
Dinodon_semicarinatus : 5586
Boa_constrictor : 5592
Python_regius : 5592
Acrochordus_granulatus : 5589
Cylindrophis_ruffus : 5556
Ovophis_okinavensis : 5586
Xenopeltis_unicolor : 5595
Typhlops_reticulatus : 5520
Leptotyphlops_dulcis : 5539
Caiman_crocodilus : 5598
Alligator_sinensis : 5628
Alligator_mississippiensis : 5577
Gavialis_gangeticus : 5646
Crocodylus_moreletii : 5622
Dogania_subplana : 5547
Pelomedusa_subrufa : 5565
Chrysemys_picta : 5583
Chelonia_mydas : 5586
Tinamus_major : 5562
Smithornis_sharpei : 5568
Corvus_frugilegus : 5571
Vidua_chalybeata : 5571
Buteo_buteo : 5571
Falco_peregrinus : 5571
Dromaius_novaehollandiae : 5568
Struthio_camelus : 5571
Apteryx_haastii : 5568
Rhea_americana : 5571
Gallus_gallus : 5568
Ciconia_ciconia : 5571
Ciconia_boyciana : 5571
Iguana_iguana : 5565
Eumeces_egregius : 5568
Sphenodon_punctatus : 5565
Sceloporus_occidentalis : 5565
Cordylus_warreni : 5577
Abronia_graminea : 5562
Shinisaurus_crocodilurus : 5556
Varanus_komodoensis : 5607
Rhineura_floridana : 5550
Geocalamus_acutus : 5541
Diplometopon_zarudnyi : 5547
Amphisbaena_schmidti : 5547
Bipes_tridactylus : 5538
Bipes_canaliculatus : 5550
Bipes_biporus : 5589
Anolis_carolinensis : 5239
Ophisaurus_attnuatus : 5194
Varanus_salvator : 5599
Mertensiella_luschani : 5580
Xenopus_laevis : 5568

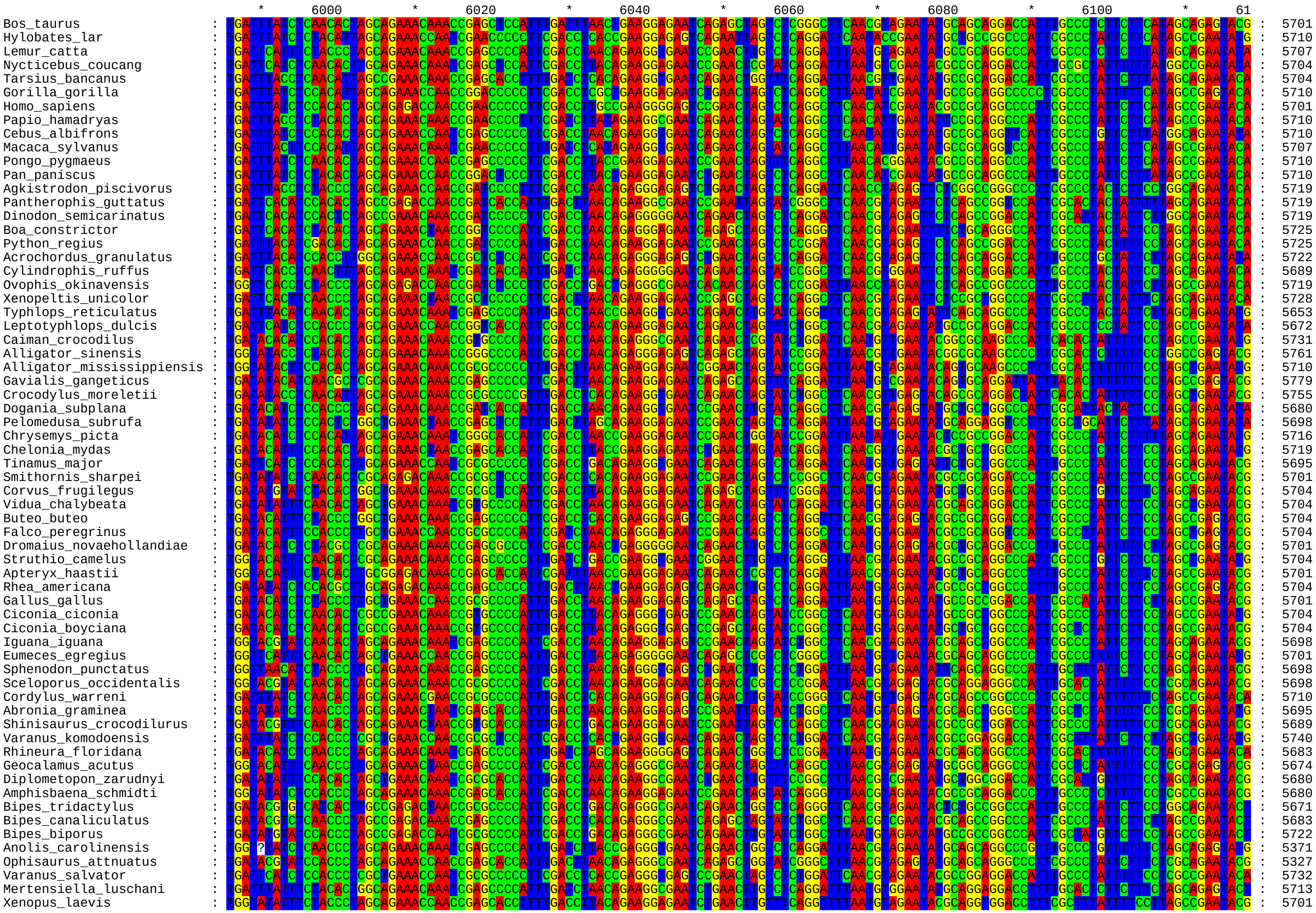
* 6000 * 6020 * 6040 * 6060 * 6080 * 6100 * 61
Bos_taurus : 5701
Hylobates_lar : 5710
Lemur_catta : 5707
Nycticebus_coucang : 5704
Tarsius_bancanus : 5704
Gorilla_gorilla : 5710
Homo_sapiens : 5701
Papio_hamadryas : 5710
Cebus_albifrons : 5710
Macaca_sylvanus : 5707
Pongo_pygmaeus : 5710
Pan_paniscus : 5710
Agkistrodon_piscivorus : 5719
Pantherophis_guttatus : 5719
Dinodon_semicarinatus : 5719
Boa_constrictor : 5725
Python_regius : 5725
Acrochordus_granulatus : 5722
Cylindrophis_ruffus : 5689
Ovophis_okinavensis : 5719
Xenopeltis_unicolor : 5728
Typhlops_reticulatus : 5653
Leptotyphlops_dulcis : 5672
Caiman_crocodilus : 5731
Alligator_sinensis : 5761
Alligator_mississippiensis : 5710
Gavialis_gangeticus : 5779
Crocodylus_moreletii : 5755
Dogania_subplana : 5680
Pelomedusa_subrufa : 5698
Chrysemys_picta : 5716
Chelonia_mydas : 5719
Tinamus_major : 5695
Smithornis_sharpei : 5701
Corvus_frugilegus : 5704
Vidua_chalybeata : 5704
Buteo_buteo : 5704
Falco_peregrinus : 5704
Dromaius_novaehollandiae : 5701
Struthio_camelus : 5704
Apteryx_haastii : 5701
Rhea_americana : 5704
Gallus_gallus : 5701
Ciconia_ciconia : 5704
Ciconia_boyciana : 5704
Iguana_iguana : 5698
Eumeces_egregius : 5701
Sphenodon_punctatus : 5698
Sceloporus_occidentalis : 5698
Cordylus_warreni : 5710
Abronia_graminea : 5695
Shinisaurus_crocodilurus : 5689
Varanus_komodoensis : 5740
Rhineura_floridana : 5683
Geocalamus_acutus : 5674
Diplometopon_zarudnyi : 5680
Amphisbaena_schmidti : 5680
Bipes_tridactylus : 5671
Bipes_canaliculatus : 5683
Bipes_biporus : 5722
Anolis_carolinensis : 5371
Ophisaurus_attnuatus : 5327
Varanus_salvator : 5732
Mertensiella_luschani : 5713
Xenopus_laevis : 5701

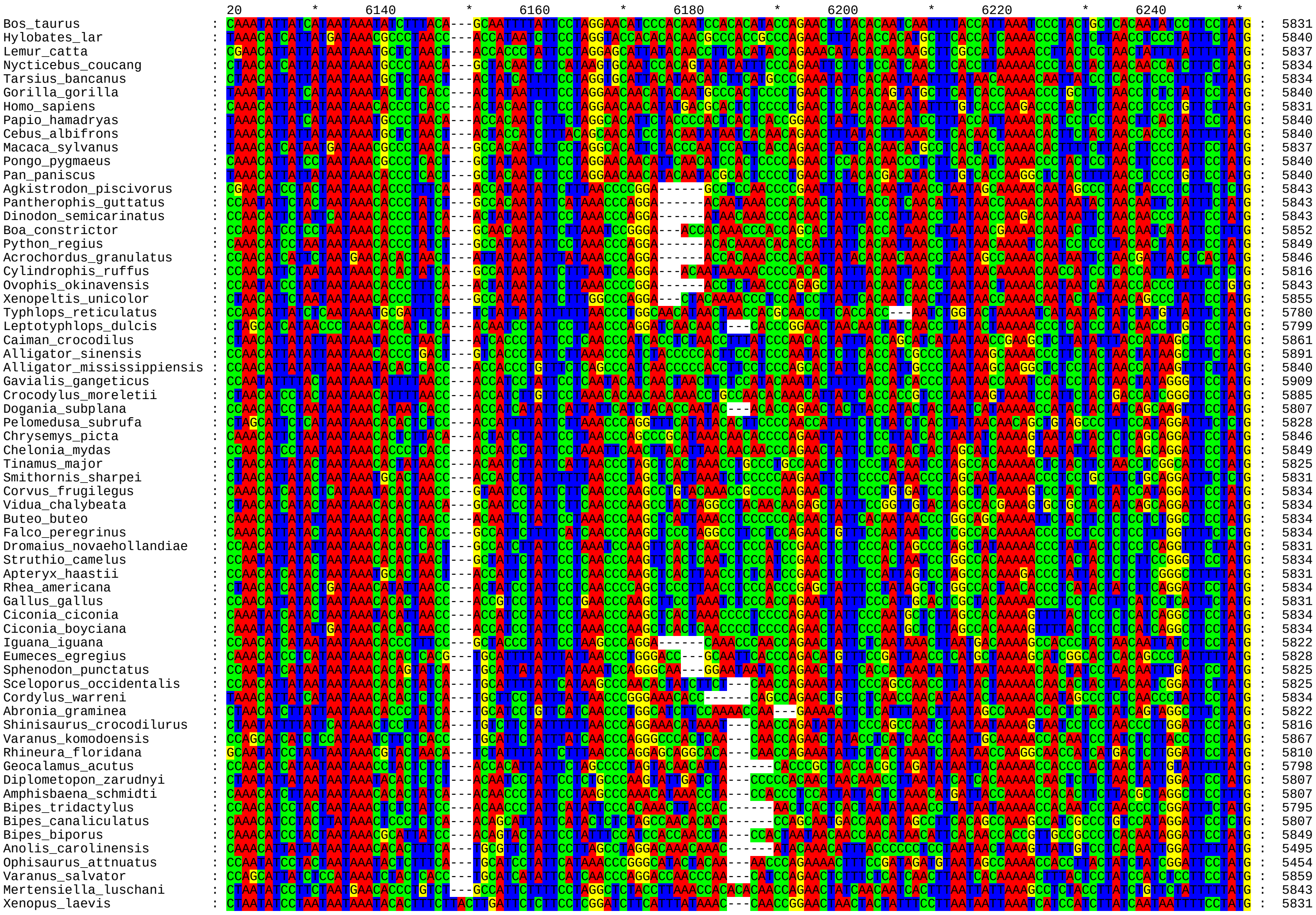
20 * 6140 * 6160 * 6180 * 6200 * 6220 * 6240 *
Bos_taurus : 5831
Hylobates_lar : 5840
Lemur_catta : 5837
Nycticebus_coucang : 5834
Tarsius_bancanus : 5834
Gorilla_gorilla : 5840
Homo_sapiens : 5831
Papio_hamadryas : 5840
Cebus_albifrons : 5840
Macaca_sylvanus : 5837
Pongo_pygmaeus : 5840
Pan_paniscus : 5840
Agkistrodon_piscivorus : 5843
Pantherophis_guttatus : 5843
Dinodon_semicarinatus : 5843
Boa_constrictor : 5852
Python_regius : 5849
Acrochordus_granulatus : 5846
Cylindrophis_ruffus : 5816
Ovophis_okinavensis : 5843
Xenopeltis_unicolor : 5855
Typhlops_reticulatus : 5780
Leptotyphlops_dulcis : 5799
Caiman_crocodilus : 5861
Alligator_sinensis : 5891
Alligator_mississippiensis : 5840
Gavialis_gangeticus : 5909
Crocodylus_moreletii : 5885
Dogania_subplana : 5807
Pelomedusa_subrufa : 5828
Chrysemys_picta : 5846
Chelonia_mydas : 5849
Tinamus_major : 5825
Smithornis_sharpei : 5831
Corvus_frugilegus : 5834
Vidua_chalybeata : 5834
Buteo_buteo : 5834
Falco_peregrinus : 5834
Dromaius_novaehollandiae : 5831
Struthio_camelus : 5834
Apteryx_haastii : 5831
Rhea_americana : 5834
Gallus_gallus : 5831
Ciconia_ciconia : 5834
Ciconia_boyciana : 5834
Iguana_iguana : 5822
Eumeces_egregius : 5828
Sphenodon_punctatus : 5825
Sceloporus_occidentalis : 5825
Cordylus_warreni : 5834
Abronia_graminea : 5822
Shinisaurus_crocodilurus : 5816
Varanus_komodoensis : 5867
Rhineura_floridana : 5810
Geocalamus_acutus : 5798
Diplometopon_zarudnyi : 5807
Amphisbaena_schmidti : 5807
Bipes_tridactylus : 5795
Bipes_canaliculatus : 5807
Bipes_biporus : 5849
Anolis_carolinensis : 5495
Ophisaurus_attnuatus : 5454
Varanus_salvator : 5859
Mertensiella_luschani : 5843
Xenopus_laevis : 5831

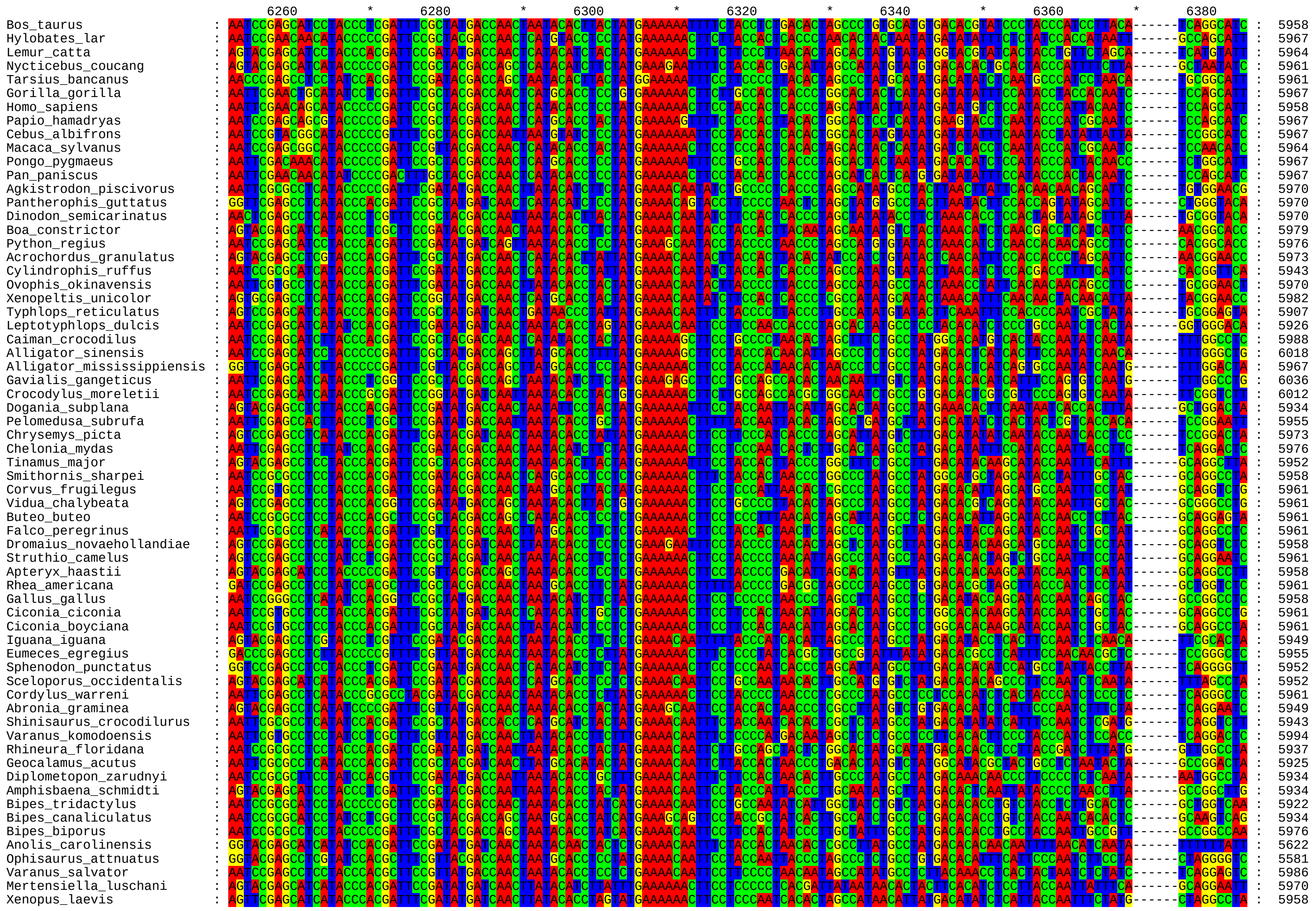
6260
6280
6300
6320
6340
6360
6380
Bos_taurus 5958
Hylobates_lar 5967
Lemur_catta 5964
Nycticebus_coucang 5961
Tarsius_bancanus 5961
Gorilla_gorilla 5967
Homo_sapiens 5958
Papio_hamadryas 5967
Cebus_albifrons 5967
Macaca_sylvanus 5964
Pongo_pygmaeus 5967
Pan_paniscus 5967
Agkistrodon_piscivorus 5970
Pantherophis_guttatus 5970
Dinodon_semicarinatus 5970
Boa_constrictor 5979
Python_regius 5976
Acrochordus_granulatus 5973
Cylindrophis_ruffus 5943
Ovophis_okinavensis 5970
Xenopeltis_unicolor 5982
Typhlops_reticulatus 5907
Leptotyphlops_dulcis 5926
Caiman_crocodilus 5988
Alligator_sinensis 6018
Alligator_mississippiensis 5967
Gavialis_gangeticus 6036
Crocodylus_moreletii 6012
Dogania_subplana 5934
Pelomedusa_subrufa 5955
Chrysemys_picta 5973
Chelonia_mydas 5976
Tinamus_major 5952
Smithornis_sharpei 5958
Corvus_frugilegus 5961
Vidua_chalybeata 5961
Buteo_buteo 5961
Falco_peregrinus 5961
Dromaius_novaehollandiae 5958
Struthio_camelus 5961
Apteryx_haastii 5958
Rhea_americana 5961
Gallus_gallus 5958
Ciconia_ciconia 5961
Ciconia_boyciana 5961
Iguana_iguana 5949
Eumeces_egregius 5955
Sphenodon_punctatus 5952
Sceloporus_occidentalis 5952
Cordylus_warreni 5961
Abronia_graminea 5949
Shinisaurus_crocodilurus 5943
Varanus_komodoensis 5994
Rhineura_floridana 5937
Geocalamus_acutus 5925
Diplometopon_zarudnyi 5934
Amphisbaena_schmidti 5934
Bipes_tridactylus 5922
Bipes_canaliculatus 5934
Bipes_biporus 5976
Anolis_carolinensis 5622
Ophisaurus_attenuatus 5581
Varanus_salvator 5986
Mertensiella_luschani 5970
Xenopus_laevis 5958

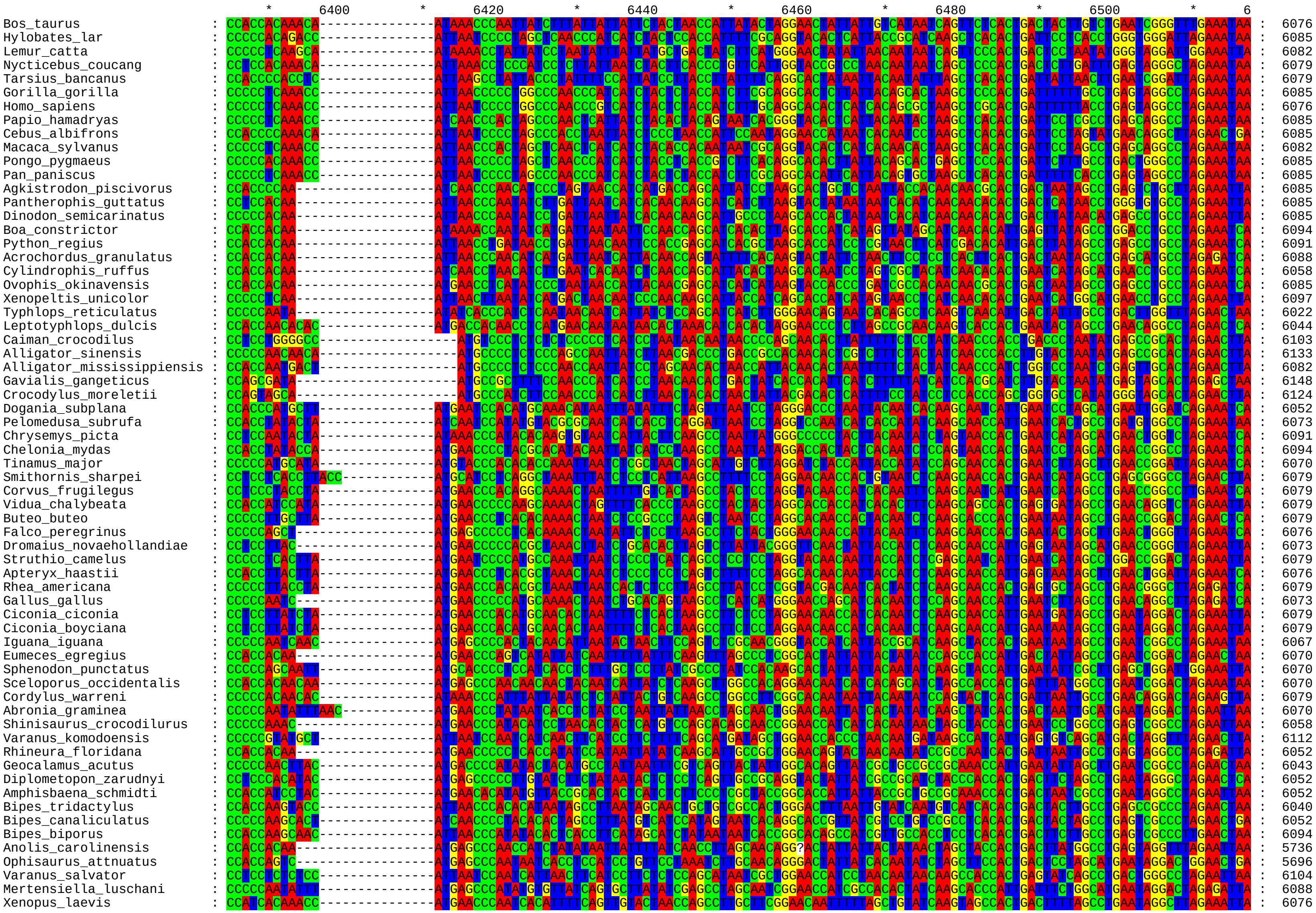

6400
6420
6440
6460
6480
6500
Bos_taurus 6076
Hylobates_lar 6085
Lemur_catta 6082
Nycticebus_coucang 6079
Tarsius_bancanus 6079
Gorilla_gorilla 6085
Homo_sapiens 6076
Papio_hamadryas 6085
Cebus_albifrons 6085
Macaca_sylvanus 6082
Pongo_pygmaeus 6085
Pan_paniscus 6085
Agkistrodon_piscivorus 6085
Pantherophis_guttatus 6085
Dinodon_semicarinatus 6085
Boa_constrictor 6094
Python_regius 6091
Acrochordus_granulatus 6088
Cylindrophis_ruffus 6058
Ovophis_okinavensis 6085
Xenopeltis_unicolor 6097
Typhlops_reticulatus 6022
Leptotyphlops_dulcis 6044
Caiman_crocodilus 6103
Alligator_sinensis 6133
Alligator_mississippiensis 6082
Gavialis_gangeticus 6148
Crocodylus_moreletii 6124
Dogania_subplana 6052
Pelomedusa_subrufa 6073
Chrysemys_picta 6091
Chelonia_mydas 6094
Tinamus_major 6070
Smithornis_sharpei 6079
Corvus_frugilegus 6079
Vidua_chalybeata 6079
Buteo_buteo 6079
Falco_peregrinus 6076
Dromaius_novaehollandiae 6073
Struthio_camelus 6079
Apteryx_haastii 6076
Rhea_americana 6079
Gallus_gallus 6073
Ciconia_ciconia 6079
Ciconia_boyciana 6079
Iguana_iguana 6067
Eumeces_egregius 6070
Sphenodon_punctatus 6070
Sceloporus_occidentalis 6070
Cordylus_warreni 6079
Abronia_graminea 6070
Shinisaurus_crocodilurus 6058
Varanus_komodoensis 6112
Rhineura_floridana 6052
Geocalamus_acutus 6043
Diplometopon_zarudnyi 6052
Amphisbaena_schmidti 6052
Bipes_tridactylus 6040
Bipes_canaliculatus 6052
Bipes_biporus 6094
Anolis_carolinensis 5736
Ophisaurus_attnuatus 5696
Varanus_salvator 6104
Mertensiella_luschani 6088
Xenopus_laevis 6076

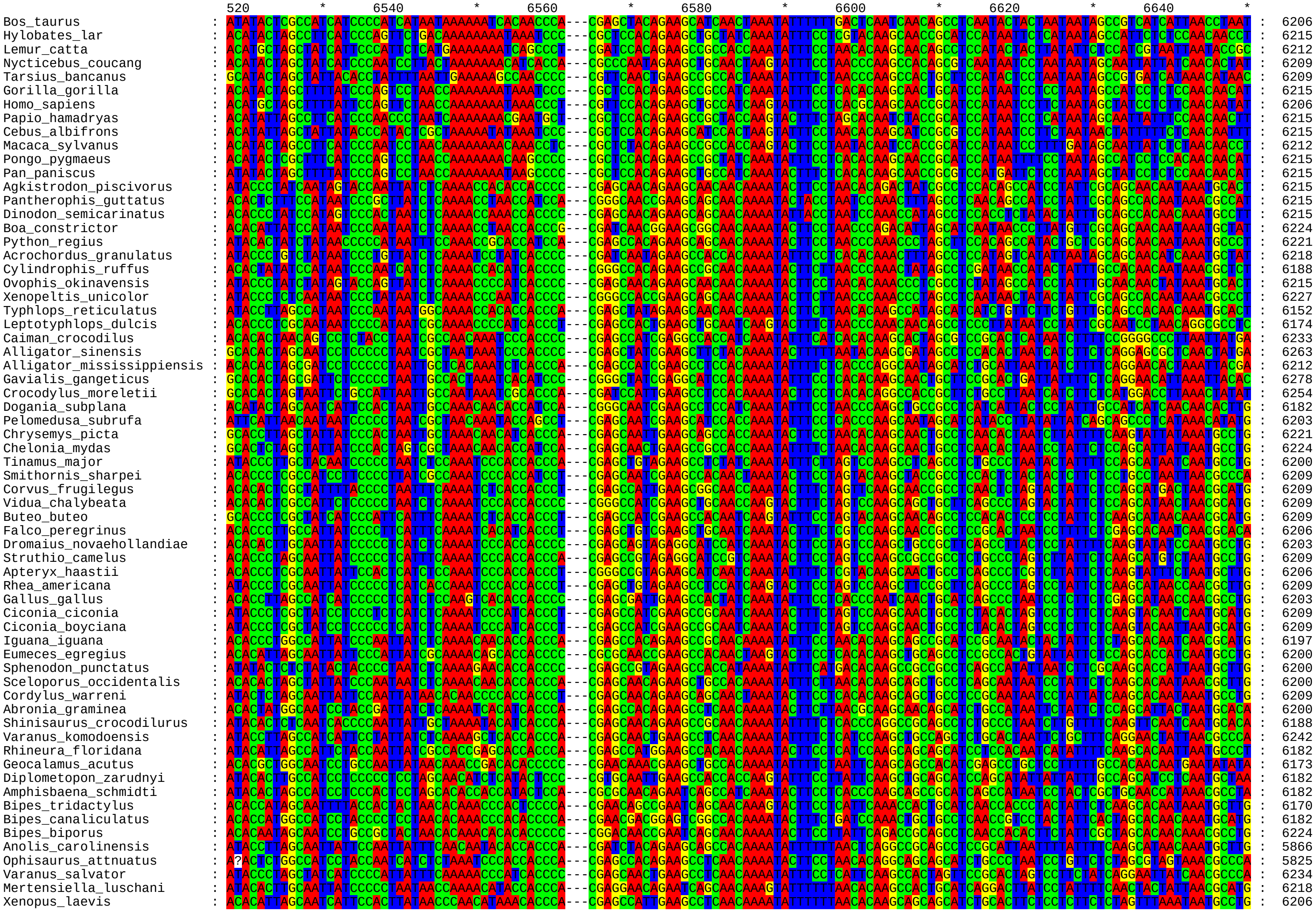
520 * 6540 * 6560 * 6580 * 6600 * 6620 * 6640 *
Bos_taurus 6206
Hylobates_lar 6215
Lemur_catta 6212
Nycticebus_coucang 6209
Tarsius_bancanus 6209
Gorilla_gorilla 6215
Homo_sapiens 6206
Papio_hamadryas 6215
Cebus_albifrons 6215
Macaca_sylvanus 6212
Pongo_pygmaeus 6215
Pan_paniscus 6215
Agkistrodon_piscivorus 6215
Pantherophis_guttatus 6215
Dinodon_semicarinatus 6215
Boa_constrictor 6224
Python_regius 6221
Acrochordus_granulatus 6218
Cylindrophis_ruffus 6188
Ovophis_okinavensis 6215
Xenopeltis_unicolor 6227
Typhlops_reticulatus 6152
Leptotyphlops_dulcis 6174
Caiman_crocodilus 6233
Alligator_sinensis 6263
Alligator_mississippiensis 6212
Gavialis_gangeticus 6278
Crocodylus_moreletii 6254
Dogania_subplana 6182
Pelomedusa_subrufa 6203
Chrysemys_picta 6221
Chelonia_mydas 6224
Tinamus_major 6200
Smithornis_sharpei 6209
Corvus_frugilegus 6209
Vidua_chalybeata 6209
Buteo_buteo 6209
Falco_peregrinus 6206
Dromaius_novaehollandiae 6203
Struthio_camelus 6209
Apteryx_haastii 6206
Rhea_americana 6209
Gallus_gallus 6203
Ciconia_ciconia 6209
Ciconia_boyciana 6209
Iguana_iguana 6197
Eumeces_egregius 6200
Sphenodon_punctatus 6200
Sceloporus_occidentalis 6200
Cordylus_warreni 6209
Abronia_graminea 6200
Shinisaurus_crocodilurus 6188
Varanus_komodoensis 6242
Rhineura_floridana 6182
Geocalamus_acutus 6173
Diplometopon_zarudnyi 6182
Amphisbaena_schmidti 6182
Bipes_tridactylus 6170
Bipes_canaliculatus 6182
Bipes_biporus 6224
Anolis_carolinensis 5866
Ophisaurus_attnuatus 5825
Varanus_salvator 6234
Mertensiella_luschani 6218
Xenopus_laevis 6206

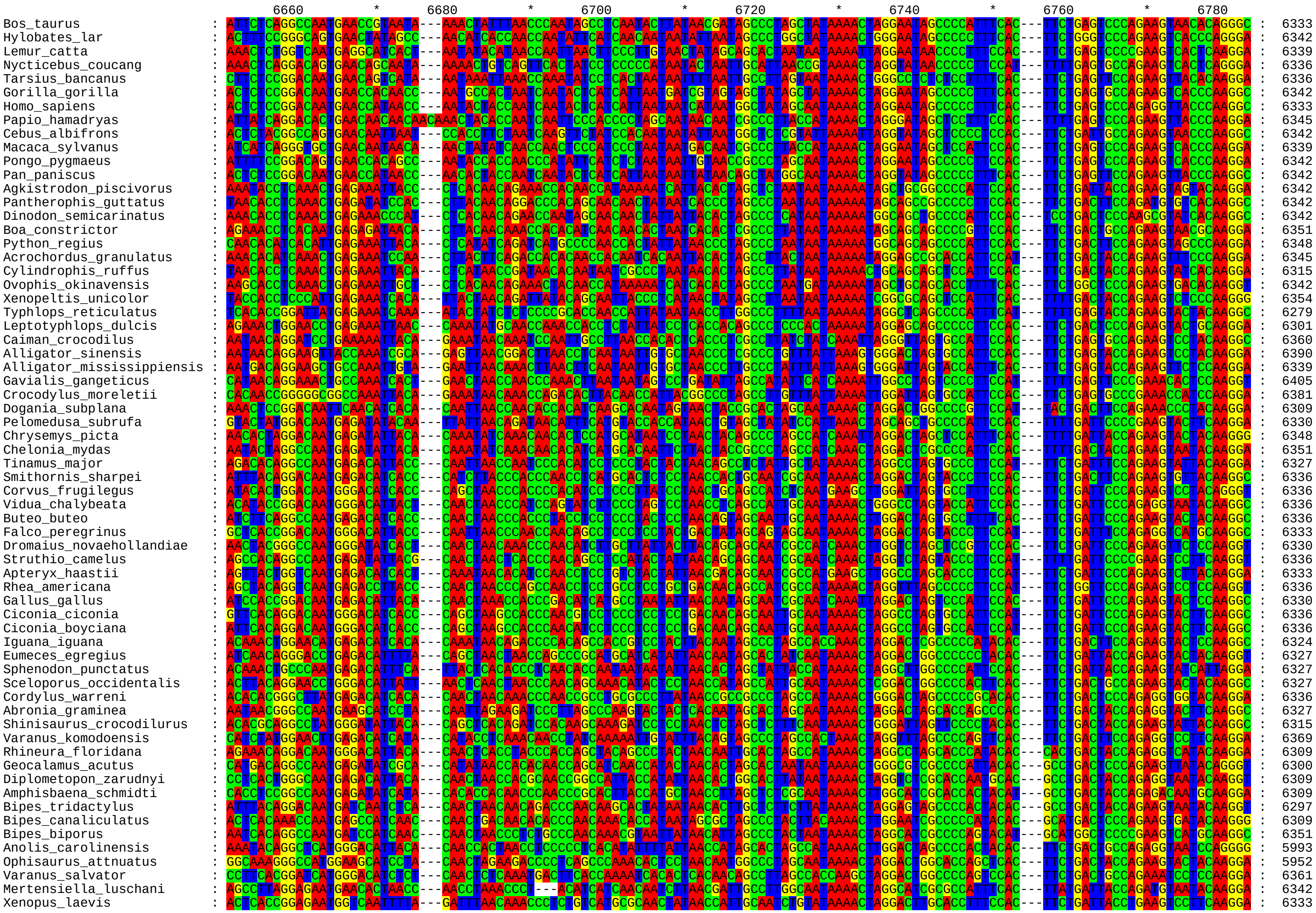
6660
6680
6700
6720
6740
6760
6780
Bos_taurus 6333
Hylobates_lar 6342
Lemur_catta 6339
Nycticebus_coucang 6336
Tarsius_bancanus 6336
Gorilla_gorilla 6342
Homo_sapiens 6333
Papio_hamadryas 6345
Cebus_albifrons 6342
Macaca_sylvanus 6339
Pongo_pygmaeus 6342
Pan_paniscus 6342
Agkistrodon_piscivorus 6342
Pantherophis_guttatus 6342
Dinodon_semicarinatus 6342
Boa_constrictor 6351
Python_regius 6348
Acrochordus_granulatus 6345
Cylindrophis_ruffus 6315
Ovophis_okinavensis 6342
Xenopeltis_unicolor 6354
Typhlops_reticulatus 6279
Leptotyphlops_dulcis 6301
Caiman_crocodilus 6360
Alligator_sinensis 6390
Alligator_mississippiensis 6339
Gavialis_gangeticus 6405
Crocodylus_moreletii 6381
Dogania_subplana 6309
Pelomedusa_subrufa 6330
Chrysemys_picta 6348
Chelonia_mydas 6351
Tinamus_major 6327
Smithornis_sharpei 6336
Corvus_frugilegus 6336
Vidua_chalybeata 6336
Buteo_buteo 6336
Falco_peregrinus 6333
Dromaius_novaehollandiae 6330
Struthio_camelus 6336
Apteryx_haastii 6333
Rhea_americana 6336
Gallus_gallus 6330
Ciconia_ciconia 6336
Ciconia_boyciana 6336
Iguana_iguana 6324
Eumeces_egregius 6327
Sphenodon_punctatus 6327
Sceloporus_occidentalis 6327
Cordylus_warreni 6336
Abronia_graminea 6327
Shinisaurus_crocodilurus 6315
Varanus_komodoensis 6369
Rhineura_floridana 6309
Geocalamus_acutus 6300
Diplometopon_zarudnyi 6309
Amphisbaena_schmidti 6309
Bipes_tridactylus 6297
Bipes_canaliculatus 6309
Bipes_biporus 6351
Anolis_carolinensis 5993
Ophisaurus_attnuatus 5952
Varanus_salvator 6361
Mertensiella_luschani 6342
Xenopus_laevis 6333

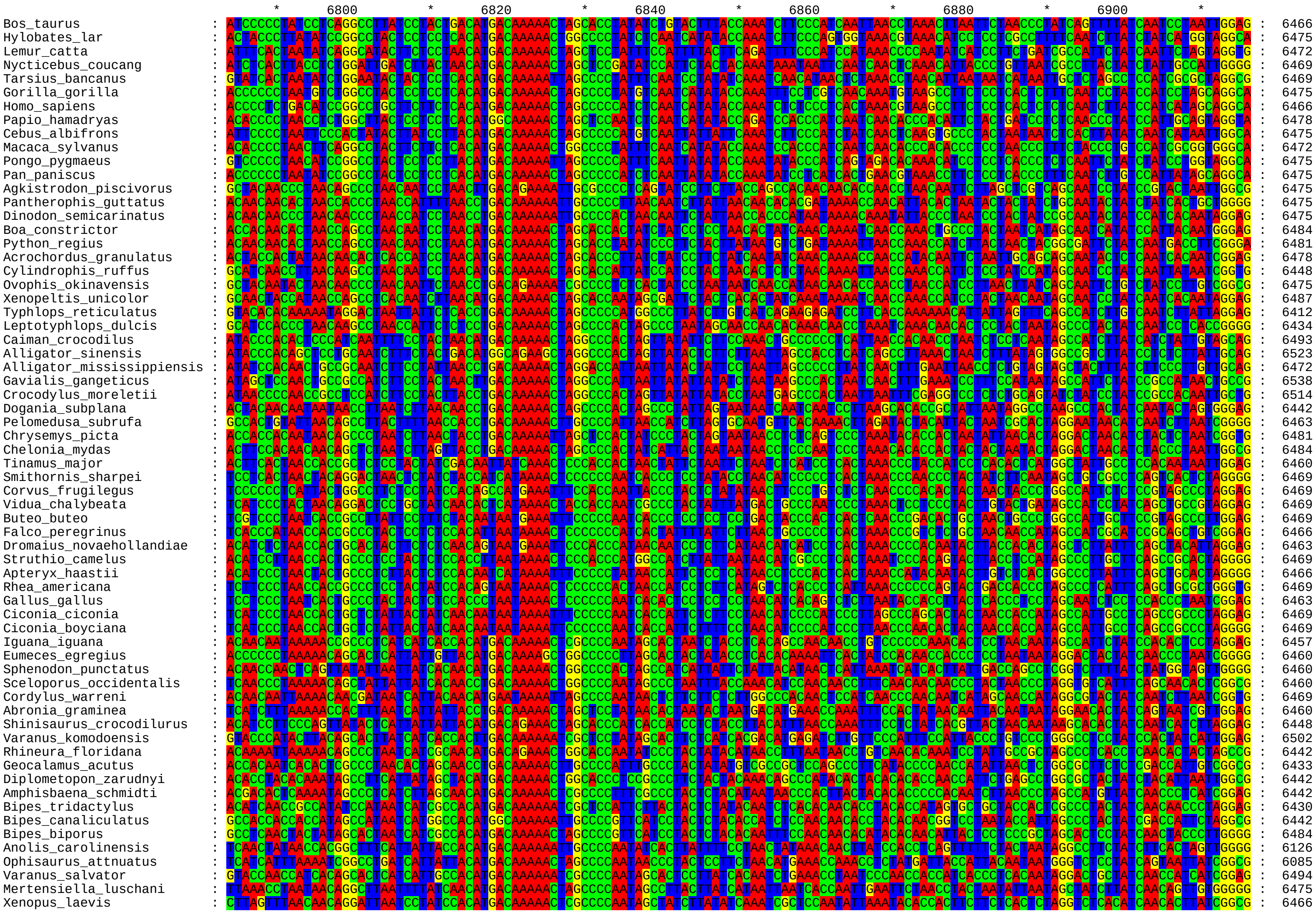

6800
6820
6840
6860
6880
6900
Bos_taurus : 6466
Hylobates_lar : 6475
Lemur_catta : 6472
Nycticebus_coucang : 6469
Tarsius_bancanus : 6469
Gorilla_gorilla : 6475
Homo_sapiens : 6466
Papio_hamadryas : 6478
Cebus_albifrons : 6475
Macaca_sylvanus : 6472
Pongo_pygmaeus : 6475
Pan_paniscus : 6475
Agkistrodon_piscivorus : 6475
Pantherophis_guttatus : 6475
Dinodon_semicarinatus : 6475
Boa_constrictor : 6484
Python_regius : 6481
Acrochordus_granulatus : 6478
Cylindrophis_ruffus : 6448
Ovophis_okinavensis : 6475
Xenopeltis_unicolor : 6487
Typhlops_reticulatus : 6412
Leptotyphlops_dulcis : 6434
Caiman_crocodilus : 6493
Alligator_sinensis : 6523
Alligator_mississippiensis : 6472
Gavialis_gangeticus : 6538
Crocodylus_moreletii : 6514
Dogania_subplana : 6442
Pelomedusa_subrufa : 6463
Chrysemys_picta : 6481
Chelonia_mydas : 6484
Tinamus_major : 6460
Smithornis_sharpei : 6469
Corvus_frugilegus : 6469
Vidua_chalybeata : 6469
Buteo_buteo : 6469
Falco_peregrinus : 6466
Dromaius_novaehollandiae : 6463
Struthio_camelus : 6469
Apteryx_haastii : 6466
Rhea_americana : 6469
Gallus_gallus : 6463
Ciconia_ciconia : 6469
Ciconia_boyciana : 6469
Iguana_iguana : 6457
Eumeces_egregius : 6460
Sphenodon_punctatus : 6460
Sceloporus_occidentalis : 6460
Cordylus_warreni : 6469
Abronia_graminea : 6460
Shinisaurus_crocodilurus : 6448
Varanus_komodoensis : 6502
Rhineura_floridana : 6442
Geocalamus_acutus : 6433
Diplometopon_zarudnyi : 6442
Amphisbaena_schmidti : 6442
Bipes_tridactylus : 6430
Bipes_canaliculatus : 6442
Bipes_biporus : 6484
Anolis_carolinensis : 6126
Ophisaurus_attnuatus : 6085
Varanus_salvator : 6494
Mertensiella_luschani : 6475
Xenopus_laevis : 6466

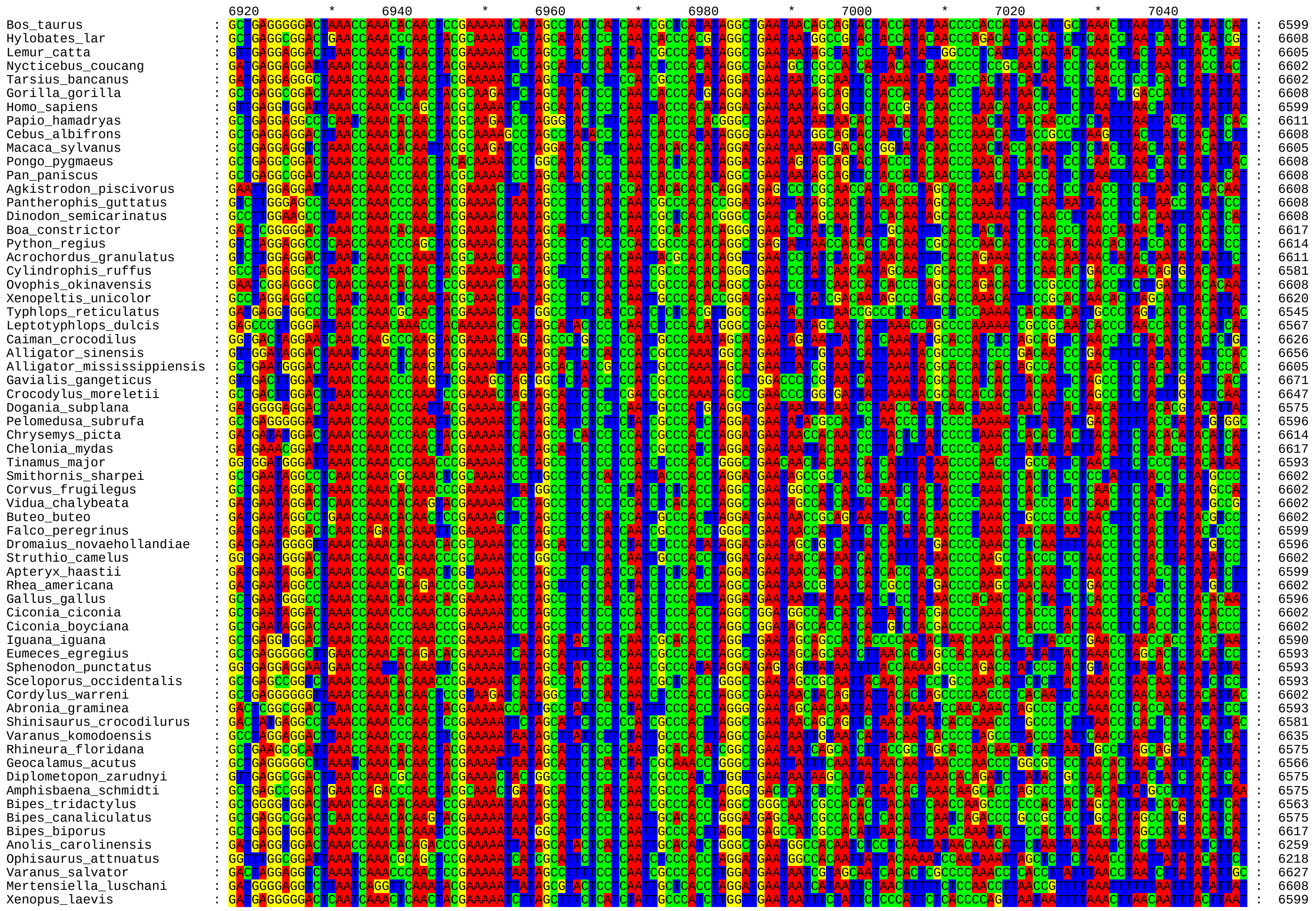

6920 * 6940 * 6960 * 6980 * 7000 * 7020 * 7040
Bos_taurus : 6599
Hylobates_lar : 6608
Lemur_catta : 6605
Nycticebus_coucang : 6602
Tarsius_bancanus : 6602
Gorilla_gorilla : 6608
Homo_sapiens : 6599
Papio_hamadryas : 6611
Cebus_albifrons : 6608
Macaca_sylvanus : 6605
Pongo_pygmaeus : 6608
Pan_paniscus : 6608
Agkistrodon_piscivorus : 6608
Pantherophis_guttatus : 6608
Dinodon_semicarinatus : 6608
Boa_constrictor : 6617
Python_regius : 6614
Acrochordus_granulatus : 6611
Cylindrophis_ruffus : 6581
Ovophis_okinavensis : 6608
Xenopeltis_unicolor : 6620
Typhlops_reticulatus : 6545
Leptotyphlops_dulcis : 6567
Caiman_crocodilus : 6626
Alligator_sinensis : 6656
Alligator_mississippiensis : 6605
Gavialis_gangeticus : 6671
Crocodylus_moreletii : 6647
Dogania_subplana : 6575
Pelomedusa_subrufa : 6596
Chrysemys_picta : 6614
Chelonia_mydas : 6617
Tinamus_major : 6593
Smithornis_sharpei : 6602
Corvus_frugilegus : 6602
Vidua_chalybeata : 6602
Buteo_buteo : 6602
Falco_peregrinus : 6599
Dromaius_novaehollandiae : 6596
Struthio_camelus : 6602
Apteryx_haastii : 6599
Rhea_americana : 6602
Gallus_gallus : 6596
Ciconia_ciconia : 6602
Ciconia_boyciana : 6602
Iguana_iguana : 6590
Eumeces_egregius : 6593
Sphenodon_punctatus : 6593
Sceloporus_occidentalis : 6593
Cordylus_warreni : 6602
Abronia_graminea : 6593
Shinisaurus_crocodilurus : 6581
Varanus_komodoensis : 6635
Rhineura_floridana : 6575
Geocalamus_acutus : 6566
Diplometopon_zarudnyi : 6575
Amphisbaena_schmidti : 6575
Bipes_tridactylus : 6563
Bipes_canaliculatus : 6575
Bipes_biporus : 6617
Anolis_carolinensis : 6259
Ophisaurus_attnuatus : 6218
Varanus_salvator : 6627
Mertensiella_luschani : 6608
Xenopus_laevis : 6599

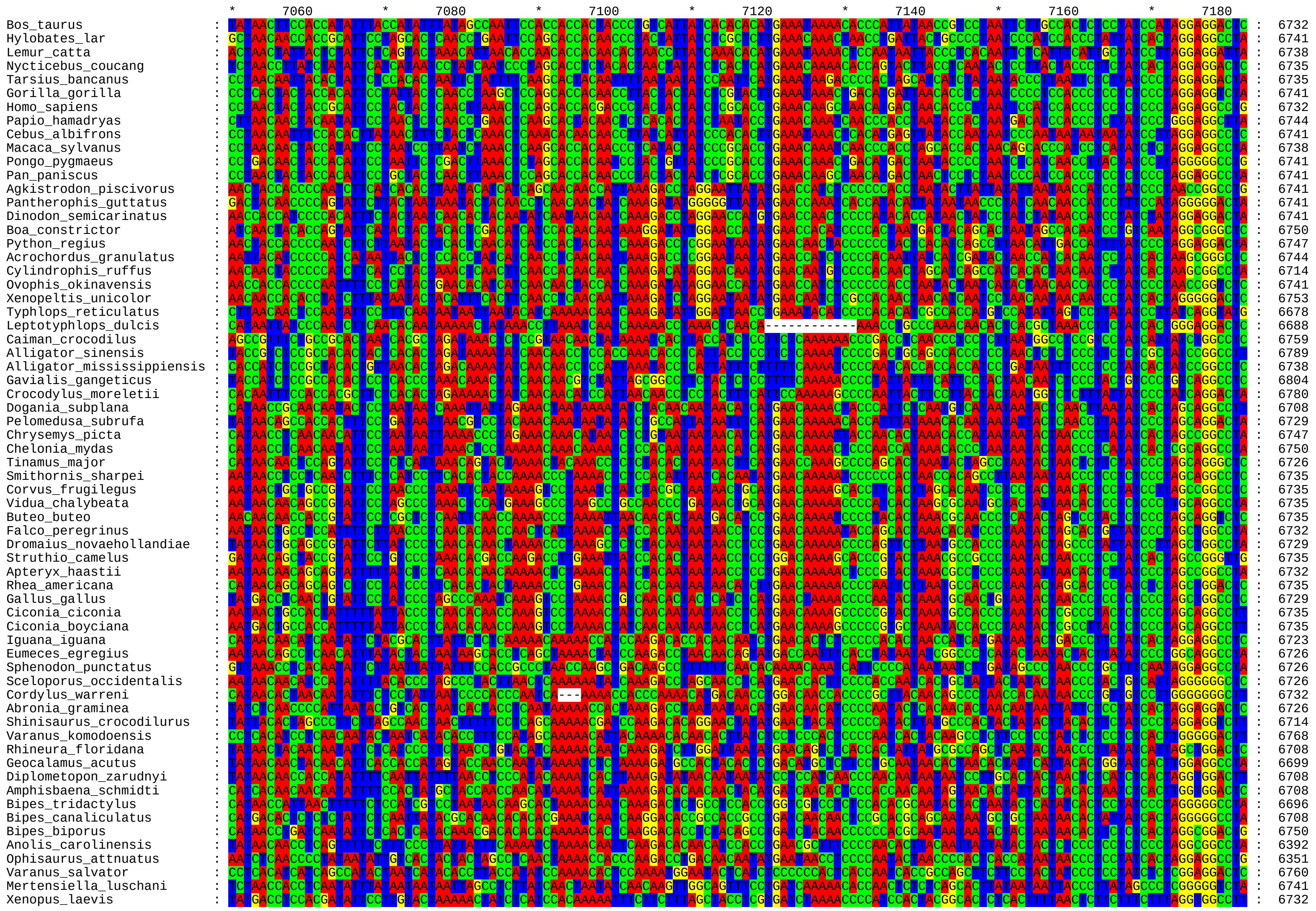
* 7060 * 7080 * 7100 * 7120 * 7140 * 7160 * 7180
Bos_taurus : 6732
Hylobates_lar : 6741
Lemur_catta : 6738
Nycticebus_coucang : 6735
Tarsius_bancanus : 6735
Gorilla_gorilla : 6741
Homo_sapiens : 6732
Papio_hamadryas : 6744
Cebus_albifrons : 6741
Macaca_sylvanus : 6738
Pongo_pygmaeus : 6741
Pan_paniscus : 6741
Agkistrodon_piscivorus : 6741
Pantherophis_guttatus : 6741
Dinodon_semicarinatus : 6741
Boa_constrictor : 6750
Python_regius : 6747
Acrochordus_granulatus : 6744
Cylindrophis_ruffus : 6714
Ovophis_okinavensis : 6741
Xenopeltis_unicolor : 6753
Typhlops_reticulatus : 6678
Leptotyphlops_dulcis : 6688
Caiman_crocodilus : 6759
Alligator_sinensis : 6789
Alligator_mississippiensis : 6738
Gavialis_gangeticus : 6804
Crocodylus_moreletii : 6780
Dogania_subplana : 6708
Pelomedusa_subrufa : 6729
Chrysemys_picta : 6747
Chelonia_mydas : 6750
Tinamus_major : 6726
Smithornis_sharpei : 6735
Corvus_frugilegus : 6735
Vidua_chalybeata : 6735
Buteo_buteo : 6735
Falco_peregrinus : 6732
Dromaius_novaehollandiae : 6729
Struthio_camelus : 6735
Apteryx_haastii : 6732
Rhea_americana : 6735
Gallus_gallus : 6729
Ciconia_ciconia : 6735
Ciconia_boyciana : 6735
Iguana_iguana : 6723
Eumeces_egregius : 6726
Sphenodon_punctatus : 6726
Sceloporus_occidentalis : 6726
Cordylus_warreni : 6732
Abronia_graminea : 6726
Shinisaurus_crocodilurus : 6714
Varanus_komodoensis : 6768
Rhineura_floridana : 6708
Geocalamus_acutus : 6699
Diplometopon_zarudnyi : 6708
Amphisbaena_schmidti : 6708
Bipes_tridactylus : 6696
Bipes_canaliculatus : 6708
Bipes_biporus : 6750
Anolis_carolinensis : 6392
Ophisaurus_attnuatus : 6351
Varanus_salvator : 6760
Mertensiella_luschani : 6741
Xenopus_laevis : 6732

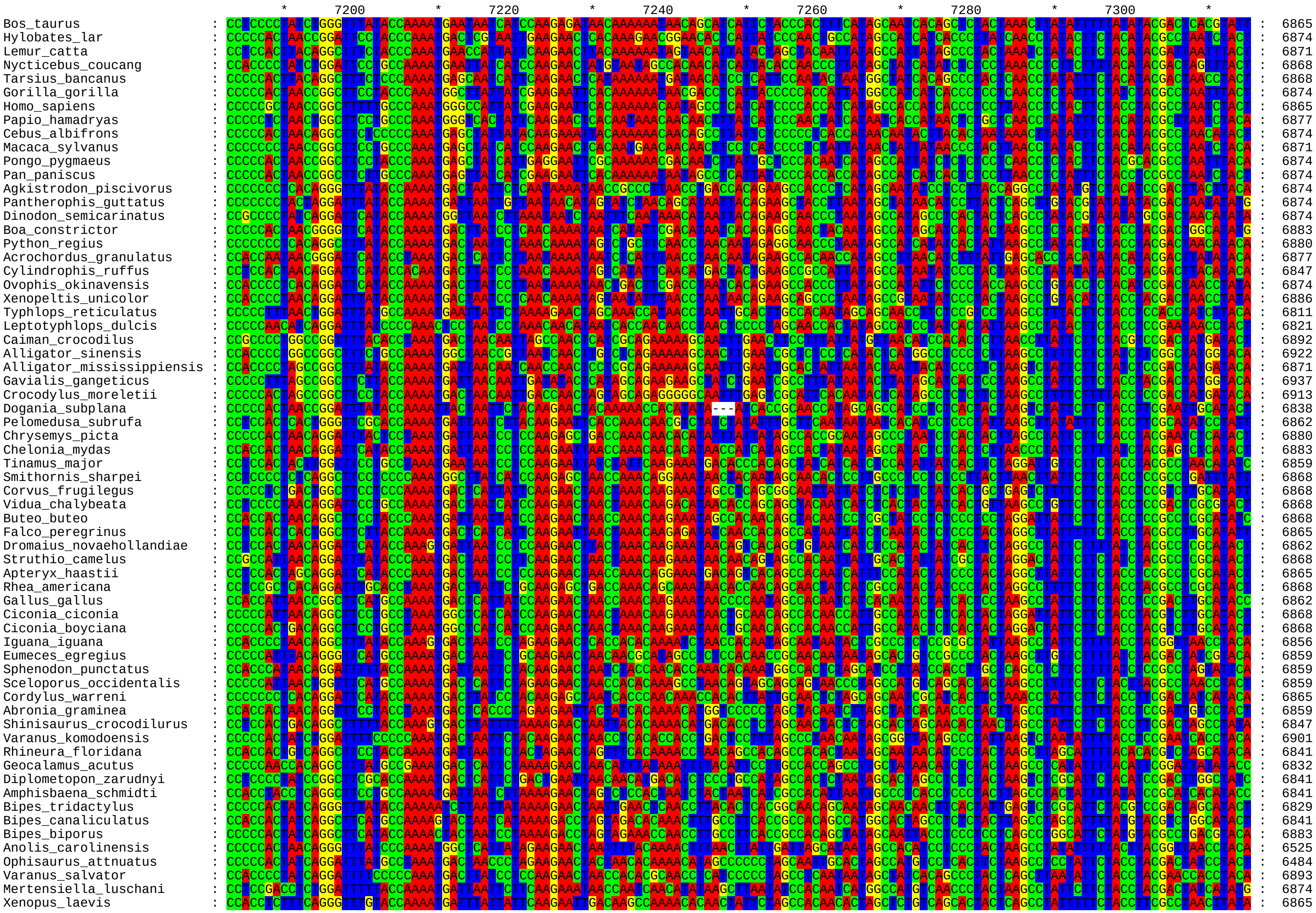
* 7200 * 7220 * 7240 * 7260 * 7280 * 7300 *
Bos_taurus : 6865
Hylobates_lar : 6874
Lemur_catta : 6871
Nycticebus_coucang : 6868
Tarsius_bancanus : 6868
Gorilla_gorilla : 6874
Homo_sapiens : 6865
Papio_hamadryas : 6877
Cebus_albifrons : 6874
Macaca_sylvanus : 6871
Pongo_pygmaeus : 6874
Pan_paniscus : 6874
Agkistrodon_piscivorus : 6874
Pantherophis_guttatus : 6874
Dinodon_semicarinatus : 6874
Boa_constrictor : 6883
Python_regius : 6880
Acrochordus_granulatus : 6877
Cylindrophis_ruffus : 6847
Ovophis_okinavensis : 6874
Xenopeltis_unicolor : 6886
Typhlops_reticulatus : 6811
Leptotyphlops_dulcis : 6821
Caiman_crocodilus : 6892
Alligator_sinensis : 6922
Alligator_mississippiensis : 6871
Gavialis_gangeticus : 6937
Crocodylus_moreletii : 6913
Dogania_subplana : 6838
Pelomedusa_subrufa : 6862
Chrysemys_picta : 6880
Chelonia_mydas : 6883
Tinamus_major : 6859
Smithornis_sharpei : 6868
Corvus_frugilegus : 6868
Vidua_chalybeata : 6868
Buteo_buteo : 6868
Falco_peregrinus : 6865
Dromaius_novaehollandiae : 6862
Struthio_camelus : 6868
Apteryx_haastii : 6865
Rhea_americana : 6868
Gallus_gallus : 6862
Ciconia_ciconia : 6868
Ciconia_boyciana : 6868
Iguana_iguana : 6856
Eumeces_egregius : 6859
Sphenodon_punctatus : 6859
Sceloporus_occidentalis : 6859
Cordylus_warreni : 6865
Abronia_graminea : 6859
Shinisaurus_crocodilurus : 6847
Varanus_komodoensis : 6901
Rhineura_floridana : 6841
Geocalamus_acutus : 6832
Diplometopon_zarudnyi : 6841
Amphisbaena_schmidti : 6841
Bipes_tridactylus : 6829
Bipes_canaliculatus : 6841
Bipes_biporus : 6883
Anolis_carolinensis : 6525
Ophisaurus_attnuatus : 6484
Varanus_salvator : 6893
Mertensiella_luschani : 6874
Xenopus_laevis : 6865

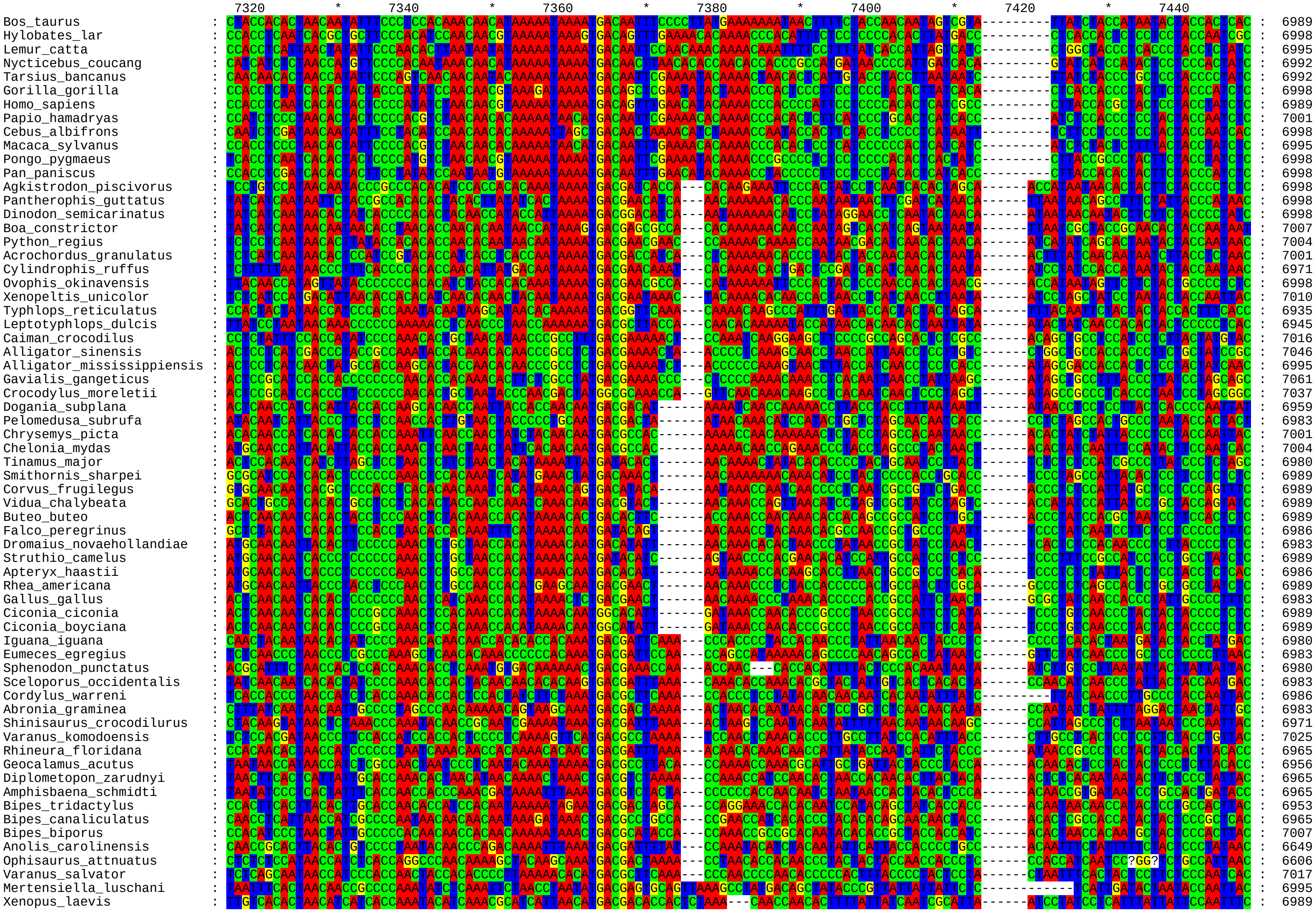
7320
7340
7360
7380
7400
7420
7440
Bos_taurus
Hylobates_lar
Lemur_catta
Nycticebus_coucang
Tarsius_bancanus
Gorilla_gorilla
Homo_sapiens
Papio_hamadryas
Cebus_albifrons
Macaca_sylvanus
Pongo_pygmaeus
Pan_paniscus
Agkistrodon_piscivorus
Pantherophis_guttatus
Dinodon_semicarinatus
Boa_constrictor
Python_regius
Acrochordus_granulatus
Cylindrophis_ruffus
Ovophis_okinavensis
Xenopeltis_unicolor
Typhlops_reticulatus
Leptotyphlops_dulcis
Caiman_crocodilus
Alligator_sinensis
Alligator_mississippiensis
Gavialis_gangeticus
Crocodylus_moreletii
Dogania_subplana
Pelomedusa_subrufa
Chrysemys_picta
Chelonia_mydas
Tinamus_major
Smithornis_sharpei
Corvus_frugilegus
Vidua_chalybeata
Buteo_buteo
Falco_peregrinus
Dromaius_novaehollandiae
Struthio_camelus
Apteryx_haastii
Rhea_americana
Gallus_gallus
Ciconia_ciconia
Ciconia_boyciana
Iguana_iguana
Eumeces_egregius
Sphenodon_punctatus
Sceloporus_occidentalis
Cordylus_warreni
Abronia_graminea
Shinisaurus_crocodilurus
Varanus_komodoensis
Rhineura_floridana
Geocalamus_acutus
Diplometopon_zarudnyi
Amphisbaena_schmidti
Bipes_tridactylus
Bipes_canaliculatus
Bipes_biporus
Anolis_carolinensis
Ophisaurus_attnuatus
Varanus_salvator
Mertensiella_luschani
Xenopus_laevis

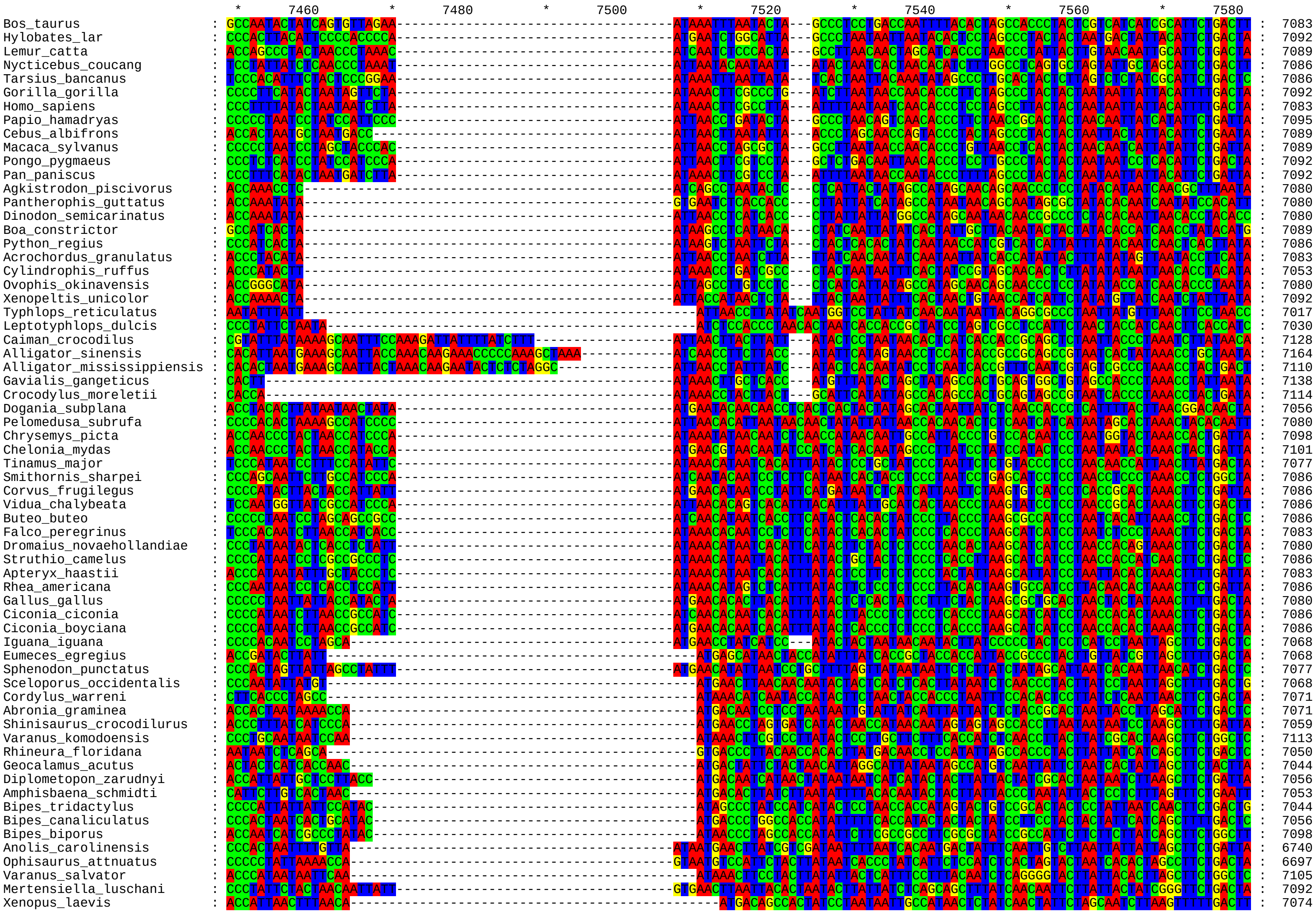
7460
7480
7500
7520
7540
7560
7580
Bos_taurus 7083
Hylobates_lar 7092
Lemur_catta 7089
Nycticebus_coucang 7086
Tarsius_bancanus 7086
Gorilla_gorilla 7092
Homo_sapiens 7083
Papio_hamadryas 7095
Cebus_albifrons 7089
Macaca_sylvanus 7089
Pongo_pygmaeus 7092
Pan_paniscus 7092
Agkistrodon_piscivorus 7080
Pantherophis_guttatus 7080
Dinodon_semicarinatus 7080
Boa_constrictor 7089
Python_regius 7086
Acrochordus_granulatus 7083
Cylindrophis_ruffus 7053
Ovophis_okinavensis 7080
Xenopeltis_unicolor 7092
Typhlops_reticulatus 7017
Leptotyphlops_dulcis 7030
Caiman_crocodilus 7128
Alligator_sinensis 7164
Alligator_mississippiensis 7110
Gavialis_gangeticus 7138
Crocodylus_moreletii 7114
Dogania_subplana 7056
Pelomedusa_subrufa 7080
Chrysemys_picta 7098
Chelonia_mydas 7101
Tinamus_major 7077
Smithornis_sharpei 7086
Corvus_frugilegus 7086
Vidua_chalybeata 7086
Buteo_buteo 7086
Falco_peregrinus 7083
Dromaius_novaehollandiae 7080
Struthio_camelus 7086
Apteryx_haastii 7083
Rhea_americana 7086
Gallus_gallus 7080
Ciconia_ciconia 7086
Ciconia_boyciana 7086
Iguana_iguana 7068
Eumeces_egregius 7068
Sphenodon_punctatus 7077
Sceloporus_occidentalis 7068
Cordylus_warreni 7071
Abronia_graminea 7071
Shinisaurus_crocodilurus 7059
Varanus_komodoensis 7113
Rhineura_floridana 7050
Geocalamus_acutus 7044
Diplometopon_zarudnyi 7056
Amphisbaena_schmidti 7053
Bipes_tridactylus 7044
Bipes_canaliculatus 7056
Bipes_biporus 7098
Anolis_carolinensis 6740
Ophisaurus_attnuatus 6697
Varanus_salvator 7105
Mertensiella_luschani 7092
Xenopus_laevis 7074

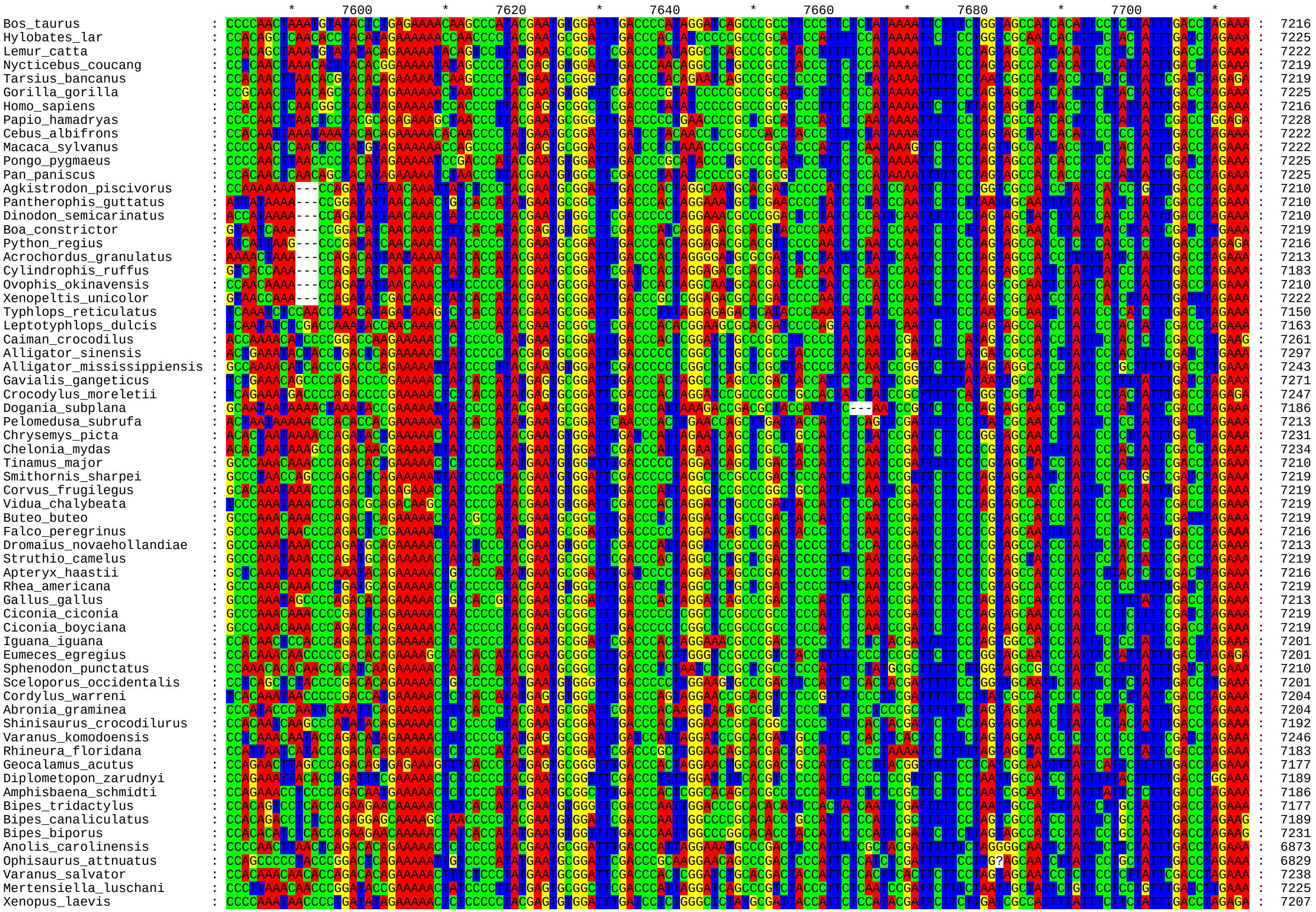
* 7600 * 7620 * 7640 * 7660 * 7680 * 7700 *
Bos_taurus : 7216
Hylobates_lar : 7225
Lemur_catta : 7222
Nycticebus_coucang : 7219
Tarsius_bancanus : 7219
Gorilla_gorilla : 7225
Homo_sapiens : 7216
Papio_hamadryas : 7228
Cebus_albifrons : 7222
Macaca_sylvanus : 7222
Pongo_pygmaeus : 7225
Pan_paniscus : 7225
Agkistrodon_piscivorus : 7210
Pantherophis_guttatus : 7210
Dinodon_semicarinatus : 7210
Boa_constrictor : 7219
Python_regius : 7216
Acrochordus_granulatus : 7213
Cylindrophis_ruffus : 7183
Ovophis_okinavensis : 7210
Xenopeltis_unicolor : 7222
Typhlops_reticulatus : 7150
Leptotyphlops_dulcis : 7163
Caiman_crocodilus : 7261
Alligator_sinensis : 7297
Alligator_mississippiensis : 7243
Gavialis_gangeticus : 7271
Crocodylus_moreletii : 7247
Dogania_subplana : 7186
Pelomedusa_subrufa : 7213
Chrysemys_picta : 7231
Chelonia_mydas : 7234
Tinamus_major : 7210
Smithornis_sharpei : 7219
Corvus_frugilegus : 7219
Vidua_chalybeata : 7219
Buteo_buteo : 7219
Falco_peregrinus : 7216
Dromaius_novaehollandiae : 7213
Struthio_camelus : 7219
Apteryx_haastii : 7216
Rhea_americana : 7219
Gallus_gallus : 7213
Ciconia_ciconia : 7219
Ciconia_boyciana : 7219
Iguana_iguana : 7201
Eumeces_egregius : 7201
Sphenodon_punctatus : 7210
Sceloporus_occidentalis : 7201
Cordylus_warreni : 7204
Abronia_graminea : 7204
Shinisaurus_crocodilurus : 7192
Varanus_komodoensis : 7246
Rhineura_floridana : 7183
Geocalamus_acutus : 7177
Diplometopon_zarudnyi : 7189
Amphisbaena_schmidti : 7186
Bipes_tridactylus : 7177
Bipes_canaliculatus : 7189
Bipes_biporus : 7231
Anolis_carolinensis : 6873
Ophisaurus_attnuatus : 6829
Varanus_salvator : 7238
Mertensiella_luschani : 7225
Xenopus_laevis : 7207

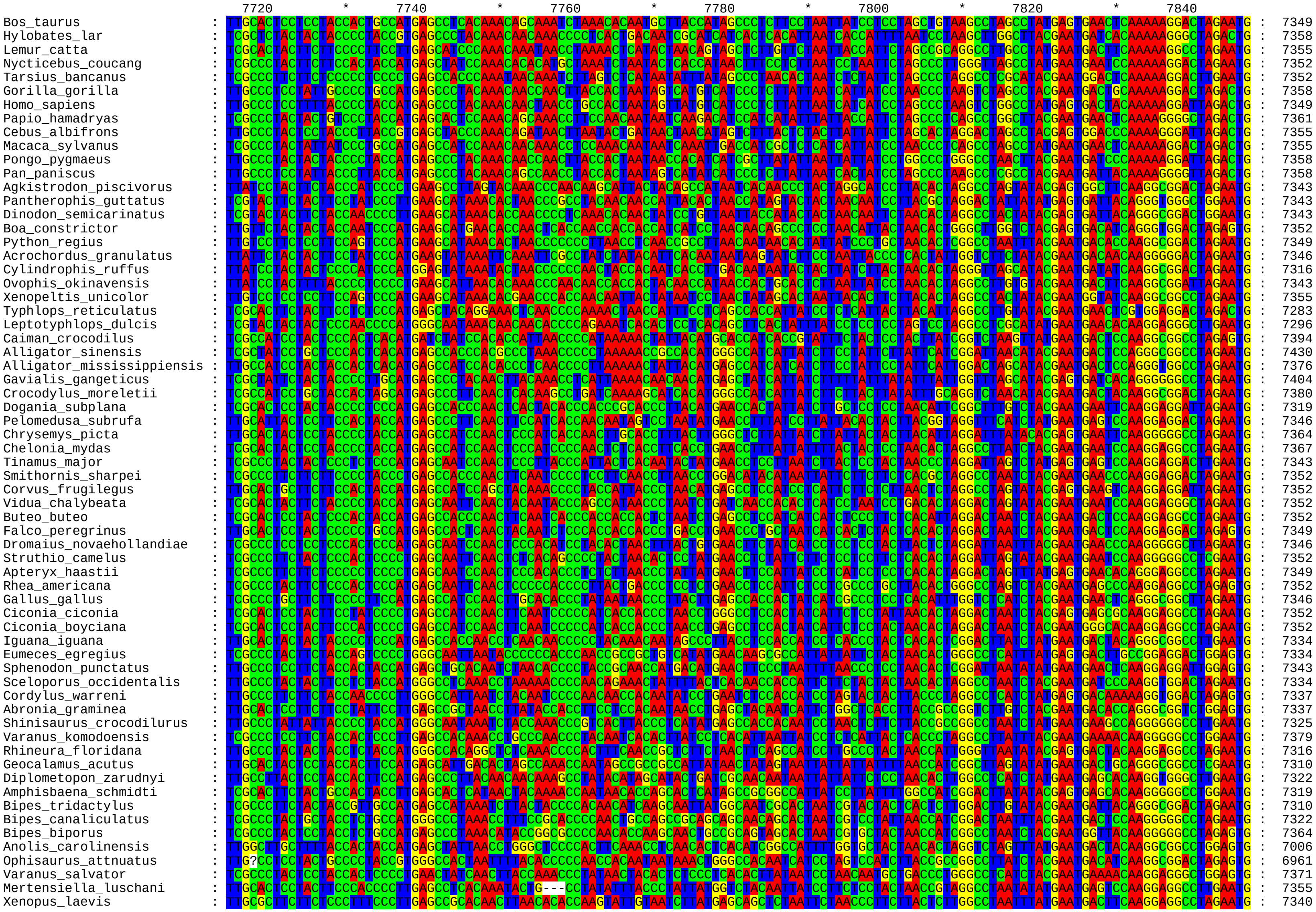
7720 * 7740 * 7760 * 7780 * 7800 * 7820 * 7840
Bos_taurus : 7349
Hylobates_lar : 7358
Lemur_catta : 7355
Nycticebus_coucang : 7352
Tarsius_bancanus : 7352
Gorilla_gorilla : 7358
Homo_sapiens : 7349
Papio_hamadryas : 7361
Cebus_albifrons : 7355
Macaca_sylvanus : 7355
Pongo_pygmaeus : 7358
Pan_paniscus : 7358
Agkistrodon_piscivorus : 7343
Pantherophis_guttatus : 7343
Dinodon_semicarinatus : 7343
Boa_constrictor : 7352
Python_regius : 7349
Acrochordus_granulatus : 7346
Cylindrophis_ruffus : 7316
Ovophis_okinavensis : 7343
Xenopeltis_unicolor : 7355
Typhlops_reticulatus : 7283
Leptotyphlops_dulcis : 7296
Caiman_crocodilus : 7394
Alligator_sinensis : 7430
Alligator_mississippiensis : 7376
Gavialis_gangeticus : 7404
Crocodylus_moreletii : 7380
Dogania_subplana : 7319
Pelomedusa_subrufa : 7346
Chrysemys_picta : 7364
Chelonia_mydas : 7367
Tinamus_major : 7343
Smithornis_sharpei : 7352
Corvus_frugilegus : 7352
Vidua_chalybeata : 7352
Buteo_buteo : 7352
Falco_peregrinus : 7349
Dromaius_novaehollandiae : 7346
Struthio_camelus : 7352
Apteryx_haastii : 7349
Rhea_americana : 7352
Gallus_gallus : 7346
Ciconia_ciconia : 7352
Ciconia_boyciana : 7352
Iguana_iguana : 7334
Eumeces_egregius : 7334
Sphenodon_punctatus : 7343
Sceloporus_occidentalis : 7334
Cordylus_warreni : 7337
Abronia_graminea : 7337
Shinisaurus_crocodilurus : 7325
Varanus_komodoensis : 7379
Rhineura_floridana : 7316
Geocalamus_acutus : 7310
Diplometopon_zarudnyi : 7322
Amphisbaena_schmidti : 7319
Bipes_tridactylus : 7310
Bipes_canaliculatus : 7322
Bipes_biporus : 7364
Anolis_carolinensis : 7006
Ophisaurus_attnuatus : 6961
Varanus_salvator : 7371
Mertensiella_luschani : 7355
Xenopus_laevis : 7340

```
                                   *      7860         *      7880         *      7900         *      7920         *      7940         *      7960         *      7980
Bos_taurus                 : AACCGAA------------------------------------------ATGCTAAAATACATTATTCCAACAATTATACTTATACCCCTAACCTGGTTATCAAAAAATAAT---ATAATTTGGGTTAACTCC :  7437
Hylobates_lar              : GGTCGAA------------------------------------------ATGCTAAAGCTAATTATCCCCACCACTATACTATTACCCCTGACATGACTATCTAAAAAACAC---ATAATCTGAATTAACACA :  7446
Lemur_catta                : AGTAGAA------------------------------------------ATGCTAAAAATCATTATCCCTACAATTATACTATTCCCAGTAACTTGATGTTCCAGTAGCCCT---ATAATCTGAATTAACACA :  7443
Nycticebus_coucang         : ACAAGAA------------------------------------------ATGCTAAAAATCATCATCCCGACTATTATACTAGTTCCAGTGACCTGGTTATCTCATCACCGC---ACACTGTGAATCAATGTA :  7440
Tarsius_bancanus           : AACCGAA------------------------------------------ATGCTAAAAATCATCCTACCAACATTTATGTTAATTCCACTAACATGACACTCCAAAAAAAAC---AATATCTGAATTAATACT :  7440
Gorilla_gorilla            : GACCGAA------------------------------------------ATGCTAAAACTAATCGCCCCAACAATTATACTACTACCGCTAACATGACTCTCCAAAAAACAT---ATAATTTGAATCAACACA :  7446
Homo_sapiens               : AGCCGAA------------------------------------------ATGCTAAAACTAATCGTCCCAACAATTATATTACTACCACTGACATGACTTTCCAAAAAGCAC---ATAATTTGAATCAACACA :  7437
Papio_hamadryas            : AACT---------------------------------------------ATGTTAAAAATAGTTATCCCCACAACTATACTACTACCAACAACATGACTCTCCAAAGACAGC---ACAATCTGAATCAATTTA :  7446
Cebus_albifrons            : AGTTGAT------------------------------------------ATGCTAAAAATTATTCTCCCGACAATCATAATAATCCCAACAATATATCTATCAAAAAACCAT---ATAATATGAATCAATACA :  7443
Macaca_sylvanus            : AGCTGAA------------------------------------------ATGCTAAAATTAATTATCCCCACAACCATGTTACTACCCATAACATGACTTTCTAAAAATAAT---ATAATCTGAATCAACTTA :  7443
Pongo_pygmaeus             : AACCGAA------------------------------------------ATGCTAAAACTAATTATCCCAACAGTCATACTACTACCCCTAACATGACTCTCCAAAACACAC---ATAATCTGAATCAACACC :  7446
Pan_paniscus               : AGCCGAA------------------------------------------ATGCTAAAACTAATTATCCCAACAATTATATTACTACCACTAACATGATTCTCTAAAAAACGT---ATAATTTGAATCAACACA :  7446
Agkistrodon_piscivorus     : AACAGAA------------------------------------------ATGCTAAAAACTTTTATCATAACAGCAATACTAATCCCAACAATCCTTACCCTAAAACCCAAA---ATGCTCTACCCAGCAACA :  7431
Pantherophis_guttatus      : AACAGAA------------------------------------------ATGCTAAAAATTATCTATACTACTATAATACTTATCCCAATAATTCTAGTATTAAAACCAAAA---ATAATCTATCAAACTACA :  7431
Dinodon_semicarinatus      : AACTGAA------------------------------------------ATGCTAAAAATCACTTTTATAACCATAATACTAATCCCAACAACTTTAATTCTAAAACCCAAA---ATACTTTATCAAACAACA :  7431
Boa_constrictor            : AGCAGAA------------------------------------------ATGTTAAAAATTTTAATTGCCACAATAACCCTCGTACCAATAGCCCTCTTACTAAAACCAAAA---ACACTATACCAAACAATG :  7440
Python_regius              : AACAGAA------------------------------------------ATGCTAAAAATTCTCCTCGCAACAACCATACTGATCCCAACAGCCTTCATCCTAAAACCCCAA---ATCCTACACCAAACAATA :  7437
Acrochordus_granulatus     : ATCACAC------------------------------------------ATGATAAAAATAATTATTGCAACTATAATCCTTTCCCCAACAGCACTAACACTGAAACCCAAA---ATCTTATATCAAGTAATA :  7434
Cylindrophis_ruffus        : AGCTGAC------------------------------------------ATGCTAAAAATTATTATCATAACAACAATAATAATTCCAACAACCCTACTACTAAAACCAAAG---ATTCTTTACCAAACGACT :  7404
Ovophis_okinavensis        : AACAGAG------------------------------------------ATGCTAAAAGCTCTTATCACAACAACAATACTAATCCCAACAATTCTCCTCCTCAAGCCAAAA---ACACTATATCCGGCAACA :  7431
Xenopeltis_unicolor        : AACAGAA------------------------------------------ATGCTAAAAATAATTCTCGCAACCACAGCCCTAATCCCAATAGCCCTACTCCTAAACCCAAAA---ATTATACACCAAACAATA :  7443
Typhlops_reticulatus       : AGCCAAA------------------------------------------ATGATAAAAATAATACTACCAACATTATTACTGATTCCATCTACCCTAATCCTAAAACCGAAG---CTTTTATTTCCAACCATA :  7371
Leptotyphlops_dulcis       : AGCACAC------------------------------------------ATGCTAACCCTACTAATCCCAACGGCCATAATAATCCCCATAACAATACTCCTAAAACCAAAA---ACACTCTTTCCAACATCA :  7384
Caiman_crocodilus          : GGCAGAA------------------------------------------ATGTTAAAAATCCTTACCCCCACGGCCACGTCCACCCTCATCACCTCCTTCATCCCGAACAAG---GCCACCTGACTCTCATCA :  7482
Alligator_sinensis         : AGCAGAA------------------------------------------ATGCTAAAACTTCTTCTTCCCACAACCATGCTGATCCCAACAATCAACCTCCTCCCAAACAAA---ATTACCTGGTTGCCGCCA :  7518
Alligator_mississippiensis : AGCAGAA------------------------------------------ATGCTAAAACTTTTATTAGCCACATTCATGTTAATCCCTACGACCCCCCTGCTCCCAAATAAA---ATTACTTGACTAACTCCA :  7464
Gavialis_gangeticus        : AGCAGAA------------------------------------------ATGCTAAAACTCGTAGTACCAACAATAATGTTAATCCCCTCCACCTGCTTCACACATCACAAA---ACAATCTGACTATCACCA :  7492
Crocodylus_moreletii       : AGCAGAG------------------------------------------ATGTTAAAAATCATTGTACCTACAATAATACTAATCCCCTCAGCCTGCCTAACAACCACAAAA---AACACCTGACTATCACCA :  7468
Dogania_subplana           : AGCAGAA------------------------------------------ATGCTGGGAATTATTATTCCAACAATCATACTAATTCCTACCGCCACAATATCCAAACCAACA---CACATATGATACTCCACA :  7407
Pelomedusa_subrufa         : ACCAAAT------------------------------------------ATGTTAAAATTACTTCTACCTATAATTATATTACTACCTACTTCCTGATTATGTAAAAAAAAT---CACCTCTCATACACAACC :  7434
Chrysemys_picta            : GGCAGAG------------------------------------------GTGCTAAAAATCCTACTCCCAATAACTGCACTTCTCCTAACAACCACAATCTGCAAGCCAAAA---CAACTATGACCCACCACA :  7452
Chelonia_mydas             : AGCAGAA------------------------------------------ATGCTAAAAATCTTACTCCCAACAATTATACTATTACCAACAATCACATTATGTAAACCAAAA---CAACTATGACTTTCTACA :  7455
Tinamus_major              : AGCCGAA------------------------------------------ATGTTAAAAATCATAATTCCCACTATTATACTCATCCCGACAGTCCTACTATCCCCTAAAAAT---CTTATCTGAACAAACGCA :  7431
Smithornis_sharpei         : AGCAGAA------------------------------------------ATGCTAAAAATTATCCTCCCAACCCTCACCCTCCTCCCCATAACTCTTTTATCACCACTTAAA---TACCTATGACTCAACATC :  7440
Corvus_frugilegus          : AGCAGAA------------------------------------------ATGCTAAAAATCATCATTCCAACCATCATACTCCTACCACTAACTTTCCTCTCTCCTTGCAAG---CACCTATGAACCAATACC :  7440
Vidua_chalybeata           : GGCAGAA------------------------------------------ATGCTAAAAATCATTATACCCACTATTATACTACTCCCACTGACCTTCCTATCCACACCCAAA---CACCTGTGGACCAACATT :  7440
Buteo_buteo                : AGCTGAA------------------------------------------ATGCTAAAAATCCTCCTCCCAACAATCATACTCCTCCCCACAGCCCTCCTATCTCCCCAAAAA---CTCCTATGAACCAACACC :  7440
Falco_peregrinus           : AGCAGAA------------------------------------------ATGCTAAAAATCCTCCTCCCAACCCTCCTTCTACCCCCAACAGCTCTCTTATCCCCAACTAAA---TTCCTATGAATTAATACT :  7437
Dromaius_novaehollandiae   : AGCAGAA------------------------------------------ATGCTAAAAATTATTATTCCTACAATTATACTTCTGCCTACAGCCCTCCTATCCCCAAAAAAC---CTGATCTGAACTAACACT :  7434
Struthio_camelus           : GGCAGAA------------------------------------------ATGCTAAAAATCATCATCCCCACTATTATACTCCTGCCTACAGCCCTTCTTTCTCCTAAAGCC---TCACTATGGACAAATACC :  7440
Apteryx_haastii            : GGCAGAG------------------------------------------ATGCTAAAAATTATTTTTCCCACAGCCATGCTCTTACCCATAACCCTTTTATCCCCTAAAAAC---CTTCTCTGAACGAACATT :  7437
Rhea_americana             : AGCAGAA------------------------------------------ATGCTAAAAATCATTATCCCAACAATCATACTCCTGCCCATAACCCTCCTCTCACCCCCCAAC---TTCTTGTGAACAAACACT :  7440
Gallus_gallus              : AGCAGAA------------------------------------------ATGCTAAAGATCATCCTACCAACAATCATACTCTTGCCCACAGCCCTCTTATCCCCAGCAAAA---TCCATATGAACTAACACC :  7434
Ciconia_ciconia            : AGCAGAA------------------------------------------ATGCTAAAAATTATCCTCCCAACAATCATACTCATCCCCACAGCACTCCTATCTCCCCAAAAA---TTCCTATGGACAAACACC :  7440
Ciconia_boyciana           : AGCAGAA------------------------------------------ATGCTAAAAATTATCCTCCCAACAATCATACTCATCCCTACAGCACTCCTATCTCCCCAAAAA---TTCTTATGAACAAACACC :  7440
Iguana_iguana              : AGCAGAA------------------------------------------ATGCTAAAAATTATCCTACCCACAATCCTCCTGACCCCCACAATCTTAACCACAAAAGCCCCC---CACACATTCAACACTTTC :  7422
Eumeces_egregius           : GGCAGAA------------------------------------------ATGCTAAAAATTCTGCTACCAACAACCCTACTTATCCCAACAGCGCTTCTATCCAAACCAAAC---ACCCTGTTCTTAACATAC :  7422
Sphenodon_punctatus        : AGCCGAA------------------------------------------ATGCTAAAAATTATTATTCCAACAATTTTCCTTCTCCCTGTAATCCTTTTATCAAACCCCAAG---TCAACATGAATAAACTTC :  7431
Sceloporus_occidentalis    : AGCAGAA------------------------------------------ATGCTAAAAGTAATCCTGCCCACATTAATACTTGCACCCACTGCAATAATGACAAAACCACTG---TACACCTTTAATCTCTTC :  7422
Cordylus_warreni           : GGCCGAA------------------------------------------ATGCTAAAAATCATTATCCCAACACTCCTAATAATTCCCATAGCCTCCCTCCTAAAACCAAGC---CTGCTCTTTAATGCAACC :  7425
Abronia_graminea           : GGCAGAA------------------------------------------ATGCTAAAAATCCTCATCCCAACCACAATACTTATTCCCGCAACGATATTCTGCAGCCAAAAA---CTTATTTTTACCGCATTT :  7425
Shinisaurus_crocodilurus   : GGCCGAA------------------------------------------ATGCTAACAATTCTTTTCCCAACACTAATACTAATCCCCACCTCACTTCTCATAAAACAAAAA---CATCTTTTTGCCTCCTTC :  7413
Varanus_komodoensis        : AGCAATC------------------------------------------ATGCTAAAAATCCTCATTCCCTCCATTATACTAATTCCAACAGTTATATTAACTAAGCCCAAA---CACCTATTCATCTCATTT :  7467
Rhineura_floridana         : AGCAGAA------------------------------------------ATGTTAAAAGTAATTATCCCAACAGCAATGTTAATCCCACTAGCACTAGTGTTAAAACCAAAC---ATATTATTTACAACATAC :  7404
Geocalamus_acutus          : AGCAGAT------------------------------------------ATGTTAAAAGTACTTCTACCAACTCTAATACTCATTCCAACCACCATACTCAGCCCACAAAAT---CTAATTACTCCAATCTAT :  7398
Diplometopon_zarudnyi      : AGCGGAA------------------------------------------ATGCTAAAAATCCTCCTACCTACAATTATACTAATTCCCTCTACCGCACTCATCCCTCAAAAA---CTTATTTTTATACACTTT :  7410
Amphisbaena_schmidti       : AGCCGAC------------------------------------------ATGCTAAAGGTACTACTACCAACCCTGATACTGATTCCCACCGCCATCTTTACACCACAAAAC---CACGTCTTCAACAACTAC :  7407
Bipes_tridactylus          : AGCAGAA------------------------------------------ATGTTAAAAGTACTAATCCCAACGCTAATACTAATTCCTACCTCCGCCCTACTGCACCAACGA---ACCCTATTTCAAGCCTAC :  7398
Bipes_canaliculatus        : AGCCGAA------------------------------------------ATGCTAAAGATTATTTTACCAACCCTGCTCCTCATTCCCACCGCAGCCCTAGCTCACGAACGC---ACCTTATTCACAGCCTAC :  7410
Bipes_biporus              : GGCCGAA------------------------------------------ATGCTAAAAATCATTCTCCCAACCATAATACTTATCCCAACCGCAGCCCTACTGCATCCTCGC---TTCCTATTCACCGCTTAC :  7452
Anolis_carolinensis        : GGCGGAG------------------------------------------ATGTTAAAAATTATTTTACCAACAATTTTACTTACCCCAACAGCCTTCATTATTAAACCAAGC---ATTCTATTTTCCTCCTAC :  7094
Ophisaurus_attnuatus       : GGCCGAA------------------------------------------ATGCTAAAAATCCTACTCCCTACAATAATACTAATCCCAACAACACTATTCTTGAGCCCCTCA---CTCCTCTTTACCTCACAC :  7049
Varanus_salvator           : AACAAGC------------------------------------------ATGCTAAAAACTCTCCTCCCAACAATTTTACTCATCCCAATAATTATGCTAACTAAACCCAAA---TTTCTGTTTACCTCATTC :  7459
Mertensiella_luschani      : GGCTGAA------------------------------------------ATGCTAAAAATTCTGATTCCAACCATTATGCTACTCCCAATAACATGACTAGCTAGTAAAAAA---TGACTTTGAACATACCTT :  7443
Xenopus_laevis             : AGCTGAA------------------------------------------ATGTTAAAAATTTTATTACCAACATTAATGCTAATCCCATCAACATGATTAACAAATAAAAAA---TGATTATGACCGTCCTTA :  7428
```

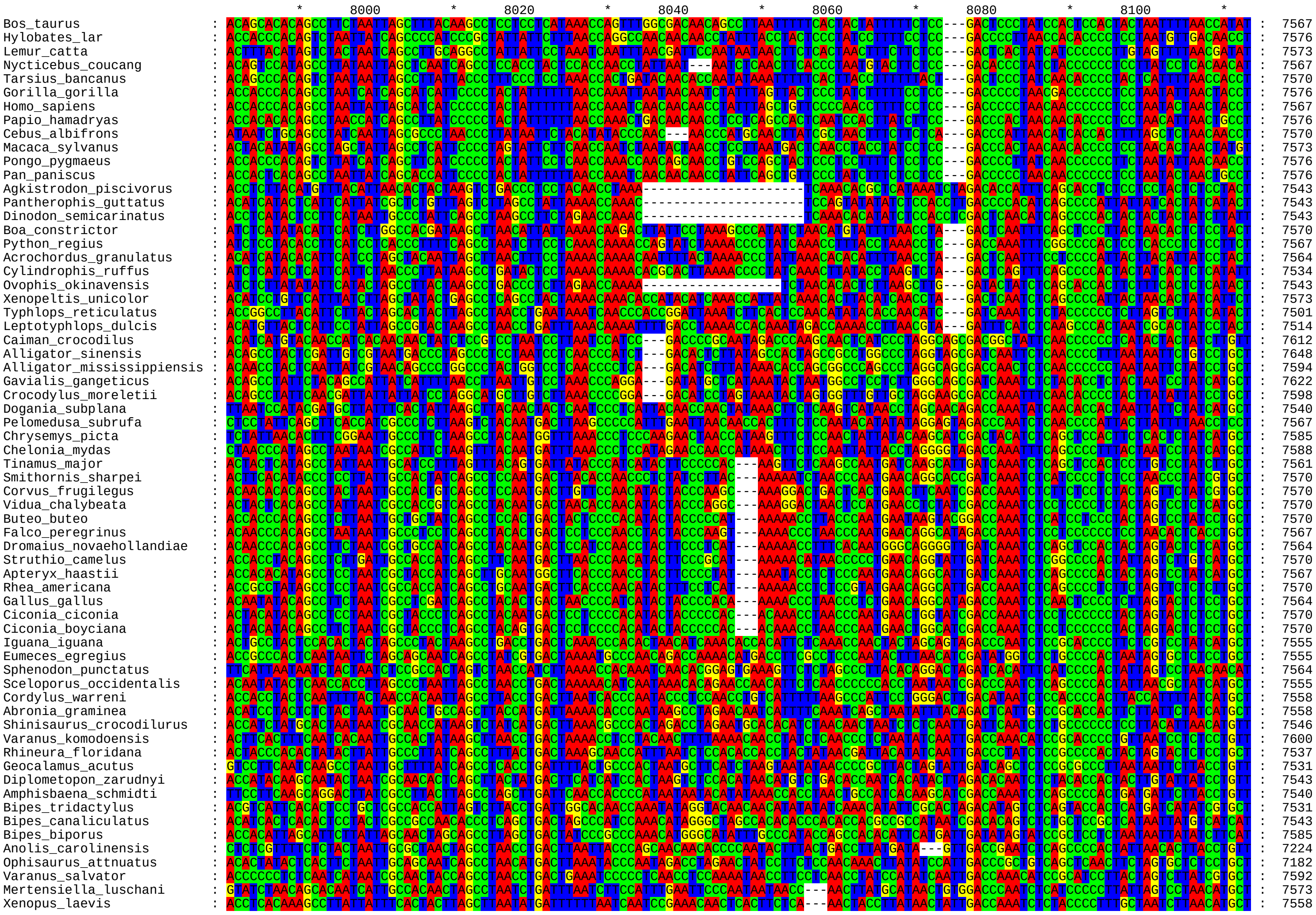

8000
8020
8040
8060
8080
8100
Bos_taurus 7567
Hylobates_lar 7576
Lemur_catta 7573
Nycticebus_coucang 7567
Tarsius_bancanus 7570
Gorilla_gorilla 7576
Homo_sapiens 7567
Papio_hamadryas 7576
Cebus_albifrons 7570
Macaca_sylvanus 7573
Pongo_pygmaeus 7576
Pan_paniscus 7576
Agkistrodon_piscivorus 7543
Pantherophis_guttatus 7543
Dinodon_semicarinatus 7543
Boa_constrictor 7570
Python_regius 7567
Acrochordus_granulatus 7564
Cylindrophis_ruffus 7534
Ovophis_okinavensis 7543
Xenopeltis_unicolor 7573
Typhlops_reticulatus 7501
Leptotyphlops_dulcis 7514
Caiman_crocodilus 7612
Alligator_sinensis 7648
Alligator_mississippiensis 7594
Gavialis_gangeticus 7622
Crocodylus_moreletii 7598
Dogania_subplana 7540
Pelomedusa_subrufa 7567
Chrysemys_picta 7585
Chelonia_mydas 7588
Tinamus_major 7561
Smithornis_sharpei 7570
Corvus_frugilegus 7570
Vidua_chalybeata 7570
Buteo_buteo 7570
Falco_peregrinus 7567
Dromaius_novaehollandiae 7564
Struthio_camelus 7570
Apteryx_haastii 7567
Rhea_americana 7570
Gallus_gallus 7564
Ciconia_ciconia 7570
Ciconia_boyciana 7570
Iguana_iguana 7555
Eumeces_egregius 7555
Sphenodon_punctatus 7564
Sceloporus_occidentalis 7555
Cordylus_warreni 7558
Abronia_graminea 7558
Shinisaurus_crocodilurus 7546
Varanus_komodoensis 7600
Rhineura_floridana 7537
Geocalamus_acutus 7531
Diplometopon_zarudnyi 7543
Amphisbaena_schmidti 7540
Bipes_tridactylus 7531
Bipes_canaliculatus 7543
Bipes_biporus 7585
Anolis_carolinensis 7224
Ophisaurus_attnuatus 7182
Varanus_salvator 7592
Mertensiella_luschani 7573
Xenopus_laevis 7558

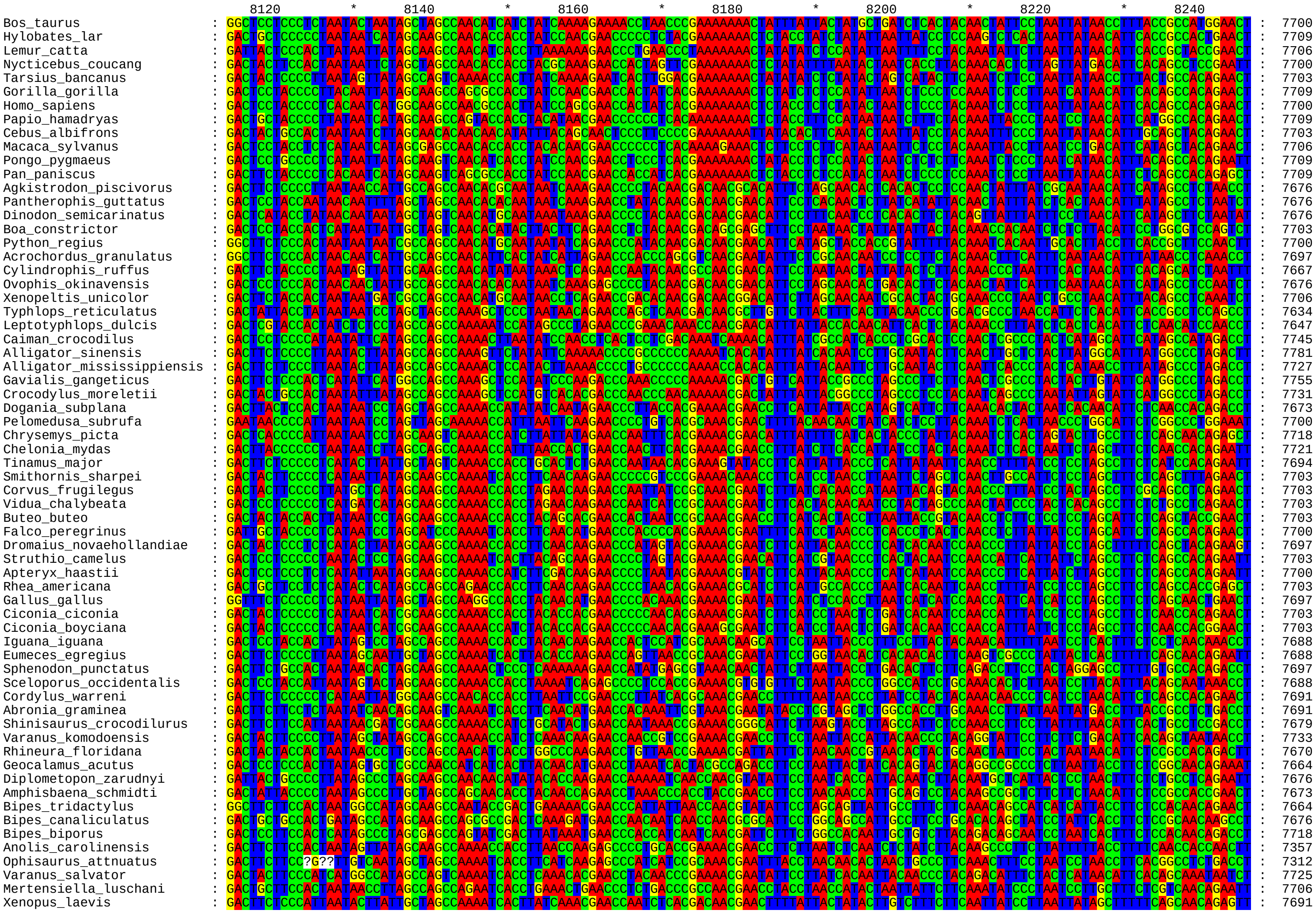
8120 * 8140 * 8160 * 8180 * 8200 * 8220 * 8240
Bos_taurus : 7700
Hylobates_lar : 7709
Lemur_catta : 7706
Nycticebus_coucang : 7700
Tarsius_bancanus : 7703
Gorilla_gorilla : 7709
Homo_sapiens : 7700
Papio_hamadryas : 7709
Cebus_albifrons : 7703
Macaca_sylvanus : 7706
Pongo_pygmaeus : 7709
Pan_paniscus : 7709
Agkistrodon_piscivorus : 7676
Pantherophis_guttatus : 7676
Dinodon_semicarinatus : 7676
Boa_constrictor : 7703
Python_regius : 7700
Acrochordus_granulatus : 7697
Cylindrophis_ruffus : 7667
Ovophis_okinavensis : 7676
Xenopeltis_unicolor : 7706
Typhlops_reticulatus : 7634
Leptotyphlops_dulcis : 7647
Caiman_crocodilus : 7745
Alligator_sinensis : 7781
Alligator_mississippiensis : 7727
Gavialis_gangeticus : 7755
Crocodylus_moreletii : 7731
Dogania_subplana : 7673
Pelomedusa_subrufa : 7700
Chrysemys_picta : 7718
Chelonia_mydas : 7721
Tinamus_major : 7694
Smithornis_sharpei : 7703
Corvus_frugilegus : 7703
Vidua_chalybeata : 7703
Buteo_buteo : 7703
Falco_peregrinus : 7700
Dromaius_novaehollandiae : 7697
Struthio_camelus : 7703
Apteryx_haastii : 7700
Rhea_americana : 7703
Gallus_gallus : 7697
Ciconia_ciconia : 7703
Ciconia_boyciana : 7703
Iguana_iguana : 7688
Eumeces_egregius : 7688
Sphenodon_punctatus : 7697
Sceloporus_occidentalis : 7688
Cordylus_warreni : 7691
Abronia_graminea : 7691
Shinisaurus_crocodilurus : 7679
Varanus_komodoensis : 7733
Rhineura_floridana : 7670
Geocalamus_acutus : 7664
Diplometopon_zarudnyi : 7676
Amphisbaena_schmidti : 7673
Bipes_tridactylus : 7664
Bipes_canaliculatus : 7676
Bipes_biporus : 7718
Anolis_carolinensis : 7357
Ophisaurus_attnuatus : 7312
Varanus_salvator : 7725
Mertensiella_luschani : 7706
Xenopus_laevis : 7691

```
                                   *     8260         *     8280         *     8300         *     8320         *     8340         *     8360         *     838
Bos_taurus                     : AATCTTATTTTATATTCTATTTGAAGCAACACTAGTCCCAACACTCATTATTATTACCCGATGAGGAAACCAAACAGAACGCCTAAACGCCGGACTCTATTTCCTATTCTATACACTAGCTGGCTCCCTACCC : 7833
Hylobates_lar                  : AATAATATTCTACGTCCTCTTTGAAACTACACTCATCCCCACTCTAGTTATCATTACTCGATGGGGTAACCAACCGGAACGCCTAAACGCAGGCTCATACTTTCTATTCTACACCCTAGTAGGCTCCCTCCCC : 7842
Lemur_catta                    : AATTCTATTTTACATTCTATTTGAAGCCACATTAATCCCAACTCTAATTATCATTACCCGTTGAGGTAATCAAACAGAACGATTAAACGCAGGCTTATACTTTCTATTTTATACTCTCATTGGATCCTTACCA : 7839
Nycticebus_coucang             : GATCTTATTTTACATCTTATTCGAAGCTACACTAATCCCAACCCTAGTTATCATCACCCGATGGGGAAATCAGACAGAACGATTAAACGCTGGATTATATTTCCTGTTCTACACACTACTAGGATCACTCCCC : 7833
Tarsius_bancanus               : TGTCCTTTTCTACATCCTATTTGAAGCAACCTTAATTCCCACCCTCATTATTATTACACGATGAGGAAACCAGACCGAGCGACTAAACGCCGGCCTTTACTTTTTATTTTATACACTAATTGGATCACTCCCC : 7836
Gorilla_gorilla                : AATCATATTCTATATCTTCTTCGAAGCCACACTTATCCCTACCCTAGTTATCATCACCCGATGAGGCAACCAACCCGAACGTCTAAACGCGGGTACATACTTCCTATTCTACACCCTAGTAGGATCCCTCCCC : 7842
Homo_sapiens                   : AATCATATTTTATATCTTCTTCGAAACCACACTTATCCCCACCTTGGCTATCATCACCCGATGAGGCAACCAGCCAGAACGCCTGAACGCAGGCACATACTTCCTATTCTACACCCTAGTAGGCTCCCTTCCC : 7833
Papio_hamadryas                : AATCATATTTTATATCCTGTTCGAAACCACTCTCATCCCAACCCTTATTATCATCACTAAATGAGGCAACCAAGCAGAACGTCTCAACGCAGGTTCATACTTCCTATTCTATACCCTAACCGGCTCCTTACCA : 7842
Cebus_albifrons                : AATCCTATTCTACATTTTATTTGAAACTACTCTAATTCCAACCCTAATTATTATTACCCGCTGAGGATACCAGCCAGAGCGCCTCAACGCCGGCTCGTACTTTCTATTCTACACACTAGCTGGATCACTTCCA : 7836
Macaca_sylvanus                : AATTATATTCTACATCCTGTTTGAAACCACCCTTATCCCCACTCTAATTATTATCACCAAATGGGGCAGCCAAGCAAAACGCCTTAATGCAAGCACATACTTCCTATTCTACACACTAACCGGCTCCCTACCC : 7839
Pongo_pygmaeus                 : AATTATATTTTACATCCTCTTCGAAACCACTCTCATCCCCACCCTAATCATTATCACCCGCTGAGGCAACCAACCAGAACGCTTAAACGCAGGCACATATTTTCTATTCTACACACTAGTGGGCTCCCTCCCC : 7842
Pan_paniscus                   : AATTATATTTTACATCTTCTTCGAAACCACACTCATCCCCACCCTAGCCATCATCACCCGATGGGGTAACCAACCAGAACGCCTGAACGCAGGTACATACTTCCTATTCTATACCCTAGTAGGCTCCCTCCCC : 7842
Agkistrodon_piscivorus         : TACCCTAATGTACATCATATTTGAAGCTACCCTTATCCCCACCCTAATTATCATTACACGCTGAGGACAACAAACAGAACGACTAACCGCAGGAACATACTTTATGCTATACACACTAACAACTTCACTACCC : 7809
Pantherophis_guttatus          : AACCCTCATGTATATTATATTCGAAGCTACCCTAATTCCAACTCTTATTATTATTACACGATGAGGACAACAAACTGAACGCCTAACCGCAGGCACATACTTCATACTATATACACTAATAACCTCAATACCG : 7809
Dinodon_semicarinatus          : CACCCTAATATACATTATATTCGAAGCAACCTTAATCCCAACCCTAATCATTATTACACGATGAGGACAACAAACTGAGCGCCTAACCGCAGGCACATATTTCATAATATATACACTAACAACTTCAATACCC : 7809
Boa_constrictor                : AACCCTAATATACGTTATATTTGAGGCCACCTTAATTCCAACACTCATCCTAATTACGCGATGAGGTCAACAAGCAGAGCGACTAACCGCGGGAACATACTTCATTATATACACAATAACAACCTCAATACCC : 7836
Python_regius                  : AACCTTAATGTATATTATATTCGAAGCTACCCTGGTCCCCACCCTTATTATCATCACACGATGGGGCCAACAAGCAGAACGACTAACAGCAGGAACCTACTTCATAATATATACCCTAACAACCTCCATACCA : 7833
Acrochordus_granulatus         : AACCACAATATACATTATATTCGAAGCTACACTTATCCCAACATTAATTATCATCACACGATGAGGCCACCAATCTGAACGACTTACCGCAGGAACCTATTTTATACTTTACACCTTAGCAACATCTATACCC : 7830
Cylindrophis_ruffus            : AACGTTAATATATATTATATTTGAAGCCACCCTAATTCCAACACTAATTATCATCACACGATGAGGCCAACAATCCGAACGATTAACAGCAGGAACCTACTTCATTATATACACCCTAACAACATCAATACCC : 7800
Ovophis_okinavensis            : CACTCTCATATACATCATATTCGAAGCCACCCTAATCCCCACGCTAATCATTATTACCCGATGAGGACAGCAAACAGAACGACTGACCGCAGGAACATACTTTATACTGTACACACTTGCAACCTCCTTACCC : 7809
Xenopeltis_unicolor            : AACTCTAATATACATTATATTTGAAGCAACCCTTATCCCAACCCTAATTGTAATCACACGATGAGGACAACAGGCCGAACGATTAACCGCTGGCACTTACTTTATAATATATACCCTGGCAACATCAATACCA : 7839
Typhlops_reticulatus           : CACCCTAATATACATTGCATTTGAAGCCACCCTTATTCCAACTATAGTCTTAATCACCCGATGAGGACACCAGGCTGAACGATTAACCGCTGGCTCCTATTTCATAATCTACACACTACTAAGCTCAATACCC : 7767
Leptotyphlops_dulcis           : AATAGCCTTTTTCATCATATTTGAAGCGACCTTAATCCCAACACTAATCATTATCACACGATGAGGACAGCAACAAGAACGCCTAACTGCAGGAACATACTTTATACTCTACACCATTATAACCTCTATACCA : 7780
Caiman_crocodilus              : AATACTATTCTACATCACATTCGAAGCTACACTAATCCCAACTCTTGTAATCATCTCCCGATGGGGCAGCCAACTAGCACGGCTAAACGCTGGAACCTACTTCCTATTCTACACTATTACCACCTCCATCCCC : 7878
Alligator_sinensis             : AATACTATTCTACATCTCCTTCGAAGCCACCCTAATCCCCACCCTTGTAATCATCTCCCGCTGAGGGGCTCAAACAGACCGCCTAAACGCAGGTATTTATTTTTGTTTTACACTATCGCCAGCTCAATCCCG : 7914
Alligator_mississippiensis     : CATATTATTTTACATTTCCTTTGAAGCCACCTTAATCCCCACCCTCATCATTATCTCACGGTGAGGAGCCCAAGCAGATCGCCTAAACGCAGGCATTTACTTCCTGTTTTATACCATTACTAGCTCAATTCCA : 7860
Gavialis_gangeticus            : AATATTATTTTATACCGCGTTCGAAGCAACCCTTATTCCAACACTAATGGTAATTGCTCGTTGAGGCACCCAAACAGAACGCCTGGGGGCCGGAATATATTTCCTACTCTACACCATCACCAGCTCAATACCA : 7888
Crocodylus_moreletii           : AATATTATTTTACATCGCCTTTGAAGCAACCCTCATTCCCACCCTAATAGTAATCGCCCGATGAGGCTCCCAGACAGAACGGCTCGGGGCCGGTCTATACTTCCTCCTATACACCATCACTAGCTCTATACCG : 7864
Dogania_subplana               : AATAATATTTTTTATTTTTTTCGAAGCCACACTTATCCCAACACTAATAATAATCACACGCTGAGGAAACCAAATAGAACGCCTAAATGCTGGAACCTATTTCCTATTTTATACACTTATATGATCACTACCC : 7806
Pelomedusa_subrufa             : AATATTGTTTTTTACAATATTTGAAGCCACCCTAATCCCCACCTTAATTATCATCACACGATGAGGAAATCAAATAGAACGACTAAGCGCTGGTACCTATTTCCTATTTTACACCCTAATCGGCTCTCTCCCA : 7833
Chrysemys_picta                : AATTATATTCTTCATTATATTCGAAACTACACTAATCCCAACACTAGCAATTATCACACGTTGAGGTAACCAAATAGAACGGCTAAACGCTGGGACTTATTTCCTATTCTACACCCTTATTGGATCCCTCCCA : 7851
Chelonia_mydas                 : AATTATATTTTTTATCACATTTGAAACCACACTCATCCCAACATTAGTAATCATCACACGATGAGGTAATCAAATAGAACGACTAAATGCAGGAACCTACTTCCTATTTTATACCCTTATTGGATCTCTCCCA : 7854
Tinamus_major                  : ATCCCTATTTTACATTTCCTTTGAAGCTACCCTGATTCCAACACTAATCCTAATCACACGATGGGGAAACCAACCAGAACGTCTCAGCGCCGGTATTTACCTCTTATTCTACACCCTAATCAGCTCCCTCCCC : 7827
Smithornis_sharpei             : GACACTTTTCTACATCACATTTGAAGCAACCTTAATCCCCACTCTTATCCTCATCACCCGATGAGGCAGTCAACCAGAACGCCTAAACGCCGGAATTTACTTGCTATTCTACACACTCATCAGCTCTCTCCCA : 7836
Corvus_frugilegus              : AATACTATTCTACATCGCATTTGAGGCAACCCTTATTCCAACTCTAATCCTCATTACACGATGAGGAAATCAACCTGAACGACTAAACGCAGGCATTTACCTACTATTCTACACGCTTGCCAGCTCTCTCCCC : 7836
Vidua_chalybeata               : GATACTATTCTACATCGCATTTGAGGCAACCCTAATCCCAACCCTAATCCTCATTACACGATGAGGAAACCAACCAGAACGCCTAAACGCAGGCATCTACCTACTATTTTACACACTCGCCAGCTCACTCCCC : 7836
Buteo_buteo                    : AATACTATTCTACATCACATTCGAAGCAACCCTAATCCCCACCCTAATCCTAATCACACGCTGAGGAAGCCAACCAGAACGCCTAAGCGCAGGCATCTATCTACTATTCTACACCCTTATCAGCTCCCTACCG : 7836
Falco_peregrinus               : TACACTATTCTATGTCTCATTCGAAGCAACTCTAATCCCAACCCTGGCCCTGATCACTCGATGAGGAAATCAACCAGACCGTCTAAGTGCAGGAATTTACCTACTATTTTACACCCTTATCAGCTCCTTACCA : 7833
Dromaius_novaehollandiae       : AATCCTATTTTATATCTCATTCGAAGCAACTCTCATCCCAACACTAATTCTAATCACTCGATGAGGAAATCAGCCTGAACGACTAAGTGCTGGCATTTACCTACTATTCTACACTCTAATCAGCTCTTTACCG : 7830
Struthio_camelus               : AACCCTATTTTACATTTCATTCGAAGCCACCCTAATCCCCACACTAATCCTTATTACCCGTTGAGGTAATCAACCCGAGCGACTTAGTGCCGGAATCTATTTACTGTTTTACACTCTCATTAGCTCACTACCC : 7836
Apteryx_haastii                : AATCCTATTCTACATCTCATTTGAAGCCACCCTAATTCCTACACTAATCCTAATTACTCGATGAGGAAACCAACCTGAACGACTTAGTGCTGGTATCTACCTACTATTTTATACCCTTATCAGCTCCCTTCCA : 7833
Rhea_americana                 : AATCCTATTCTATATCTCATTCGAAGCAACCCTAATCCCCACCCTAATCTTAATCACCCGATGAGGAAATCAACCAGAACGACTTAGTGCTGGCATTTACCTCCTATTCTACACTCTAATCAGCTCCCTCCCA : 7836
Gallus_gallus                  : CATACTATTCTACATCTCATTCGAAGCAACCCTAATCCCAACCCTCATCTTAATCACACGCTGAGGAAACCAACCAGAACGACTTAGCGCAGGCATTTACCTTCTATTCTATACCCTAATTAGCTCTCTTCCC : 7830
Ciconia_ciconia                : AACACTATTCTACATCTCATTCGAAGCAACCCTAATCCCCACTCTGATTCTAATCACACGATGAGGTAACCAACCAGAACGACTCAGTGCTGGCATCTACCTCCTATTCTACACCCTCATCAGCTCCCTGCCA : 7836
Ciconia_boyciana               : AACATTATTCTACATCTCATTCGAAGCAACTCTAATCCCCACTCTAATTCTAATCACACGATGAGGGAACCAACCAGAACGACTCAGTGCTGGCATCTACCTCCTATTCTACACCCTCATCAGCTCCCTGCCA : 7836
Iguana_iguana                  : TACATTATTCTACATTATATTTGAAGCAACGCTTATTCCAACACTAATTCTCATCACCCGATGAGGCAACCAAGCAGAACGCCTTAATGCAGGAACCTACTTTCTCTTCTACACCTTAGCAAGCTCCCTACCA : 7821
Eumeces_egregius               : TACAATATTTTACATTATATTTGAGACAACATTAATTCCAACACTCATTATCATCACGCGGTGGGGAAACCAAGCGGAACGTCTTTCAGCCGGAATTTATTTTCTATTTTACACCCTGGCCGGGTCACTCCCC : 7821
Sphenodon_punctatus            : GGCACTGTTTTATATCATATTCGAAGCAACGCTTATCCCCACATTAATTATTATCACCCGATGAGGAAACCAACCAGAACGCCTTAATGCCGGAATATATTTCTTATTTTACACACTCACAGCCTCTCTTCCG : 7830
Sceloporus_occidentalis        : AACCTTATTTTATATTATATTTGAAGCAACCTTAGTTCCAACCCTAATTATTATTACTCGATGGGGAAACCAAGCAGAACGACTAACAGCAGGGACATACTTTTTATTTTACACCTTAGCAAGCTCCCTCCCC : 7821
Cordylus_warreni               : AACCCTATTCTACATCCTATTTGAAACCACCCTAATCCCAACCCTCATCCTCATTACCCGCTGAGGCGGACAAGCTGAACGCCTAACAGCCGGAATTTACTTCCTATTTTACACCCTTTTAGGCTCTATACCC : 7824
Abronia_graminea               : TACGCTATTTTATGTTATATTCGAAGCAACACTGGTCCCCACATTAATTATCATCACACGATGAGGCCATCAGACAGAACGACTAAGCGCAGGAATTTACTTCCTATTCTATACTATTGCAAGCTCTCTTCCA : 7824
Shinisaurus_crocodilurus       : AATAATGTTCTACGTAATATTTGAAGCAACCCTAATCCCCACCCTCATCATTATTACACGATGGGGCGCCCAAGCAGAACGCCTAAACGCAGGCACATATTTTCTGCTTTATACCCTAACCAGCTCCCTCCCC : 7812
Varanus_komodoensis            : TATGCTTCTCTACATTTCATTTGAAGCAACACTCATCCCAACCCTCATTATCATCACTCGATGGGGGTCCCAAGCAGAGCGACTCAAAGCAGGAACATACTTCATATTCTACACCCTAGCAAGCTCCCTTCCC : 7866
Rhineura_floridana             : TATAATATTTTATATCATATTTGAAGCAACACTAGTGCCAACCCTAGTAGTAATCACCCGCTGAGGTGCACAAATAGAACGCCTAACCGCAGGAACTTATTTTCTATTCTACACCCTGGCCAGCTCACTCCCC : 7803
Geocalamus_acutus              : AACAATATTTTATGTTATATTTGAGACAACACTTATTCCAACCCTACTTTTAATTACCCGCTGAGGGGCACAACAAGAACGGCTTATTGCAGGCTCATACTTTATTTTTTATACCCTGACTAGCTCACTGCCA : 7797
Diplometopon_zarudnyi          : TATTATATTCTATATCATGTTTGAAACAACAATTATCCCCACACTAATACTGATCACACGCTGAGGCTCCCAACAAGAACGCCTTGCAGCCGGAACCTACTTTCTTTTTTATACACTAATCAGCTCACTACCT : 7809
Amphisbaena_schmidti           : AACAATATTTTATATTATGTTTGAAACTACCCTAATCCCGACCCTCATACTAATCACTCGCTGAGGCTCACAACACGAACGCCTTGCCGCAGGATCTTACTTTATTTTTTATACACTAATTAGCTCACTCCCC : 7806
Bipes_tridactylus              : TACTATATTCTACATCGCATTTGAGACCACACTACCACCAACTCTAATACTAATTACACGATGAGGTGCGCAAACAGACCGCCTAGAAGCCGGCACATACTTCATTTTCTACACTCTTGCTGCATCCCTACCT : 7797
Bipes_canaliculatus            : AACAATATTCTACATTGCCTTCGAAGCCACCTTACTGCCAACCCTAATACTAATCACACGCTGAGGGGCCCAAGCAGACCGACTAGAAGCGGGCTCATATTTCATCTTCTACACACTTGTTTCATCCTTACCA : 7809
Bipes_biporus                  : AATAATATTCTACATTGCCTTTGAGACCACCTTACTTCCAACCATAGTACTAATCACCCGATGAGGTGCCCAAGCAGACCGCCTAGAAGCGGGCTCATACTTCATCTTTTACACACTAGCCTCATCCCTGCCT : 7851
Anolis_carolinensis            : AATTTTGTTTTATATTATGTTTGAGGCCACCTTAATTCCAACCCTAGTGGTAATTACCCGGTGGGGAAATCAGGCCGAACGACTAACGGCTGGTACTTACTTTTTATTCTACACCCTGGCAAGCTCCTTACCC : 7490
Ophisaurus_attnuatus           : AACCATACTCTATATCATATTCGAGGCTACACTAATTCCAACACTAATTGTAATCACACGATGGGGCCACCAAGCAGAGCGCCTAAGCGCAGGAACCTACTTCCTATTTTACACCCTAGCAGGCTCCCTCCCC : 7445
Varanus_salvator               : CATCCTATTTTATATCTCGTT?GAAGCAACATTGATCCCTACTCTTACCATCATCACCCGATGAGGCTCCCAAGCAGAACGCCTCAAAGCAGGAACATACTTTATATTCTATACCTTAGCAAGCTCCCTCCCC : 7857
Mertensiella_luschani          : AATCCTTTTTTATATCTCCTTTGAAGCAACCTTAATTCCAACTTTAATTATTATTACCCGATGAGGTAATCAAACAGAGCGACTAAATGCTGGTACATACTTCCTATTCTACACCCTTGCAGGATCATTCCCC : 7839
Xenopus_laevis                 : AATTTTATTTTATATTATATTTGAAATTACATTAATCCCAACATTAATTATTATTACACGTTGAGGAAACCAAGCAGAACGCTTAAATGCAGGTACTTACTTTTTATTTTATACTCTAGCAGGTTCCTTACCT : 7824
```

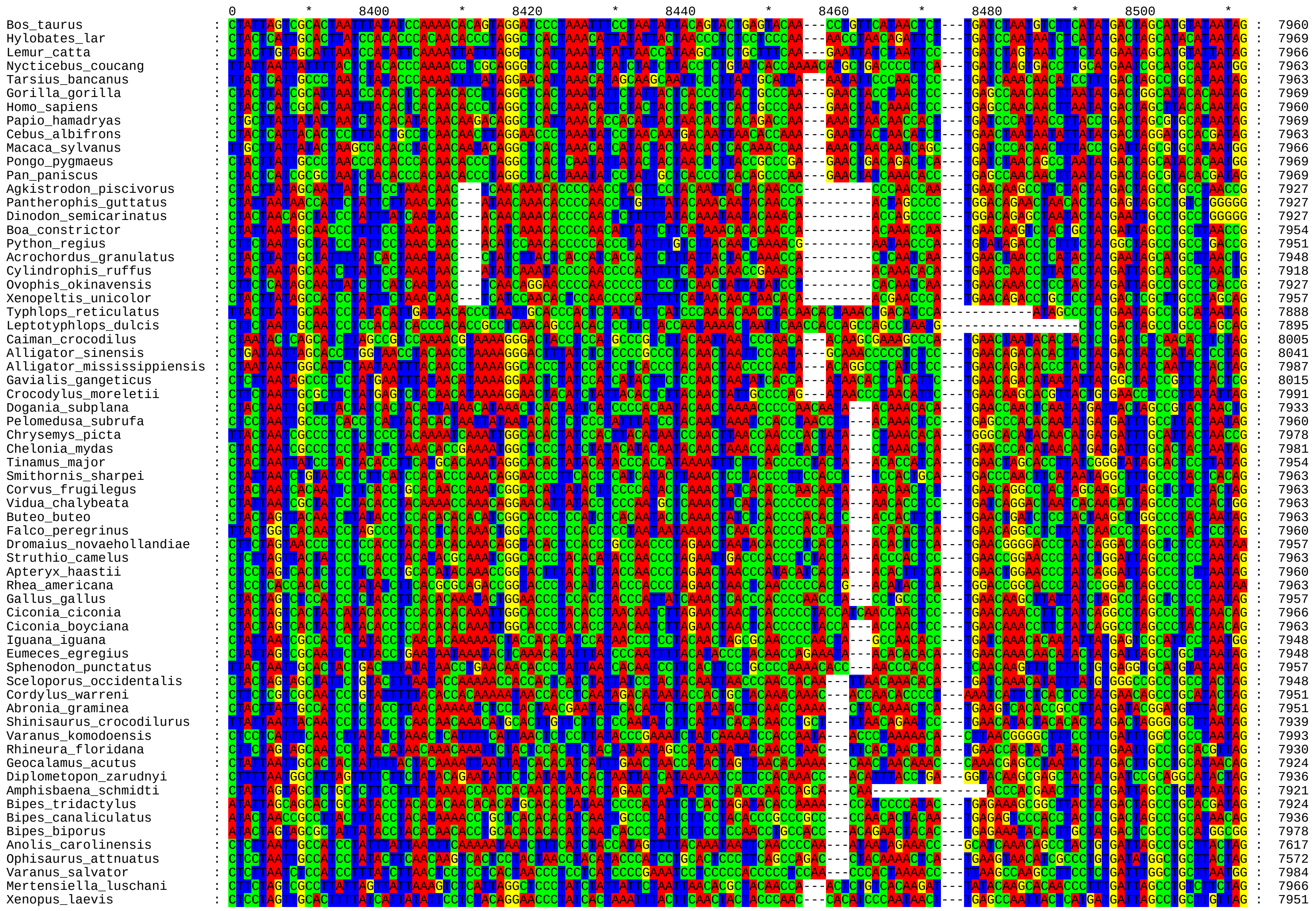
0 * 8400 * 8420 * 8440 * 8460 * 8480 * 8500 *
Bos_taurus 7960
Hylobates_lar 7969
Lemur_catta 7966
Nycticebus_coucang 7963
Tarsius_bancanus 7963
Gorilla_gorilla 7969
Homo_sapiens 7960
Papio_hamadryas 7969
Cebus_albifrons 7963
Macaca_sylvanus 7966
Pongo_pygmaeus 7969
Pan_paniscus 7969
Agkistrodon_piscivorus 7927
Pantherophis_guttatus 7927
Dinodon_semicarinatus 7927
Boa_constrictor 7954
Python_regius 7951
Acrochordus_granulatus 7948
Cylindrophis_ruffus 7918
Ovophis_okinavensis 7927
Xenopeltis_unicolor 7957
Typhlops_reticulatus 7888
Leptotyphlops_dulcis 7895
Caiman_crocodilus 8005
Alligator_sinensis 8041
Alligator_mississippiensis 7987
Gavialis_gangeticus 8015
Crocodylus_moreletii 7991
Dogania_subplana 7933
Pelomedusa_subrufa 7960
Chrysemys_picta 7978
Chelonia_mydas 7981
Tinamus_major 7954
Smithornis_sharpei 7963
Corvus_frugilegus 7963
Vidua_chalybeata 7963
Buteo_buteo 7963
Falco_peregrinus 7960
Dromaius_novaehollandiae 7957
Struthio_camelus 7963
Apteryx_haastii 7960
Rhea_americana 7963
Gallus_gallus 7957
Ciconia_ciconia 7966
Ciconia_boyciana 7963
Iguana_iguana 7948
Eumeces_egregius 7948
Sphenodon_punctatus 7957
Sceloporus_occidentalis 7948
Cordylus_warreni 7951
Abronia_graminea 7951
Shinisaurus_crocodilurus 7939
Varanus_komodoensis 7993
Rhineura_floridana 7930
Geocalamus_acutus 7924
Diplometopon_zarudnyi 7936
Amphisbaena_schmidti 7921
Bipes_tridactylus 7924
Bipes_canaliculatus 7936
Bipes_biporus 7978
Anolis_carolinensis 7617
Ophisaurus_attnuatus 7572
Varanus_salvator 7984
Mertensiella_luschani 7966
Xenopus_laevis 7951

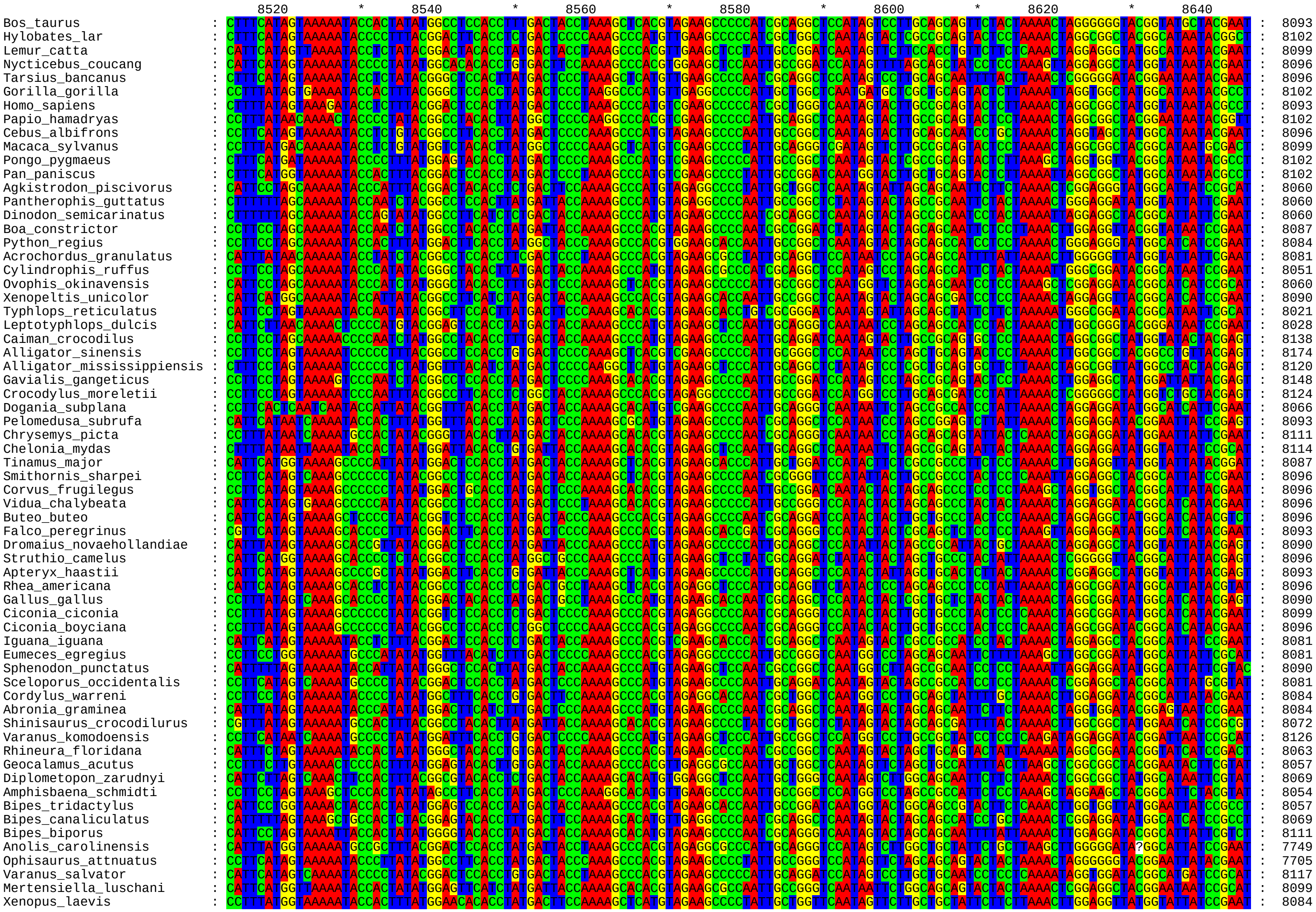
8520 * 8540 * 8560 * 8580 * 8600 * 8620 * 8640
Bos_taurus : 8093
Hylobates_lar : 8102
Lemur_catta : 8099
Nycticebus_coucang : 8096
Tarsius_bancanus : 8096
Gorilla_gorilla : 8102
Homo_sapiens : 8093
Papio_hamadryas : 8102
Cebus_albifrons : 8096
Macaca_sylvanus : 8099
Pongo_pygmaeus : 8102
Pan_paniscus : 8102
Agkistrodon_piscivorus : 8060
Pantherophis_guttatus : 8060
Dinodon_semicarinatus : 8060
Boa_constrictor : 8087
Python_regius : 8084
Acrochordus_granulatus : 8081
Cylindrophis_ruffus : 8051
Ovophis_okinavensis : 8060
Xenopeltis_unicolor : 8090
Typhlops_reticulatus : 8021
Leptotyphlops_dulcis : 8028
Caiman_crocodilus : 8138
Alligator_sinensis : 8174
Alligator_mississippiensis : 8120
Gavialis_gangeticus : 8148
Crocodylus_moreletii : 8124
Dogania_subplana : 8066
Pelomedusa_subrufa : 8093
Chrysemys_picta : 8111
Chelonia_mydas : 8114
Tinamus_major : 8087
Smithornis_sharpei : 8096
Corvus_frugilegus : 8096
Vidua_chalybeata : 8096
Buteo_buteo : 8096
Falco_peregrinus : 8093
Dromaius_novaehollandiae : 8090
Struthio_camelus : 8096
Apteryx_haastii : 8093
Rhea_americana : 8096
Gallus_gallus : 8090
Ciconia_ciconia : 8099
Ciconia_boyciana : 8096
Iguana_iguana : 8081
Eumeces_egregius : 8081
Sphenodon_punctatus : 8090
Sceloporus_occidentalis : 8081
Cordylus_warreni : 8084
Abronia_graminea : 8084
Shinisaurus_crocodilurus : 8072
Varanus_komodoensis : 8126
Rhineura_floridana : 8063
Geocalamus_acutus : 8057
Diplometopon_zarudnyi : 8069
Amphisbaena_schmidti : 8054
Bipes_tridactylus : 8057
Bipes_canaliculatus : 8069
Bipes_biporus : 8111
Anolis_carolinensis : 7749
Ophisaurus_attnuatus : 7705
Varanus_salvator : 8117
Mertensiella_luschani : 8099
Xenopus_laevis : 8084

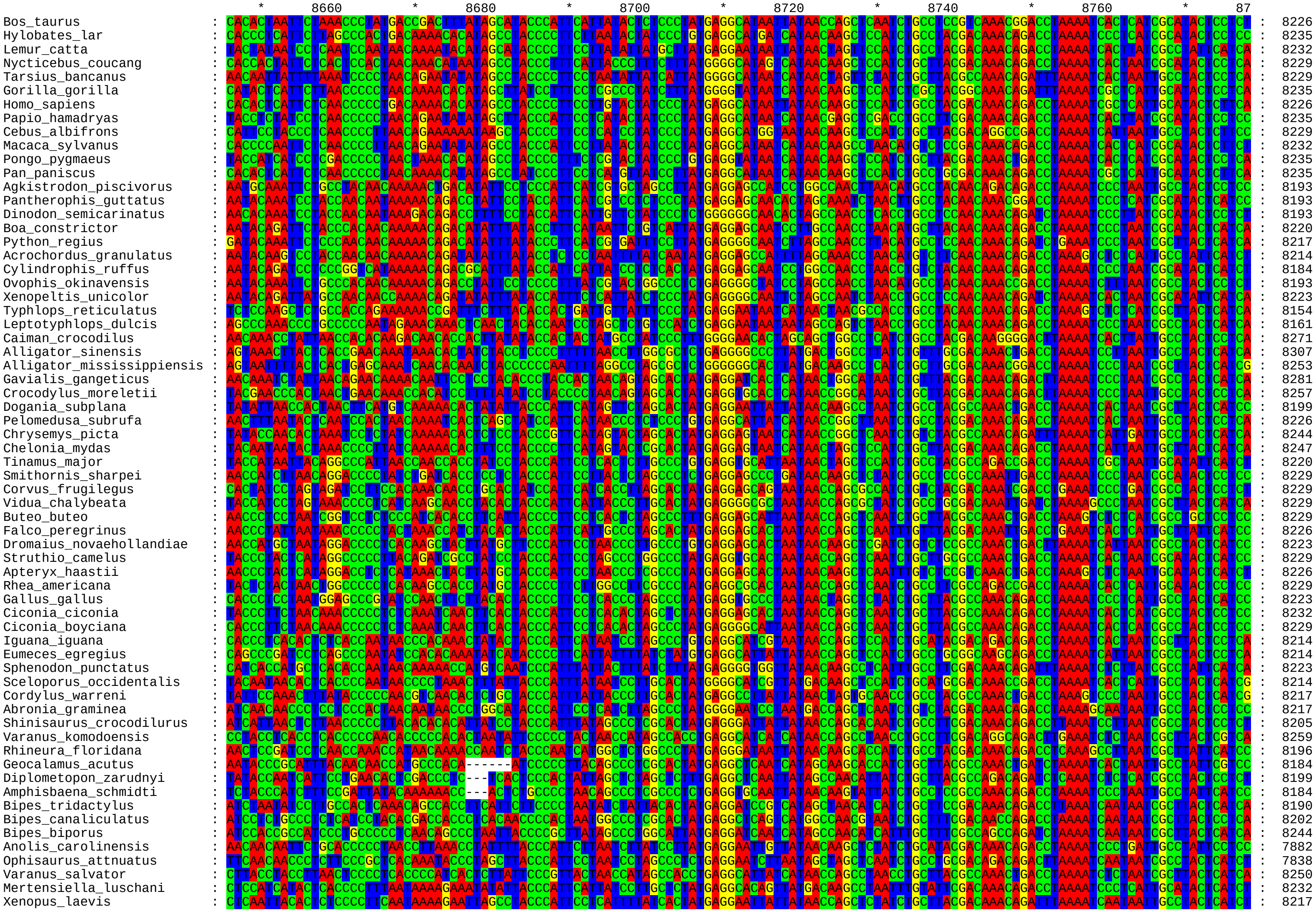
* 8660 * 8680 * 8700 * 8720 * 8740 * 8760 * 87
Bos_taurus : 8226
Hylobates_lar : 8235
Lemur_catta : 8232
Nycticebus_coucang : 8229
Tarsius_bancanus : 8229
Gorilla_gorilla : 8235
Homo_sapiens : 8226
Papio_hamadryas : 8235
Cebus_albifrons : 8229
Macaca_sylvanus : 8232
Pongo_pygmaeus : 8235
Pan_paniscus : 8235
Agkistrodon_piscivorus : 8193
Pantherophis_guttatus : 8193
Dinodon_semicarinatus : 8193
Boa_constrictor : 8220
Python_regius : 8217
Acrochordus_granulatus : 8214
Cylindrophis_ruffus : 8184
Ovophis_okinavensis : 8193
Xenopeltis_unicolor : 8223
Typhlops_reticulatus : 8154
Leptotyphlops_dulcis : 8161
Caiman_crocodilus : 8271
Alligator_sinensis : 8307
Alligator_mississippiensis : 8253
Gavialis_gangeticus : 8281
Crocodylus_moreletii : 8257
Dogania_subplana : 8199
Pelomedusa_subrufa : 8226
Chrysemys_picta : 8244
Chelonia_mydas : 8247
Tinamus_major : 8220
Smithornis_sharpei : 8229
Corvus_frugilegus : 8229
Vidua_chalybeata : 8229
Buteo_buteo : 8229
Falco_peregrinus : 8226
Dromaius_novaehollandiae : 8223
Struthio_camelus : 8229
Apteryx_haastii : 8226
Rhea_americana : 8229
Gallus_gallus : 8223
Ciconia_ciconia : 8232
Ciconia_boyciana : 8229
Iguana_iguana : 8214
Eumeces_egregius : 8214
Sphenodon_punctatus : 8223
Sceloporus_occidentalis : 8214
Cordylus_warreni : 8217
Abronia_graminea : 8217
Shinisaurus_crocodilurus : 8205
Varanus_komodoensis : 8259
Rhineura_floridana : 8196
Geocalamus_acutus : 8184
Diplometopon_zarudnyi : 8199
Amphisbaena_schmidti : 8184
Bipes_tridactylus : 8190
Bipes_canaliculatus : 8202
Bipes_biporus : 8244
Anolis_carolinensis : 7882
Ophisaurus_attnuatus : 7838
Varanus_salvator : 8250
Mertensiella_luschani : 8232
Xenopus_laevis : 8217

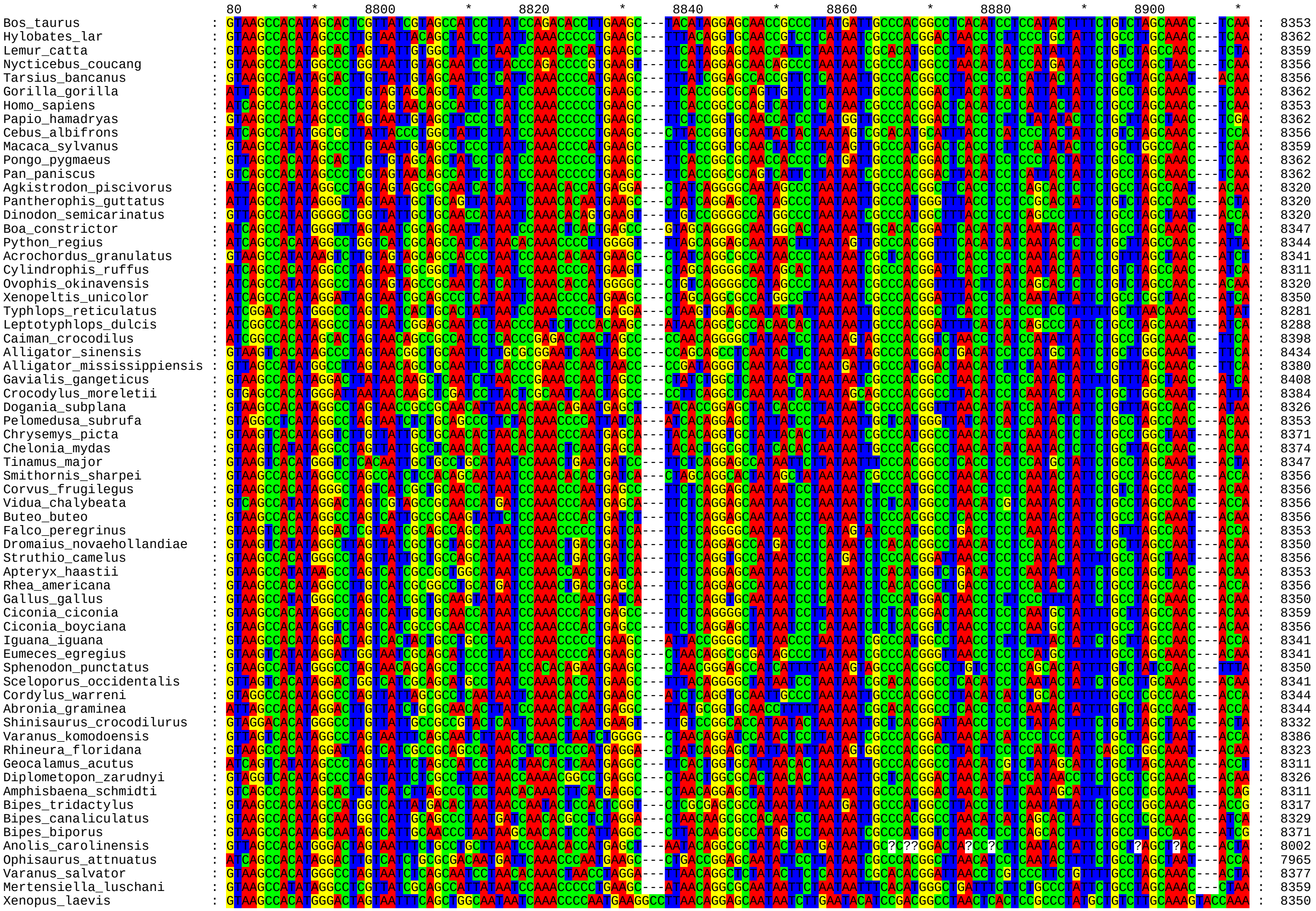
80 * 8800 * 8820 * 8840 * 8860 * 8880 * 8900 *
Bos_taurus 8353
Hylobates_lar 8362
Lemur_catta 8359
Nycticebus_coucang 8356
Tarsius_bancanus 8356
Gorilla_gorilla 8362
Homo_sapiens 8353
Papio_hamadryas 8362
Cebus_albifrons 8356
Macaca_sylvanus 8359
Pongo_pygmaeus 8362
Pan_paniscus 8362
Agkistrodon_piscivorus 8320
Pantherophis_guttatus 8320
Dinodon_semicarinatus 8320
Boa_constrictor 8347
Python_regius 8344
Acrochordus_granulatus 8341
Cylindrophis_ruffus 8311
Ovophis_okinavensis 8320
Xenopeltis_unicolor 8350
Typhlops_reticulatus 8281
Leptotyphlops_dulcis 8288
Caiman_crocodilus 8398
Alligator_sinensis 8434
Alligator_mississippiensis 8380
Gavialis_gangeticus 8408
Crocodylus_moreletii 8384
Dogania_subplana 8326
Pelomedusa_subrufa 8353
Chrysemys_picta 8371
Chelonia_mydas 8374
Tinamus_major 8347
Smithornis_sharpei 8356
Corvus_frugilegus 8356
Vidua_chalybeata 8356
Buteo_buteo 8356
Falco_peregrinus 8353
Dromaius_novaehollandiae 8350
Struthio_camelus 8356
Apteryx_haastii 8353
Rhea_americana 8356
Gallus_gallus 8350
Ciconia_ciconia 8359
Ciconia_boyciana 8356
Iguana_iguana 8341
Eumeces_egregius 8341
Sphenodon_punctatus 8350
Sceloporus_occidentalis 8341
Cordylus_warreni 8344
Abronia_graminea 8344
Shinisaurus_crocodilurus 8332
Varanus_komodoensis 8386
Rhineura_floridana 8323
Geocalamus_acutus 8311
Diplometopon_zarudnyi 8326
Amphisbaena_schmidti 8311
Bipes_tridactylus 8317
Bipes_canaliculatus 8329
Bipes_biporus 8371
Anolis_carolinensis 8002
Ophisaurus_attnuatus 7965
Varanus_salvator 8377
Mertensiella_luschani 8359
Xenopus_laevis 8350

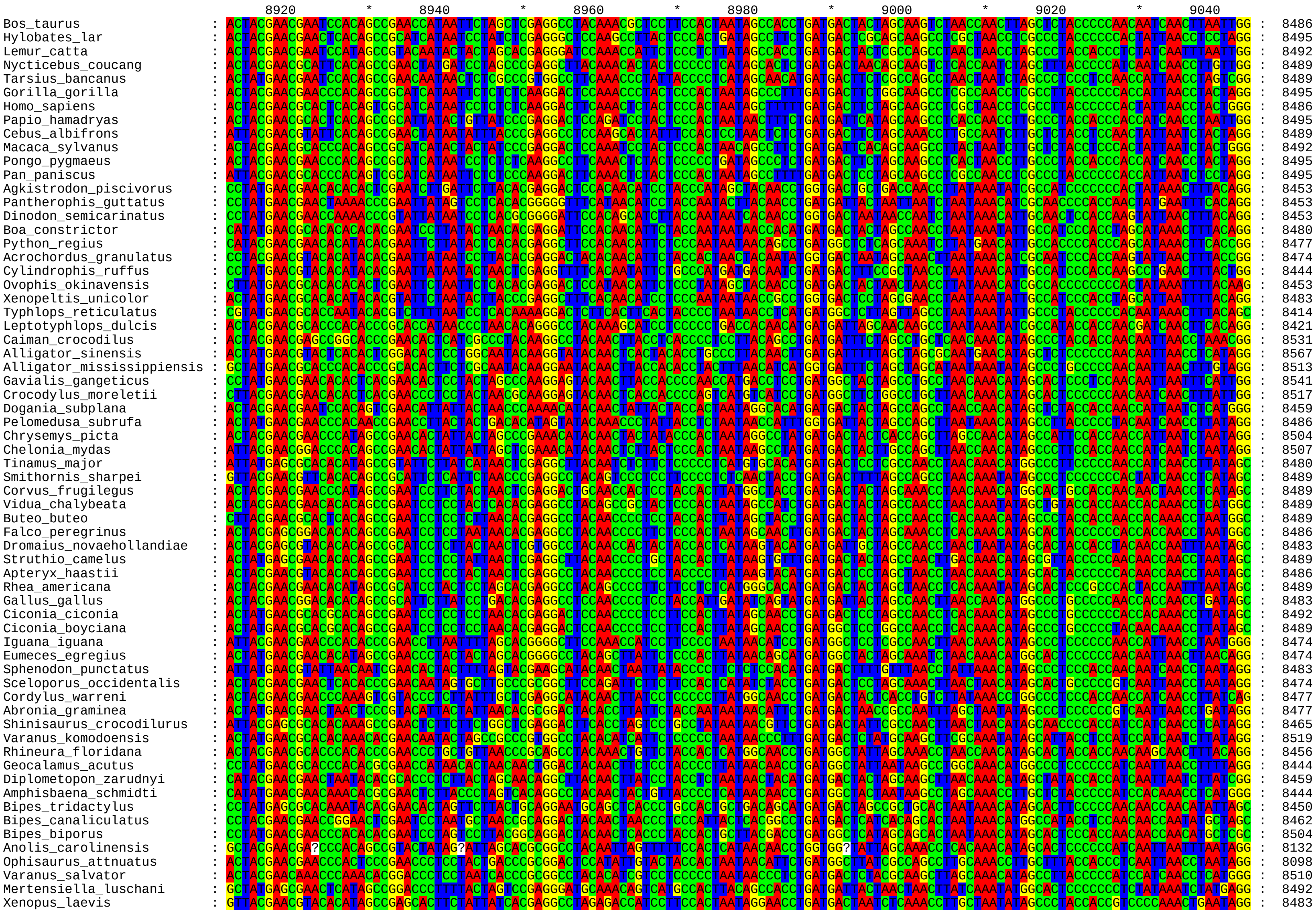

8920 * 8940 * 8960 * 8980 * 9000 * 9020 * 9040
Bos_taurus : 8486
Hylobates_lar : 8495
Lemur_catta : 8492
Nycticebus_coucang : 8489
Tarsius_bancanus : 8489
Gorilla_gorilla : 8495
Homo_sapiens : 8486
Papio_hamadryas : 8495
Cebus_albifrons : 8489
Macaca_sylvanus : 8492
Pongo_pygmaeus : 8495
Pan_paniscus : 8495
Agkistrodon_piscivorus : 8453
Pantherophis_guttatus : 8453
Dinodon_semicarinatus : 8453
Boa_constrictor : 8480
Python_regius : 8477
Acrochordus_granulatus : 8474
Cylindrophis_ruffus : 8444
Ovophis_okinavensis : 8453
Xenopeltis_unicolor : 8483
Typhlops_reticulatus : 8414
Leptotyphlops_dulcis : 8421
Caiman_crocodilus : 8531
Alligator_sinensis : 8567
Alligator_mississippiensis : 8513
Gavialis_gangeticus : 8541
Crocodylus_moreletii : 8517
Dogania_subplana : 8459
Pelomedusa_subrufa : 8486
Chrysemys_picta : 8504
Chelonia_mydas : 8507
Tinamus_major : 8480
Smithornis_sharpei : 8489
Corvus_frugilegus : 8489
Vidua_chalybeata : 8489
Buteo_buteo : 8489
Falco_peregrinus : 8486
Dromaius_novaehollandiae : 8483
Struthio_camelus : 8489
Apteryx_haastii : 8486
Rhea_americana : 8489
Gallus_gallus : 8483
Ciconia_ciconia : 8492
Ciconia_boyciana : 8489
Iguana_iguana : 8474
Eumeces_egregius : 8474
Sphenodon_punctatus : 8483
Sceloporus_occidentalis : 8474
Cordylus_warreni : 8477
Abronia_graminea : 8477
Shinisaurus_crocodilurus : 8465
Varanus_komodoensis : 8519
Rhineura_floridana : 8456
Geocalamus_acutus : 8444
Diplometopon_zarudnyi : 8459
Amphisbaena_schmidti : 8444
Bipes_tridactylus : 8450
Bipes_canaliculatus : 8462
Bipes_biporus : 8504
Anolis_carolinensis : 8132
Ophisaurus_attnuatus : 8098
Varanus_salvator : 8510
Mertensiella_luschani : 8492
Xenopus_laevis : 8483

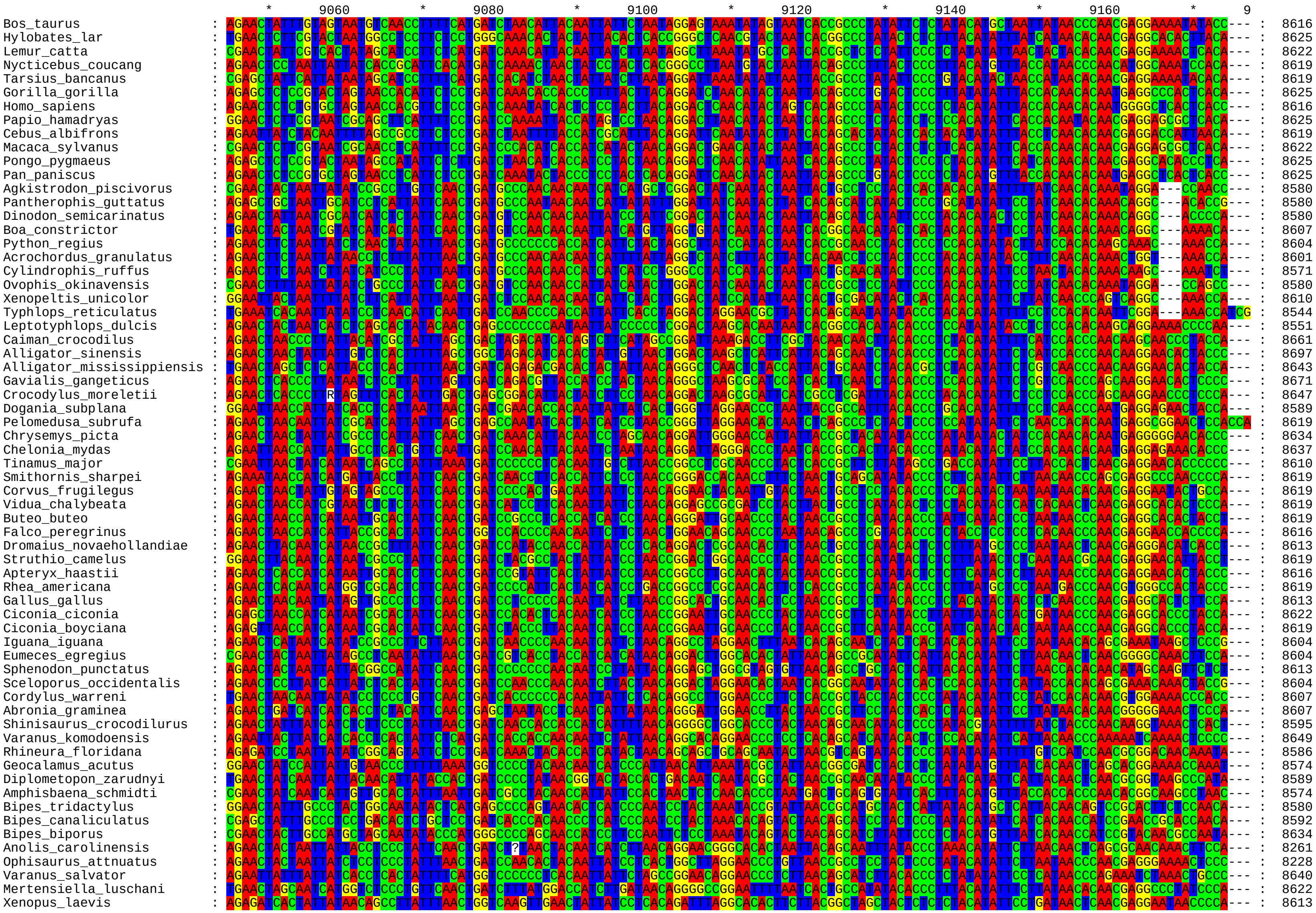

9060
9080
9100
9120
9140
9160
Bos_taurus : 8616
Hylobates_lar : 8625
Lemur_catta : 8622
Nycticebus_coucang : 8619
Tarsius_bancanus : 8619
Gorilla_gorilla : 8625
Homo_sapiens : 8616
Papio_hamadryas : 8625
Cebus_albifrons : 8619
Macaca_sylvanus : 8622
Pongo_pygmaeus : 8625
Pan_paniscus : 8625
Agkistrodon_piscivorus : 8580
Pantherophis_guttatus : 8580
Dinodon_semicarinatus : 8580
Boa_constrictor : 8607
Python_regius : 8604
Acrochordus_granulatus : 8601
Cylindrophis_ruffus : 8571
Ovophis_okinavensis : 8580
Xenopeltis_unicolor : 8610
Typhlops_reticulatus : 8544
Leptotyphlops_dulcis : 8551
Caiman_crocodilus : 8661
Alligator_sinensis : 8697
Alligator_mississippiensis : 8643
Gavialis_gangeticus : 8671
Crocodylus_moreletii : 8647
Dogania_subplana : 8589
Pelomedusa_subrufa : 8619
Chrysemys_picta : 8634
Chelonia_mydas : 8637
Tinamus_major : 8610
Smithornis_sharpei : 8619
Corvus_frugilegus : 8619
Vidua_chalybeata : 8619
Buteo_buteo : 8619
Falco_peregrinus : 8616
Dromaius_novaehollandiae : 8613
Struthio_camelus : 8619
Apteryx_haastii : 8616
Rhea_americana : 8619
Gallus_gallus : 8613
Ciconia_ciconia : 8622
Ciconia_boyciana : 8619
Iguana_iguana : 8604
Eumeces_egregius : 8604
Sphenodon_punctatus : 8613
Sceloporus_occidentalis : 8604
Cordylus_warreni : 8607
Abronia_graminea : 8607
Shinisaurus_crocodilurus : 8595
Varanus_komodoensis : 8649
Rhineura_floridana : 8586
Geocalamus_acutus : 8574
Diplometopon_zarudnyi : 8589
Amphisbaena_schmidti : 8574
Bipes_tridactylus : 8580
Bipes_canaliculatus : 8592
Bipes_biporus : 8634
Anolis_carolinensis : 8261
Ophisaurus_attnuatus : 8228
Varanus_salvator : 8640
Mertensiella_luschani : 8622
Xenopus_laevis : 8613

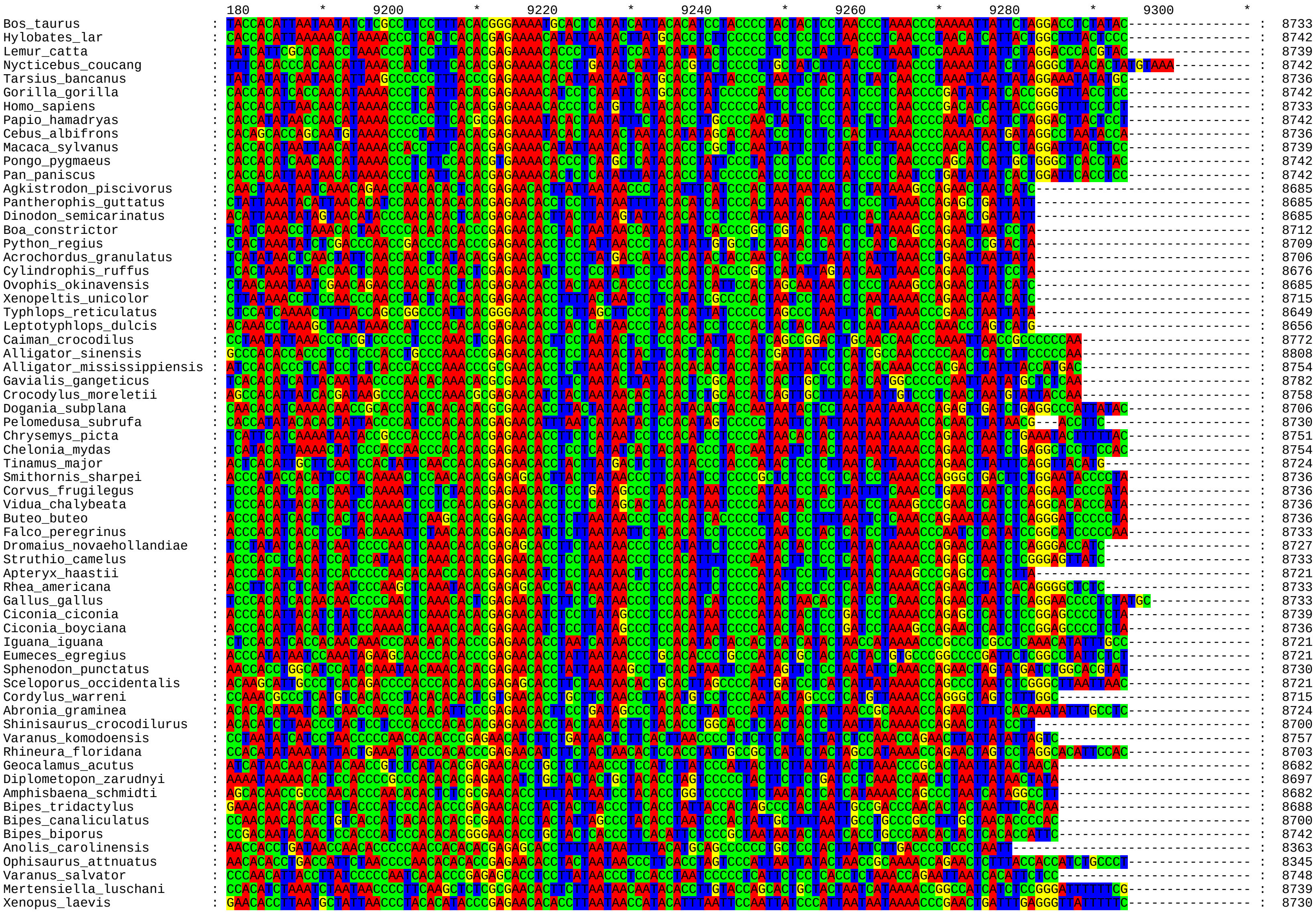
180 * 9200 * 9220 * 9240 * 9260 * 9280 * 9300 *
Bos_taurus : 8733
Hylobates_lar : 8742
Lemur_catta : 8739
Nycticebus_coucang : 8742
Tarsius_bancanus : 8736
Gorilla_gorilla : 8742
Homo_sapiens : 8733
Papio_hamadryas : 8742
Cebus_albifrons : 8736
Macaca_sylvanus : 8739
Pongo_pygmaeus : 8742
Pan_paniscus : 8742
Agkistrodon_piscivorus : 8685
Pantherophis_guttatus : 8685
Dinodon_semicarinatus : 8685
Boa_constrictor : 8712
Python_regius : 8709
Acrochordus_granulatus : 8706
Cylindrophis_ruffus : 8676
Ovophis_okinavensis : 8685
Xenopeltis_unicolor : 8715
Typhlops_reticulatus : 8649
Leptotyphlops_dulcis : 8656
Caiman_crocodilus : 8772
Alligator_sinensis : 8808
Alligator_mississippiensis : 8754
Gavialis_gangeticus : 8782
Crocodylus_moreletii : 8758
Dogania_subplana : 8706
Pelomedusa_subrufa : 8730
Chrysemys_picta : 8751
Chelonia_mydas : 8754
Tinamus_major : 8724
Smithornis_sharpei : 8736
Corvus_frugilegus : 8736
Vidua_chalybeata : 8736
Buteo_buteo : 8736
Falco_peregrinus : 8733
Dromaius_novaehollandiae : 8727
Struthio_camelus : 8733
Apteryx_haastii : 8721
Rhea_americana : 8733
Gallus_gallus : 8733
Ciconia_ciconia : 8739
Ciconia_boyciana : 8736
Iguana_iguana : 8721
Eumeces_egregius : 8721
Sphenodon_punctatus : 8730
Sceloporus_occidentalis : 8721
Cordylus_warreni : 8715
Abronia_graminea : 8724
Shinisaurus_crocodilurus : 8700
Varanus_komodoensis : 8757
Rhineura_floridana : 8703
Geocalamus_acutus : 8682
Diplometopon_zarudnyi : 8697
Amphisbaena_schmidti : 8682
Bipes_tridactylus : 8688
Bipes_canaliculatus : 8700
Bipes_biporus : 8742
Anolis_carolinensis : 8363
Ophisaurus_attnuatus : 8345
Varanus_salvator : 8748
Mertensiella_luschani : 8739
Xenopus_laevis : 8730

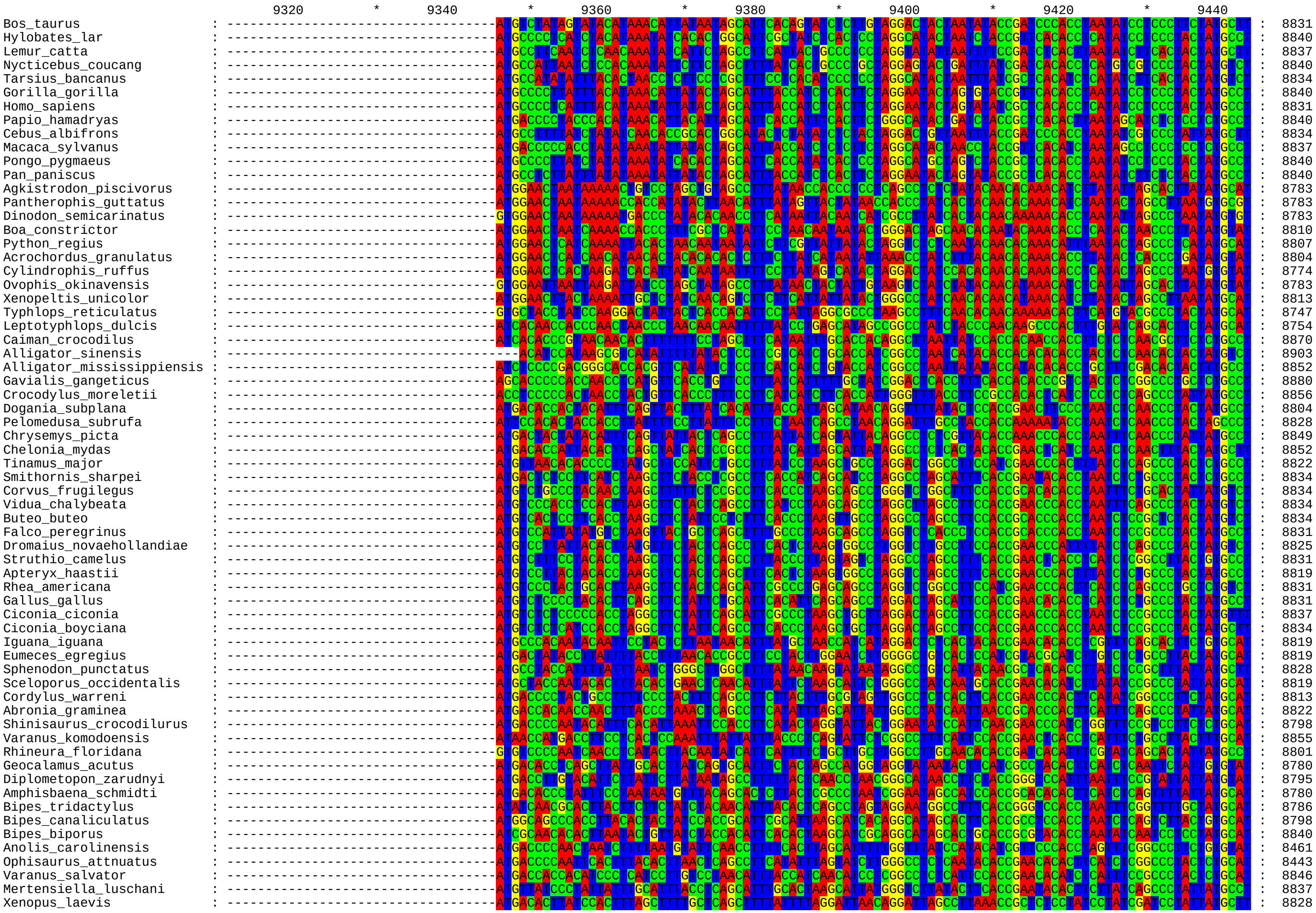
9320 * 9340 * 9360 * 9380 * 9400 * 9420 * 9440
Bos_taurus : 8831
Hylobates_lar : 8840
Lemur_catta : 8837
Nycticebus_coucang : 8840
Tarsius_bancanus : 8834
Gorilla_gorilla : 8840
Homo_sapiens : 8831
Papio_hamadryas : 8840
Cebus_albifrons : 8834
Macaca_sylvanus : 8837
Pongo_pygmaeus : 8840
Pan_paniscus : 8840
Agkistrodon_piscivorus : 8783
Pantherophis_guttatus : 8783
Dinodon_semicarinatus : 8783
Boa_constrictor : 8810
Python_regius : 8807
Acrochordus_granulatus : 8804
Cylindrophis_ruffus : 8774
Ovophis_okinavensis : 8783
Xenopeltis_unicolor : 8813
Typhlops_reticulatus : 8747
Leptotyphlops_dulcis : 8754
Caiman_crocodilus : 8870
Alligator_sinensis : 8903
Alligator_mississippiensis : 8852
Gavialis_gangeticus : 8880
Crocodylus_moreletii : 8856
Dogania_subplana : 8804
Pelomedusa_subrufa : 8828
Chrysemys_picta : 8849
Chelonia_mydas : 8852
Tinamus_major : 8822
Smithornis_sharpei : 8834
Corvus_frugilegus : 8834
Vidua_chalybeata : 8834
Buteo_buteo : 8834
Falco_peregrinus : 8831
Dromaius_novaehollandiae : 8825
Struthio_camelus : 8831
Apteryx_haastii : 8819
Rhea_americana : 8831
Gallus_gallus : 8831
Ciconia_ciconia : 8837
Ciconia_boyciana : 8834
Iguana_iguana : 8819
Eumeces_egregius : 8819
Sphenodon_punctatus : 8828
Sceloporus_occidentalis : 8819
Cordylus_warreni : 8813
Abronia_graminea : 8822
Shinisaurus_crocodilurus : 8798
Varanus_komodoensis : 8855
Rhineura_floridana : 8801
Geocalamus_acutus : 8780
Diplometopon_zarudnyi : 8795
Amphisbaena_schmidti : 8780
Bipes_tridactylus : 8786
Bipes_canaliculatus : 8798
Bipes_biporus : 8840
Anolis_carolinensis : 8461
Ophisaurus_attnuatus : 8443
Varanus_salvator : 8846
Mertensiella_luschani : 8837
Xenopus_laevis : 8828

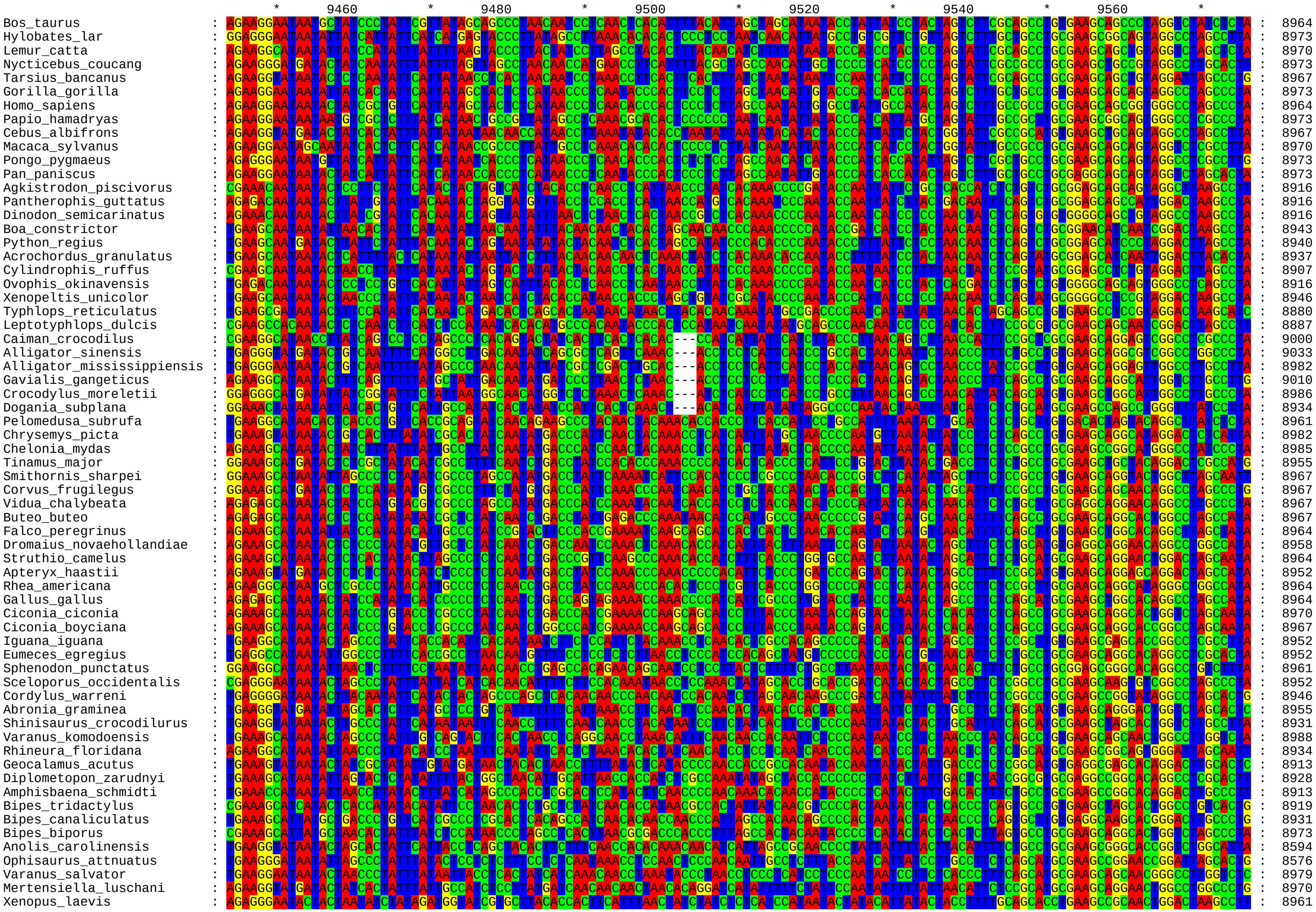
9460 9480 9500 9520 9540 9560
Bos_taurus : 8964
Hylobates_lar : 8973
Lemur_catta : 8970
Nycticebus_coucang : 8973
Tarsius_bancanus : 8967
Gorilla_gorilla : 8973
Homo_sapiens : 8964
Papio_hamadryas : 8973
Cebus_albifrons : 8967
Macaca_sylvanus : 8970
Pongo_pygmaeus : 8973
Pan_paniscus : 8973
Agkistrodon_piscivorus : 8916
Pantherophis_guttatus : 8916
Dinodon_semicarinatus : 8916
Boa_constrictor : 8943
Python_regius : 8940
Acrochordus_granulatus : 8937
Cylindrophis_ruffus : 8907
Ovophis_okinavensis : 8916
Xenopeltis_unicolor : 8946
Typhlops_reticulatus : 8880
Leptotyphlops_dulcis : 8887
Caiman_crocodilus : 9000
Alligator_sinensis : 9033
Alligator_mississippiensis : 8982
Gavialis_gangeticus : 9010
Crocodylus_moreletii : 8986
Dogania_subplana : 8934
Pelomedusa_subrufa : 8961
Chrysemys_picta : 8982
Chelonia_mydas : 8985
Tinamus_major : 8955
Smithornis_sharpei : 8967
Corvus_frugilegus : 8967
Vidua_chalybeata : 8967
Buteo_buteo : 8967
Falco_peregrinus : 8964
Dromaius_novaehollandiae : 8958
Struthio_camelus : 8964
Apteryx_haastii : 8952
Rhea_americana : 8964
Gallus_gallus : 8964
Ciconia_ciconia : 8970
Ciconia_boyciana : 8967
Iguana_iguana : 8952
Eumeces_egregius : 8952
Sphenodon_punctatus : 8961
Sceloporus_occidentalis : 8952
Cordylus_warreni : 8946
Abronia_graminea : 8955
Shinisaurus_crocodilurus : 8931
Varanus_komodoensis : 8988
Rhineura_floridana : 8934
Geocalamus_acutus : 8913
Diplometopon_zarudnyi : 8928
Amphisbaena_schmidti : 8913
Bipes_tridactylus : 8919
Bipes_canaliculatus : 8931
Bipes_biporus : 8973
Anolis_carolinensis : 8594
Ophisaurus_attnuatus : 8576
Varanus_salvator : 8979
Mertensiella_luschani : 8970
Xenopus_laevis : 8961

```
                             9580         *      9600         *      9620         *      9640         *      9660         *      9680         *      9700
Bos_taurus                 : CTAGTAATAGTATCAAATACATATGGTACTGATTATGTACAAAACCTCAACTTACTCCAATGC---------------ATAAAC---ATATTCTCCTCACTCTCACTAGTTACTTTACTCTTACTAACTATAC : 9079
Hylobates_lar              : CTAGTCTCAATCTCTAACACATACGGCCTAGACCACATCCACAACCTAAACCTACTCCAATGC---------------ATAGCA---ATGTACACCACCATAGCCATTCTAACGCTAACCTCCCTAATTCCCC : 9088
Lemur_catta                : CTAGTTACAGTATCCAACACCTACGGCCTAGATTATATCCAAAATCTAAATCTCCTCCAATGC---------------ATAAAT---CTATTATCCTCTTTCACCCTTGTCACACTGATTATCCTAACTTTAC : 9085
Nycticebus_coucang         : CTAGTAATGGTATCCAACACCTACGGCATAGACTACATCCAGAACCTAAACCTCCTGCAATGC---------------ATTAACACACTACTACCTACAATGGCTACCACTTCATTAATCATTCTAACTTTGC : 9091
Tarsius_bancanus           : CTAGTAATAGTATCAAACACATATGGCCTAGACTATGTACAAAACCTAAACCTTCTCCAATGC---------------ATAAAT---TTATTTACACCCTCCATCTTACTCTCACTCATACTATTAATCATCC : 9082
Gorilla_gorilla            : CTAGTCTCAATCTCTAACACATATGGTCTTGACTATGTACAAAACCTAAACCTGCTCCAATGC---------------ATAACT---ATGTACGCTACCATAACCACCTTAGCCCTAACTTCCTTAATTCCCC : 9088
Homo_sapiens               : CTAGTCTCAATCTCCAACACATATGGCCTAGACTACGTACATAACCTAAACCTACTCCAATGC---------------ATAACC---ATGCACACTACTATAACCACCCTAACCCTGACTTCCCTAATTCCCC : 9079
Papio_hamadryas            : CTAGTTTCAATCTCCAACACATATGGACTAGATCACATCAACAACCTGAGCCTGCTCCAATGT---------------ATAGCC---ATGTACACTTCCATTATACTAATAACCCTCGCCTCCCTAGCCCTTC : 9088
Cebus_albifrons            : TTAATTTTAATCTCCAACTTATATGGCTTAGACTACGTACAAAACCTAAACTTACTCCAATGC------------------------ATGCACTTTTCTATTACTCTAATAACACTAATTCCCTTACTAGTAC : 9076
Macaca_sylvanus            : CTAGTCTCAATCTCCAACACATACGGACTAGATTACATCCACAACCTGAACCTGCTTCAATGC---------------ATAATC---ATGTATACCCCCATCATAATAACAACTCTCATCTCCCTAACTCTTC : 9085
Pongo_pygmaeus             : CTAGCCTCAATCTCCAACACATACGGCCTAGACTACGTCAACAACCTAAACCTGCTTCAATGC---------------ACAGCC---ATGTTCACCACCATAACCGCCCTCACCTTAACTTCCCTAATCCCCC : 9088
Pan_paniscus               : TTAGTTTCAATCTCTAACACGTATGGCCTAGACTACGTACATAATCTAAACCTACTCCAATGC---------------ATAACC---ATGTATACTACCATAACCACCTTAACCCTAACTCCCTTAATTCTCC : 9088
Agkistrodon_piscivorus     : GTTGTTGCAATCACACGAACCCACGGAAACGACTTCCTAAAAAACTTAAACCTCCTA---------------------ATGGGCCTAACAACACCAACTATCATCCTGACAGTCTTCCTCTCACTAACCGCTT : 9028
Pantherophis_guttatus      : GTAGTTGCAATTACACGAACTCACGGAAATGACTTCTTAAAAAACTTAAATCTTTTA---------------------ATGGGCTCAATCACCCCAACAATCATCATAACCATTTTCCTATCTCTTACCATAT : 9028
Dinodon_semicarinatus      : GTAGTCGCAATTACACGAACTCATGGAAACGACTTTCTAAAAAATCTAAACCTATTA---------------------ATGAACTTAATCACCCCCACAATTATCCTAACAATTATACTATCTTTAATAATAT : 9028
Boa_constrictor            : GTCGTAACCACTACTCGAATACATGGAAACGACTTCCTAAAAAATCTTAACCTTTTA---------------------ATGAATACAACACCAACATTAATAATAACCACATTTATCATCCTAATCATAACAA : 9055
Python_regius              : GTTGTTGCTACCACTCGAACACACGGCAGCGACTTTCTAAAAAATCTAAACCTCCTA---------------------ATGAATACTATCACCCCAACAATAACCCTAGCCGTATTCCTATTACTAATACTCA : 9052
Acrochordus_granulatus     : GTAGTAGCAATCACTCGAACACACGGAAACGACTTCTTAAAAAACCTAAATTTATTA---------------------GTGACCATCATTGTCCCCACAATCACTATTACACTATTCACAATACTACTAATTA : 9049
Cylindrophis_ruffus        : GTCGTAGCAACCACACGAACTCACGGTAACGATTTCCTAAAAAACCTAAACCTCCTA---------------------ATGAACACCATCACTCCCACACTAACAGTAACCATTTTTATATCTCTAACGCTCC : 9019
Ovophis_okinavensis        : GTTGTTGCAATCACACGAACTCACGGAAATGACTTTCTAAAAAACTTAAATCTTCTA---------------------ATGGACCTAATTGCACCAACCATCATCCTAGCAATCTTTTTCTCCCTAACCCTCT : 9028
Xenopeltis_unicolor        : GTAGTAGCAACAACTCGAACACATGGAAGTGATTTCCTTAAAAACCTCAATTTACTA---------------------ATGGACACTATTATCACAACACTAACAATAACAATTTTCATCTATCTCACTACTT : 9058
Typhlops_reticulatus       : CTAATTGCAACCATCCGAACCCACGGGATGCGATTTATAAGTAATCTCAACATATTA---------------------ATGACACCCATCATTATCAGCACCACAACCCTACTAATTATACTATGACTTCTTC : 8992
Leptotyphlops_dulcis       : ATAATTGCAACTATACGAACACACGGCTCCGACAACCTAGCCCTCCTAAATCTACTAAAATGC------------ATATCAACTCTAATCCCAGGAGCCATCCTACTATCAATCCCAATCATCCTAGCCATCC : 9008
Caiman_crocodilus          : CTTATCGCCTCCACCCGAACACACAACACCACAAACCTTAAAAACCTAAACCTCCTCCAATGT------ATGCCTAATCCCCCCCTCATCCTAACCACCTTTATCCTTCCC---TTTCTACTCCTACTAACA- : 9123
Alligator_sinensis         : CTGGTTGCCTCTGCTCGAACACATAACACAGCAAACCTTAAAAACCTAAACCTCCTCCAATGC------ATGCAACAATCAACCCTATTTGTTACACTATTCACCCTACCCCCCTTCATCCTCGTACTATCCT : 9160
Alligator_mississippiensis : TTAGTCGCCTCTGCTCGAACTCATAATACGGCAAGCCTCAAAAACCTAAACCTGCTACAATGC------ATGCAACAGCCGACCCTATCTCTTACCTTCTTCACCCTACCTGCTTTTATCCTTTTACTATCT- : 9108
Gavialis_gangeticus        : CTAATTGCCTCAGCCCGCACACACAACACAACCAACCTTAAAAACCTTAACCTCCTCCAATGC------ATGAATCCAGCTATCATGCTTTTCATACTACCACTAACCATTATATTATCGTCACTATTCCTT- : 9136
Crocodylus_moreletii       : CTGATTGCCTCAGCCCGAACACATAATACAGCCAACCTCAAAAACCTAAACCTACTCCAATGT------TCAGCCTTAATTATTATACTTTTCCTACTACCCCTAGCAATCTTAACACTCCCAATACTAATC- : 9112
Dogania_subplana           : TTAGTAGCATCATCACGAACACACAACCCAAACAACCTACAAAACCTTAACCTCCTACAATGC---------------ATGACAACAATTTTTAACTCAACATTTCTACTAGGACTCACAATTCTCACATTCC : 9052
Pelomedusa_subrufa         : CTAGTAGCTACATCACGAACCCATGGACTTGATATATTAAAAAACCTAAATTTACTTCAATGT------ATGCCAAACCTACAACTCATTCTAAAAACAATCCTCATCACCGAACTCCTAATCCTAGCTTTAT : 9088
Chrysemys_picta            : TTAGTAGCATCCTCACGAACTCATGGCTCAGACCACCTCCAAAACTTAAACCTTTTACAGTGC------------------ATGGCTTTAATTAACCTGTCAATTTTATTCGCCATACTTACATTAGTATCGC : 9097
Chelonia_mydas             : CTAGTAGCATCCTCACGAACCCACGGTTCAGATCAACTACAAAACTTAAATCTCTTACAATGC---------------ATGACCCTACTAATAAATTCAACTTTCTTACTAGCCTTAATCACCCTAACACTCC : 9103
Tinamus_major              : TTGGTAGCATCCACCCGAACCCACGGCTCAGACCACCTCCACAACCTAAGCCTACTGCAATGT---------ATAAAAACCTCGTTACTCCTAAATACCCTAACCCTATTAACATTAATAATCCTCTCTATAC : 9079
Smithornis_sharpei         : CTCGTCGCCTCCACACGAACCCATGGCTCAGACCACCTCATCAACTTAAACACCTTACGATGC---------ATAAACCTCCCACTCATTCTCAACACATCAATACTACTCACCCTAACCATCACCCTCACCC : 9091
Corvus_frugilegus          : CTAGTCGCCTCTACTCGGACCCACGGCTCCGACCACCTACACAACTTTAACCTACTACAATGC---------ATGGATCAAATATTTATCCTAAACACATTCATACTACTCACCCTAGCAGTCCTCTGCACCC : 9091
Vidua_chalybeata           : CTAGTAGCCTCTACCCGAACCCACGGCTCCGATTACCTGCACAACTTCAACCTACTACGATGC---------ATGGACCTATCACTAGTCCTAAACACATTCATACTCCTCACCCTAGTCACTCTATCCACTC : 9091
Buteo_buteo                : CTAGTTGCCTCCACACGTACCCACGGATCCGACCACCTACATAACCTAAACCTCCTACAATGC---------GTGGAAGCAACCCTACTCCTTAACACCTTCATACTCCTCACACTACTAATTATCCTCACAC : 9091
Falco_peregrinus           : CTAGTAGCCTCAACCCGCACCCACGGTTCTGACCACCTACAAAACTTAAACCTCCTCCAATGC---------ATGGGAACCACAATACTCTTAAGCACCTCCATAATCCTCACGCTCACATTAATTTTAACCC : 9088
Dromaius_novaehollandiae   : CTAGTATCATCAACCCGAACACATGGTTCTGACCACCTACATAACCTAAACCTCCTACAATGC---------ATGGAGACTACACTACTCCTCAATACCTCCATACTCCTTACACTAATCATCTTACTCACAC : 9082
Struthio_camelus           : CTAGTCGCATCAACACGAACACACGGCTCAGACCACCTCCACAACCTAAACCTATTACAATGC---------GTGGAAACCACACTACTTTTCAACACCTCCCTTTTACTCACACTAATAATTCTTCTTACAC : 9088
Apteryx_haastii            : TTAGTAGCCTCTACTCGCACGCATGGCTCCGACCACCTACATAACCTAAACCTCCTACAATGC---------ATGGAGACCACACTACTCCTTAACACCTCTATCCTTCTCACACTGACAATTTTATTCACAC : 9076
Rhea_americana             : CTAGTGGCATCTACTCGAACCCACGGCTCCGACCACCTCCACAACCTCAACCTCCTACAATGC---------ATGGAGACTACCCTTCTCCTCAACACCTCCATACTTCTTCTACTAACAACCCTCCTCACAC : 9088
Gallus_gallus              : CTAGTAGCTTCTGCACGAACACATGGCTCTGACCACCTACACAACCTAAACCTTCTACAATGC---------ATGGAGACTCCACTGCTCCTTAACACCCTCACAACATTAACACTACTCACCCTCCTTACCC : 9088
Ciconia_ciconia            : CTAGTAGCCTCTACCCGAACCCATGGCTCTGACCACCTACACAACCTAAATCTCCTACAATGC---------ATGGAAACCGCATTACTCCTCAACACCTCTATGCTCCTCACGCTAGCAACCATCCTTACCC : 9094
Ciconia_boyciana           : CTAGTAGCCTCTACCCGAACCCATGGCTCTGACCACCTACACAACCTAAACCTTCTACAATGC---------ATGGAAACCGCATTACTCCTCAACACCTCTATACTCCTCACGCTAGCAGCCATCCTTACCC : 9091
Iguana_iguana              : CTAGTAGCAACATCACGCACACACGGAAACGACCACCTAAAAAACCTAAACCTCCTACAATGC---------------ATGCACACACTCCCTACCACAACAACACTCACCACACTACTAACCCTCACACTTC : 9070
Eumeces_egregius           : CTAGTCGCAACAGCCCGAACCCACGGGGCAGACCACCTGCAAAACCTGAACCTACTACAATGC---------------ATGCACAGCCTCTCCCAAAGCCTAATCACCGCCACCTTTATTCTTCTACTTGTCC : 9070
Sphenodon_punctatus        : CTTGTAACTACAACTCGAACACACGGATCAAGCAATATAAACAACCTAAATCTCCTTCAATGC---------------------------------------------------------------------- : 9024
Sceloporus_occidentalis    : CTAGTAGCAACCTCTCGTACACACGGCAACGACCACCTTAAAAACCTAAGCCTATTACAATGC------------ATGCACACCACCCTATCCACAACCACCATATTAACTGCTATAGTCATCCTAGCAGCCC : 9073
Cordylus_warreni           : CTAGTTGCAACATCTCGCACACACGCAACAGATCACCTCAAAAACCTCAGCCTCCTACAATGC------------ATTACCAATTTCTTAGCACACACCTCTATTATAACCACCCTTCTAGTCCTCCTCATCC : 9067
Abronia_graminea           : CTAGTATCAACATCACGAACACACGGAAACGATCATCTAAGCAACCTCAACATTCTACAATGC------------ATGTACCCAATATTGACACAAACACTCACCCTAACAATATTAATTATCCTTATATACC : 9076
Shinisaurus_crocodilurus   : CTAGTCGCTACCTCTCGCACTCACGGAAATGACCACCTCCATAATCTAAGTATCCTACAATGC------------ATGCACTCCCTACTCACCCAAACCTCCATAATACTTATACTTTTTTTACTACTTTTTC : 9052
Varanus_komodoensis        : CTAGTAGCGACAGCCCGAACCCATTCCAGCGACCACCTCAACACTATAAACATCCTAAAATGC------------ATATACCACCCCCTCATCCTGACCTCCATAATCCTCACCCTAATCCTCCTGACCCTCC : 9109
Rhineura_floridana         : CTAGTGACCACATCACACACACACGGCTCAGACAACCTTAAAACCATAAGTCTACTAAAATGT---------------ATGGCCACACTCATAACATCCTCTTCACTAACAATACTATTAGTATTAATTACAC : 9052
Geocalamus_acutus          : CTTGTCGCAACAATTCGAACTCACAGCTCAGACCACATAGCTGCTATAAATATATTACAATGT------------ATGACTATAACCTACACCAGTGTGCTGGTCACTTTAATATTACTTTTATCCCCAATTA : 9034
Diplometopon_zarudnyi      : CTAGTCGCCACCACCCGAACACACAGCTCAGATCATCTAAACACAATAAACCTCCTACAATGC------------ATGAATCTAATCTTATATTCAACCAACCTACTAACCGCACTACTACTAACTCTACCCT : 9049
Amphisbaena_schmidti       : CTTGTTGCCACTGTCCGCACCCACAGCTCAGATCACATAAACAACATCAACATATTACAATGC------------ATGAACGCATTATTTCTCACTAGCAGCTCCATCATCCTCCCACTTCTCTCGCTACCAA : 9034
Bipes_tridactylus          : CTGGTGACAACGATTCGAACCCACACCACCGATCATATTAACAGCCTTAACGTCCTACAATGT------------ATGCTTGATCTTTTCATCCTCTTCGCCGCTGTCCCACTAATTCTATTCCTCCCACTAG : 9040
Bipes_canaliculatus        : TTAGTGGCAACAACCCGCACGCACAGCACAGACCATGTAAACAGCATAAACATCCTGCAATGC------------ATGCCAAGCCTATTTCTACTATTTATCACCATCCCACTAATCTTACTCACCCCCCTAC : 9052
Bipes_biporus              : CTAGTAGCAACAACCCGCACACACAGCACAGACCACGTAAGCAACTTGAACATCCTACAATGC------------ATGTTTGACCCGCTAATACTATTCGCAACCATCCCCCTAATCCTAGCCACTCCACTAC : 9094
Anolis_carolinensis        : CTTGTAGCAACTGCCCGAACACACGGAAACGACCACCTACAAAATCTAAACCTACTACAATGT------------ATGCACAACACTATTTTCTTCTCTTCAATATTATCCACACTTTTTATTATTATTTTAC : 8715
Ophisaurus_attnuatus       : CTAGTTGCAACTTCCCGCACCCATGGAAACGACCACTTAAACAACCTAAACATCCTACAATGC------------ATGCACTCTACTCTCACCCAAACCTCAATCCTAGCCACATTATTGATCCTCATCTTTC : 8697
Varanus_salvator           : CTAGTAGCAACAGCCCGCACCCACTCCAACGACCATCTTAACACAATAAACATCCTAAAATGC------------ATACACCACCCTCTCACCCTAACCTCCATAATTTTATCCATGACCCTCCTAATCCTTC : 9100
Mertensiella_luschani      : ATAGTAGCCACCACACGAACACACGGAACAGATCATTTAAAAAACTTAAACCTCCTACAATGC---------ATGAATCTCGTATTAACGCTAAACTCTTCATTTATACTCTCATTAGTTATCTTAGCATTAC : 9094
Xenopus_laevis             : AATAGTGACCACTACACGACTCATGGAACAGATAAATTATTTAGCCTAAACCTCCTAGAATGT---------ATGAATTTTCCACTAATCTTCAACTCCTCCATATTAATTACAATCTCAATTCTAATTTTAC : 9085
```

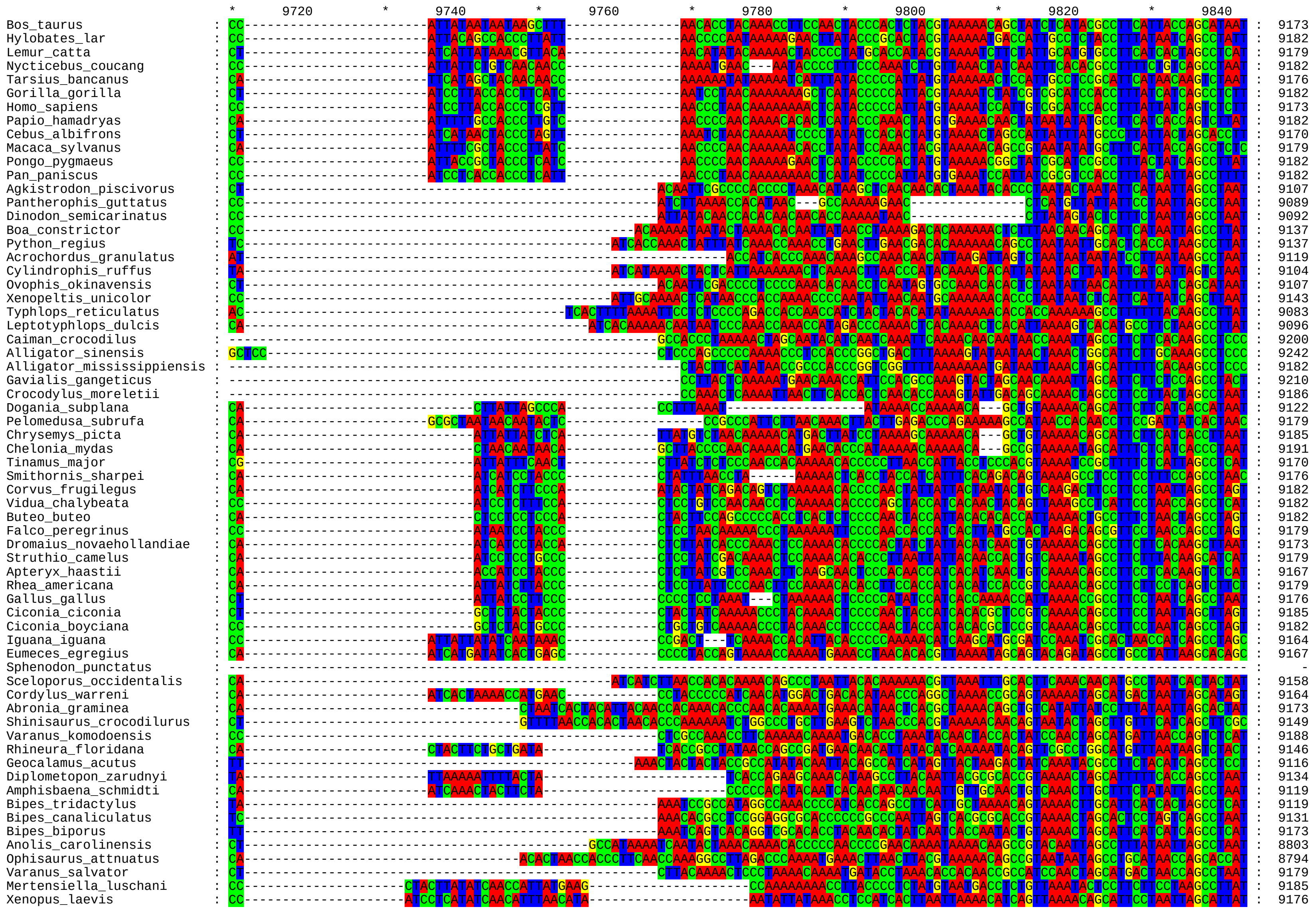
9720 9740 9760 9780 9800 9820 9840
Bos_taurus 9173
Hylobates_lar 9182
Lemur_catta 9179
Nycticebus_coucang 9182
Tarsius_bancanus 9176
Gorilla_gorilla 9182
Homo_sapiens 9173
Papio_hamadryas 9182
Cebus_albifrons 9170
Macaca_sylvanus 9179
Pongo_pygmaeus 9182
Pan_paniscus 9182
Agkistrodon_piscivorus 9107
Pantherophis_guttatus 9089
Dinodon_semicarinatus 9092
Boa_constrictor 9137
Python_regius 9137
Acrochordus_granulatus 9119
Cylindrophis_ruffus 9104
Ovophis_okinavensis 9107
Xenopeltis_unicolor 9143
Typhlops_reticulatus 9083
Leptotyphlops_dulcis 9096
Caiman_crocodilus 9200
Alligator_sinensis 9242
Alligator_mississippiensis 9182
Gavialis_gangeticus 9210
Crocodylus_moreletii 9186
Dogania_subplana 9122
Pelomedusa_subrufa 9179
Chrysemys_picta 9185
Chelonia_mydas 9191
Tinamus_major 9170
Smithornis_sharpei 9176
Corvus_frugilegus 9182
Vidua_chalybeata 9182
Buteo_buteo 9182
Falco_peregrinus 9179
Dromaius_novaehollandiae 9173
Struthio_camelus 9179
Apteryx_haastii 9167
Rhea_americana 9179
Gallus_gallus 9176
Ciconia_ciconia 9185
Ciconia_boyciana 9182
Iguana_iguana 9164
Eumeces_egregius 9167
Sphenodon_punctatus -
Sceloporus_occidentalis 9158
Cordylus_warreni 9164
Abronia_graminea 9173
Shinisaurus_crocodilurus 9149
Varanus_komodoensis 9188
Rhineura_floridana 9146
Geocalamus_acutus 9116
Diplometopon_zarudnyi 9134
Amphisbaena_schmidti 9119
Bipes_tridactylus 9119
Bipes_canaliculatus 9131
Bipes_biporus 9173
Anolis_carolinensis 8803
Ophisaurus_attnuatus 8794
Varanus_salvator 9179
Mertensiella_luschani 9185
Xenopus_laevis 9176

```
                                     *     9860        *     9880        *     9900        *     9920        *     9940        *     9960        *
Bos_taurus                      : TCCCACAATAATATTTATCCACTCAGGCCAAGAACTAATTATTTCAAACTGACACTGACTAACCATCCAAACTCTTAAATTATCCCTCAGCTTTAAAATAGACTATTTCTCAATAATATTTATCCCAGTAGCA :  9306
Hylobates_lar                   : TCCCACAATAATATTCATGTGCACAGACCAAGAAACCATTATTTCAAACTGACACTGAACTGCAACCCAAACGCTAGAACTCTCCCTAAGCTTCAAACTAGACTACTTCTCCATAATATTCATCCCCATCGCA :  9315
Lemur_catta                     : CCCAACTATATTATTTATCTCCTCAGGACAAGAAACAATCATTTCCAACTGACATTGAATAACAATCCAAACCCTAAAACTATCTATTAGCTTTAAATTAGACTACTTCTCTATACTATTTATACCAGTAGCA :  9312
Nycticebus_coucang              : TCCAGCTCTTCTCTTTGTTTTCTCAGGCCAAGACTCAGTCATTACAAATTGACACTGAATAACAATCCAAACCCTAAAACTATCCACAAGCCTAAAATTAGACTATTTCTCCATCTTATTTATACCTGTAGCA :  9315
Tarsius_bancanus                : TCCAATAACTATATTCCTACATACAAATCAAGAAGCAATTGTTTCTAACTGACACTGAATGACCATCCAAACCATAAAACTCTCCTTAAGCTTCAAAATAGACTATTTCTCCATAATTTTTATACCCGTAGCC :  9309
Gorilla_gorilla                 : CCCCACAACAATATTTCTATGCCTAGACCAAGAAGCTATTATCTCAAGCTGACACTGAGCAACAACCCAAACAATTCAACTCTCCCTAAGCTTTAAACTAGATTATTTCTCCATAATATTTATCCCTGTAGCA :  9315
Homo_sapiens                    : CCCCACAACAATATTCATGTGCCTAGACCAAGAAGTTATTATCTCGAACTGACACTGAGCCACAACCCAAACAACCCAGCTCTCCCTAAGCTTCAAACTAGACTACTTCTCCATAATATTCATCCCTGTAGCA :  9306
Papio_hamadryas                 : TCCAACAACCTTATACATCTTCTCAAACCAAGAAACAACCATCTGGAGCTGACACTGAATAATAACCCAAACATTAGACCTAACACTAAGCTTTAAACTAGACTACTTTTCCATAATGTTTATCCCAATCGCA :  9315
Cebus_albifrons                 : GTCTATAACAATATTTATTCTCACAGGCCAAGAATCAATAATTTCAAACTGACATTGAGCGACAATCCAAACTATCAAACTATCACTAAACTTTAAACTAGACTTCTTCTCCATAATATTCACGCCTGTAGCA :  9303
Macaca_sylvanus                 : TTCAACAACTTTATATATATTCTTAAACCAAGAAACAATCATCTGAAGCTGGCACTGAATAATAACCCAAACACTAAGCCTAACATTAAGCTTTAAACTAGACTACTTCTCCATAATATTCACCCCAATCGCA :  9312
Pongo_pygmaeus                  : CCCAACAACAATATTTATCTGCCTAGGGCAAGAAACCATCATCACAAACTGATGTTGAACAACCACCCAAACACTGCAACTCTCACTAAGCTTTAAACTTGACTACTTCTCCATAACATTCCTCCCCGTAGCA :  9315
Pan_paniscus                    : CCCCACAACAATATTCATATGCCTAGACCAAGAAGCTATTATCTCAAACTGGCACTGAGCAACAACCCAAACAACCCAGCTCTCCCTAAGCTTTAAACTAGACTACTTTTCCATAACATTTATCCCCGTAGCA :  9315
Agkistrodon_piscivorus          : CCCCCTTAACACACTATTAAACAATAACAACGAACTAACAATAACGCTATTCCCCCTAATTACAACTCCAACAGAAAACATTAATATCTCCCTCACACTCGATACACTATCCTTAACCTTTATCCCAGTAGCG :  9240
Pantherophis_guttatus           : CCCAATTAACCAGCTATCAAACAACAGT---GAACTTACACTATCGCTAACACCATTAATCATTACACCAACAGAAAACATTAATATTTCCATTAACATAGACACCCTATCACTAACATTTATCCCAATTACC :  9219
Dinodon_semicarinatus           : TCCAATCAACCCGCTATTAAATAACAAC---GAACTAACACTAACACTCACACCACTAATTATCTCACCAACAGAAAATATCAATATCTCTATTACCCTGGACACAGCATCACTACTATTCACCCCAATCGCC :  9222
Boa_constrictor                 : CCCCCTATCTACACTACTTAACAACGAAAACGAAATCATAATATCTTCTCCAACCATTATCAACACAACAACCATAAACATCAACATAACCATTATACTAGACTACCCATCACTCACATTCATCCCAATCGCC :  9270
Python_regius                   : TCCCCTGACCCTCCTCCTAAATAATGAAAACGAAATAACACTATCTACACCTCCAATTATTAACACAGCCACAACAAATATTAACATCAGCTTCATCCTTGACACACCCTCACTAACCTTCATCCCTATTGCT :  9270
Acrochordus_granulatus          : CCCTTTAAACTTCATTACCAATAAAGAACAAGAAACAACAATCTCTTCTTTACCAATAATTTCAACACCAACAGAAAACATCAACATCTCAATCCTATTAGATTTACCATCACTTACATTCATGCCAGTGGCC :  9252
Cylindrophis_ruffus             : TCCACTTAGCCTCCTACTGAATAACGAAAACGAAACCATACTATCATCCCCCCCAATTATTTATATAACAACAACAAACATCAGCATCAGCCTCATCTTAGATACTTTCTCATTAACCTTTATCCCAGTCTCT :  9237
Ovophis_okinavensis             : CCCCCTTAACATACTACTAAACAACAATAACGAACTAACAATCTCACTGCCCCCCCTAATTATAACTCCAACAGAAAATATCAACATCACCATCACCCTCGACACACTCTCCTTAACCTTTATCCCAGTAGCA :  9240
Xenopeltis_unicolor             : CCCCCTAAGCCTAATACTAAACTATAACATAGAAATTACAATATCTTCATCCCCAATTATCCTCATAGCAACAACAAACATCAATATCAGCCTCATTCTAGACACACTCTCATTAACTTTGTTCCAATCGCC :  9276
Typhlops_reticulatus            : CCCGACTACCCTCCTACTAAAAAATGAAATTAACTCCTCACTTAAATTTCCATCCCACATCAGTTTAGAAACTTTTAATATCTACGTTAACTTTTCACTTGACACATATTCAATCATATTCTCCTCTATTGCT :  9216
Leptotyphlops_dulcis            : CCCACTAACATCCTCC---CTCCAAAAAACAGAAACCCACGTAACCATCATAACATACATACAAATAAACACATTAAACCTAAACCTCAACACAACCTTAGACCAACAAACAACAGTATTACAACAACAGCA :  9226
Caiman_crocodilus               : CCCTCTTCTCTTAACCTTACCAAACAACAACACAACCTTGTCAAACCACTTTTACTTACTACACTTAGAATCTTTCAACCTACCCATCGCCCTAAAAATTGACACATACTCAGCCTTCTTCATTCCAACAGCA :  9333
Alligator_sinensis              : TCCCCTCCTCTTATTAGTCTATAACAACACAACCGCTCTCTCATTCCAATGACACTGACTCAACGTAGGAACTTGCTCTGTTCACCTGGGCCTTAAAGTTGACACCTTCTCAGTCTTCTTTATCCCAACAGCC :  9375
Alligator_mississippiensis      : TCCCCTTATTTTATTAACTTACAACAACATAGTTCCCCTCTCATTCACTTGACATTGACTCAGCATAGGAACCTGCCCCATCTACTTAAGCCTTAAACTCGACACCTTCTCAATCTTCTTCGTCCCCACAGCA :  9315
Gavialis_gangeticus             : TCCATTAGCCTTTTTCTCTACGACGGATTAGTTGTTACCTCACACGAAGTTTATTGACTAGCCTTCAACGTATGCCCCATTCACATTAGCTTCACACTAGACAAATATTCAGTCCTCTTCCTACCAATCTCA :  9343
Crocodylus_moreletii            : CCCACTGACCTATCTCATCTATAATGACTTAACCATTACTACATACGAAACCCAATGATCAACAATCAGCACAACCACAATTCGCATTAGCTTCACGCTAGACATTTACTCCACCTTCTTCTTACCCATCCTT :  9319
Dogania_subplana                : TCCATCAACCACGTTCATCTTCTCCAATACTGAACACATTATCACTAATTGGTCCTGAACAATAACCTCCACATTCACAGTATCAATGAGCTTCAAATTCGACCAATACTCAATCATATTTATCCCAGTAGCC :  9255
Pelomedusa_subrufa              : TTCAATTCTCATCCATATTTTAATCGAAGAGCCTTCATCAATTTCA------TCCCTTTATTCACCCACCATACTTAACCTAGCCATAAGCATTAAAATTGACTACTACTCCCTAATTTTTATTTCAATTGCC :  9306
Chrysemys_picta                 : TCCATTAGGCATATTAGTAATAATACCC---GAAAATATCTACACTAACATTACTCTGGTAAGCATATCTTCATTCTTCATCGAACTAAACTTAATTCATGACATATATGCCCTCATTTTTATACCAGTCGCC :  9315
Chelonia_mydas                  : TCCACTATCTATTTTCACTTACACAGATATTGAATCTATCATCACCAACTTACACTGATCAACTACATCCACATTCACCATAAATATAAGCTTTAAACTTGACCAATACTCCATCATATTTGTCCCAATTGCC :  9324
Tinamus_major                   : TCCCATACTCACCTTCATCCTCTCCGGAGCTAACTCATTTACCTCCTACTGACACTGAAACTTCATCACTAATTTCAAAATCCCCATAACCTTCAAACTTGACCAGTACTCCATAATATTCTTCCCAATCGCC :  9303
Smithornis_sharpei              : CACAACAATCATCTTCCTCCACTCAAACCTAGAAAGCATTACTTCACACTGAGAATGAAAATTTACCATAAACTTTAAAATCCCAATTGACCTCAAAATAGACTACTACTCCATGACATTCTTCCCCATTGCA :  9309
Corvus_frugilegus               : CCCCATAACCATTTACATTCACTCAGGAATAGACAGCCTAACTTCTTTCTGAGAATGAAAATTCATTATAAACTTCAAAATCCCTGTCAGCCTAACAATAGACTTCTACTCACTAACTTTCTTCCCAATCGCC :  9315
Vidua_chalybeata                : CCCAATAACAATCTACATCCATTCAGGGACAGAAAGCCTAACCACCCTTTGAGAATGAAAATTCATCATAGGATTTAAAATCCCAGTCAGCCTAAAAATAGACTTCTACTCCCTCACTTTCTTCCCAATCGCC :  9315
Buteo_buteo                     : ACCAATATTCCTCTTCATATGCTCAGGATCAGAAAGCATCACCTCCCACTGAGAATGGAAATTCATCACAAACTTTAAAATCCCCCTCAGCTTCAAAATCGATCAGTACTCCATAATATTCTTTCCAATCGCC :  9315
Falco_peregrinus                : ACCCATAGTAATCTTCATATACTCAAACACAGAAAGCATTACCACCTGCTGAGAATGAAAATTCACCGCAAACTTCAAAATCCCAATTAGTCTAAAGATAGATCAGTACTCAATCATATTCCTCCCTATTGCA :  9312
Dromaius_novaehollandiae        : CCCTATATCAATCTTCATCTACTCTGGGACAGAGACCATCATCTCACAGTGATATTGAAACTTCACCCCTAACTTCAAAATCCCCCTCAGCTTTAAAATAGACCAATACTCAATACTATTCTTCCCTATTGCC :  9306
Struthio_camelus                : CCCAACAACCATCTTCATCTACTCAGGAACAGAAGCAATTATTTCCCACTGGTACTGAAGTTTCACCCCCAATCTCAAAATCCCACTCAGCTTCAAAATAGACCAATATTCAATACTATTCCTCCCTGTAGCC :  9312
Apteryx_haastii                 : TCCTACAACTATCTTCATTTACTCCGGGGCAGAAACCATCATCTCTCATTGGTACTGAAACTTCACCCCCAACCTCAAAGTCCCACTCAGCTTCAAAATAGATCAGTATTCAATATTATTTCTCCCCATCGCC :  9300
Rhea_americana                  : CCCCATATTCATCTTCATCTACTCTGGCACCGAAACTATCGTCTCCCACTGACACTGAAACTTTACCCCTAACTTCAAAATTCCCCTAAGCTTCAAAATAGACCAATACTCCATACTATTTTTCCCTATCGCC :  9312
Gallus_gallus                   : CCCAACAACAATCTTCATCCACTCAGGGGCAGAAAGCATTGCCACCCACTGAGAATGACAATTTATCCCCAACTTCAAGATCCCCATCTCCCTAAAAATAGACATATACTCCATAATATTCTTCCCCATTGCA :  9309
Ciconia_ciconia                 : ACCAGCAACCCTCTTCACGTACTCAGGCACAGAAAGTATCGTCTCCTGCTGAGAATGAAAATTCATCATAAACTTCAAAATCCCCCTTAGCCTCAAAATAGACCAATACTCCACACTATTCTTCCCCATCGCA :  9318
Ciconia_boyciana                : CCCAGCAACCCTCTTCACGTACTCAGGCACAGAAAGTATCGTCTCCTGCTGAGAATGAAAATTCATCATAAACTTCAAAATCCCCCTTAGCCTCAAAATAGACCAATACTCAATACTATTCTTCCCCATCGCA :  9315
Iguana_iguana                   : CCCAATACTCAACTTCATCAACTACGGCACAGAATCCACAATCACCAACATCTCCCTCACCACTGTCAACAACTTCAACATTAAAATCAGCTTCATCCTAGACAGCTACTCCCTAATCTTCGTACCTGTCGCC :  9297
Eumeces_egregius                : GCCCCTTCTCATGTTCATCAACTATGGCATAGAGTCAACAATCACAAACCTGCACTGAGTACATATCACAAACTTTAACATCAACATTAGCTTCACACTGGACACTTACTCAATCATATTTACACCTATTGCC :  9300
Sphenodon_punctatus             : ------------------------------------------------------------------------------------------------------------------------------------ :     -
Sceloporus_occidentalis         : CCCAGCAATACTATTCATCAACTATGGGGAAGAGTTGGCACTTACTAACTTCTCCCTACTACTAATTAGTAATTTTAACATCAAAATCAGCTTTATTATAGACATATACTCACTATCATTTACCCCAATTGCA :  9291
Cordylus_warreni                : TCCCCTACTAACCTTTTATACTCACGGCGTAGAAACCTCTATAACAAGCCTAAAATGAACAACCATTGAAAACTTCGACATTAATCTCAGCATCATACTTGACCAGTACTCCACTATATTTCTCCCAGCTGCC :  9297
Abronia_graminea                : TCCCCTACTATCCTACATCAATAATGGAGTCGAAACTACTATTTTACACATTCACCTAATTAATATCACCAATTTCAGCATTAACTTAAGCTTTATTTTTGACCAATACTCACTTACATTCACCCCAATTGCC :  9306
Shinisaurus_crocodilurus        : TCCCCTCTCCTCTTTTATTAATAACGGTGTAGAAACAACAATCATTAACCTCCATCTAACTAACATTACCACGTTTAATGTCAACATTAGCTTCATCTTCGACCTTTACTCACTCGTGTTCTCACCAATCGCG :  9282
Varanus_komodoensis             : TCCTCTCCTCTCTCACATTAACAAAGGAGCAGAAACCACTATCACCAATATTCAATGAATTTTAACCAATAACTTCAATATCAATATTAGCTTCATTTTCGACACCTATGCACTCACCTTTCTCCCCATTGCC :  9321
Rhineura_floridana              : CCCCCTTGCAATCCACCTAAACACAGGGCTAGAAGCACAAACACTTAACTACCAACTACTTCTGGCCCAAAACCTGACTATTAATTTCAACTTCATCATAGACACATACTCTCTAATCTTTATCCCAATTGCT :  9279
Geocalamus_acutus               : GCCACTCTTAACTATACAGAACACAGGATTAATGCACATCACACTATCACTAACCTGATTACAACTAAATAATGTACAAGTACAATTAAACTTTATGTACGATATTTACAGCCTTCTATTTACGCCCGTCGCA :  9249
Diplometopon_zarudnyi           : CCCACTGAGCATTTTATTAAACACCGGAATAGACCACTCAACTATTTCTATAACCTGATTCTCAATAATTAACCTCCAACTAAAACTAAATATACTATACGATATATATTCATTAATATTTATCCCAGTGGCC :  9267
Amphisbaena_schmidti            : CCCACTACTGATCGCCTACAACACAGGACTAGAACATATAACCATCTCTATAACATGGTACAGCTTCACCAACTTTCAACTAAGCTTAGACCTAATACAAGATACCTACACCCTCACCTTCACCCCCGTAGCA :  9252
Bipes_tridactylus               : GCCACCAGTACACATACTAAACCTTGGAGTCAACCAAATAACCTGATCATTTACATGGTTTACCACCAGCAACTTGCCCCTTCACATCAACCTACTCATGGATCCATATACAATGATATTTACACCAACAGCT :  9252
Bipes_canaliculatus             : CCCACCAATCCACATGTTAAACACAGGCATCAGCCAAATAACATGATCCATTACGTGATTTGACACCAACAACCTACCATTGCAAATAGGCCTCCTCGTAGACACATACACAATAATATTCACCCCAACCGCC :  9264
Bipes_biporus                   : CCCACCAACGTACATGCTAAACACTGGCGGCCAACAAATGAATTGCACCATTACATGATTTGCTACCAACACCTTACCACTACAACTAGACCTAATCGTAGACCCCTACACAATAGCATTCACCCCAACCGCC :  9306
Anolis_carolinensis             : TCCCCTTCTTCTTTTT?TTAACAATGGCATAGAAGC?ACCCTAACAAACTTTTCACTATTGTCAATTAATAACTTCAATATTAAACTTAGCTTTATACTAGACAGCTACTCACTAATATTCATACCCATTGCC :  8934
Ophisaurus_attnuatus            : CCCCCTTATATCACTAATCAACAAAGGAGTAGAAATAACGATTATACACATCCACCTACTCAGCATCTCCAACTTC?ACGTTAATTTAAGCCTCATCTTTGACCAGTACTCACTCACCTTCACCCCCATTGCT :  8926
Varanus_salvator                : CCCCCTTCTTTTCTTTATTAACAACGGGGCAGAAACCACCATCACTAACATTCAATGGATACTAACCAGCAACCTAAACATCAACATTAGTTTCATTTTTGATATCTACTCCATCACCTTCCTCCCAATCGCC :  9312
Mertensiella_luschani           : GCCCTTACTTATCTTTATCAACCACGGCCTAGAATCTACTACAATTAACTTCTCATGAATAACCATCCATAACTTCAATATCTCATCCAGCATTAAGCTAGATCAGTATTCAATCTTATTCACACCAATTGCC :  9318
Xenopus_laevis                  : TCCACTTATCATTTTTCTAGACCAAGGTCTTGAATCAATTACCACTAACTTTCACTGAATAAACATTAATACATTTGACATTAATATGAGCTTTAAATTTGATATTTATTCTTCTATTTTTATCCCTATCGCT :  9309
```

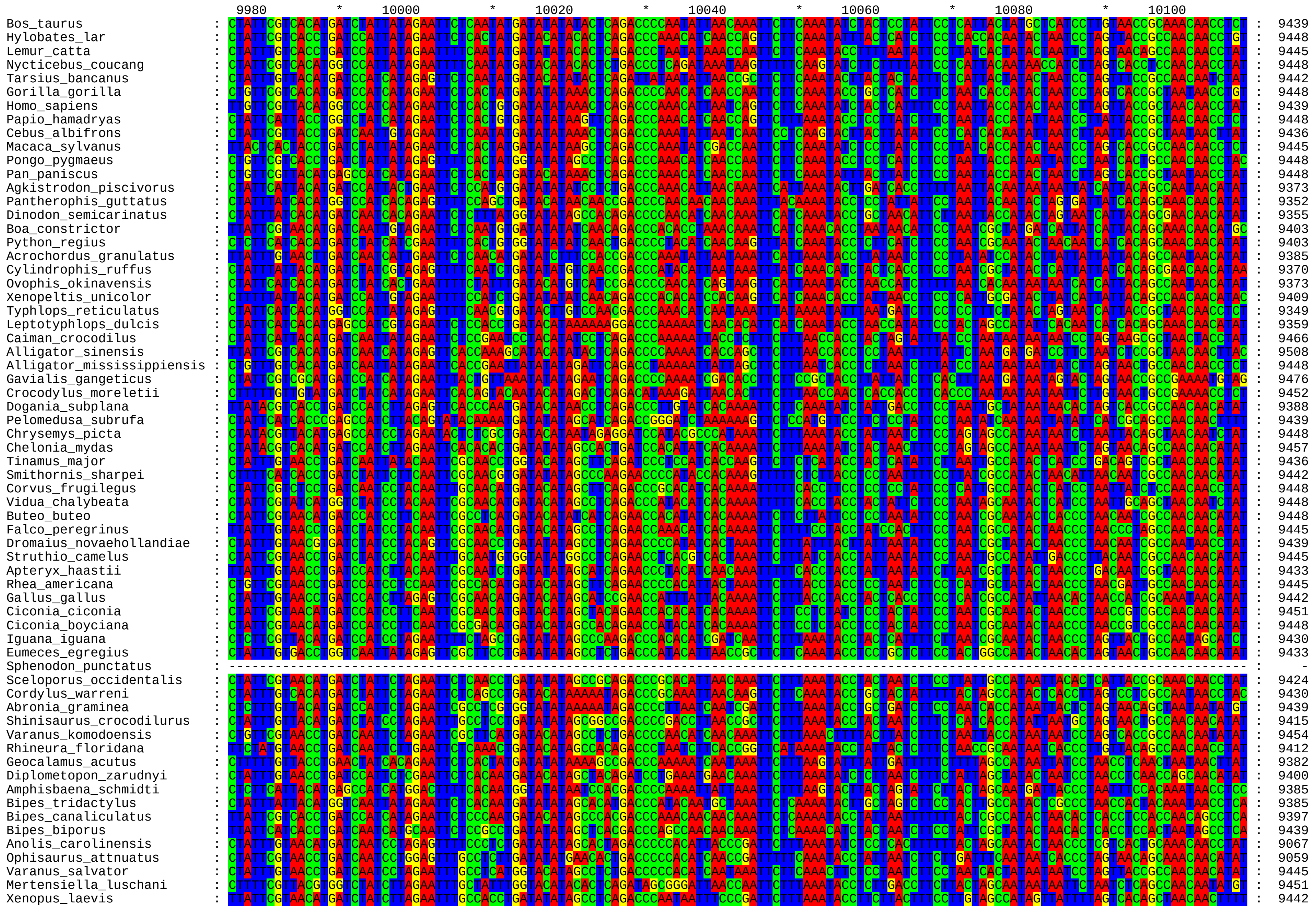
9980 * 10000 * 10020 * 10040 * 10060 * 10080 * 10100
Bos_taurus : 9439
Hylobates_lar : 9448
Lemur_catta : 9445
Nycticebus_coucang : 9448
Tarsius_bancanus : 9442
Gorilla_gorilla : 9448
Homo_sapiens : 9439
Papio_hamadryas : 9448
Cebus_albifrons : 9436
Macaca_sylvanus : 9445
Pongo_pygmaeus : 9448
Pan_paniscus : 9448
Agkistrodon_piscivorus : 9373
Pantherophis_guttatus : 9352
Dinodon_semicarinatus : 9355
Boa_constrictor : 9403
Python_regius : 9403
Acrochordus_granulatus : 9385
Cylindrophis_ruffus : 9370
Ovophis_okinavensis : 9373
Xenopeltis_unicolor : 9409
Typhlops_reticulatus : 9349
Leptotyphlops_dulcis : 9359
Caiman_crocodilus : 9466
Alligator_sinensis : 9508
Alligator_mississippiensis : 9448
Gavialis_gangeticus : 9476
Crocodylus_moreletii : 9452
Dogania_subplana : 9388
Pelomedusa_subrufa : 9439
Chrysemys_picta : 9448
Chelonia_mydas : 9457
Tinamus_major : 9436
Smithornis_sharpei : 9442
Corvus_frugilegus : 9448
Vidua_chalybeata : 9448
Buteo_buteo : 9448
Falco_peregrinus : 9445
Dromaius_novaehollandiae : 9439
Struthio_camelus : 9445
Apteryx_haastii : 9433
Rhea_americana : 9445
Gallus_gallus : 9442
Ciconia_ciconia : 9451
Ciconia_boyciana : 9448
Iguana_iguana : 9430
Eumeces_egregius : 9433
Sphenodon_punctatus : -
Sceloporus_occidentalis : 9424
Cordylus_warreni : 9430
Abronia_graminea : 9439
Shinisaurus_crocodilurus : 9415
Varanus_komodoensis : 9454
Rhineura_floridana : 9412
Geocalamus_acutus : 9382
Diplometopon_zarudnyi : 9400
Amphisbaena_schmidti : 9385
Bipes_tridactylus : 9385
Bipes_canaliculatus : 9397
Bipes_biporus : 9439
Anolis_carolinensis : 9067
Ophisaurus_attnuatus : 9059
Varanus_salvator : 9445
Mertensiella_luschani : 9451
Xenopus_laevis : 9442

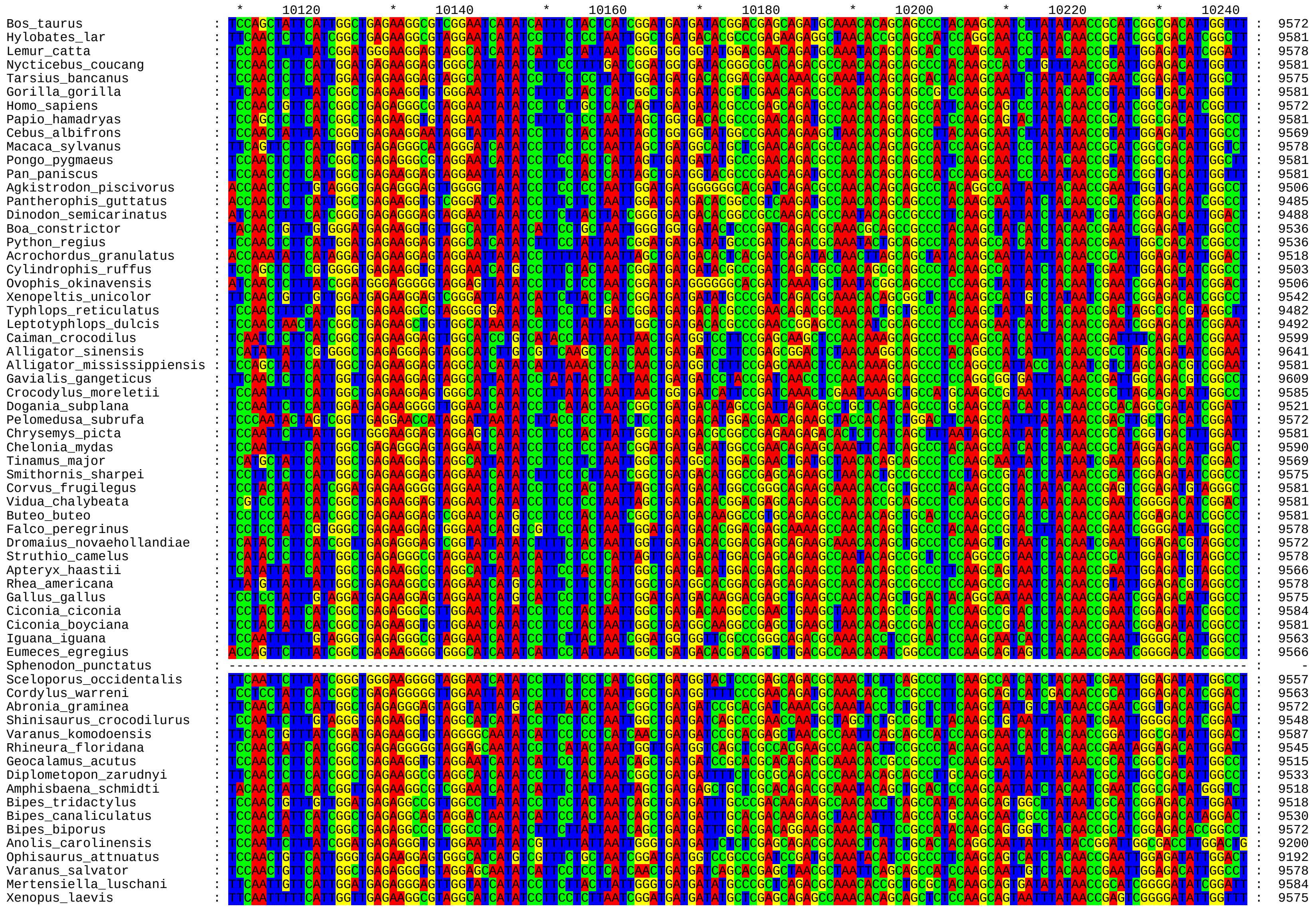

10120 10140 10160 10180 10200 10220 10240
Bos_taurus : 9572
Hylobates_lar : 9581
Lemur_catta : 9578
Nycticebus_coucang : 9581
Tarsius_bancanus : 9575
Gorilla_gorilla : 9581
Homo_sapiens : 9572
Papio_hamadryas : 9581
Cebus_albifrons : 9569
Macaca_sylvanus : 9578
Pongo_pygmaeus : 9581
Pan_paniscus : 9581
Agkistrodon_piscivorus : 9506
Pantherophis_guttatus : 9485
Dinodon_semicarinatus : 9488
Boa_constrictor : 9536
Python_regius : 9536
Acrochordus_granulatus : 9518
Cylindrophis_ruffus : 9503
Ovophis_okinavensis : 9506
Xenopeltis_unicolor : 9542
Typhlops_reticulatus : 9482
Leptotyphlops_dulcis : 9492
Caiman_crocodilus : 9599
Alligator_sinensis : 9641
Alligator_mississippiensis : 9581
Gavialis_gangeticus : 9609
Crocodylus_moreletii : 9585
Dogania_subplana : 9521
Pelomedusa_subrufa : 9572
Chrysemys_picta : 9581
Chelonia_mydas : 9590
Tinamus_major : 9569
Smithornis_sharpei : 9575
Corvus_frugilegus : 9581
Vidua_chalybeata : 9581
Buteo_buteo : 9581
Falco_peregrinus : 9578
Dromaius_novaehollandiae : 9572
Struthio_camelus : 9578
Apteryx_haastii : 9566
Rhea_americana : 9578
Gallus_gallus : 9575
Ciconia_ciconia : 9584
Ciconia_boyciana : 9581
Iguana_iguana : 9563
Eumeces_egregius : 9566
Sphenodon_punctatus : -
Sceloporus_occidentalis : 9557
Cordylus_warreni : 9563
Abronia_graminea : 9572
Shinisaurus_crocodilurus : 9548
Varanus_komodoensis : 9587
Rhineura_floridana : 9545
Geocalamus_acutus : 9515
Diplometopon_zarudnyi : 9533
Amphisbaena_schmidti : 9518
Bipes_tridactylus : 9518
Bipes_canaliculatus : 9530
Bipes_biporus : 9572
Anolis_carolinensis : 9200
Ophisaurus_attnuatus : 9192
Varanus_salvator : 9578
Mertensiella_luschani : 9584
Xenopus_laevis : 9575

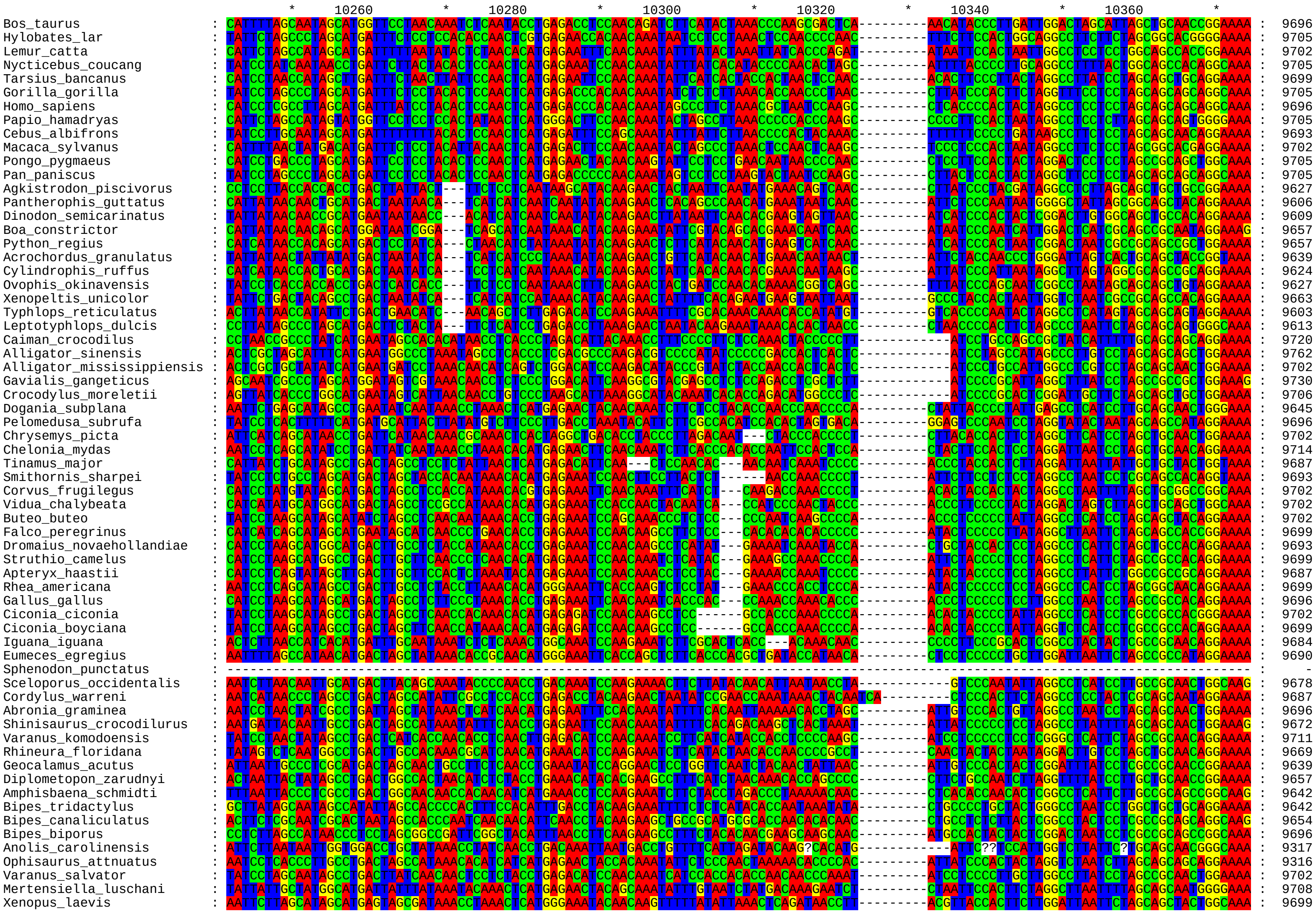
10260
10280
10300
10320
10340
10360
Bos_taurus 9696
Hylobates_lar 9705
Lemur_catta 9702
Nycticebus_coucang 9705
Tarsius_bancanus 9699
Gorilla_gorilla 9705
Homo_sapiens 9696
Papio_hamadryas 9705
Cebus_albifrons 9693
Macaca_sylvanus 9702
Pongo_pygmaeus 9705
Pan_paniscus 9705
Agkistrodon_piscivorus 9627
Pantherophis_guttatus 9606
Dinodon_semicarinatus 9609
Boa_constrictor 9657
Python_regius 9657
Acrochordus_granulatus 9639
Cylindrophis_ruffus 9624
Ovophis_okinavensis 9627
Xenopeltis_unicolor 9663
Typhlops_reticulatus 9603
Leptotyphlops_dulcis 9613
Caiman_crocodilus 9720
Alligator_sinensis 9762
Alligator_mississippiensis 9702
Gavialis_gangeticus 9730
Crocodylus_moreletii 9706
Dogania_subplana 9645
Pelomedusa_subrufa 9696
Chrysemys_picta 9702
Chelonia_mydas 9714
Tinamus_major 9687
Smithornis_sharpei 9693
Corvus_frugilegus 9702
Vidua_chalybeata 9702
Buteo_buteo 9702
Falco_peregrinus 9699
Dromaius_novaehollandiae 9693
Struthio_camelus 9699
Apteryx_haastii 9687
Rhea_americana 9699
Gallus_gallus 9696
Ciconia_ciconia 9702
Ciconia_boyciana 9699
Iguana_iguana 9684
Eumeces_egregius 9690
Sphenodon_punctatus -
Sceloporus_occidentalis 9678
Cordylus_warreni 9687
Abronia_graminea 9696
Shinisaurus_crocodilurus 9672
Varanus_komodoensis 9711
Rhineura_floridana 9669
Geocalamus_acutus 9639
Diplometopon_zarudnyi 9657
Amphisbaena_schmidti 9642
Bipes_tridactylus 9642
Bipes_canaliculatus 9654
Bipes_biporus 9696
Anolis_carolinensis 9317
Ophisaurus_attnuatus 9316
Varanus_salvator 9702
Mertensiella_luschani 9708
Xenopus_laevis 9699

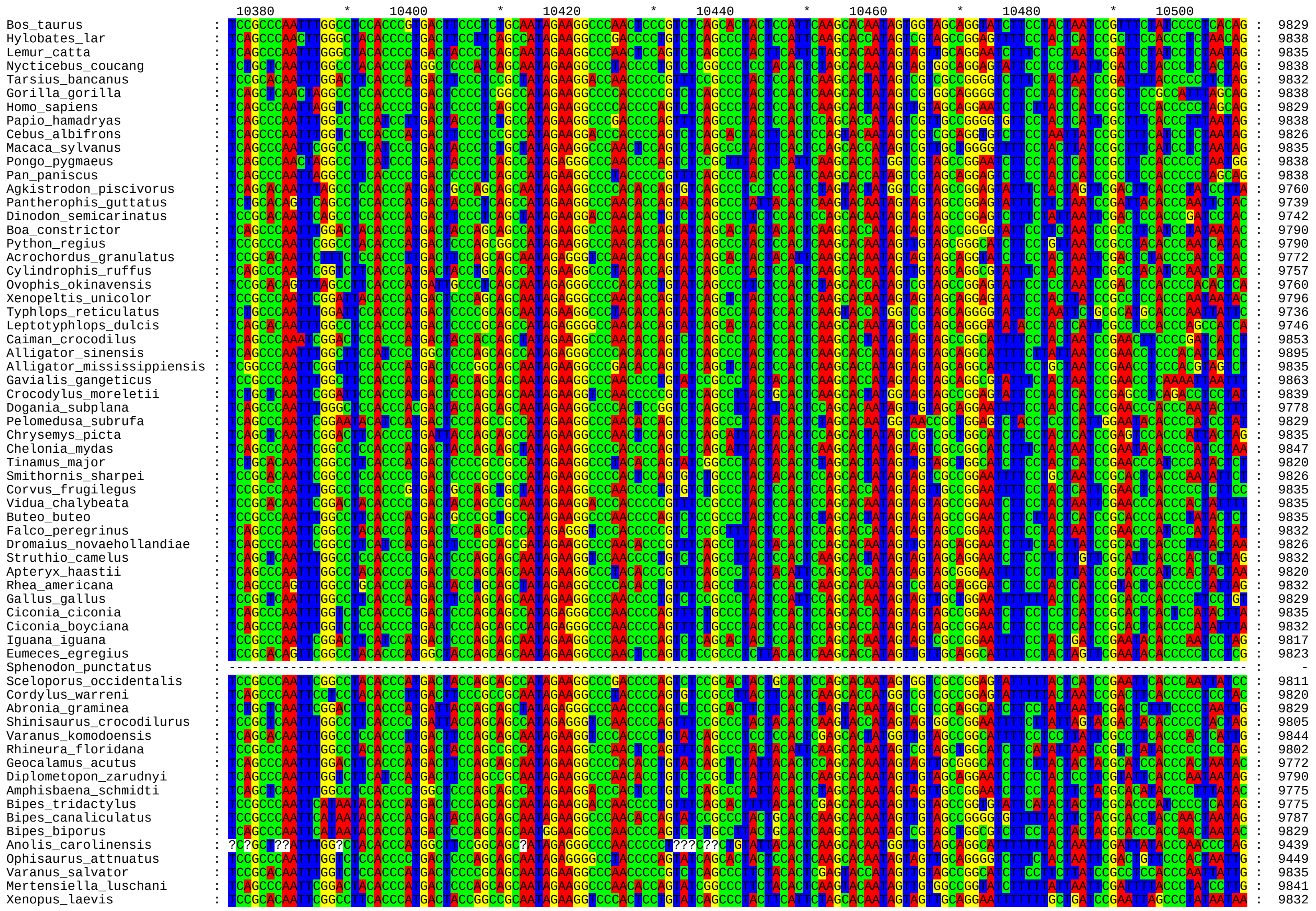
10380 * 10400 * 10420 * 10440 * 10460 * 10480 * 10500
Bos_taurus : 9829
Hylobates_lar : 9838
Lemur_catta : 9835
Nycticebus_coucang : 9838
Tarsius_bancanus : 9832
Gorilla_gorilla : 9838
Homo_sapiens : 9829
Papio_hamadryas : 9838
Cebus_albifrons : 9826
Macaca_sylvanus : 9835
Pongo_pygmaeus : 9838
Pan_paniscus : 9838
Agkistrodon_piscivorus : 9760
Pantherophis_guttatus : 9739
Dinodon_semicarinatus : 9742
Boa_constrictor : 9790
Python_regius : 9790
Acrochordus_granulatus : 9772
Cylindrophis_ruffus : 9757
Ovophis_okinavensis : 9760
Xenopeltis_unicolor : 9796
Typhlops_reticulatus : 9736
Leptotyphlops_dulcis : 9746
Caiman_crocodilus : 9853
Alligator_sinensis : 9895
Alligator_mississippiensis : 9835
Gavialis_gangeticus : 9863
Crocodylus_moreletii : 9839
Dogania_subplana : 9778
Pelomedusa_subrufa : 9829
Chrysemys_picta : 9835
Chelonia_mydas : 9847
Tinamus_major : 9820
Smithornis_sharpei : 9826
Corvus_frugilegus : 9835
Vidua_chalybeata : 9835
Buteo_buteo : 9835
Falco_peregrinus : 9832
Dromaius_novaehollandiae : 9826
Struthio_camelus : 9832
Apteryx_haastii : 9820
Rhea_americana : 9832
Gallus_gallus : 9829
Ciconia_ciconia : 9835
Ciconia_boyciana : 9832
Iguana_iguana : 9817
Eumeces_egregius : 9823
Sphenodon_punctatus : -
Sceloporus_occidentalis : 9811
Cordylus_warreni : 9820
Abronia_graminea : 9829
Shinisaurus_crocodilurus : 9805
Varanus_komodoensis : 9844
Rhineura_floridana : 9802
Geocalamus_acutus : 9772
Diplometopon_zarudnyi : 9790
Amphisbaena_schmidti : 9775
Bipes_tridactylus : 9775
Bipes_canaliculatus : 9787
Bipes_biporus : 9829
Anolis_carolinensis : 9439
Ophisaurus_attenuatus : 9449
Varanus_salvator : 9835
Mertensiella_luschani : 9841
Xenopus_laevis : 9832

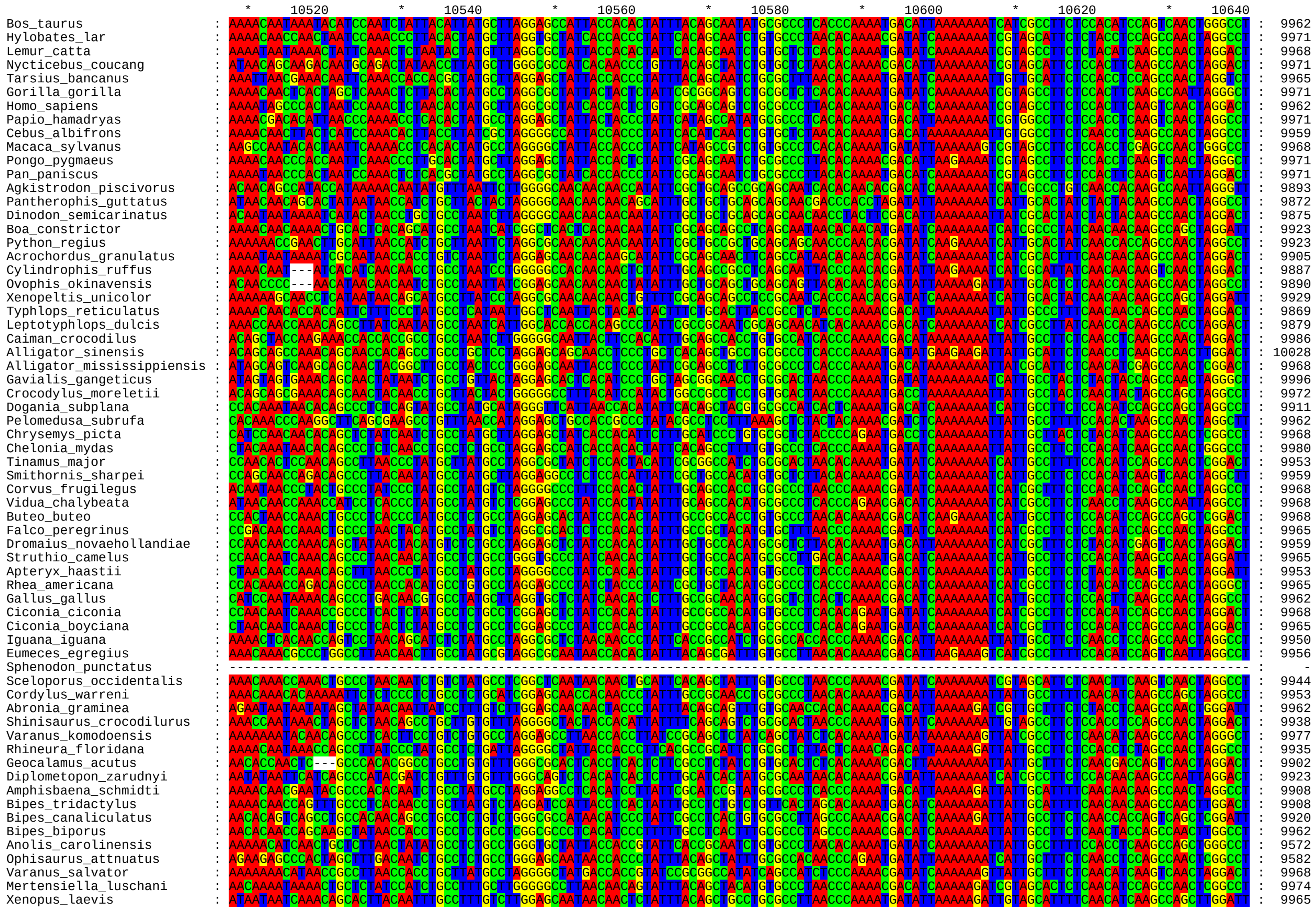
10520
10540
10560
10580
10600
10620
10640
Bos_taurus 9962
Hylobates_lar 9971
Lemur_catta 9968
Nycticebus_coucang 9971
Tarsius_bancanus 9965
Gorilla_gorilla 9971
Homo_sapiens 9962
Papio_hamadryas 9971
Cebus_albifrons 9959
Macaca_sylvanus 9968
Pongo_pygmaeus 9971
Pan_paniscus 9971
Agkistrodon_piscivorus 9893
Pantherophis_guttatus 9872
Dinodon_semicarinatus 9875
Boa_constrictor 9923
Python_regius 9923
Acrochordus_granulatus 9905
Cylindrophis_ruffus 9887
Ovophis_okinavensis 9890
Xenopeltis_unicolor 9929
Typhlops_reticulatus 9869
Leptotyphlops_dulcis 9879
Caiman_crocodilus 9986
Alligator_sinensis 10028
Alligator_mississippiensis 9968
Gavialis_gangeticus 9996
Crocodylus_moreletii 9972
Dogania_subplana 9911
Pelomedusa_subrufa 9962
Chrysemys_picta 9968
Chelonia_mydas 9980
Tinamus_major 9953
Smithornis_sharpei 9959
Corvus_frugilegus 9968
Vidua_chalybeata 9968
Buteo_buteo 9968
Falco_peregrinus 9965
Dromaius_novaehollandiae 9959
Struthio_camelus 9965
Apteryx_haastii 9953
Rhea_americana 9965
Gallus_gallus 9962
Ciconia_ciconia 9968
Ciconia_boyciana 9965
Iguana_iguana 9950
Eumeces_egregius 9956
Sphenodon_punctatus -
Sceloporus_occidentalis 9944
Cordylus_warreni 9953
Abronia_graminea 9962
Shinisaurus_crocodilurus 9938
Varanus_komodoensis 9977
Rhineura_floridana 9935
Geocalamus_acutus 9902
Diplometopon_zarudnyi 9923
Amphisbaena_schmidti 9908
Bipes_tridactylus 9908
Bipes_canaliculatus 9920
Bipes_biporus 9962
Anolis_carolinensis 9572
Ophisaurus_attnuatus 9582
Varanus_salvator 9968
Mertensiella_luschani 9974
Xenopus_laevis 9965

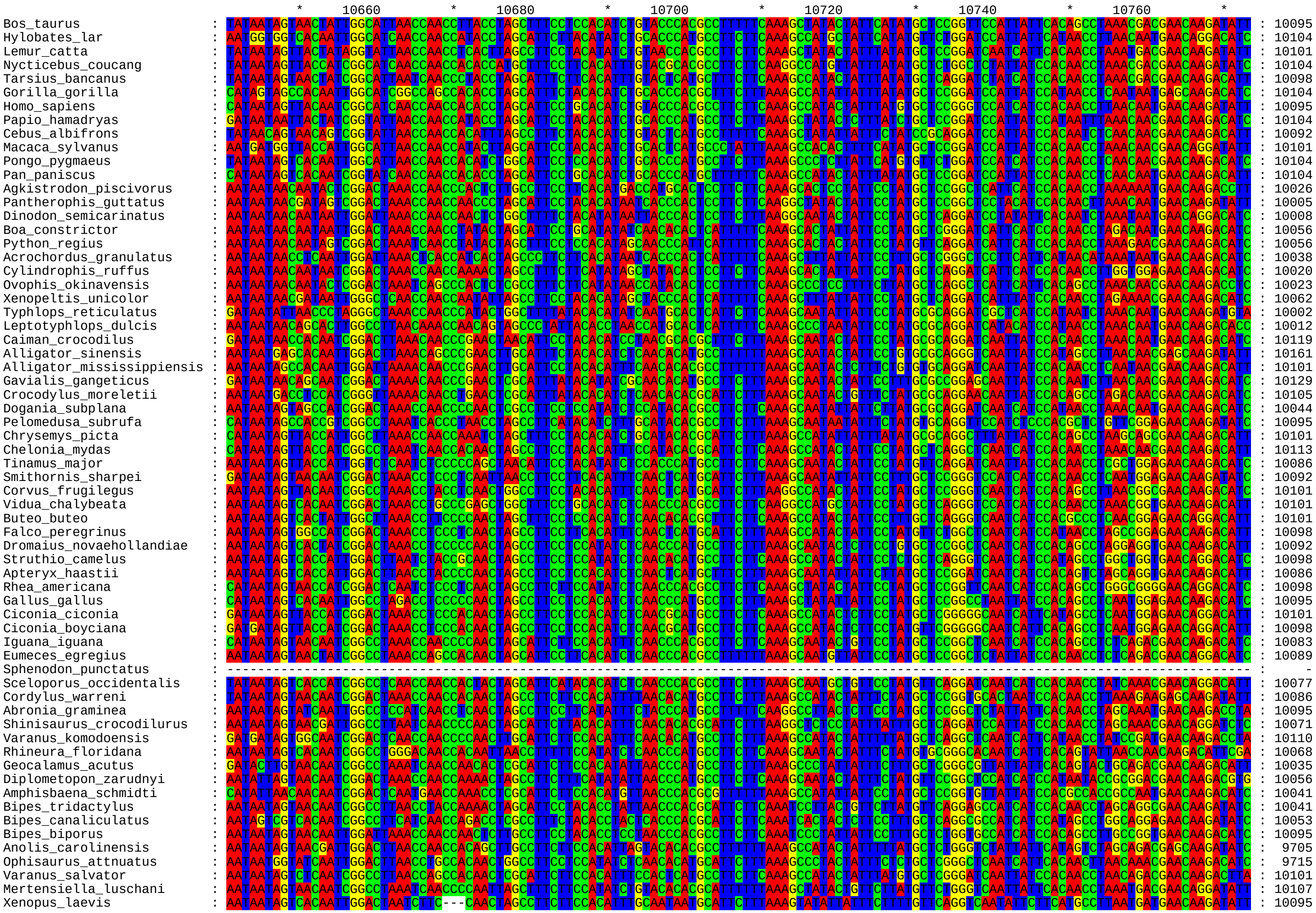

* 10660 * 10680 * 10700 * 10720 * 10740 * 10760 *
Bos_taurus : 10095
Hylobates_lar : 10104
Lemur_catta : 10101
Nycticebus_coucang : 10104
Tarsius_bancanus : 10098
Gorilla_gorilla : 10104
Homo_sapiens : 10095
Papio_hamadryas : 10104
Cebus_albifrons : 10092
Macaca_sylvanus : 10101
Pongo_pygmaeus : 10104
Pan_paniscus : 10104
Agkistrodon_piscivorus : 10026
Pantherophis_guttatus : 10005
Dinodon_semicarinatus : 10008
Boa_constrictor : 10056
Python_regius : 10056
Acrochordus_granulatus : 10038
Cylindrophis_ruffus : 10020
Ovophis_okinavensis : 10023
Xenopeltis_unicolor : 10062
Typhlops_reticulatus : 10002
Leptotyphlops_dulcis : 10012
Caiman_crocodilus : 10119
Alligator_sinensis : 10161
Alligator_mississippiensis : 10101
Gavialis_gangeticus : 10129
Crocodylus_moreletii : 10105
Dogania_subplana : 10044
Pelomedusa_subrufa : 10095
Chrysemys_picta : 10101
Chelonia_mydas : 10113
Tinamus_major : 10086
Smithornis_sharpei : 10092
Corvus_frugilegus : 10101
Vidua_chalybeata : 10101
Buteo_buteo : 10101
Falco_peregrinus : 10098
Dromaius_novaehollandiae : 10092
Struthio_camelus : 10098
Apteryx_haastii : 10086
Rhea_americana : 10098
Gallus_gallus : 10095
Ciconia_ciconia : 10101
Ciconia_boyciana : 10098
Iguana_iguana : 10083
Eumeces_egregius : 10089
Sphenodon_punctatus : -
Sceloporus_occidentalis : 10077
Cordylus_warreni : 10086
Abronia_graminea : 10095
Shinisaurus_crocodilurus : 10071
Varanus_komodoensis : 10110
Rhineura_floridana : 10068
Geocalamus_acutus : 10035
Diplometopon_zarudnyi : 10056
Amphisbaena_schmidti : 10041
Bipes_tridactylus : 10041
Bipes_canaliculatus : 10053
Bipes_biporus : 10095
Anolis_carolinensis : 9705
Ophisaurus_attnuatus : 9715
Varanus_salvator : 10101
Mertensiella_luschani : 10107
Xenopus_laevis : 10095

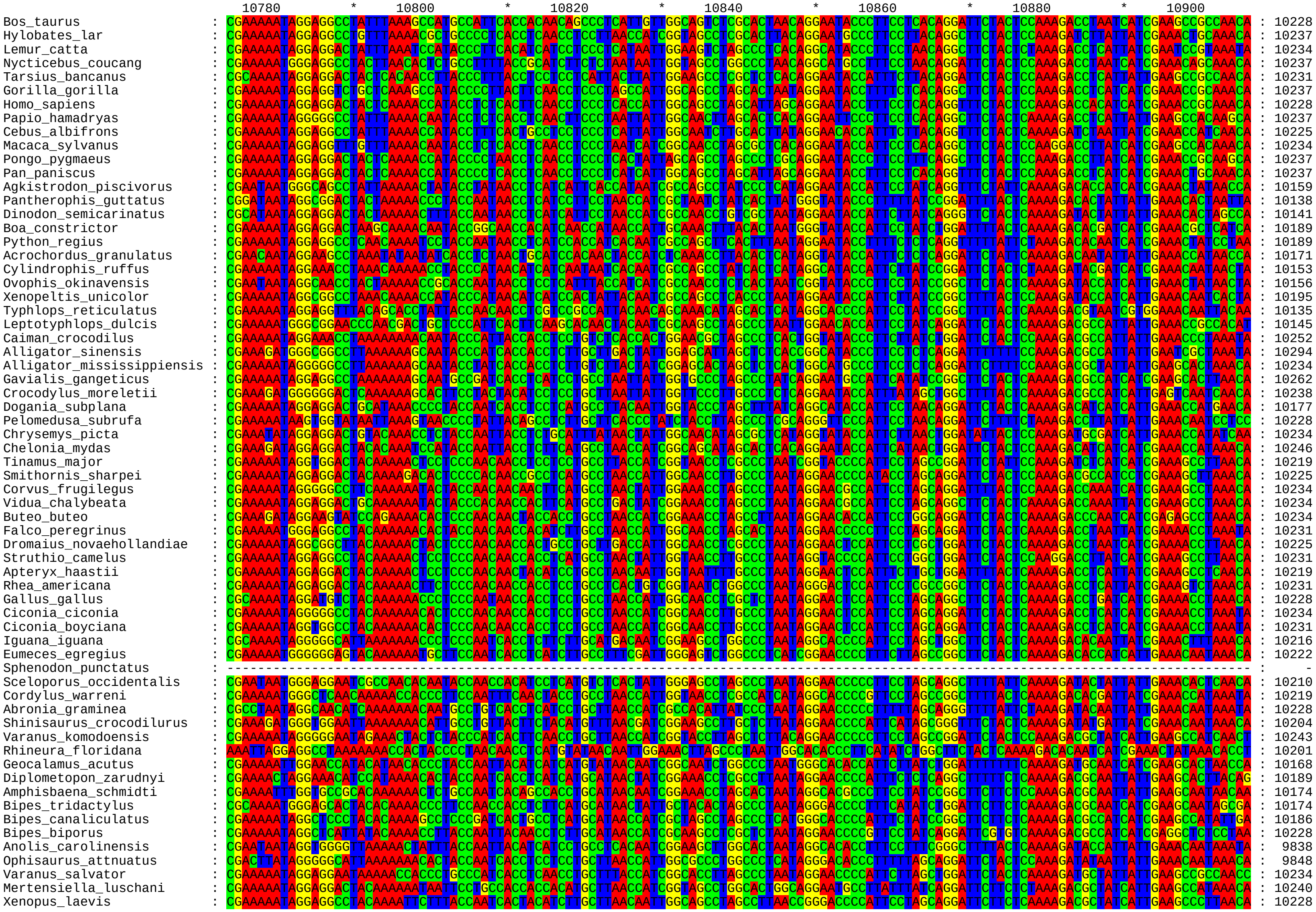
10780 * 10800 * 10820 * 10840 * 10860 * 10880 * 10900
Bos_taurus : 10228
Hylobates_lar : 10237
Lemur_catta : 10234
Nycticebus_coucang : 10237
Tarsius_bancanus : 10231
Gorilla_gorilla : 10237
Homo_sapiens : 10228
Papio_hamadryas : 10237
Cebus_albifrons : 10225
Macaca_sylvanus : 10234
Pongo_pygmaeus : 10237
Pan_paniscus : 10237
Agkistrodon_piscivorus : 10159
Pantherophis_guttatus : 10138
Dinodon_semicarinatus : 10141
Boa_constrictor : 10189
Python_regius : 10189
Acrochordus_granulatus : 10171
Cylindrophis_ruffus : 10153
Ovophis_okinavensis : 10156
Xenopeltis_unicolor : 10195
Typhlops_reticulatus : 10135
Leptotyphlops_dulcis : 10145
Caiman_crocodilus : 10252
Alligator_sinensis : 10294
Alligator_mississippiensis : 10234
Gavialis_gangeticus : 10262
Crocodylus_moreletii : 10238
Dogania_subplana : 10177
Pelomedusa_subrufa : 10228
Chrysemys_picta : 10234
Chelonia_mydas : 10246
Tinamus_major : 10219
Smithornis_sharpei : 10225
Corvus_frugilegus : 10234
Vidua_chalybeata : 10234
Buteo_buteo : 10234
Falco_peregrinus : 10231
Dromaius_novaehollandiae : 10225
Struthio_camelus : 10231
Apteryx_haastii : 10219
Rhea_americana : 10231
Gallus_gallus : 10228
Ciconia_ciconia : 10234
Ciconia_boyciana : 10231
Iguana_iguana : 10216
Eumeces_egregius : 10222
Sphenodon_punctatus : -
Sceloporus_occidentalis : 10210
Cordylus_warreni : 10219
Abronia_graminea : 10228
Shinisaurus_crocodilurus : 10204
Varanus_komodoensis : 10243
Rhineura_floridana : 10201
Geocalamus_acutus : 10168
Diplometopon_zarudnyi : 10189
Amphisbaena_schmidti : 10174
Bipes_tridactylus : 10174
Bipes_canaliculatus : 10186
Bipes_biporus : 10228
Anolis_carolinensis : 9838
Ophisaurus_attenuatus : 9848
Varanus_salvator : 10234
Mertensiella_luschani : 10240
Xenopus_laevis : 10228

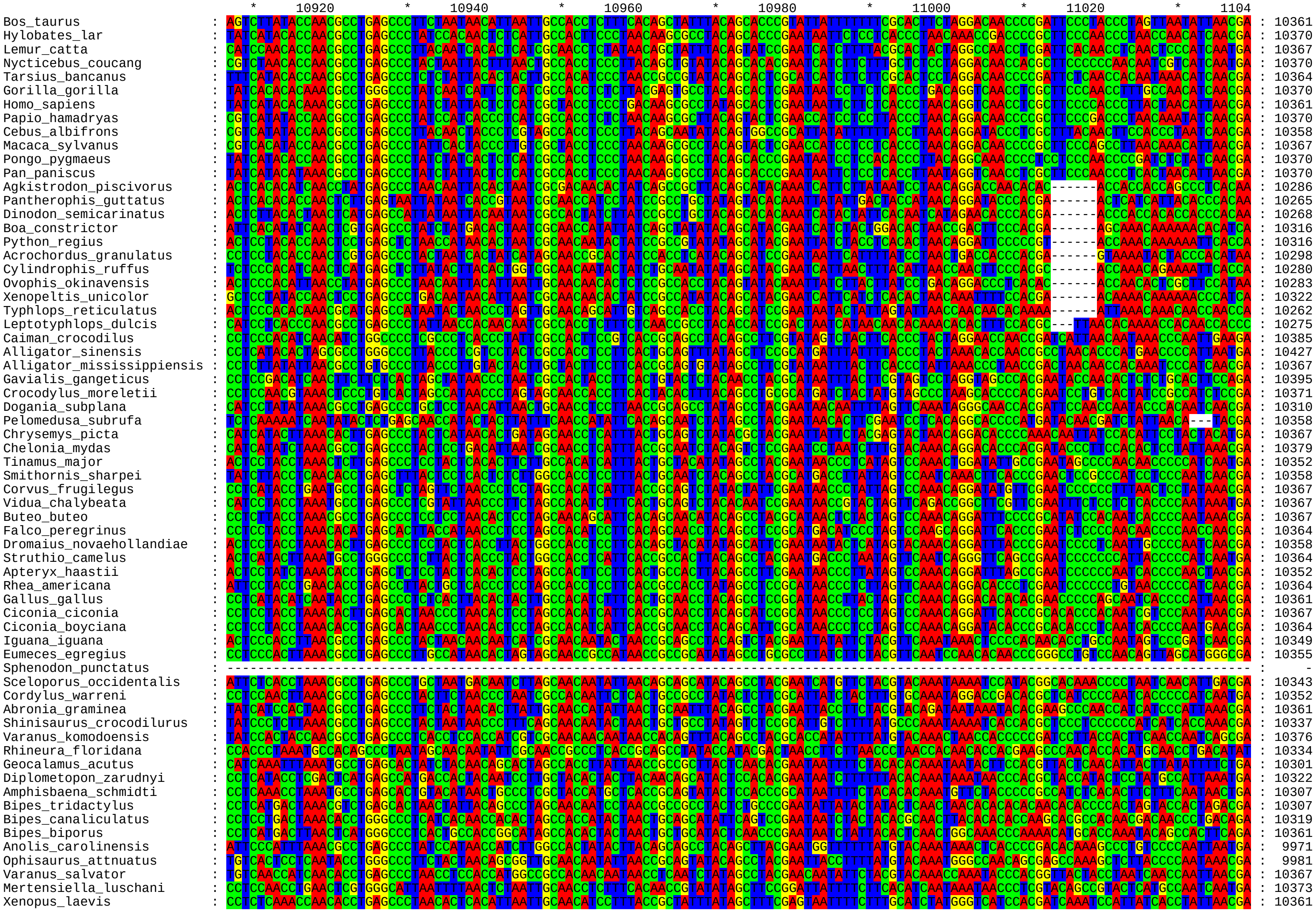

10920
10940
10960
10980
11000
11020
1104
Bos_taurus : 10361
Hylobates_lar : 10370
Lemur_catta : 10367
Nycticebus_coucang : 10370
Tarsius_bancanus : 10364
Gorilla_gorilla : 10370
Homo_sapiens : 10361
Papio_hamadryas : 10370
Cebus_albifrons : 10358
Macaca_sylvanus : 10367
Pongo_pygmaeus : 10370
Pan_paniscus : 10370
Agkistrodon_piscivorus : 10286
Pantherophis_guttatus : 10265
Dinodon_semicarinatus : 10268
Boa_constrictor : 10316
Python_regius : 10316
Acrochordus_granulatus : 10298
Cylindrophis_ruffus : 10280
Ovophis_okinavensis : 10283
Xenopeltis_unicolor : 10322
Typhlops_reticulatus : 10262
Leptotyphlops_dulcis : 10275
Caiman_crocodilus : 10385
Alligator_sinensis : 10427
Alligator_mississippiensis : 10367
Gavialis_gangeticus : 10395
Crocodylus_moreletii : 10371
Dogania_subplana : 10310
Pelomedusa_subrufa : 10358
Chrysemys_picta : 10367
Chelonia_mydas : 10379
Tinamus_major : 10352
Smithornis_sharpei : 10358
Corvus_frugilegus : 10367
Vidua_chalybeata : 10367
Buteo_buteo : 10367
Falco_peregrinus : 10364
Dromaius_novaehollandiae : 10358
Struthio_camelus : 10364
Apteryx_haastii : 10352
Rhea_americana : 10364
Gallus_gallus : 10361
Ciconia_ciconia : 10367
Ciconia_boyciana : 10364
Iguana_iguana : 10349
Eumeces_egregius : 10355
Sphenodon_punctatus : -
Sceloporus_occidentalis : 10343
Cordylus_warreni : 10352
Abronia_graminea : 10361
Shinisaurus_crocodilurus : 10337
Varanus_komodoensis : 10376
Rhineura_floridana : 10334
Geocalamus_acutus : 10301
Diplometopon_zarudnyi : 10322
Amphisbaena_schmidti : 10307
Bipes_tridactylus : 10307
Bipes_canaliculatus : 10319
Bipes_biporus : 10361
Anolis_carolinensis : 9971
Ophisaurus_attnuatus : 9981
Varanus_salvator : 10367
Mertensiella_luschani : 10373
Xenopus_laevis : 10361

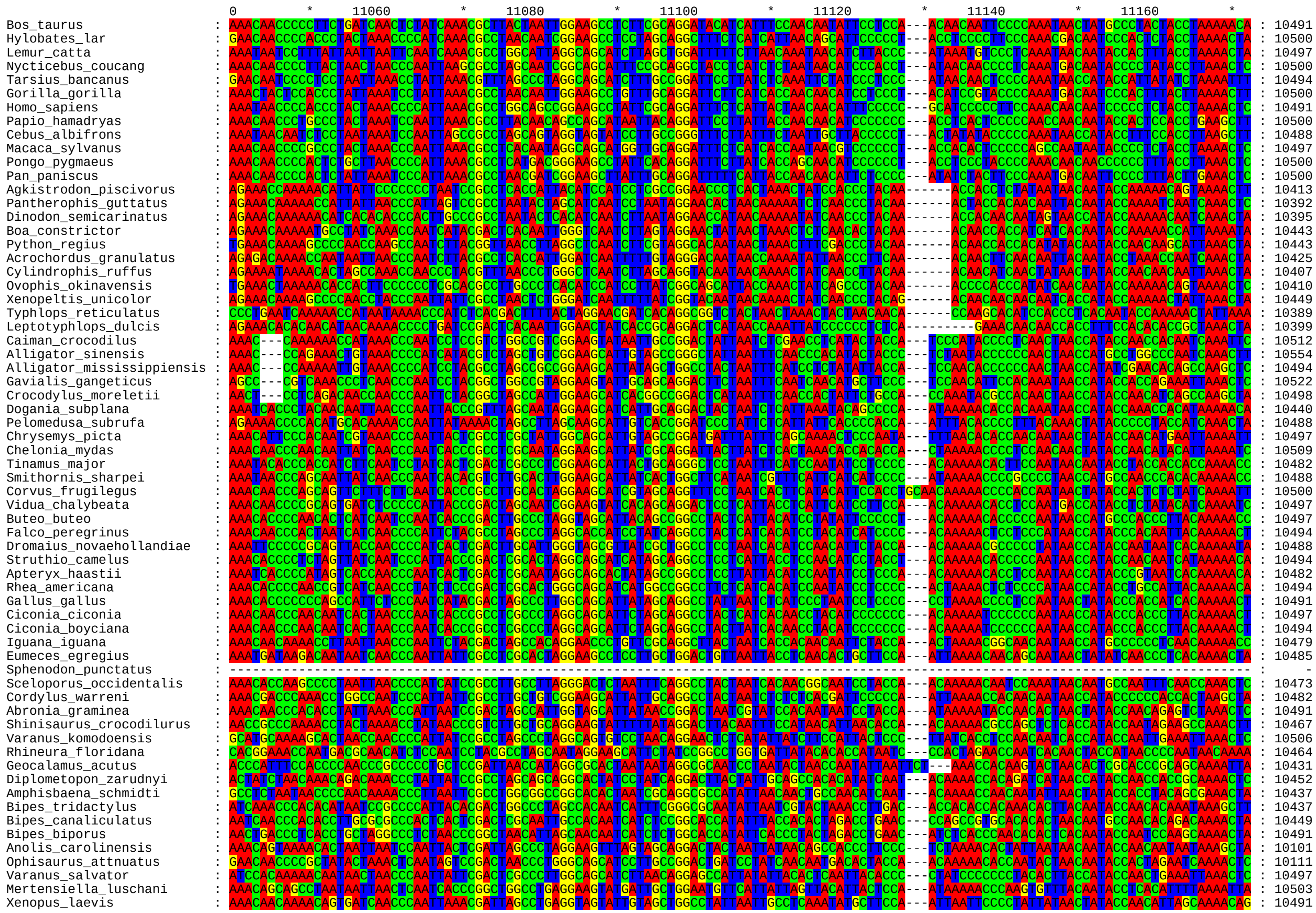
0 * 11060 * 11080 * 11100 * 11120 * 11140 * 11160 *
Bos_taurus : 10491
Hylobates_lar : 10500
Lemur_catta : 10497
Nycticebus_coucang : 10500
Tarsius_bancanus : 10494
Gorilla_gorilla : 10500
Homo_sapiens : 10491
Papio_hamadryas : 10500
Cebus_albifrons : 10488
Macaca_sylvanus : 10497
Pongo_pygmaeus : 10500
Pan_paniscus : 10500
Agkistrodon_piscivorus : 10413
Pantherophis_guttatus : 10392
Dinodon_semicarinatus : 10395
Boa_constrictor : 10443
Python_regius : 10443
Acrochordus_granulatus : 10425
Cylindrophis_ruffus : 10407
Ovophis_okinavensis : 10410
Xenopeltis_unicolor : 10449
Typhlops_reticulatus : 10389
Leptotyphlops_dulcis : 10399
Caiman_crocodilus : 10512
Alligator_sinensis : 10554
Alligator_mississippiensis : 10494
Gavialis_gangeticus : 10522
Crocodylus_moreletii : 10498
Dogania_subplana : 10440
Pelomedusa_subrufa : 10488
Chrysemys_picta : 10497
Chelonia_mydas : 10509
Tinamus_major : 10482
Smithornis_sharpei : 10488
Corvus_frugilegus : 10500
Vidua_chalybeata : 10497
Buteo_buteo : 10497
Falco_peregrinus : 10494
Dromaius_novaehollandiae : 10488
Struthio_camelus : 10494
Apteryx_haastii : 10482
Rhea_americana : 10494
Gallus_gallus : 10491
Ciconia_ciconia : 10497
Ciconia_boyciana : 10494
Iguana_iguana : 10479
Eumeces_egregius : 10485
Sphenodon_punctatus : -
Sceloporus_occidentalis : 10473
Cordylus_warreni : 10482
Abronia_graminea : 10491
Shinisaurus_crocodilurus : 10467
Varanus_komodoensis : 10506
Rhineura_floridana : 10464
Geocalamus_acutus : 10431
Diplometopon_zarudnyi : 10452
Amphisbaena_schmidti : 10437
Bipes_tridactylus : 10437
Bipes_canaliculatus : 10449
Bipes_biporus : 10491
Anolis_carolinensis : 10101
Ophisaurus_attnuatus : 10111
Varanus_salvator : 10497
Mertensiella_luschani : 10503
Xenopus_laevis : 10491

```
                                    11180         *      11200         *      11220         *      11240         *      11260         *      11280         *      11300
Bos_taurus                      : ACAGCCCTAATTGTTACAATCCTAGGCTTCATCTTAGCCCTAGAAATCAGTAATATAACTAAAAATCTAAAATATCACTACCCCTCA------------------------ : 10578
Hylobates_lar                   : ACAGCCTTAAGCATCACCCTCCTAGGCTTCCTAACAGCTTTTGACCTTCATCTCTTGACCAACAAACTTAAAATAAAAAACCCCTCA------------------------ : 10587
Lemur_catta                     : ACAGCTCTAATAGTAACCATTACAGGATTTGCCCTAGCAATAGAACTAAATCTTATAACAAACAACTTAAAATTTAAATTACCTTCA------------------------ : 10584
Nycticebus_coucang              : ACCGCCATAATAATTACAATCCTAGGCTTCTCCCTGGCAATAGAAATAAACTTTATTACTACAAACCTAAAATTTAAGCACGCCCGC------------------------ : 10587
Tarsius_bancanus                : TCTGCACTTCTTATCACTATTCTAGGATTTACACTAGCAATAGAACTCAACAATCTCACATATTTCCTAAAACTAAAACATTACACC------------------------ : 10581
Gorilla_gorilla                 : ACAGCTCTAAGCATCACTCTCCTAGGACTCCTGACAGCCCTAGACCTCAACTACCTCACCAACAAACTCAAAATAAAACACCCACCA------------------------ : 10587
Homo_sapiens                    : ACAGCCCTCGCTGTCACTTTCCTAGGACTTCTAACAGCCCTAGACCTCAACTACCTAACCAACAAACTTAAAATAAAATCCCCACTA------------------------ : 10578
Papio_hamadryas                 : TTAGCCCTATACATAACTGCCCTAGGCTTCATTATCACCCTAGACCTTACCCTCATAACCAACAAATTAAAAGTAAAGACCCCACCA------------------------ : 10587
Cebus_albifrons                 : ACAGCTCTAAGTGTAACCACCCTAGGACTCCTTATAGCAATAGAACTTAACTCTTTAACTAACAACATAAAACTATATACCCCAGTA------------------------ : 10575
Macaca_sylvanus                 : TCGGCCCTGTGCGCAACCACCCTAGGCTTCCTAGCAGCCCTAGATCTTACCCTTATAACAAACAAACTAAAAATAAAAAACCCATCA------------------------ : 10584
Pongo_pygmaeus                  : GCAGCCCTAACTGCCACCCTCCTAGGTCTCCTAGTAGCCCTAGACTTAAACTACCTAGCCAACAAACTCAAAGTAAAAACCCCTCCA------------------------ : 10587
Pan_paniscus                    : ACAGCCCTAAGCGTTACTTTCCTAGGACTTCTAACAGCCCTAGACCTCAATTACCTAACCAACAAACTCAAAATAAAATCCCCACCA------------------------ : 10587
Agkistrodon_piscivorus          : ATAGCACTAACCATAACAGCCCTTGGAGTCATCATATCAAAAGACTTCATACAAGCAACATCACCTTTACCTCCACAAAAACCAAAA------------------------ : 10500
Pantherophis_guttatus           : ATAGCACTAATAGCCACCCTTATAGGCATCATTTTATCAAAAGATTTATCAGACACAACCTACCACATAAAACCAAAAAAACTCAAT------------------------ : 10479
Dinodon_semicarinatus           : ATAGCACTCATCTCTACCATCATTGGAGTTTTACTATCAAAAGACCTCACTCACATAACCCACCATATAAAACCTAAAAAACCTAAC------------------------ : 10482
Boa_constrictor                 : GGTGCACTACTGATCACTATAATGGGTATTATAGTATCAGCAGATTTAATATTTATACACCCCCACCAGCCACCACAAAAACCAAAA------------------------ : 10530
Python_regius                   : TCAGCACTCACCATTACCCTAACAGGAATTATCCTATCAACAGACCTACTATTCCTATCAACCAAACAGCCCCCACAAAAACCAAAA------------------------ : 10530
Acrochordus_granulatus          : ACCACATTAACAATCACTATTATAAGTATAATACTATCCCTAGACATACTATTAATTAATAAATACCTTTCACCAAAAAACCCTAAA------------------------ : 10512
Cylindrophis_ruffus             : AGCGCACTAACAATCACCATCGCAGGCATTCTACTTTCAACAGACCTAATATTTCTATCACCCCACCAACCCCCTAAAAAACCAAAA------------------------ : 10494
Ovophis_okinavensis             : ATGGCACTAACTATAACTATCCTAGGAATCGCCCTATCAAAAGACTTTATACAAACAGCCCTCCCCCTACCCCCACAAAAATCACAA------------------------ : 10497
Xenopeltis_unicolor             : AGCGCACTAATAGTTACCATAGCAGGCATTATACTAGCAATCGACCTGCTCTATTCTACAACACATCAAACACCACAAAAACCAAAA------------------------ : 10536
Typhlops_reticulatus            : CTGGCTGCCATCATAATTACCCTAGCTAGCCTCATCTTAACTACAGACACAATATACTTAAAAAGCCTAAATAAACAAAAATCAACT--------------------AAAC : 10480
Leptotyphlops_dulcis            : GCCGCCCCCACCCTCCTTGTTCTAGCAACAATATGCACACTAGACATCAAAAACCTCTCCATAAAAAACCCCCCCAAAAAACATAAT------------------------ : 10486
Caiman_crocodilus               : ACAGCCTTAGCCATCACAACAGCCGGCCTTCTAACCGCAATAGCCTTAATATTCACCACAGATAAATTCCCATCAACCACAAAAGAC------------------------ : 10599
Alligator_sinensis              : GCAGCCCTCACCATCACAATAGCTGGCCTACTAGTCGCAATAGCCCTGACCTACGCCACCAACAAATTCCCCCCATCCACCAACGAC------------------------ : 10641
Alligator_mississippiensis      : GCAGCCCTTATTATCACAATGATCGGACTACTAATTGCAGCAACCCTAACCCATGCCACCAATAAATTCCCGCCCATTACCAACGAC------------------------ : 10581
Gavialis_gangeticus             : GCTGCCCTGATCGTTACAGCCTTAGGATTCCTGACAGGCGCAGCCTTAATATCCACAGCCCCAACCCTCCAGCCATCCACCAAAGGC------------------------ : 10609
Crocodylus_moreletii            : GCCGCACTAAGTATCCTAATCCTAGGACTACTAGTCGGCTCAATCCTAATTACAGTAGCAGACCAACTTCCCAACTCCACCAAAGGC------------------------ : 10585
Dogania_subplana                : TCAGCATTAACCATAACAATTCTAGGGTTAATCCTAGCTATAGAACTTATCACAATAACAAAC---AAAACTACA---AAACCATCAAAACCC----------------- : 10527
Pelomedusa_subrufa              : GCAGCATTGACCCTCACCTTCATAAGCGCATTCTTAGCAATATACCTAATTAGTCTAGCTAATAATAAACCCCTTCCAAAAAACACC------------------------ : 10575
Chrysemys_picta                 : GCAGCATTTACAGTAACAATCATCGGTATCCTAGTAGGCCTACAACTAACCTCAATAGCTAAT---AAAATACCCATAAAACCTTCCGAAACC----------------- : 10587
Chelonia_mydas                  : GCAGCACTAACAGTAACAATACTAGGCCTTTTAATAGCCCTAGAACTAACTATAATAATAAAC---AAAACAACCCAAAAACCCTCTAACACC----------------- : 10599
Tinamus_major                   : GCCGCCATCCTAGTCACAATCCTAGGTATCCTCCTAGCCCTAGAACTTACTAACCTAACCCACAATCTCACACCCCCTAACCAAAATACCTTA----------------- : 10575
Smithornis_sharpei              : ACCGCCATTGTCATCACAATCCTAGGAGCCATCACAGCGCTAGAACTCTCAAAAATAACTCATTCCTTCCTCAAACCCAAACAAAATATAACT----------------- : 10581
Corvus_frugilegus               : ACAGCCCTCGTAGTAACACTACTAGGAATTATTCTAGCCCTAGAACTCTCAAAAATAACTCAAGCCTTAATCCTCCCTAAACAGAATCGCTTC----------------- : 10593
Vidua_chalybeata                : ACAGCAATTTTAGTGACAGCTCTAGGAATCGCTCTAGCCCTAGAAATATCAAAAATAGCTCAAGCCTATATCCTAACAAAACAAACACCATTC----------------- : 10590
Buteo_buteo                     : GCCGCAATTATCGTCACAATCCTAGGCATTATCCTAGCCCTAGAACTCTCAAACATAACACATACCCTGACCCAACCAAAACAAACCATATAC----------------- : 10590
Falco_peregrinus                : GCAGCTATCCTAGTCACAACCCTAGGCATTATTTTAGCATTAGAACTATCAAACATAACCCACACCCTAACACACCCCAAACAAAACATCTAC----------------- : 10587
Dromaius_novaehollandiae        : GCTGCTATCATTGTTACTATCCTAGGCATCATACTAGCATTAGAACTACTAAACCTAACCCACAGTCTTACCCTCTCCAAACAAAATACCTAC----------------- : 10581
Struthio_camelus                : GCCGCCATCGTCGTCACAATCCTAGGTATCCTCCTAGCACTCGAACTATCAAACATAACTCATAACCTCACGGCCCCTAAACAAAACATCTAC----------------- : 10587
Apteryx_haastii                 : GCCGCTATCATCGTCACTATCTTAGGCATTCTCTTAGCTCTAGAACTATCAAACATAACCCACAACCTCATCCCCCCTAAACAAAGCACCTAC----------------- : 10575
Rhea_americana                  : GCTGCCATCCTCGTCACAATTCTCGGCATTCTCTTATCTCTAGAACTATCAAACATAACACACAACCTCACCCTTCCCAAACAAAACATCTAT----------------- : 10587
Gallus_gallus                   : GCTGCCATCATTGTTACAACCCTAGGAATTATCCTAGCCCTAGAACTCTCAAGCCTATCATACTCCCTCACTCCCCCAAAACACAATCCCCTC----------------- : 10584
Ciconia_ciconia                 : GCAGCCATCATCGTTACAATCCTAGGAATTCTCCTAGCCCTAGAGCTATCAAATATAGCCCATGCTCTAACCCAACCCAAGCAAAGCATCCCC----------------- : 10590
Ciconia_boyciana                : GCAGCCATCATCGTTACAATCCTAGGAATTCTCCTAGCCCTAGAGCTATCAAATATAGCCCATACCCTCACCCAACCAAAGCAAAGCATCCCC----------------- : 10587
Iguana_iguana                   : CTAGCACTAATTATTACCCTCATCGGACTCACCCTTGCCTTAGACATTACCAACCAAACCTCCACACTCCGCCCCCAAAAACATCCT------------------------ : 10566
Eumeces_egregius                : TCAGCCCTACTCGTAACAGTCTTCGGCCTCGTATATGCCTTAGAACTAGCTACTCAAACAACAACATTAGTGGCCCCAAAACACAAT------------------------ : 10572
Sphenodon_punctatus             : ---------------------------------------------------------------------------------------------------------------- :     -
Sceloporus_occidentalis         : ATAGCTATCTCTATTACAGTTATTGGACTCATATTAGCACTAGATATCTCAAACCAAACCACCACACTCATGCCAACAAAACAAACA------------------------ : 10560
Cordylus_warreni                : TCCGCACTACTCATCACAATCCTAGGACTAATCATTGCCATAGAACTAACAACCAAAACAACCCTTACACAAAACCCATCCAACAAA------------------------ : 10569
Abronia_graminea                : TTAGCCCTTCTTGTAACACTCGTAGGACTCATAACTGCCATAGAAATTTCTAATCAAACAACCCTGGCCATCCAACATAAACCCTCA------------------------ : 10578
Shinisaurus_crocodilurus        : GCAGCACTCATAATTACTATCCTAGGATTACTTTATGCCCTAGAACTAGCCACTCAGACCACATCAATACTCTCATGAAAGCCCTCA------------------------ : 10554
Varanus_komodoensis             : TCCGCCCTAATCGCCTCTATCCTAGGCCTTCTAATTGCACTCGAACTAATAACCCTCTCCACCCACATAACACAACAAAAATCAGCACCT---------------------- : 10596
Rhineura_floridana              : CTATCTGCTATATTAGTTACCATTATTGGCCTTATTACTGCCATAGAACTAACTAAACTCACCACTAAATGAATTCGACCAAACACC------------------------ : 10551
Geocalamus_acutus               : CTTGCACTTATACTTGCACTGCTAGGGGCTACAATAATTATACAGCTAGTCATAATAATAAAAAATTATTCACCACAACAAAACACACCT---------------------- : 10521
Diplometopon_zarudnyi           : CTCGCAGTCTCACTAATAATTTTAGGCATCTCCATAACAATTCAACTAATAACTCTAACAAACAAATATTACTATCCACAGAACCCA------------------------ : 10539
Amphisbaena_schmidti            : CTTGCACTCACTCTTGCACTACTAGGCATAACCCTAACACTACAACTAATACACACAACAACTTCCTACAAAATACAAAAAATACCC------------------------ : 10524
Bipes_tridactylus               : TTAGCACTTACCTTAGCACTACTCGGACTAACAGCTACAATCCACCTAACTCTCACACCTCGCCCACCAGTACAAAACTCTACAACA------------------------ : 10524
Bipes_canaliculatus             : ACCGCCATCACACTGGCAGCACTAGGACTAGCCACCACCCTTCACTTGACCACAACACCAAAACGCCCAATGCAAAACAACCCAACA------------------------ : 10536
Bipes_biporus                   : ACAGCTTTAACCCTGGCTCTCTTAGGAGCAGCTATCACCATCCACCTAGCTCTTACGCCAACACTACCAGTACAGGCCCGCCCTTCG------------------------ : 10578
Anolis_carolinensis             : CTAGCACTGATTATTACTATTTAGGCATCATTATTACAATAGATACCATAAATTGAACTACAACACTCCCTATAAAAACCCCCAAC------------------------ : 10188
Ophisaurus_attnuatus            : CTGGCCCTAATAGTCACAGTAGCCGGACTAGCAGTGGCCCTAGAAATTGCCAAACAAACATCCTTAGCTGTACCACATAAGTCAACT------------------------ : 10198
Varanus_salvator                : TCAGCAATCACTGCTACTATCCTAGGCTTCTTAACCGCGCTAGAACTAATAGCCCTATCCACCAACATAACCCAGCAAAAACCAGTC------------------------ : 10584
Mertensiella_luschani           : TCAGCGGTGTTAGTAGCCCTTACAGGATTGTTTATTGCTATAGACCTATCAAAATTTTCTAAAAAA------------CAAAAATCTACC---------------------- : 10581
Xenopus_laevis                  : GCAGCTATTATCGTTTCGGTTACAGGATTAATCATTGCAATAGACCTTTCTAAGTTAACAACCTACATTAACCAAGAATCAAAAACAAAT---------------------- : 10581
```

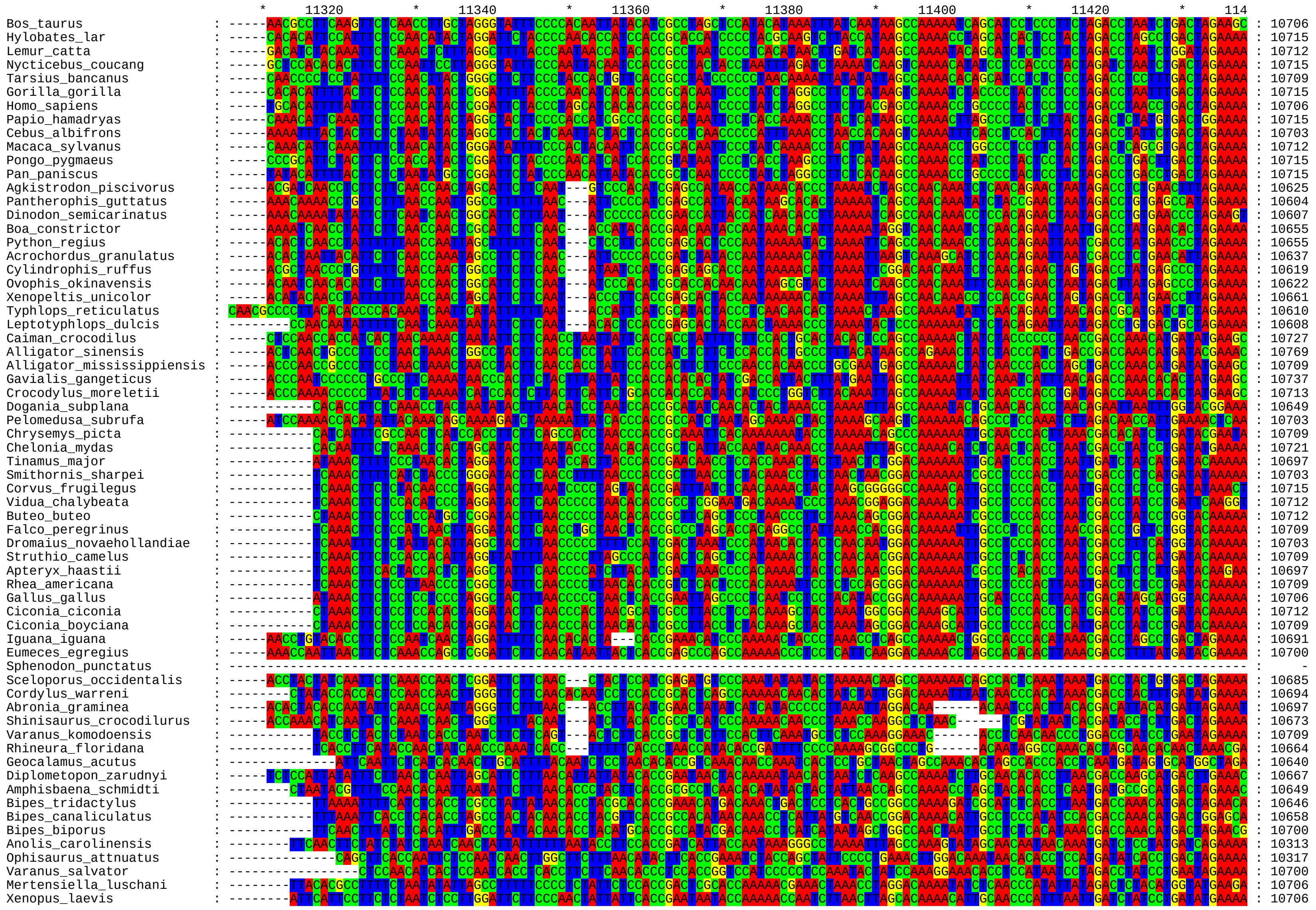
* 11320 * 11340 * 11360 * 11380 * 11400 * 11420 * 114
Bos_taurus : 10706
Hylobates_lar : 10715
Lemur_catta : 10712
Nycticebus_coucang : 10715
Tarsius_bancanus : 10709
Gorilla_gorilla : 10715
Homo_sapiens : 10706
Papio_hamadryas : 10715
Cebus_albifrons : 10703
Macaca_sylvanus : 10712
Pongo_pygmaeus : 10715
Pan_paniscus : 10715
Agkistrodon_piscivorus : 10625
Pantherophis_guttatus : 10604
Dinodon_semicarinatus : 10607
Boa_constrictor : 10655
Python_regius : 10655
Acrochordus_granulatus : 10637
Cylindrophis_ruffus : 10619
Ovophis_okinavensis : 10622
Xenopeltis_unicolor : 10661
Typhlops_reticulatus : 10610
Leptotyphlops_dulcis : 10608
Caiman_crocodilus : 10727
Alligator_sinensis : 10769
Alligator_mississippiensis : 10709
Gavialis_gangeticus : 10737
Crocodylus_moreletii : 10713
Dogania_subplana : 10649
Pelomedusa_subrufa : 10703
Chrysemys_picta : 10709
Chelonia_mydas : 10721
Tinamus_major : 10697
Smithornis_sharpei : 10703
Corvus_frugilegus : 10715
Vidua_chalybeata : 10712
Buteo_buteo : 10712
Falco_peregrinus : 10709
Dromaius_novaehollandiae : 10703
Struthio_camelus : 10709
Apteryx_haastii : 10697
Rhea_americana : 10709
Gallus_gallus : 10706
Ciconia_ciconia : 10712
Ciconia_boyciana : 10709
Iguana_iguana : 10691
Eumeces_egregius : 10700
Sphenodon_punctatus : -
Sceloporus_occidentalis : 10685
Cordylus_warreni : 10694
Abronia_graminea : 10697
Shinisaurus_crocodilurus : 10673
Varanus_komodoensis : 10709
Rhineura_floridana : 10664
Geocalamus_acutus : 10640
Diplometopon_zarudnyi : 10667
Amphisbaena_schmidti : 10649
Bipes_tridactylus : 10646
Bipes_canaliculatus : 10658
Bipes_biporus : 10700
Anolis_carolinensis : 10313
Ophisaurus_attnuatus : 10317
Varanus_salvator : 10700
Mertensiella_luschani : 10706
Xenopus_laevis : 10706

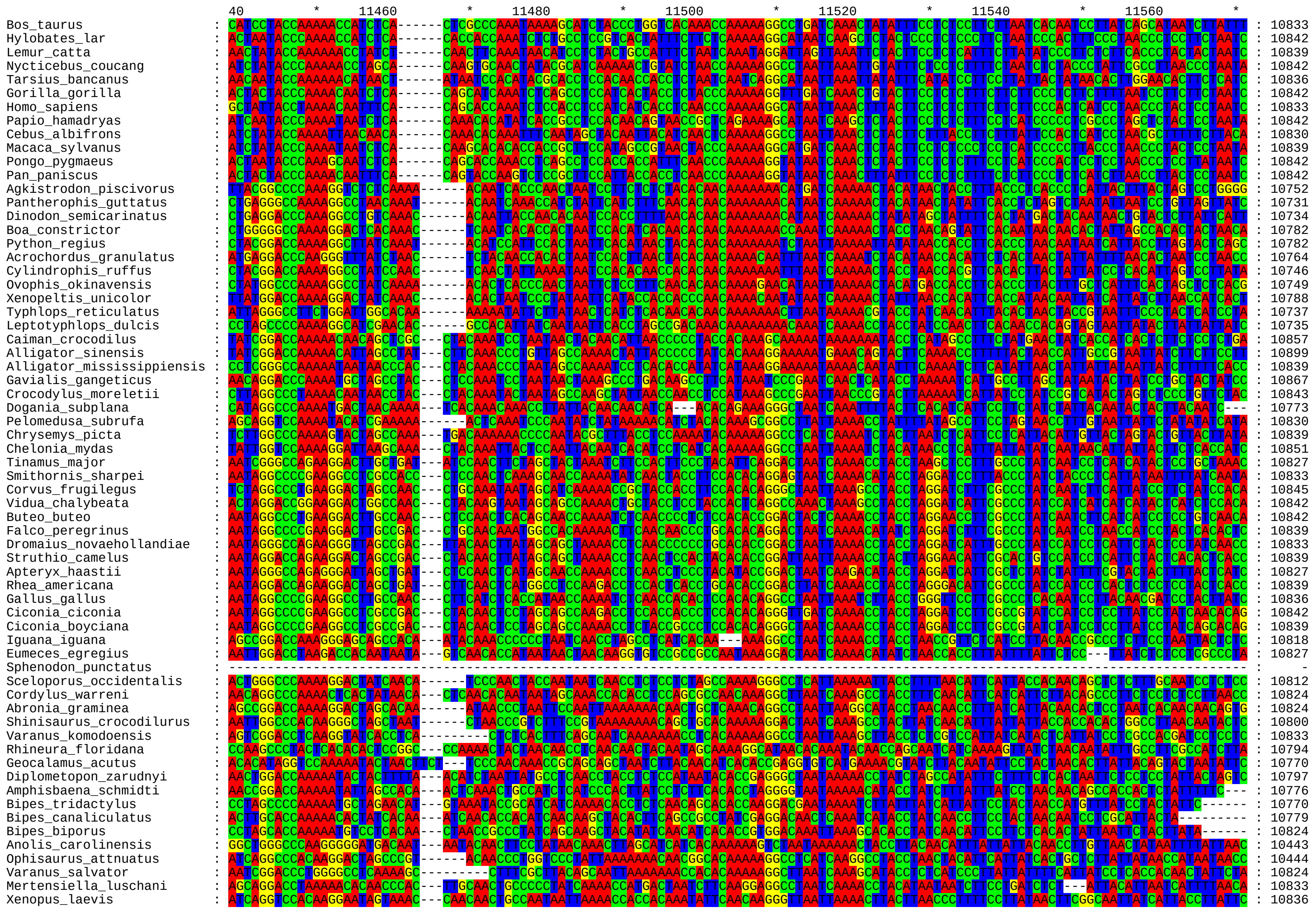
40 * 11460 * 11480 * 11500 * 11520 * 11540 * 11560 *
Bos_taurus : 10833
Hylobates_lar : 10842
Lemur_catta : 10839
Nycticebus_coucang : 10842
Tarsius_bancanus : 10836
Gorilla_gorilla : 10842
Homo_sapiens : 10833
Papio_hamadryas : 10842
Cebus_albifrons : 10830
Macaca_sylvanus : 10839
Pongo_pygmaeus : 10842
Pan_paniscus : 10842
Agkistrodon_piscivorus : 10752
Pantherophis_guttatus : 10731
Dinodon_semicarinatus : 10734
Boa_constrictor : 10782
Python_regius : 10782
Acrochordus_granulatus : 10764
Cylindrophis_ruffus : 10746
Ovophis_okinavensis : 10749
Xenopeltis_unicolor : 10788
Typhlops_reticulatus : 10737
Leptotyphlops_dulcis : 10735
Caiman_crocodilus : 10857
Alligator_sinensis : 10899
Alligator_mississippiensis : 10839
Gavialis_gangeticus : 10867
Crocodylus_moreletii : 10843
Dogania_subplana : 10773
Pelomedusa_subrufa : 10830
Chrysemys_picta : 10839
Chelonia_mydas : 10851
Tinamus_major : 10827
Smithornis_sharpei : 10833
Corvus_frugilegus : 10845
Vidua_chalybeata : 10842
Buteo_buteo : 10842
Falco_peregrinus : 10839
Dromaius_novaehollandiae : 10833
Struthio_camelus : 10839
Apteryx_haastii : 10827
Rhea_americana : 10839
Gallus_gallus : 10836
Ciconia_ciconia : 10842
Ciconia_boyciana : 10839
Iguana_iguana : 10818
Eumeces_egregius : 10827
Sphenodon_punctatus : -
Sceloporus_occidentalis : 10812
Cordylus_warreni : 10824
Abronia_graminea : 10824
Shinisaurus_crocodilurus : 10800
Varanus_komodoensis : 10833
Rhineura_floridana : 10794
Geocalamus_acutus : 10770
Diplometopon_zarudnyi : 10797
Amphisbaena_schmidti : 10776
Bipes_tridactylus : 10770
Bipes_canaliculatus : 10779
Bipes_biporus : 10824
Anolis_carolinensis : 10443
Ophisaurus_attnuatus : 10444
Varanus_salvator : 10824
Mertensiella_luschani : 10833
Xenopus_laevis : 10836

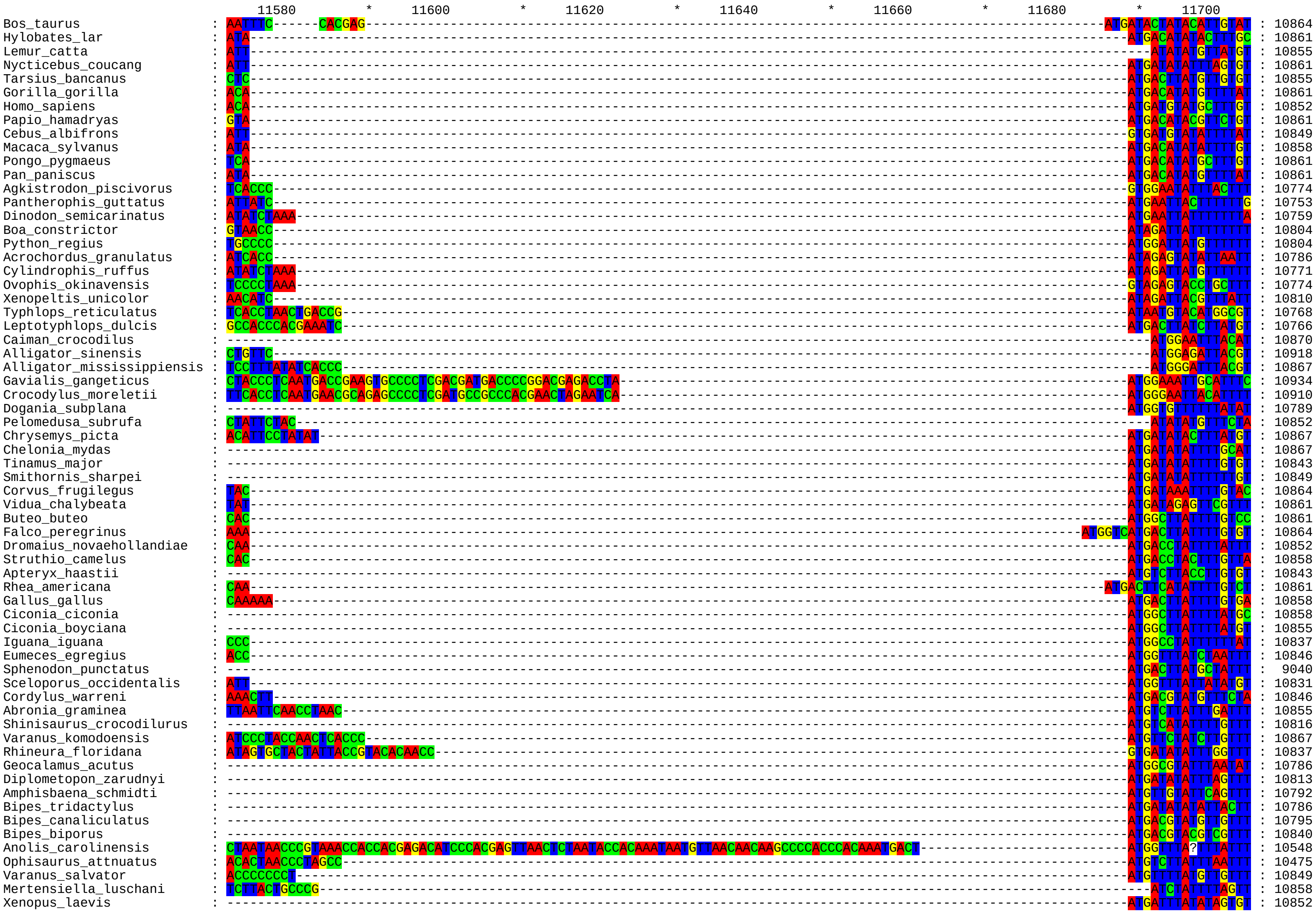

11580 * 11600 * 11620 * 11640 * 11660 * 11680 * 11700
Bos_taurus : 10864
Hylobates_lar : 10861
Lemur_catta : 10855
Nycticebus_coucang : 10861
Tarsius_bancanus : 10855
Gorilla_gorilla : 10861
Homo_sapiens : 10852
Papio_hamadryas : 10861
Cebus_albifrons : 10849
Macaca_sylvanus : 10858
Pongo_pygmaeus : 10861
Pan_paniscus : 10861
Agkistrodon_piscivorus : 10774
Pantherophis_guttatus : 10753
Dinodon_semicarinatus : 10759
Boa_constrictor : 10804
Python_regius : 10804
Acrochordus_granulatus : 10786
Cylindrophis_ruffus : 10771
Ovophis_okinavensis : 10774
Xenopeltis_unicolor : 10810
Typhlops_reticulatus : 10768
Leptotyphlops_dulcis : 10766
Caiman_crocodilus : 10870
Alligator_sinensis : 10918
Alligator_mississippiensis : 10867
Gavialis_gangeticus : 10934
Crocodylus_moreletii : 10910
Dogania_subplana : 10789
Pelomedusa_subrufa : 10852
Chrysemys_picta : 10867
Chelonia_mydas : 10867
Tinamus_major : 10843
Smithornis_sharpei : 10849
Corvus_frugilegus : 10864
Vidua_chalybeata : 10861
Buteo_buteo : 10861
Falco_peregrinus : 10864
Dromaius_novaehollandiae : 10852
Struthio_camelus : 10858
Apteryx_haastii : 10843
Rhea_americana : 10861
Gallus_gallus : 10858
Ciconia_ciconia : 10858
Ciconia_boyciana : 10855
Iguana_iguana : 10837
Eumeces_egregius : 10846
Sphenodon_punctatus : 9040
Sceloporus_occidentalis : 10831
Cordylus_warreni : 10846
Abronia_graminea : 10855
Shinisaurus_crocodilurus : 10816
Varanus_komodoensis : 10867
Rhineura_floridana : 10837
Geocalamus_acutus : 10786
Diplometopon_zarudnyi : 10813
Amphisbaena_schmidti : 10792
Bipes_tridactylus : 10786
Bipes_canaliculatus : 10795
Bipes_biporus : 10840
Anolis_carolinensis : 10548
Ophisaurus_attnuatus : 10475
Varanus_salvator : 10849
Mertensiella_luschani : 10858
Xenopus_laevis : 10852

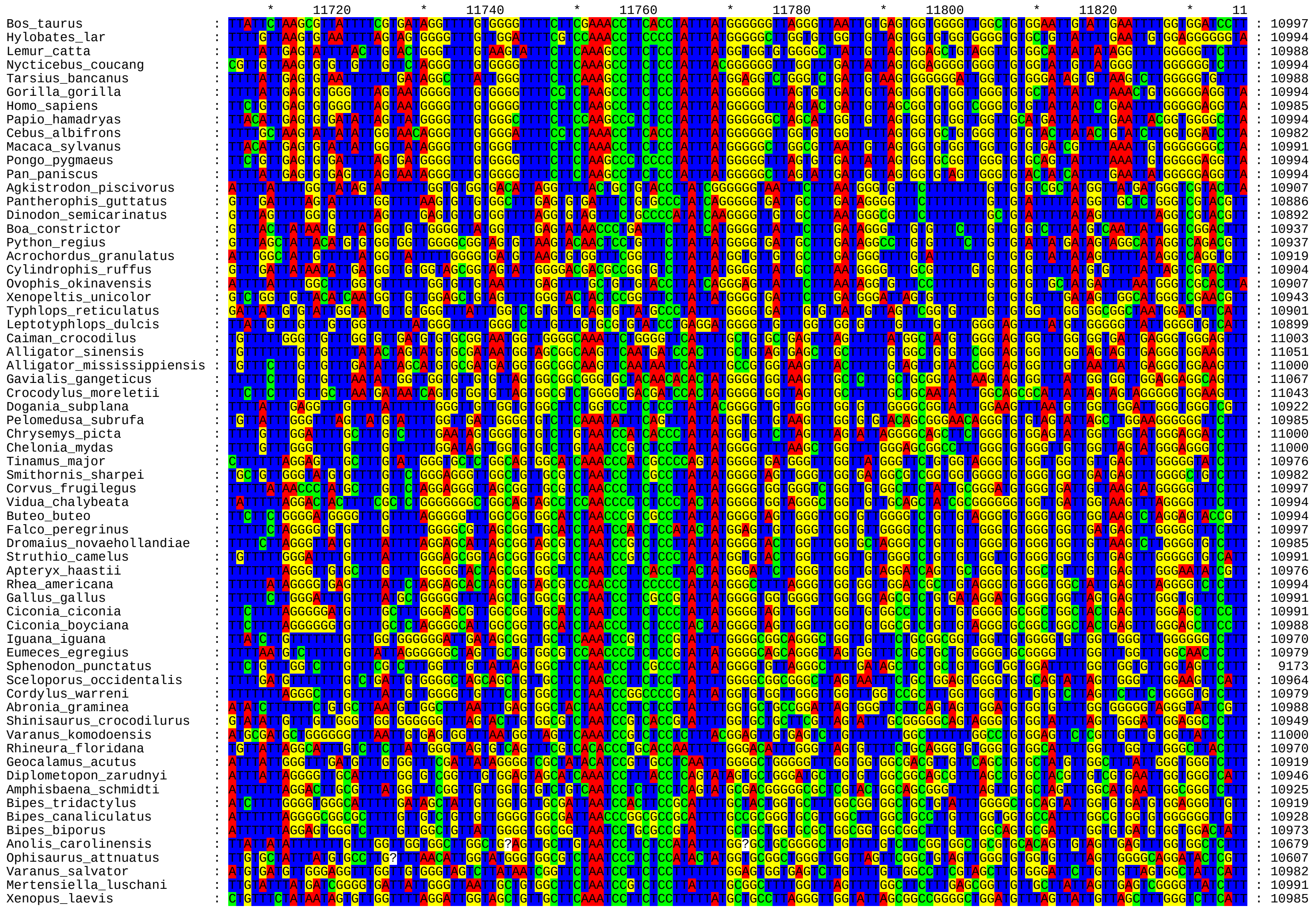
* 11720 * 11740 * 11760 * 11780 * 11800 * 11820 * 11
Bos_taurus : 10997
Hylobates_lar : 10994
Lemur_catta : 10988
Nycticebus_coucang : 10994
Tarsius_bancanus : 10988
Gorilla_gorilla : 10994
Homo_sapiens : 10985
Papio_hamadryas : 10994
Cebus_albifrons : 10982
Macaca_sylvanus : 10991
Pongo_pygmaeus : 10994
Pan_paniscus : 10994
Agkistrodon_piscivorus : 10907
Pantherophis_guttatus : 10886
Dinodon_semicarinatus : 10892
Boa_constrictor : 10937
Python_regius : 10937
Acrochordus_granulatus : 10919
Cylindrophis_ruffus : 10904
Ovophis_okinavensis : 10907
Xenopeltis_unicolor : 10943
Typhlops_reticulatus : 10901
Leptotyphlops_dulcis : 10899
Caiman_crocodilus : 11003
Alligator_sinensis : 11051
Alligator_mississippiensis : 11000
Gavialis_gangeticus : 11067
Crocodylus_moreletii : 11043
Dogania_subplana : 10922
Pelomedusa_subrufa : 10985
Chrysemys_picta : 11000
Chelonia_mydas : 11000
Tinamus_major : 10976
Smithornis_sharpei : 10982
Corvus_frugilegus : 10997
Vidua_chalybeata : 10994
Buteo_buteo : 10994
Falco_peregrinus : 10997
Dromaius_novaehollandiae : 10985
Struthio_camelus : 10991
Apteryx_haastii : 10976
Rhea_americana : 10994
Gallus_gallus : 10991
Ciconia_ciconia : 10991
Ciconia_boyciana : 10988
Iguana_iguana : 10970
Eumeces_egregius : 10979
Sphenodon_punctatus : 9173
Sceloporus_occidentalis : 10964
Cordylus_warreni : 10979
Abronia_graminea : 10988
Shinisaurus_crocodilurus : 10949
Varanus_komodoensis : 11000
Rhineura_floridana : 10970
Geocalamus_acutus : 10919
Diplometopon_zarudnyi : 10946
Amphisbaena_schmidti : 10925
Bipes_tridactylus : 10919
Bipes_canaliculatus : 10928
Bipes_biporus : 10973
Anolis_carolinensis : 10679
Ophisaurus_attnuatus : 10607
Varanus_salvator : 10982
Mertensiella_luschani : 10991
Xenopus_laevis : 10985

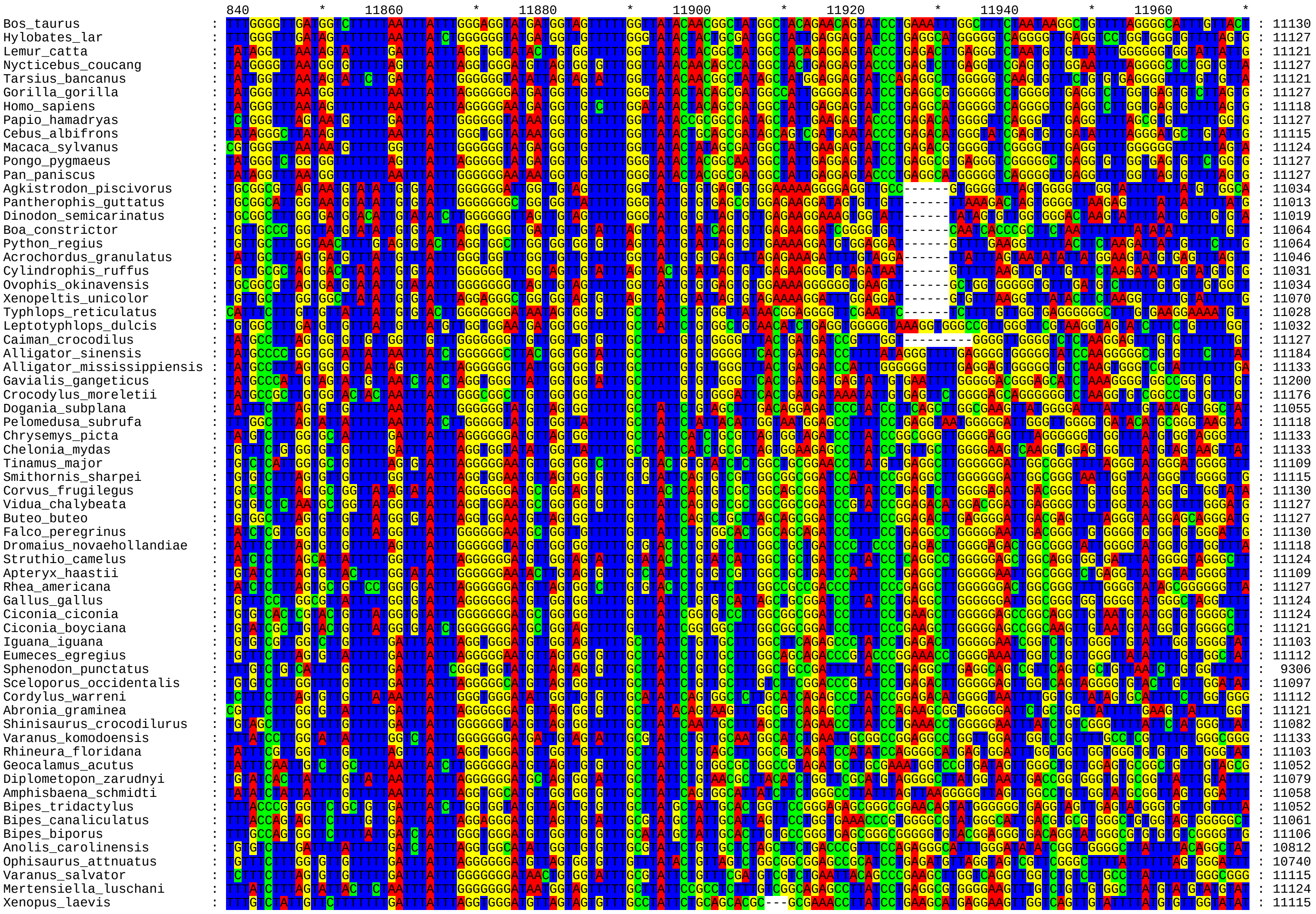
840 * 11860 * 11880 * 11900 * 11920 * 11940 * 11960 *
Bos_taurus : 11130
Hylobates_lar : 11127
Lemur_catta : 11121
Nycticebus_coucang : 11127
Tarsius_bancanus : 11121
Gorilla_gorilla : 11127
Homo_sapiens : 11118
Papio_hamadryas : 11127
Cebus_albifrons : 11115
Macaca_sylvanus : 11124
Pongo_pygmaeus : 11127
Pan_paniscus : 11127
Agkistrodon_piscivorus : 11034
Pantherophis_guttatus : 11013
Dinodon_semicarinatus : 11019
Boa_constrictor : 11064
Python_regius : 11064
Acrochordus_granulatus : 11046
Cylindrophis_ruffus : 11031
Ovophis_okinavensis : 11034
Xenopeltis_unicolor : 11070
Typhlops_reticulatus : 11028
Leptotyphlops_dulcis : 11032
Caiman_crocodilus : 11127
Alligator_sinensis : 11184
Alligator_mississippiensis : 11133
Gavialis_gangeticus : 11200
Crocodylus_moreletii : 11176
Dogania_subplana : 11055
Pelomedusa_subrufa : 11118
Chrysemys_picta : 11133
Chelonia_mydas : 11133
Tinamus_major : 11109
Smithornis_sharpei : 11115
Corvus_frugilegus : 11130
Vidua_chalybeata : 11127
Buteo_buteo : 11127
Falco_peregrinus : 11130
Dromaius_novaehollandiae : 11118
Struthio_camelus : 11124
Apteryx_haastii : 11109
Rhea_americana : 11127
Gallus_gallus : 11124
Ciconia_ciconia : 11124
Ciconia_boyciana : 11121
Iguana_iguana : 11103
Eumeces_egregius : 11112
Sphenodon_punctatus : 9306
Sceloporus_occidentalis : 11097
Cordylus_warreni : 11112
Abronia_graminea : 11121
Shinisaurus_crocodilurus : 11082
Varanus_komodoensis : 11133
Rhineura_floridana : 11103
Geocalamus_acutus : 11052
Diplometopon_zarudnyi : 11079
Amphisbaena_schmidti : 11058
Bipes_tridactylus : 11052
Bipes_canaliculatus : 11061
Bipes_biporus : 11106
Anolis_carolinensis : 10812
Ophisaurus_attnuatus : 10740
Varanus_salvator : 11115
Mertensiella_luschani : 11124
Xenopus_laevis : 11115

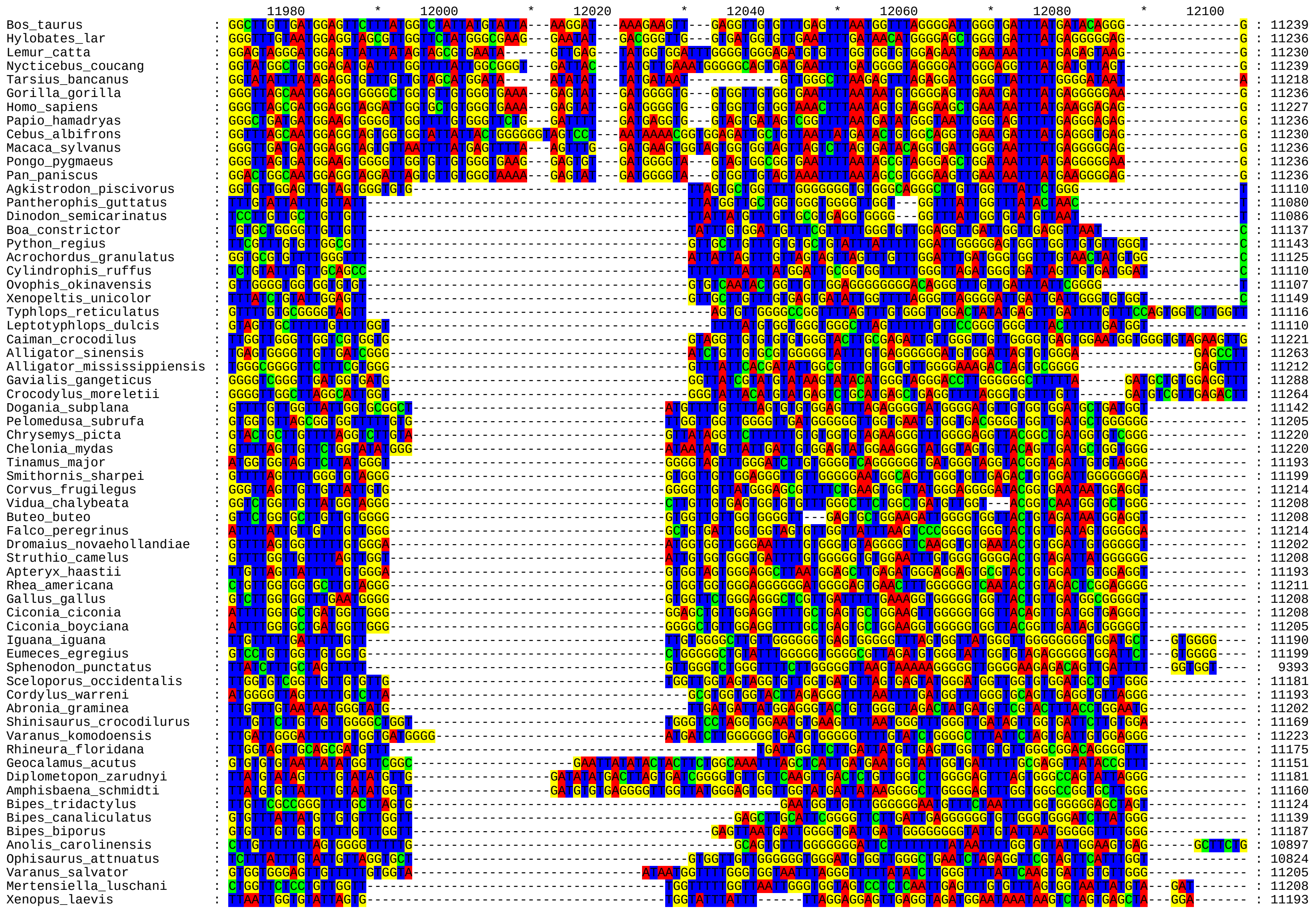
11980 * 12000 * 12020 * 12040 * 12060 * 12080 * 12100
Bos_taurus : 11239
Hylobates_lar : 11236
Lemur_catta : 11230
Nycticebus_coucang : 11239
Tarsius_bancanus : 11218
Gorilla_gorilla : 11236
Homo_sapiens : 11227
Papio_hamadryas : 11236
Cebus_albifrons : 11230
Macaca_sylvanus : 11236
Pongo_pygmaeus : 11236
Pan_paniscus : 11236
Agkistrodon_piscivorus : 11110
Pantherophis_guttatus : 11080
Dinodon_semicarinatus : 11086
Boa_constrictor : 11137
Python_regius : 11143
Acrochordus_granulatus : 11125
Cylindrophis_ruffus : 11110
Ovophis_okinavensis : 11107
Xenopeltis_unicolor : 11149
Typhlops_reticulatus : 11116
Leptotyphlops_dulcis : 11110
Caiman_crocodilus : 11221
Alligator_sinensis : 11263
Alligator_mississippiensis : 11212
Gavialis_gangeticus : 11288
Crocodylus_moreletii : 11264
Dogania_subplana : 11142
Pelomedusa_subrufa : 11205
Chrysemys_picta : 11220
Chelonia_mydas : 11220
Tinamus_major : 11193
Smithornis_sharpei : 11199
Corvus_frugilegus : 11214
Vidua_chalybeata : 11208
Buteo_buteo : 11208
Falco_peregrinus : 11214
Dromaius_novaehollandiae : 11202
Struthio_camelus : 11208
Apteryx_haastii : 11193
Rhea_americana : 11211
Gallus_gallus : 11208
Ciconia_ciconia : 11208
Ciconia_boyciana : 11205
Iguana_iguana : 11190
Eumeces_egregius : 11199
Sphenodon_punctatus : 9393
Sceloporus_occidentalis : 11181
Cordylus_warreni : 11193
Abronia_graminea : 11202
Shinisaurus_crocodilurus : 11169
Varanus_komodoensis : 11223
Rhineura_floridana : 11175
Geocalamus_acutus : 11151
Diplometopon_zarudnyi : 11181
Amphisbaena_schmidti : 11160
Bipes_tridactylus : 11124
Bipes_canaliculatus : 11139
Bipes_biporus : 11187
Anolis_carolinensis : 10897
Ophisaurus_attnuatus : 10824
Varanus_salvator : 11205
Mertensiella_luschani : 11208
Xenopus_laevis : 11193

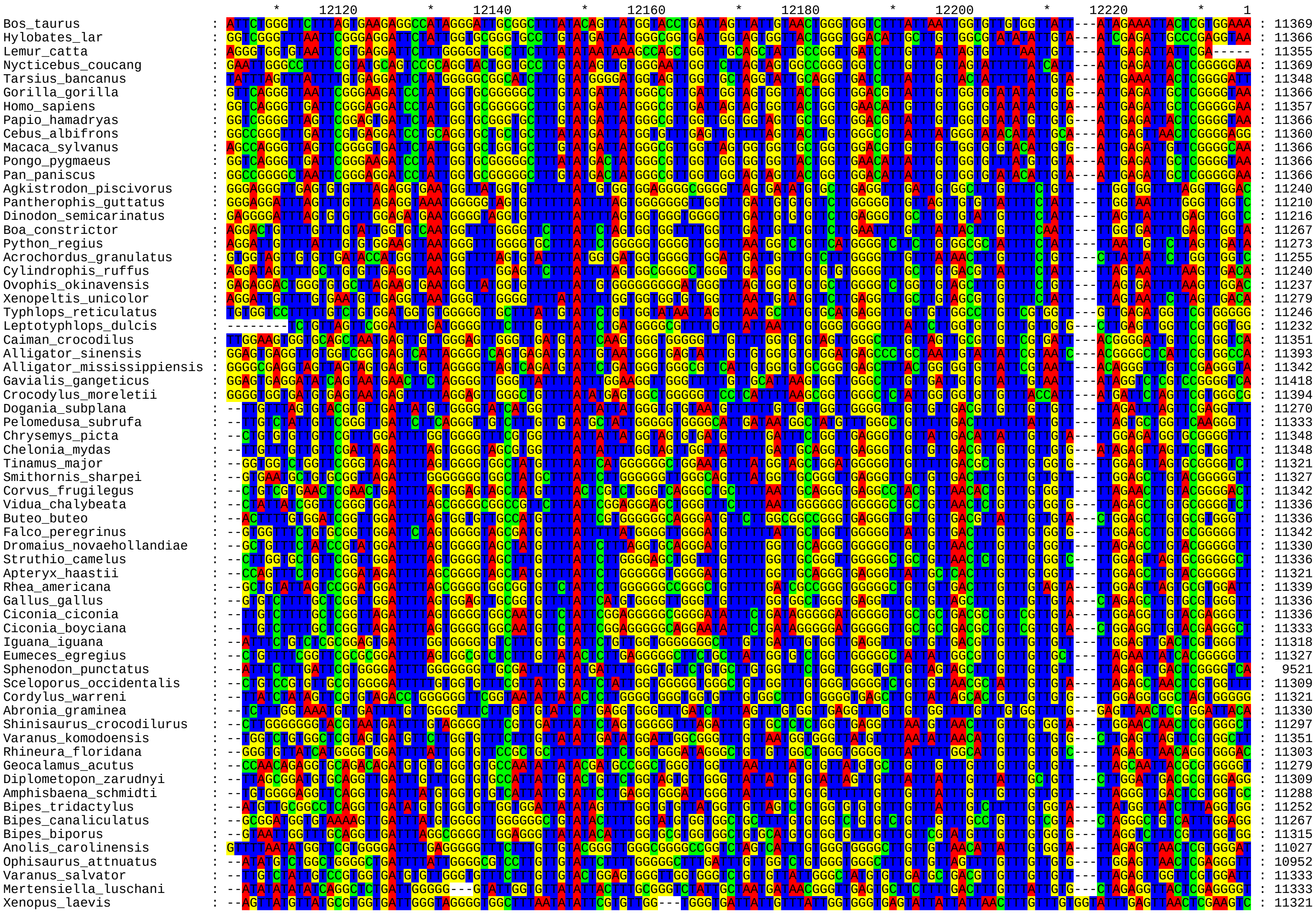
12120
12140
12160
12180
12200
12220
Bos_taurus : 11369
Hylobates_lar : 11366
Lemur_catta : 11355
Nycticebus_coucang : 11369
Tarsius_bancanus : 11348
Gorilla_gorilla : 11366
Homo_sapiens : 11357
Papio_hamadryas : 11366
Cebus_albifrons : 11360
Macaca_sylvanus : 11366
Pongo_pygmaeus : 11366
Pan_paniscus : 11366
Agkistrodon_piscivorus : 11240
Pantherophis_guttatus : 11210
Dinodon_semicarinatus : 11216
Boa_constrictor : 11267
Python_regius : 11273
Acrochordus_granulatus : 11255
Cylindrophis_ruffus : 11240
Ovophis_okinavensis : 11237
Xenopeltis_unicolor : 11279
Typhlops_reticulatus : 11246
Leptotyphlops_dulcis : 11232
Caiman_crocodilus : 11351
Alligator_sinensis : 11393
Alligator_mississippiensis : 11342
Gavialis_gangeticus : 11418
Crocodylus_moreletii : 11394
Dogania_subplana : 11270
Pelomedusa_subrufa : 11333
Chrysemys_picta : 11348
Chelonia_mydas : 11348
Tinamus_major : 11321
Smithornis_sharpei : 11327
Corvus_frugilegus : 11342
Vidua_chalybeata : 11336
Buteo_buteo : 11336
Falco_peregrinus : 11342
Dromaius_novaehollandiae : 11330
Struthio_camelus : 11336
Apteryx_haastii : 11321
Rhea_americana : 11339
Gallus_gallus : 11336
Ciconia_ciconia : 11336
Ciconia_boyciana : 11333
Iguana_iguana : 11318
Eumeces_egregius : 11327
Sphenodon_punctatus : 9521
Sceloporus_occidentalis : 11309
Cordylus_warreni : 11321
Abronia_graminea : 11330
Shinisaurus_crocodilurus : 11297
Varanus_komodoensis : 11351
Rhineura_floridana : 11303
Geocalamus_acutus : 11279
Diplometopon_zarudnyi : 11309
Amphisbaena_schmidti : 11288
Bipes_tridactylus : 11252
Bipes_canaliculatus : 11267
Bipes_biporus : 11315
Anolis_carolinensis : 11027
Ophisaurus_attnuatus : 10952
Varanus_salvator : 11333
Mertensiella_luschani : 11333
Xenopus_laevis : 11321

```
                               2240         *     12260         *
Bos_taurus                 : T--------------------------------- : 11370
Hylobates_lar              : T--------------------------------- : 11367
Lemur_catta                : ---------------------------------- :     -
Nycticebus_coucang         : T--------------------------------- : 11370
Tarsius_bancanus           : T--------------------------------- : 11349
Gorilla_gorilla            : T--------------------------------- : 11367
Homo_sapiens               : T--------------------------------- : 11358
Papio_hamadryas            : T--------------------------------- : 11367
Cebus_albifrons            : GGGT------------------------------ : 11364
Macaca_sylvanus            : T--------------------------------- : 11367
Pongo_pygmaeus             : T--------------------------------- : 11367
Pan_paniscus               : T--------------------------------- : 11367
Agkistrodon_piscivorus     : ACGTCTGGGG---GGGTTCCGTCCTTTT------ : 11265
Pantherophis_guttatus      : TCGGCTTGGG---GGTTTTCGTCCGTTT------ : 11235
Dinodon_semicarinatus      : TCGATTGGGT---GGTTTGCGGCCTTTT------ : 11241
Boa_constrictor            : TCGTCGTGGA---GGCTTGCGATCTTTT------ : 11292
Python_regius              : TCGGTTAGGT---GGGTTGCGACCTTTT------ : 11298
Acrochordus_granulatus     : AAAGAGTGTA---AGTTTTCGGTCATTT------ : 11280
Cylindrophis_ruffus        : TCGTTTGGGG---GGGTTGCGGTCTTTT------ : 11265
Ovophis_okinavensis        : TCGCTTAGGT---GGGTTTCGCCCTTTT------ : 11262
Xenopeltis_unicolor        : TCGTTGAGGT---GGGGTGCGGCCATTT------ : 11304
Typhlops_reticulatus       : GACGCATGGT---GGGGGTTTACGGTCAGTT--- : 11274
Leptotyphlops_dulcis       : GTTTCGTGGG---GCTTTGCGAGCGGTT------ : 11257
Caiman_crocodilus          : TTGTTTAGGG---TCGCTTCGTTCCTTC------ : 11376
Alligator_sinensis         : TCGGCGAGGG---GCATTTCGTTCTTTT------ : 11418
Alligator_mississippiensis : TCGTCAGGGG---GCGTTTCGCTCTTTT------ : 11367
Gavialis_gangeticus        : TCGTCGAGGG---GCACTTCGGTCATTG------ : 11443
Crocodylus_moreletii       : GCATCGAGGG---GCTCTGCGTTCATTG------ : 11419
Dogania_subplana           : AGTTTGTGGG---GCGATTCGTTCTATT------ : 11295
Pelomedusa_subrufa         : GTTTCGGGTA---GCTTGTCCAACTATT------ : 11358
Chrysemys_picta            : ATCTTGTGGG---ACGTTACATTCTATT------ : 11373
Chelonia_mydas             : GTCTCGTGGA---GTTATTCGTCCAATT------ : 11373
Tinamus_major              : GTCATGTGGG---GCAGTTCGGGCAGTT------ : 11346
Smithornis_sharpei         : ATCTCGAGGT---ACGATTCGGGCAGTG------ : 11352
Corvus_frugilegus          : ATCTCGGGGG---GCAATTCGGGCGGTT------ : 11367
Vidua_chalybeata           : GTCTCGTGGG---GCGATTCGGGCTGTT------ : 11361
Buteo_buteo                : GTCTTGTGGG---GCGATTCGGGCGGTT------ : 11361
Falco_peregrinus           : GTCTCGTGGG---GCAATTCGGGCGGTT------ : 11367
Dromaius_novaehollandiae   : ATCTCGGGGG---GCAATTCGGGCTGTA------ : 11355
Struthio_camelus           : ATCTCGGGGG---GCAATTCGGGCAGTT------ : 11361
Apteryx_haastii            : ATCTCGGGGG---ACGATTCGGGCAGTG------ : 11346
Rhea_americana             : ATCTCGGGGG---GCGATTCGGGCTGTT------ : 11364
Gallus_gallus              : GTCTCGGGGG---GCAATTCGGGCGGTT------ : 11361
Ciconia_ciconia            : ATCTCGTGGG---ACGATTCGAGCAGTT------ : 11361
Ciconia_boyciana           : ATCTCGTGGG---ACGATTCGAGCAGTT------ : 11358
Iguana_iguana              : GTCTCGTGGG---AGTTTGCGGGTGGTT------ : 11343
Eumeces_egregius           : GGTTCGAGGG---GGCCTTCGAGTTGTT------ : 11352
Sphenodon_punctatus        : GTCTCGTGGT---TCCTTGCGGGCGGTC------ :  9546
Sceloporus_occidentalis    : AGCTCGTGGT---GGATTGCGTGTAGTC------ : 11334
Cordylus_warreni           : TCGTCGTGGG---GGTCTGCGTGAGATT------ : 11346
Abronia_graminea           : TCGAAGCGGA---TTACGCTCTGTT--------- : 11352
Shinisaurus_crocodilurus   : TTCTCGTGGT---GGTCTTCGTTCTGTT------ : 11322
Varanus_komodoensis        : GGCTAGTGGA---TGCGTTCGTTCTGTATAC--- : 11379
Rhineura_floridana         : CTGATGTTAT---GGAGGTGTGCGGGAGGGG--- : 11331
Geocalamus_acutus          : AGCATGTGGT---GGGTTACGTGTGTTT------ : 11304
Diplometopon_zarudnyi      : ACGGCGGGGT---GGTATGCGGCCATTT------ : 11334
Amphisbaena_schmidti       : TGGGCGTGGG---GTTTCACGTCCGTTT------ : 11313
Bipes_tridactylus          : GGAACGGGGC---GCTATTCGGGTATTT------ : 11277
Bipes_canaliculatus        : AAGCCGAGGG---GGTGTTCGGCCGTTT------ : 11292
Bipes_biporus              : AGGACGTGGA---GCTGTTCGGCCATTC------ : 11340
Anolis_carolinensis        : GTCTCGTGGT---GGTTTACGGGTTATT------ : 11052
Ophisaurus_attnuatus       : ATTTCGTGGT---GGGCTCCGTTTGGTT------ : 10977
Varanus_salvator           : GGCTAGTGGG---TGTGTTCGTTCTGTTTAT--- : 11361
Mertensiella_luschani      : GTCTTTTGGT---GCATTACGGGCAGTA------ : 11358
Xenopus_laevis             : AATATGGTGG---GAGTCTTCGTGCGTT------ : 11346
```
