# Supplementary material for: Adaptive Evolution and Functional Redesign of Core Metabolic Proteins in Snakes
Source: PLoS One. 2008 May 21;3(5):e2201. doi: 10.1371/journal.pone.0002201 (PMC2376058; doi:10.1371/journal.pone.0002201)
Supplement: Table S1 — Laboratory and specimen voucher information for mitochondrial genomes sequenced in this study. (0.09 MB PDF) [file pone.0002201.s019.pdf]

Supplementary Table S1.1. Species for which mitochondrial genomes were sequenced in this study, with specimen voucher information.

Species	Specimen ID
Typhlops reticulatus	LSUMZ H-20102
Anolis carolinensis	CCA 8051
Ophisaurus attenuatus	LSUMZ H-15928
Varanus salvator	CCA 8037

LSUMZ = Louisiana State University Museum of Natural Science

CCA = Louisiana State University Museum of Natural Science - *Not Yet Cataloged*

Supplementary Table S1.2. Degenerate primers used for amplification of short fragments.

	Fragment	Forward Primer	Reverse Primer
**Snakes**	500 bp of 16sRNA	AACCCYYGTACCTYTTGCATCATG	CCGGTCTGAACTCAGATCACGT
	500 bp of COIII	GAAGCMGCWGCCTGATACTGACA	GGGTCRAAKCCRCATTCRTA
**Lizards**	500 bp of 12sRNA	AAACAAACTAGGATTAGATACCCTACTATGC	GAGGGTGACGGGCGGTGTGTGCG
	500 bp of COIII	CCAYATAGTMGACCCRAGCCC	GGKGCTTCGTARTATTCTATDGCTTG

Supplementary Table S1.3. Species-specific primers used for long PCRs to amplify the mitochondrial genome in two overlapping fragments.

	Species	Length	Forward Primer	Reverse Primer
**Snakes**	***Python regius***	**9kb**	CCTCGATGTTGGATCAGGACACCC	CCTGGGGGGACCAAGTGC
		**8kb**	TTCCAAGCACTTGGTCCCCC	GGGTGTCCTGATCCAACATCGAGG
	Typhlops reticulatus	**9kb**	CCTCGATGTTGGATCAGGACACCC	GTGGAGCTTTCTGCTTGGAAGGC
		**8kb**	CCAAGCAGAAAGCTCCACCAAAGG	GGGTGTCCTGATCCAACATCGAGG
**Lizards**	***Anolis carolinensis***	**9kb**	GCCTAGCCATTAACTGACACCC	GGGCTCATGTTACGGTAACGC
		**8kb**	TGTACAAAAGGGCCTGCGATATGGG	GGTGTCAGTTAATGGCTAGGCATAGTAGGG
	Ophisaurus attenuatus	**9kb**	CGCCCAACACAGCCTATATACCGCCG	CGGAGACCTGTTTGGACGGGTGGGG
		**8kb**	ACCCGTCCAAACAGGTCTCCG	GCGGTATATAGGCTGTGTTGGGCG
	Varanus salvator	**9kb**	CCCGACCACTACTAGCACCCC	GGAGTGGGACTTCGAATGGGTTAATGG
		**8kb**	TTCTTCTTCCTGGGATTCTTCTGAGCC	GGGGTGCTAGTAGTGGTCGGG
