# Supplementary material for: Adaptive Evolution and Functional Redesign of Core Metabolic Proteins in Snakes
Source: PLoS One. 2008 May 21;3(5):e2201. doi: 10.1371/journal.pone.0002201 (PMC2376058; doi:10.1371/journal.pone.0002201)
Supplement: Table S2 — Complete mitochondrial genomes used in this study, and associated Genbank accession numbers. (0.08 MB PDF) [file pone.0002201.s020.pdf]

Supplementary Table S2. Complete mitochondrial genomes used in this study, and associated Genbank accession numbers. Double asterisks to the left of accession numbers indicate new mitochondrial genomes generated in this study.

Vertebrate Group	Genbank Accession	Taxon	Vertebrate Group	Genbank Accession	Taxon
Amphibians	NC_002756	*Mertensiella luschani*	Crocodilians	NC_004448	*Alligator sinensis*
	NC_001573	*Xenopus laevis*		NC_001922	*A. mississippiensis*
Turtles	NC_000886	*Chelonia mydas*		NC_002744	*Caiman crocodilus*
	NC_002073	*Chrysemys picta*		D. Ray	*Crocodylus moreletii*
	NC_002780	*Dogania subplana*		NC_008241	*Gavialis gangeticus*
	NC_001947	*Pelomedusa subrufa*	Birds	NC_002782	*Apteryx haastii*
Tuatara	NC_004815	*Sphenodon punctatus*		NC_003128	*Buteo buteo*
Lizards	NC_005958	*Abronia graminea*		NC_002196	*Ciconia boyciana*
	NC_006284	*Amphisbaena schmidti*		NC_002197	*C. ciconia*
	** GB_######	*Anolis carolinensis*		NC_002069	*Corvus frugilegus*
	NC_006287	*Bipes biporus*		NC_002784	*Dromaius novaehollandiae*
	NC_006288	*B. caniculatus*		NC_000878	*Falco peregrinus*
	NC_006286	*B. tridactylus*		NC_001323	*Gallus gallus*
	NC_005962	*Cordylus warreni*		NC_000846	*Rhea americana*
	NC_006283	*Diplometopon zarudnyi*		NC_000879	*Smithornis sharpei*
	NC_006285	*Geocalamus acutus*		NC_002785	*Struthio camelus*
	NC_000888	*Eumeces egregious*		NC_002781	*Tinamus major*
	NC_002793	*Iguana iguana*		NC_000880	*Vidua chalybeata*
	** GB_######	*Ophisaurus attenuatus*	Mammals	NC_001567	*Bos taurus*
	NC_006282	*Rhineura floridana*		NC_002763	*Cebus albifrons*
	NC_005960	*Sceloporus occidentalis*		NC_002082	*Hylobates lar*
	NC_005959	*Shinisaurus crocodilurus*		NC_001646	*Pongo pygmaeus*
	AB080275-6	*Varanus komodoensis*		NC_001644	*Pan paniscus*
	** GB_######	*V. salvator*		NC_001645	*Gorilla gorilla*
Snakes	NC_007400	*Acrochordus granulatus*		NC_001807	*Homo sapiens*
	DQ523162	*Agkistrodon piscivorus*		NC_001992	*Papio hamadryas*
	NC_007398	*Boa constrictor*		NC_002764	*Macaca sylvanus*
	NC_007401	*Cylindrophis ruffus*		NC_002811	*Tarsius bancanus*
	NC_001945	*Dinodon semicarinatus*		NC_004025	*Lemur catta*
	NC_005961	*Leptotyphlops dulcis*		NC_002765	*Nycticebus coucang*
	NC_007397	*Ovophis okinavensis*			
	DQ523161	*Pantherophis slowinskii*			
	NC_007399	*Python regius*			
	** GB_######	*Typhlops reticulatus*			
	NC_007402	*Xenopeltis unicolor*			
