# Supplementary material for: Adaptive Evolution and Functional Redesign of Core Metabolic Proteins in Snakes
Source: PLoS One. 2008 May 21;3(5):e2201. doi: 10.1371/journal.pone.0002201 (PMC2376058; doi:10.1371/journal.pone.0002201)
Supplement: Table S3 — Sites that have undergone positive selection in COI along the branches leading to all snakes and that leading to the Alethinophidia, based on the branch-site model of PAML. (0.06 MB PDF) [file pone.0002201.s021.pdf]

**Supplementary Table S3**. Sites that have undergone positive selection in COI along the branches leading to all snakes and that leading to the Alethinophidia, based on the branch-site model of PAML. The probability of a site being under positive selection (*dN/dS* > 1) is based on the posterior probabilities from Bayes empirical Bayes support estimated in PAML. Only sites with posterior probabilities > 90% are shown. The reference to sites refers to amino acid sites of the cow COI protein.

| | **Branch** | |
|---|---|---|
| **Site** | **Snakes** | **Alethinophidia** |
| **32** | | 0.96 |
| **35** | | 0.90 |
| **42** | | 0.96 |
| **42** | 0.93 | |
| **50** | | 0.93 |
| **54** | | 0.90 |
| **57** | | 0.93 |
| **73** | 0.92 | |
| **114** | | 0.92 |
| **141** | | 0.90 |
| **178** | | 0.99 |
| **197** | 0.92 | |
| **223** | | 0.95 |
| **256** | | 1.00 |
| **266** | | 1.00 |
| **299** | | 0.92 |
| **301** | | 0.92 |
| **307** | | 0.91 |
| **322** | | 0.91 |
| **328** | 0.98 | 1.00 |
| **335** | | 0.98 |
| **339** | | 1.00 |
| **434** | | 0.91 |
| **443** | | 0.91 |
| **459** | | 0.93 |
| **463** | 0.99 | |
| **472** | | 0.98 |
| **478** | | 0.95 |
| **481** | 0.98 | |
| **486** | | 0.98 |
| **487** | | 1.00 |
| **491** | | 0.92 |
| **505** | 0.93 | |
