# Supplementary material for: Adaptive Evolution and Functional Redesign of Core Metabolic Proteins in Snakes
Source: PLoS One. 2008 May 21;3(5):e2201. doi: 10.1371/journal.pone.0002201 (PMC2376058; doi:10.1371/journal.pone.0002201)
Supplement: Table S4 — Results of likelihood-based analysis of coevolution among protein residues within the COI protein. (0.09 MB PDF) [file pone.0002201.s022.pdf]

Supplementary Table S4. Results of likelihood-based analysis of coevolution among protein residues within the COI protein. In part A, the total number of pairs are shown for analyzing coevolution with respect to volume, polarity, and hydrophobicity within the set of 23 unique snake sites, as well as with the results for polarity among all sites. The fraction of sites that demonstrated significant evidence of coevolutionary replacements at the $p<0.05$ level are given for all analyses, along with the fraction of the fraction of these "significant" pairs that are expected to be truly coevolving based on the number of comparisons made (i.e., 1 – the false discovery rate). In part B, highly significant ($p<0.01$) unique site pairs are shown (based on simulations of the null log likelihood ratio distribution, which were quite close to expectations from the Chi Square distribution). Site numbers in (B) are based on the cow CO reference.

A.

	**Volume**	**Polarity**	**Hydrophobicity**	**Polar-All**
**Site Pairs**	126	232	147	3407
**Coevolving Pairs (5% cutoff)**	88.9%	65.5%	90.5%	59.8%
**Coevolving Pair Fraction**	94.38%	92.37%	94.47%	91.65%

B.

**Polarity**			**Hydrophobicity**		
**Site 1**	**Site 2**	**2ΔlnL**	**Site 1**	**Site 2**	**2ΔlnL**
**286**	**447**	17.103252	**286**	**447**	20.033842
**258**	**286**	15.344366	**231**	**443**	15.524782
**258**	**447**	15.343738	**258**	**286**	15.348934
**231**	**443**	13.402264	**258**	**447**	15.348934
**267**	**447**	12.67101	**267**	**286**	14.816056
**267**	**286**	12.670488	**267**	**447**	14.816056
**258**	**267**	11.461646	**26**	**231**	12.456162
**258**	**299**	11.423728	**26**	**443**	12.456162
**231**	**281**	10.837166	**258**	**299**	11.958086
**281**	**443**	10.837164	**258**	**267**	11.434084
**272**	**281**	10.206224	**231**	**281**	11.067008
**272**	**443**	10.197028	**281**	**443**	10.814424
**231**	**272**	10.197026	**256**	**353**	10.753326
**205**	**231**	9.548508	**301**	**353**	10.753326
**205**	**443**	9.548508	**256**	**301**	10.75308
**35**	**299**	9.483356	**194**	**353**	10.741258
**194**	**256**	9.45481	**194**	**256**	10.741256
**256**	**301**	9.45481	**194**	**301**	10.741256
**256**	**353**	9.45481	**174**	**281**	10.542516
**194**	**301**	9.454804	**272**	**443**	10.19837
**194**	**353**	9.454804	**231**	**272**	10.194886
**301**	**353**	9.454804	**272**	**281**	9.984506
**174**	**231**	9.418532	**174**	**231**	9.81607
**174**	**443**	9.418532	**174**	**443**	9.81607
**266**	**301**	9.333612	**205**	**231**	9.550284
**266**	**353**	9.333612	**205**	**443**	9.550284
**194**	**266**	9.333606	**205**	**281**	8.971526
**256**	**266**	9.33351	**299**	**353**	8.880646

**174**	**281**	9.222674	**299**	**301**	8.880642
**37**	**256**	9.156168	**194**	**299**	8.880224
**37**	**194**	9.156166	**256**	**299**	8.87743
**37**	**301**	9.156166	**174**	**272**	8.274962
**37**	**353**	9.156166	**194**	**267**	8.017124
**205**	**281**	8.959538	**256**	**267**	8.015986
**194**	**299**	8.823206	**267**	**353**	8.01271
**299**	**301**	8.823198	**267**	**301**	8.012708
**299**	**353**	8.823198	**26**	**281**	7.665456
**256**	**299**	8.823088	**231**	**353**	7.278846
**26**	**256**	8.554376	**194**	**231**	7.278812
**26**	**194**	8.554372	**194**	**443**	7.278812
**26**	**301**	8.554372	**353**	**443**	7.278812
**26**	**353**	8.554372	**231**	**256**	7.278
**35**	**205**	8.212686	**231**	**301**	7.278
**267**	**301**	8.011872	**256**	**443**	7.27794
**267**	**353**	8.011872	**301**	**443**	7.27794
**194**	**267**	8.01187	**174**	**205**	7.132574
**256**	**267**	8.01187	**26**	**272**	7.076318
**37**	**266**	7.438014	**108**	**286**	7.017936
**54**	**194**	7.28251	**108**	**447**	7.017936
**54**	**301**	7.28251	**26**	**174**	6.750424
**54**	**353**	7.28251	**205**	**272**	6.732824
**54**	**256**	7.282434			
**54**	**108**	7.195216			
**266**	**299**	7.106348			
**174**	**272**	7.02517			
**37**	**299**	6.939808			
**108**	**286**	6.73709			
**108**	**447**	6.73709			
**194**	**231**	6.71859			
**194**	**443**	6.71859			
**301**	**443**	6.71859			
**353**	**443**	6.71859			
**231**	**301**	6.718578			
**231**	**353**	6.718578			
**256**	**443**	6.71853			
**231**	**256**	6.718508			
