# Supplementary material for: Adaptive Evolution and Functional Redesign of Core Metabolic Proteins in Snakes
Source: PLoS One. 2008 May 21;3(5):e2201. doi: 10.1371/journal.pone.0002201 (PMC2376058; doi:10.1371/journal.pone.0002201)
Supplement: Table S5 — Conservation of residues in proton transfer channel D across the 65 taxon dataset used. (0.08 MB PDF) [file pone.0002201.s023.pdf]

Supplementary Table S5. Conservation of residues in proton transfer channel D across the 65 taxon dataset used. The symbol "-" refers to amino acids that are the same as the sequence of *Bos taurus* at the corresponding site.

		**Channel D**													
		**11**	**12**	**19**	**91**	**98**	**101**	**108**	**115**	**142**	**146**	**149**	**156**	**157**	**503**
**Primates**	*Bos taurus*	N	H	Y	D	N	S	S	S	S	T	S	S	S	H
	Hylobates lar	-	-	-	-	-	-	-	-	-	-	-	-	-	-
	Lemur catta	-	-	-	-	-	-	-	-	-	-	-	-	-	-
	Nycticebus coucang	-	-	-	-	-	-	-	-	-	-	-	-	-	-
	Tarsius bancanus	-	-	-	-	-	-	-	-	-	-	-	-	-	-
	Gorilla gorilla	-	-	-	-	-	-	-	-	-	-	-	-	-	-
	Homo sapiens	-	-	-	-	-	-	-	-	-	-	-	-	-	-
	Papio hamadryas	-	-	-	-	-	-	-	-	-	V	-	-	-	-
	Cebus albifrons	-	-	-	-	-	-	-	-	-	-	-	-	-	-
	Macaca sylvanus	-	-	-	-	-	-	-	-	-	I	-	-	-	-
	Pongo pygmaeus	-	-	-	-	-	-	-	-	-	-	-	-	-	-
	Pan paniscus	-	-	-	-	-	-	-	-	-	-	-	-	-	-
**Snakes**	*Agkistrodon piscivorus*	-	-	-	-	-	-	A	-	-	A	-	-	-	-
	Pantherophis slowinskii	-	-	-	-	-	-	A	-	-	A	-	-	-	-
	Dinodon semicarinatus	-	-	-	-	-	-	A	-	-	A	-	-	-	-
	Boa constrictor	-	-	-	-	-	-	A	-	-	A	-	-	-	-
	Python regius	-	-	-	-	-	-	A	-	-	A	-	-	-	-
	Acrochordus granulatus	-	-	-	-	-	-	A	-	-	A	-	-	-	-
	Cylindrophis ruffus	-	-	-	-	-	-	A	-	-	V	-	-	-	-
	Ovophis okinavensis	-	-	-	-	-	-	A	-	-	A	-	-	-	-
	Xenopeltis unicolor	-	-	-	-	-	-	A	-	-	A	-	-	-	-
	Typhlops reticulatus	-	-	-	-	-	-	-	-	-	A	-	-	-	T
	Leptotyphlops dulcis	-	-	-	-	-	-	A	-	-	A	-	-	-	-
**Lizards**	*Iguana iguana*	-	-	-	-	-	-	-	-	-	-	-	-	-	-
	Eumeces egregius	-	-	-	-	-	-	-	-	-	-	-	-	-	-
	Sceloporus occidentalis	-	-	-	-	-	-	-	-	-	-	-	-	-	-
	Cordylus warreni	-	-	-	-	-	-	-	-	-	-	-	-	-	-
	Abronia graminea	-	-	-	-	-	-	-	-	-	-	-	-	-	-
	Shinisaurus crocodilurus	-	-	-	-	-	-	-	-	-	-	-	-	-	-
	Varanus komodoensis	-	-	-	-	-	-	-	-	-	-	-	-	-	-
	Rhineura floridana	-	-	-	-	-	-	A	G	-	-	-	-	-	-
	Geocalamus acutus	-	-	-	-	-	-	-	-	-	-	-	-	-	-
	Diplometopon zarudnyi	-	-	-	-	-	-	-	-	-	-	-	-	-	-
	Amphisbaena schmidti	-	-	-	-	-	-	-	-	-	-	-	-	-	-
	Bipes tridactylus	-	-	-	-	-	-	-	-	-	-	-	-	-	-
	Bipes canaliculatus	-	-	-	-	-	-	-	-	-	-	-	-	-	-
	Bipes biporus	-	-	-	-	-	-	-	-	-	-	-	-	-	-
	Anolis carolinensis	-	-	-	-	-	-	-	-	-	-	-	-	-	-
	Ophisaurus attenuatus	-	-	-	-	-	-	-	-	-	-	-	-	-	-
	Varanus salvator	-	-	-	-	-	-	-	-	-	-	-	-	-	-
**Tuatara**	*Sphenodon punctatus*	-	-	-	-	-	-	-	-	-	-	-	-	-	-
**Crocodilians**	*Caiman crocodilus*	-	-	-	-	-	-	-	-	-	-	-	-	-	-
	Alligator sinensis	-	-	-	-	-	-	-	-	-	-	-	-	-	-
	Alligator mississippiensis	-	-	-	-	-	-	-	-	-	-	-	-	-	-
	Gavialis gangeticus	-	-	-	-	-	-	-	-	-	-	-	-	-	-
	Crocodylus moreletii	-	-	-	-	-	-	-	-	-	-	-	-	-	-
**Birds**	*Tinamus major*	-	-	-	-	-	-	-	-	-	A	-	-	-	-
	Smithornis sharpei	-	-	-	-	-	-	-	-	-	A	-	-	-	-
	Corvus frugilegus	-	-	-	-	-	-	-	-	-	A	-	-	-	-
	Vidua chalybeata	-	-	-	-	-	-	-	-	-	A	-	-	-	-
	Buteo buteo	-	-	-	-	-	-	-	-	-	A	-	-	-	-
	Falco peregrinus	-	-	-	-	-	-	-	-	-	A	-	-	-	-
	Dromaius novaehollandiae	-	-	-	-	-	-	-	-	-	A	-	-	-	-
	Struthio camelus	-	-	-	-	-	-	-	-	-	A	-	-	-	-
	Apteryx haastii	-	-	-	-	-	-	-	-	-	A	-	-	-	-
	Rhea american	-	-	-	-	-	-	-	-	-	A	-	-	-	-
	Gallus gallus	-	-	-	-	-	-	-	-	-	A	-	-	-	-
	Ciconia ciconia	-	-	-	-	-	-	-	-	-	A	-	-	-	-
	Ciconia boyciana	-	-	-	-	-	-	-	-	-	A	-	-	-	-
**Turtles**	*Dogania subplana*	-	-	-	-	-	-	-	-	-	-	-	-	-	-
	Pelomedusa subrufa	-	-	-	-	-	-	-	-	-	A	-	-	-	-
	Chrysemys picta	-	-	-	-	-	-	-	-	-	-	-	-	-	-
	Chelonia mydas	-	-	-	-	-	-	-	-	-	-	-	-	-	-
**Amphibians**	*Mertensiella luschani*	-	-	-	-	-	-	-	-	-	-	-	-	-	-
	Xenopus laevis	-	-	-	-	-	-	-	-	-	-	-	-	-	-
