# Supplementary material for: Adaptive Evolution and Functional Redesign of Core Metabolic Proteins in Snakes
Source: PLoS One. 2008 May 21;3(5):e2201. doi: 10.1371/journal.pone.0002201 (PMC2376058; doi:10.1371/journal.pone.0002201)
Supplement: Table S6 — Conservation of residues in proton transfer channel H across the 65 taxon dataset used. (0.08 MB PDF) [file pone.0002201.s024.pdf]

Supplementary Table S6. Conservation of residues in proton transfer channel H across the 65 taxon dataset used. The symbol "-" refers to amino acids that are the same as the sequence of *Bos taurus* at the corresponding site.

		Channel H									
		**38**	**382**	**407**	**413**	**424**	**428**	**443**	**451**	**454**	**461**
**Primates**	*Bos taurus*	R	S	D	H	T	Q	Y	N	S	S
	Hylobates lar	-	-	Q	-	-	-	-	-	-	-
	Lemur catta	-	-	N	-	-	-	-	-	-	-
	Nycticebus coucang	-	-	Q	-	-	-	-	-	-	-
	Tarsius bancanus	-	-	P	-	-	-	-	-	-	-
	Gorilla gorilla	-	-	Q	-	-	-	-	-	-	-
	Homo sapiens	-	-	Q	-	-	-	-	-	-	-
	Papio hamadryas	-	-	Q	-	-	-	-	-	-	-
	Cebus albifrons	-	-	Q	-	-	-	-	-	-	-
	Macaca sylvanus	-	-	Q	-	-	-	-	-	-	-
	Pongo pygmaeus	-	-	Q	-	-	-	-	-	-	-
	Pan paniscus	-	-	Q	Q	-	-	-	-	-	-
**Snakes**	*Agkistrodon piscivorus*	-	-	Q	Q	-	-	F	-	-	-
	Pantherophis slowinskii	-	-	Q	Q	-	-	F	-	-	-
	Dinodon semicarinatus	-	-	Q	Q	-	-	F	-	-	-
	Boa constrictor	-	-	Q	Q	-	-	F	-	-	-
	Python regius	-	-	Q	Q	-	-	F	-	-	-
	Acrochordus granulatus	-	-	Q	Q	-	-	F	-	-	-
	Cylindrophis ruffus	-	-	Q	Q	-	-	F	-	-	-
	Ovophis okinavensis	-	-	Q	Q	-	-	F	-	-	-
	Xenopeltis unicolor	-	-	Q	Q	-	-	F	-	-	-
	Typhlops reticulatus	-	-	Q	Q	-	-	-	-	-	-
	Leptotyphlops dulcis	-	-	P	Q	-	-	-	-	-	-
**Lizards**	*Iguana iguana*	-	-	H	Q	-	-	-	-	-	-
	Eumeces egregius	-	-	Q	-	-	-	-	-	-	-
	Sceloporus occidentalis	-	-	N	Q	-	-	-	-	-	-
	Cordylus warreni	-	-	Q	-	-	-	-	-	-	-
	Abronia graminea	-	-	S	-	-	-	-	-	-	-
	Shinisaurus crocodilurus	-	-	P	-	-	-	-	-	-	-
	Varanus komodoensis	-	-	P	Q	-	-	-	-	-	-
	Rhineura floridana	-	-	A	Q	-	-	-	-	-	-
	Geocalamus acutus	-	-	Q	Q	-	-	-	-	-	-
	Diplometopon zarudnyi	-	-	P	Q	-	-	-	-	-	-
	Amphisbaena schmidti	-	-	Q	Q	-	-	-	-	-	-
	Bipes tridactylus	-	-	Q	Q	-	-	-	-	-	-
	Bipes canaliculatus	-	-	Q	Q	-	-	-	-	-	-
	Bipes biporus	-	-	Q	Q	-	-	-	-	-	-
	Anolis carolinensis	-	-	Q	Q	-	-	-	-	-	-
	Ophisaurus attenuatus	-	-	T	-	-	-	-	-	-	-
	Varanus salvator	-	-	P	Q	-	-	-	-	-	-
**Tuatara**	*Sphenodon punctatus*	-	-	K	-	-	-	-	-	-	-
**Crocodilians**	*Caiman crocodilus*	-	-	P	Q	-	-	-	-	-	-
	Alligator sinensis	-	-	Q	Q	-	-	-	-	-	-
	Alligator mississippiensis	-	-	P	Q	-	-	-	-	-	-
	Gavialis gangeticus	-	-	P	Q	-	-	-	-	-	-
	Crocodylus moreletii	-	-	S	Q	-	-	-	-	-	-
**Turtles**	*Dogania subplana*	-	-	Q	-	-	-	-	-	-	-
	Pelomedusa subrufa	-	-	S	-	-	-	-	-	-	-
	Chrysemys picta	-	-	Q	-	-	-	-	-	-	-
	Chelonia mydas	-	-	Q	-	-	-	-	-	-	-
**Birds**	*Tinamus major*	-	-	P	-	-	-	-	-	-	-
	Smithornis sharpei	-	-	P	-	-	-	-	-	-	-
	Corvus frugilegus	-	-	S	-	-	-	-	-	-	-
	Vidua chalybeata	-	-	S	-	-	-	-	-	-	-
	Buteo buteo	-	-	P	-	-	-	-	-	-	-
	Falco peregrinus	-	-	P	-	-	-	-	-	-	-
	Dromaius novaehollandiae	-	-	P	-	-	-	-	-	-	-
	Struthio camelus	-	-	P	-	-	-	-	-	-	-
	Apteryx haastii	-	-	P	-	-	-	-	-	-	-
	Rhea americana	-	-	P	-	-	-	-	-	-	-
	Gallus gallus	-	-	P	-	-	-	-	-	-	-
	Ciconia ciconia	-	-	P	-	-	-	-	-	-	-
	Ciconia boyciana	-	-	P	-	-	-	-	-	-	-
**Amphibians**	*Mertensiella luschani*	-	-	P	-	-	-	-	-	-	-
	Xenopus laevis	-	-	E	-	-	-	-	-	-	-
