# Supplementary material for: Adaptive Evolution and Functional Redesign of Core Metabolic Proteins in Snakes
Source: PLoS One. 2008 May 21;3(5):e2201. doi: 10.1371/journal.pone.0002201 (PMC2376058; doi:10.1371/journal.pone.0002201)
Supplement: Table S7 — Conservation of residues in proton transfer channel K across the 65 taxon dataset used. (0.08 MB PDF) [file pone.0002201.s025.pdf]

Supplementary Table S7. Conservation of residues in proton transfer channel K across the 65 taxon dataset used. The symbol "-" refers to amino acids that are the same as the sequence of *Bos taurus* at the corresponding site.

		Channel K											
		**240**	**244**	**255**	**256**	**265**	**291**	**316**	**319**	**368**	**489**	**490**	**491**
**Primates**	*Bos taurus*	H	Y	S	H	K	H	T	K	H	T	T	N
	Hylobates lar	-	-	-	-	-	-	-	-	-	S	-	-
	Lemur catta	-	-	-	-	-	-	-	-	-	P	-	-
	Nycticebus coucang	-	-	-	-	-	-	-	-	-	H	-	-
	Tarsius bancanus	-	-	-	-	-	-	-	-	-	-	-	-
	Gorilla gorilla	-	-	-	-	-	-	-	-	-	S	-	-
	Homo sapiens	-	-	-	-	-	-	-	-	-	S	M	-
	Papio hamadryas	-	-	-	-	-	-	-	-	-	S	-	S
	Cebus albifrons	-	-	-	-	-	-	-	-	-	S	-	-
	Macaca sylvanus	-	-	-	-	-	-	-	-	-	L	-	-
	Pongo pygmaeus	-	-	-	-	-	-	-	-	-	S	-	S
	Pan paniscus	-	-	-	-	-	-	-	-	-	S	A	-
**Snakes**	*Agkistrodon piscivorus*	-	-	-	S	-	-	-	-	-	K	-	H
	Pantherophis slowinskii	-	-	-	S	-	-	-	-	-	K	-	H
	Dinodon semicarinatus	-	-	-	S	-	-	-	-	-	K	-	H
	Boa constrictor	-	-	-	S	-	-	-	-	-	K	-	H
	Python regius	-	-	-	S	-	-	-	-	-	K	-	H
	Acrochordus granulatus	-	-	-	S	-	-	-	-	-	K	I	H
	Cylindrophis ruffus	-	-	-	S	-	-	-	-	-	K	-	H
	Ovophis okinavensis	-	-	-	S	-	-	-	-	-	K	-	H
	Xenopeltis unicolor	-	-	-	S	-	-	-	-	-	K	-	H
	Typhlops reticulatus	-	-	-	-	-	-	-	-	-	K	-	D
	Leptotyphlops dulcis	-	-	-	-	-	-	-	-	-	K	-	S
**Lizards**	*Iguana iguana*	-	-	-	-	-	-	-	-	-	-	-	-
	Eumeces egregius	-	-	-	-	-	-	-	-	-	S	-	-
	Sceloporus occidentalis	-	-	-	-	-	-	-	-	-	-	-	-
	Cordylus warreni	-	-	-	-	-	-	-	-	-	-	-	-
	Abronia graminea	-	-	-	-	-	-	-	-	-	H	-	-
	Shinisaurus crocodilurus	-	-	-	-	-	-	-	-	-	N	-	-
	Varanus komodoensis	-	-	-	-	-	-	-	-	-	E	A	-
	Rhineura floridana	-	-	-	-	-	-	-	-	-	H	K	G
	Geocalamus acutus	-	-	-	-	-	-	-	-	-	M	-	-
	Diplometopon zarudnyi	-	-	-	-	-	-	-	-	-	A	-	-
	Amphisbaena schmidti	-	-	-	-	-	-	-	-	-	S	-	-
	Bipes tridactylus	-	-	-	-	-	-	-	-	-	-	-	-
	Bipes canaliculatus	-	-	-	-	-	-	-	-	-	M	-	-
	Bipes biporus	-	-	-	-	-	-	-	-	-	-	-	-
	Anolis carolinensis	-	-	-	-	-	-	-	-	-	S	-	-
	Ophisaurus attenuatus	-	-	-	-	-	-	-	-	-	H	-	-
	Varanus salvator	-	-	-	-	-	-	-	-	-	E	M	-
**Tuatara**	*Sphenodon punctatus*	-	-	-	-	-	-	-	-	-	F	-	G
**Crocodilians**	*Caiman crocodilus*	-	-	-	-	-	-	-	-	-	I	-	-
	Alligator sinensis	-	-	-	-	-	-	-	-	-	-	-	-
	Alligator mississippiensis	-	-	-	-	-	-	-	-	-	M	-	-
	Gavialis gangeticus	-	-	-	-	-	-	-	-	-	-	-	-
	Crocodylus moreletii	-	-	-	-	-	-	-	-	-	S	-	-
**Turtles**	*Dogania subplana*	-	-	-	-	-	-	-	-	-	-	-	-
	Pelomedusa subrufa	-	-	-	-	-	-	-	-	-	S	-	-
	Chrysemys picta	-	-	-	-	-	-	-	-	-	-	-	-
	Chelonia mydas	-	-	-	-	-	-	-	-	-	-	-	-
**Birds**	*Tinamus major*	-	-	-	-	-	-	-	-	-	S	-	-
	Smithornis sharpei	-	-	-	-	-	-	-	-	-	N	-	-
	Corvus frugilegus	-	-	-	-	-	-	-	-	-	S	-	-
	Vidua chalybeata	-	-	-	-	-	-	-	-	-	S	-	-
	Buteo buteo	-	-	-	-	-	-	-	-	-	-	-	-
	Falco peregrinus	-	-	-	-	-	-	-	-	-	S	-	-
	Dromaius novaehollandiae	-	-	-	-	-	-	-	-	-	P	-	-
	Struthio camelus	-	-	-	-	-	-	-	-	-	A	-	-
	Apteryx haastii	-	-	-	-	-	-	-	-	-	-	-	-
	Rhea americana	-	-	-	-	-	-	-	-	-	-	-	-
	Gallus gallus	-	-	-	-	-	-	-	-	-	A	-	-
	Ciconia ciconia	-	-	-	-	-	-	-	-	-	P	-	-
	Ciconia boyciana	-	-	-	-	-	-	-	-	-	P	-	-
**Amphibians**	*Mertensiella luschani*	-	-	-	-	-	-	-	-	-	S	-	-
	Xenopus laevis	-	-	-	-	-	-	-	-	-	S	-	M
