# Supplementary material for: Adaptive Evolution and Functional Redesign of Core Metabolic Proteins in Snakes
Source: PLoS One. 2008 May 21;3(5):e2201. doi: 10.1371/journal.pone.0002201 (PMC2376058; doi:10.1371/journal.pone.0002201)
Supplement: Table S8 — Conservation of residues surrounding channel D across the 65 taxon dataset used. (0.13 MB PDF) [file pone.0002201.s026.pdf]

Supplementary Table S8. Conservation of residues surrounding channel D across the 65 taxon dataset used. The symbol "-" refers to amino acids that are the same as the reference sequence of *Bos taurus* at the corresponding site.

		10	13	14	15	16	17	18	80	81	84	85	90	92	97	99	100	102	107	109	111	114	116	141	143	145	147	148	150	155	158	502	504
**Primates**	*B. taurus*	T	K	D	I	G	T	L	N	W	P	L	P	M	M	N	M	F	P	F	L	A	S	A	V	L	I	F	L	V	I	Y	T
	H. lar	-	-	-	-	-	-	-	-	-	-	-	-	-	-	-	-	-	-	-	-	-	A	-	-	-	-	-	-	-	-	-	-
	L. catta	-	-	-	-	-	-	-	-	-	-	-	-	-	-	-	-	-	-	-	-	-	-	-	-	-	-	-	-	-	-	-	-
	N. coucang	-	-	-	-	-	-	-	-	-	-	-	-	-	-	-	-	-	-	-	-	-	-	-	-	-	-	-	-	-	-	-	-
	T. bancanus	-	-	-	-	-	-	-	-	-	-	-	-	-	-	-	-	-	-	-	-	-	-	-	-	-	-	-	-	-	-	-	-
	G. gorilla	-	-	-	-	-	-	-	-	-	-	-	-	-	-	-	-	-	-	-	-	-	A	-	-	-	-	-	-	I	-	-	-
	H. sapiens	-	-	-	-	-	-	-	-	-	-	-	-	-	-	-	-	-	-	L	-	-	A	-	-	-	-	-	-	-	-	-	-
	P. hamadryas	-	-	-	-	-	-	-	-	-	-	-	-	-	L	-	-	-	-	-	-	-	T	-	-	-	-	-	-	I	-	H	-
	C. albifrons	-	-	-	-	-	-	-	-	-	-	-	-	-	-	-	-	-	-	L	-	-	-	-	-	-	-	-	-	I	-	-	-
	M. sylvanus	-	-	-	-	-	-	-	-	-	-	-	-	-	L	-	-	-	-	-	-	-	T	-	-	-	-	-	-	I	-	-	-
	P. pygmaeus	-	-	-	-	-	-	-	-	-	-	-	-	-	-	-	-	-	-	-	-	-	A	-	-	-	-	-	-	I	-	-	-
	P. paniscus	-	-	-	-	-	-	-	-	-	-	-	-	-	-	-	-	-	-	L	-	-	A	-	-	-	-	-	-	-	-	-	-
**Snakes**	*A. piscivorus*	-	-	-	-	-	-	-	-	-	-	-	-	-	-	-	-	-	-	L	-	S	-	P	-	-	-	-	-	A	-	-	-
	P. slowinskii	-	-	-	-	-	-	-	-	-	-	-	-	-	-	-	-	-	-	L	-	S	-	P	-	-	-	-	-	A	-	-	-
	D. semicarinatus	-	-	-	-	-	-	-	-	-	-	-	-	-	-	-	-	-	-	L	-	S	-	P	-	-	-	-	-	A	-	-	-
	B. constrictor	-	-	-	-	-	-	-	-	-	-	-	-	-	-	-	-	-	-	L	-	S	-	P	-	-	-	-	-	A	-	H	-
	P. regius	-	-	-	-	-	-	-	-	-	-	-	-	-	-	-	-	-	-	L	-	S	-	P	-	-	-	-	-	A	-	H	-
	A. granulatus	-	-	-	-	-	-	-	-	-	-	-	-	-	-	-	-	-	-	L	-	S	-	P	-	-	-	-	-	A	-	H	-
	C. ruffus	-	-	-	-	-	-	-	-	-	-	-	-	-	-	-	-	-	-	L	-	S	-	P	-	-	-	-	-	A	-	-	-
	O. okinavensis	-	-	-	-	-	-	-	-	-	-	-	-	-	-	-	-	-	-	L	-	S	-	P	-	-	-	-	-	A	-	H	-
	X. unicolor	-	-	-	-	-	-	-	-	-	-	-	-	-	-	-	-	-	-	L	-	S	-	P	-	-	-	-	-	A	-	-	-
	T. reticulatus	-	-	-	-	-	-	-	-	-	-	-	-	-	-	-	-	-	-	L	-	-	-	-	-	-	-	-	-	M	-	T	T
	L. dulcis	-	-	-	-	-	-	-	-	-	-	-	-	-	-	-	-	-	-	L	-	T	-	T	-	-	-	-	-	-	-	H	-
**Lizards**	*I. iguana*	-	-	-	-	-	-	-	-	-	-	-	-	-	-	-	-	-	-	-	-	-	-	-	-	-	-	-	-	I	-	-	-
	E. egregius	-	-	-	-	-	-	-	-	-	-	-	-	-	-	-	-	-	-	-	-	-	-	-	-	-	-	-	-	-	-	-	-
	S. occidentalis	-	-	-	-	-	-	-	-	-	-	-	-	-	-	-	-	-	-	-	-	-	-	-	-	-	-	-	-	-	-	-	-
	C. warreni	-	-	-	-	-	-	-	-	-	-	-	-	-	-	-	-	-	-	L	-	-	-	-	-	-	-	-	-	-	-	F	-
	A. graminea	-	-	-	-	-	-	-	-	-	-	-	-	-	-	-	-	-	-	L	-	-	-	-	-	-	-	-	-	I	-	N	-
	S. crocodilurus	-	-	-	-	-	-	-	-	-	-	-	-	-	-	-	-	-	-	L	-	-	-	-	-	-	-	-	-	-	-	F	-
	V. komodoensis	-	-	-	-	-	-	-	-	-	-	-	-	-	-	-	-	-	-	L	-	-	A	-	-	-	-	-	-	-	-	F	-
	R. floridana	-	-	-	-	-	-	-	-	-	-	-	-	-	-	-	-	-	-	L	-	S	A	P	-	-	-	-	-	-	-	-	-
	G. acutus	-	-	-	-	-	-	-	-	-	-	-	-	-	-	-	-	-	-	L	-	-	A	-	-	-	-	-	-	-	-	H	-
	D. zarudnyi	-	-	-	-	-	-	-	-	-	-	-	-	-	-	-	-	-	-	L	-	-	A	P	-	-	-	-	-	-	-	-	-
	A. schmidti	-	-	-	-	-	-	-	-	-	-	-	-	-	-	-	-	-	-	L	-	-	A	-	-	-	-	-	-	-	-	-	-
	B. tridactylus	-	-	-	-	-	-	-	-	-	-	-	-	-	-	-	-	-	-	L	-	-	A	P	-	-	-	-	-	-	-	H	-
	B. canaliculatus	-	-	-	-	-	-	-	-	-	-	-	-	-	-	-	-	-	-	L	-	-	A	P	-	-	-	-	-	-	-	H	-
	B. biporus	-	-	-	-	-	-	-	-	-	-	-	-	-	-	-	-	-	-	L	-	-	A	P	-	-	-	-	-	I	-	H	-
	A. carolinensis	-	-	-	-	-	-	-	-	-	-	-	-	-	-	-	-	-	-	-	-	-	-	-	-	-	-	-	-	-	-	-	-
	O. attenuatus	-	-	-	-	-	-	-	-	-	-	-	-	-	-	-	-	-	-	L	-	-	-	-	-	-	-	-	-	-	-	-	-
	V. salvator	-	-	-	-	-	-	-	-	-	-	-	-	-	-	-	-	-	-	L	-	-	-	-	-	-	-	-	-	-	-	F	-

Supplementary Table S8. Continued.

		10	13	14	15	16	17	18	80	81	84	85	90	92	97	99	100	102	107	109	111	114	116	141	143	145	147	148	150	155	158	502	504
**Tuatara**	*S. punctatus*	-	-	-	-	-	-	-	-	-	-	-	-	-	-	-	-	-	-	-	-	T	A	P	-	-	-	-	-	-	-	L	-
**Crocodilians**	*C. crocodilus*	-	-	-	-	-	-	-	-	-	-	-	-	-	-	-	-	-	-	-	-	-	-	P	-	-	-	-	-	-	-	-	-
	A. sinensis	-	-	-	-	-	-	-	-	-	-	-	-	-	-	-	-	-	-	-	-	S	A	P	-	-	-	-	-	-	-	H	-
	A. mississippiensis	-	-	-	-	-	-	-	-	-	-	-	-	-	-	-	-	-	-	-	-	S	A	P	-	-	-	-	-	-	-	H	-
	G. gangeticus	-	-	-	-	-	-	-	-	-	-	-	-	-	-	-	-	-	-	-	-	F	A	P	-	-	-	-	-	-	-	H	-
	C. moreletii	-	-	-	-	-	-	-	-	-	-	-	-	-	-	-	-	-	-	-	-	F	A	P	-	-	-	-	-	-	-	-	-
**Turtles**	*D. subplana*	-	-	-	-	-	-	-	-	-	-	-	-	-	-	-	-	-	-	L	-	T	-	-	-	-	-	-	-	-	-	-	-
	P. subrufa	-	-	-	-	-	-	-	-	-	-	-	-	-	L	-	-	-	-	L	-	-	-	-	-	-	-	-	-	A	-	N	-
	C. picta	-	-	-	-	-	-	-	-	-	-	M	-	-	-	-	-	-	-	L	-	-	-	-	-	-	-	-	-	-	-	H	-
	C. mydas	-	-	-	-	-	-	-	-	-	-	-	-	-	-	-	-	-	-	L	-	-	-	-	-	-	-	-	-	-	-	-	-
**Birds**	*T. major*	-	-	-	-	-	-	-	-	-	-	-	-	-	-	-	-	-	-	-	-	-	-	P	-	-	-	-	-	-	-	H	-
	S. sharpei	-	-	-	-	-	-	-	-	-	-	-	-	-	-	-	-	-	-	-	-	-	-	-	-	-	-	-	-	-	-	-	-
	C. frugilegus	-	-	-	-	-	-	-	-	-	-	-	-	-	-	-	-	-	-	-	-	-	-	-	-	-	-	-	-	I	-	F	-
	V. chalybeata	-	-	-	-	-	-	-	-	-	-	-	-	-	-	-	-	-	-	-	-	-	-	-	-	-	-	-	-	I	-	F	-
	B. buteo	-	-	-	-	-	-	-	-	-	-	-	-	-	-	-	-	-	-	-	-	-	-	-	-	-	-	-	-	-	-	-	-
	F. peregrinus	-	-	-	-	-	-	-	-	-	-	-	-	-	-	-	-	-	-	-	-	-	-	-	-	-	-	-	-	-	-	-	-
	D. novaehollandiae	-	-	-	-	-	-	-	-	-	-	-	-	-	-	-	-	-	-	-	-	-	-	-	-	-	-	-	-	-	-	H	-
	S. camelus	-	-	-	-	-	-	-	-	-	-	-	-	-	-	-	-	-	-	-	-	-	-	-	-	-	-	-	-	-	-	H	-
	A. haastii	-	-	-	-	-	-	-	-	-	-	-	-	-	-	-	-	-	-	-	-	-	-	-	-	-	-	-	-	I	-	H	-
	R. americana	-	-	-	-	-	-	-	-	-	-	-	-	-	-	-	-	-	-	-	-	-	-	-	-	-	-	-	-	-	-	H	-
	G. gallus	-	-	-	-	-	-	-	-	-	-	-	-	-	-	-	-	-	-	-	-	-	-	-	-	-	-	-	H	-	-	-	-
	C. ciconia	-	-	-	-	-	-	-	-	-	-	-	-	-	-	-	-	-	-	-	-	-	-	-	-	-	-	-	-	-	-	-	-
	C. boyciana	-	-	-	-	-	-	-	-	-	-	-	-	-	-	-	-	-	-	-	-	-	-	-	-	-	-	-	-	-	-	-	-
**Amphibians**	*M. luschani*	-	-	-	-	-	-	-	-	-	-	-	-	-	-	-	-	-	-	-	-	-	-	-	-	-	-	-	-	-	-	-	-
	X. laevis	-	-	-	-	-	-	-	-	-	-	-	-	-	-	-	-	-	-	-	-	-	-	-	-	-	-	-	-	I	-	-	-
