# Supplementary material for: Adaptive Evolution and Functional Redesign of Core Metabolic Proteins in Snakes
Source: PLoS One. 2008 May 21;3(5):e2201. doi: 10.1371/journal.pone.0002201 (PMC2376058; doi:10.1371/journal.pone.0002201)
Supplement: Table S9 — Conservation of residues surrounding channel H across the 65 taxon dataset used. (0.13 MB PDF) [file pone.0002201.s027.pdf]

Supplementary Table S9. Conservation of residues surrounding channel H across the 65 taxon dataset used. The symbol "-" refers to amino acids that are the same as the reference sequence of *Bos taurus* at the corresponding site.

		37	39	381	383	406	408	412	414	420	423	425	427	429	442	444	450	452	453	455	460	462
**Primates**	*B. taurus*	I	A	L	-	N	T	I	F	G	M	F	P	H	D	P	W	T	I	S	I	L
	H. lar	-	-	-	-	D	-	-	-	-	L	-	-	-	-	-	-	I	L	-	-	-
	L. catta	-	-	-	-	D	-	-	-	-	-	-	-	-	-	-	-	-	V	-	-	-
	N. coucang	-	-	-	-	D	-	-	-	-	L	-	-	-	-	-	-	S	-	-	-	-
	T. bancanus	-	-	-	-	H	-	T	-	-	L	-	-	-	-	-	-	-	-	-	-	-
	G. gorilla	-	-	-	-	D	-	-	-	-	L	-	-	-	-	-	-	I	L	-	-	-
	H. sapiens	-	-	-	-	D	-	-	-	-	L	-	-	-	-	-	-	I	L	-	-	-
	P. hamadryas	-	-	-	-	D	-	A	-	-	L	-	-	-	-	-	-	I	L	-	-	-
	C. albifrons	-	-	-	-	D	-	-	-	-	-	-	-	-	-	-	-	I	-	-	-	-
	M. sylvanus	-	-	-	-	-	-	V	-	-	L	-	-	-	-	-	-	I	L	-	-	-
	P. pygmaeus	-	-	-	-	-	-	-	-	-	L	-	-	-	-	-	-	I	L	-	-	-
	P. paniscus	-	-	-	-	D	-	-	-	-	L	-	-	-	-	-	-	V	L	-	-	-
**Snakes**	*A. piscivorus*	M	M	-	-	-	-	T	-	-	-	-	-	-	-	-	-	-	-	-	-	-
	P. slowinskii	M	M	-	-	-	-	T	-	-	-	-	-	-	-	-	-	-	L	-	-	M
	D. semicarinatus	M	M	-	-	-	-	T	-	-	-	-	-	-	-	-	-	-	M	-	-	M
	B. constrictor	M	M	-	-	-	-	T	-	-	-	-	-	-	-	-	-	-	-	-	-	-
	P. regius	M	M	-	-	-	-	T	-	-	-	-	-	-	-	-	-	-	-	-	-	-
	A. granulatus	M	M	-	-	-	-	A	-	-	L	-	-	-	-	-	-	-	-	-	-	M
	C. ruffus	M	M	-	-	-	-	T	-	-	-	-	-	-	-	-	-	-	V	-	-	-
	O. okinavensis	M	M	-	-	-	-	T	-	-	-	-	-	-	-	-	-	-	-	-	-	-
	X. unicolor	M	M	-	-	-	-	T	-	-	-	-	-	-	-	-	-	-	V	-	-	-
	T. reticulatus	V	M	-	-	-	-	-	-	-	-	-	-	-	-	-	-	V	L	-	-	M
	L. dulcis	-	M	-	-	H	-	A	-	-	I	-	-	-	-	-	-	-	T	-	-	T
**Lizards**	*I. iguana*	-	-	-	-	H	-	-	-	-	-	-	-	-	-	-	-	-	-	-	-	-
	E. egregius	-	-	-	-	H	-	-	-	-	L	-	-	-	-	-	-	-	M	-	-	-
	S. occidentalis	-	-	-	-	H	-	-	-	-	-	-	-	-	-	-	-	-	V	-	-	-
	C. warreni	-	T	-	-	-	A	-	-	-	L	-	-	-	-	-	-	-	V	-	-	-
	A. graminea	-	-	-	-	H	-	-	-	-	-	-	-	-	-	-	-	L	-	-	-	I
	S. crocodilurus	-	-	-	-	H	A	-	-	-	-	-	-	-	-	-	-	A	-	-	-	-
	V. komodoensis	-	-	-	-	H	L	-	-	-	-	-	-	-	-	-	-	A	L	-	-	-
	R. floridana	-	-	-	-	H	G	-	-	-	L	-	-	-	-	-	-	-	-	-	-	-
	G. acutus	V	-	-	-	S	A	M	-	-	L	-	-	-	-	-	-	-	V	-	-	T
	D. zarudnyi	-	-	-	-	-	-	M	-	-	-	-	-	-	-	-	-	-	V	-	-	T
	A. schmidti	-	-	-	-	-	M	T	-	-	L	-	-	-	-	-	-	-	L	-	-	T
	B. tridactylus	-	-	-	-	-	A	L	-	-	-	-	-	-	-	-	-	-	-	-	-	T
	B. canaliculatus	V	-	-	-	-	-	L	-	-	-	-	-	-	-	-	-	-	-	-	-	T
	B. biporus	-	-	-	-	S	-	L	-	-	-	-	-	-	-	-	-	-	V	-	V	T
	A.carolinensis	-	-	-	-	H	A	A	-	-	L	-	-	-	-	-	-	A	-	-	-	-
	O. attenuatus	-	-	-	-	H	-	V	-	-	-	-	-	-	-	-	-	I	-	-	-	I
	V. salvator	-	-	-	-	H	L	-	-	-	-	-	-	-	-	-	-	-	L	-	-	-

Supplementary Table S9. Continued.

		37	39	381	383	406	408	412	414	420	423	425	427	429	442	444	450	452	453	455	460	462
**Tuatara**	*S. punctatus*	-	G	-	-	H	-	V	-	-	L	-	-	-	-	-	-	-	L	-	-	-
**Crocodilians**	*C. crocodilus*	-	T	-	-	H	-	-	-	-	F	-	-	-	-	-	-	L	T	-	-	-
	A. sinensis	-	T	-	-	T	H	-	-	-	F	-	-	-	-	-	-	L	L	-	-	M
	A. mississippiensis	-	T	-	-	H	-	-	-	-	F	-	-	-	-	-	-	L	T	-	-	M
	G. gangeticus	-	T	-	-	H	M	T	-	-	L	-	-	-	-	-	-	M	-	-	-	M
	C. moreletii	-	T	-	-	H	-	-	-	-	L	-	-	-	-	-	-	M	-	-	-	M
**Turtles**	*D. subplana*	-	-	-	-	H	-	V	-	-	-	-	-	-	-	-	-	-	-	-	-	-
	P. subrufa	-	T	-	-	H	-	-	-	-	L	-	-	-	-	-	-	S	-	-	-	-
	C. picta	-	-	-	-	H	-	V	-	-	-	-	-	-	-	-	-	S	-	-	-	-
	C. mydas	-	-	-	-	H	-	V	-	-	-	-	-	-	-	-	-	S	-	-	-	M
**Birds**	*T. major*	-	-	-	-	H	-	A	-	-	L	-	-	-	-	-	-	-	V	-	-	M
	S. sharpei	-	-	-	-	H	-	A	-	-	L	-	-	-	-	-	-	-	L	-	-	M
	C. frugilegus	-	-	-	-	H	-	-	-	-	L	-	-	-	-	-	-	-	-	-	-	-
	V. chalybeata	-	-	-	-	H	-	T	-	-	L	-	-	-	-	-	-	-	-	-	-	-
	B. buteo	-	-	-	-	H	-	A	-	-	L	-	-	-	-	-	-	-	L	-	-	M
	F. peregrinus	-	T	-	-	H	-	T	-	-	L	-	-	-	-	-	-	-	L	-	-	M
	D. novaehollandiae	-	-	-	-	H	-	A	-	-	L	-	-	-	-	-	-	-	L	-	-	M
	S. camelus	-	-	-	-	H	-	A	-	-	L	-	-	-	-	-	-	-	M	-	-	M
	A. haastii	-	-	-	-	H	-	A	-	-	L	-	-	-	-	-	-	-	M	-	-	M
	R. americana	-	-	-	-	H	-	A	-	-	L	-	-	-	-	-	-	-	M	-	-	M
	G. gallus	-	-	-	-	H	S	A	-	-	L	-	-	-	-	-	-	-	L	-	-	M
	C. ciconia	-	-	-	-	H	-	T	-	-	L	-	-	-	-	-	-	-	M	-	-	M
	C. boyciana	-	-	-	-	H	-	T	-	-	L	-	-	-	-	-	-	-	M	-	-	M
**Amphibians**	*M. luschani*	-	-	-	-	H	V	-	-	-	L	-	-	-	-	-	-	-	-	-	-	-
	X. laevis	-	-	-	-	H	-	-	-	-	L	-	-	-	-	-	-	-	V	-	-	-
