# Supplementary material for: Adaptive Evolution and Functional Redesign of Core Metabolic Proteins in Snakes
Source: PLoS One. 2008 May 21;3(5):e2201. doi: 10.1371/journal.pone.0002201 (PMC2376058; doi:10.1371/journal.pone.0002201)
Supplement: Table S10 — Conservation of residues surrounding channel K across the 65 taxon dataset used. (0.13 MB PDF) [file pone.0002201.s028.pdf]

Supplementary Table S10. Conservation of residues surrounding channel K across the 65 taxon dataset used. The symbol "-" refers to amino acids that are the same as the reference sequence of *Bos taurus* at the corresponding site.

		182	183	239	241	243	245	254	257	264	266	290	292	315	317	318	320	367	369	488	492
**Primates**	*B. taurus*	P	L	G	P	V	I	I	I	K	E	H	M	P	G	V	V	L	D	T	L
	H. lar	-	-	-	-	-	-	-	-	-	-	-	-	-	-	-	-	-	-	P	-
	L. catta	-	-	-	-	-	-	-	-	-	-	-	-	-	-	-	-	-	-	-	-
	N. coucang	-	-	-	-	-	-	-	-	-	-	-	-	-	-	-	-	-	-	-	-
	T. bancanus	-	-	-	-	-	-	-	-	-	-	-	-	-	-	-	-	-	-	P	-
	G. gorilla	-	-	-	-	-	-	-	-	-	-	-	-	-	-	-	-	-	-	P	-
	H. sapiens	-	-	-	-	-	-	-	-	-	-	-	-	-	-	-	-	-	-	P	-
	P. hamadryas	-	-	-	-	-	-	-	-	-	-	-	-	-	-	-	-	-	-	P	-
	C. albifrons	-	-	-	-	-	-	-	-	-	-	-	-	-	-	-	-	-	-	M	-
	M. sylvanus	-	-	-	-	-	-	V	-	-	-	-	-	-	-	-	-	-	-	P	-
	P. pygmaeus	-	-	-	-	-	-	-	-	-	-	-	-	-	-	-	-	-	-	P	-
	P. paniscus	-	-	-	-	-	-	-	-	-	-	-	-	-	-	-	-	-	-	P	-
**Snakes**	*A. piscivorus*	-	-	-	-	-	-	-	-	-	N	-	-	-	-	I	-	-	-	K	I
	P. slowinskii	-	-	-	-	-	-	V	-	-	N	-	-	-	-	I	-	-	-	K	V
	D. semicarinatus	-	-	-	-	-	-	-	-	-	N	-	-	-	-	I	-	-	-	K	V
	B. constrictor	-	-	-	-	-	-	-	-	-	N	-	-	-	-	I	-	-	-	K	V
	P. regius	-	-	-	-	-	-	-	-	-	N	-	-	-	-	I	-	-	-	K	V
	A. granulatus	-	-	-	-	-	-	-	-	-	N	-	-	-	-	I	-	-	-	K	V
	C. ruffus	-	-	-	-	-	-	V	-	-	N	-	-	-	-	I	-	-	-	K	V
	O. okinavensis	-	-	-	-	-	-	-	-	-	N	-	-	-	-	I	-	-	-	K	I
	X. unicolor	-	-	-	-	-	-	-	-	-	N	-	-	-	-	I	-	-	-	K	V
	T. reticulatus	-	-	-	-	-	-	V	-	-	-	-	-	-	-	I	-	-	-	M	I
	L. dulcis	-	-	-	-	-	-	-	V	-	-	-	-	-	-	I	-	-	-	-	T
**Lizards**	*I. iguana*	-	-	-	-	-	-	-	-	-	-	-	-	-	-	-	-	-	-	-	-
	E. egregius	-	-	-	-	-	-	-	-	-	-	-	-	-	-	-	-	-	-	-	-
	S. occidentalis	-	-	-	-	-	-	-	-	-	-	-	-	-	-	-	-	-	-	-	I
	C. warreni	-	-	-	-	-	-	-	-	-	-	-	-	-	-	-	-	-	-	-	M
	A. graminea	-	-	-	-	-	-	-	-	-	-	-	-	-	-	-	-	-	-	V	-
	S. crocodilurus	-	-	-	-	-	-	-	V	-	-	-	-	-	-	-	-	-	-	-	-
	V. komodoensis	-	-	-	-	-	-	-	-	-	-	-	-	-	-	I	-	-	-	P	-
	R. floridana	-	-	-	-	-	-	-	-	-	-	-	-	-	-	-	-	-	-	A	-
	G. acutus	-	-	-	-	-	-	-	-	-	-	-	-	-	-	I	-	-	-	P	-
	D. zarudnyi	-	-	-	-	-	-	-	-	-	-	-	-	-	-	I	-	-	-	-	-
	A. schmidti	-	-	-	-	-	-	-	-	-	-	-	-	-	-	I	-	-	-	-	-
	B. tridactylus	-	-	-	-	-	-	-	-	-	-	-	-	-	-	I	-	-	-	-	-
	B. canaliculatus	-	-	-	-	-	-	-	-	-	-	-	-	-	-	I	-	-	-	-	-
	B. biporus	-	-	-	-	-	-	-	-	-	-	-	-	-	-	I	-	-	-	-	-
	A.carolinensis	-	-	-	-	-	-	-	-	-	-	-	-	-	-	-	-	-	-	A	-
	O. attenuatus	-	-	-	-	-	-	-	-	-	-	-	-	-	-	-	-	-	-	-	-
	V. salvator	-	-	-	-	-	-	-	-	-	-	-	-	-	-	I	-	-	-	P	-

Supplementary Table S10. Continued.

		**182**	**183**	**239**	**241**	**243**	**245**	**254**	**257**	**264**	**266**	**290**	**292**	**315**	**317**	**318**	**320**	**367**	**369**	**488**	**492**
**Tuatara**	*S. punctatus*	-	-	-	-	-	-	-	V	-	-	-	-	-	-	I	-	-	-	N	S
**Crocodilians**	*C. crocodilus*	-	-	-	-	-	-	-	V	-	-	-	-	-	-	-	-	-	-	-	I
	A. sinensis	-	-	-	-	-	-	-	V	-	-	-	-	-	-	-	-	-	-	-	I
	A. mississippiensis	-	-	-	-	-	-	-	V	-	-	-	-	-	-	-	-	-	-	-	I
	G. gangeticus	-	-	-	-	-	-	-	V	-	-	-	-	-	-	-	-	-	-	A	I
	C. moreletii	-	-	-	-	-	-	-	V	-	-	-	-	-	-	-	-	-	-	A	V
**Turtles**	*D. subplana*	-	-	-	-	-	-	-	V	-	-	-	-	-	-	-	-	-	-	-	V
	P. subrufa	-	-	-	-	-	-	-	-	-	-	-	-	-	-	-	-	-	-	M	V
	C. picta	-	-	-	-	-	-	-	V	-	-	-	-	-	-	-	-	-	-	-	V
	C. mydas	-	-	-	-	-	-	-	-	-	-	-	-	-	-	-	-	-	-	-	V
**Birds**	*T. major*	-	-	-	-	-	-	-	V	-	-	-	-	-	-	I	-	-	-	-	I
	S. sharpei	-	-	-	-	-	-	-	V	-	-	-	-	-	-	I	-	-	-	-	I
	C. frugilegus	-	-	-	-	-	-	-	V	-	-	-	-	-	R	I	-	-	-	I	V
	V. chalybeata	-	-	-	-	-	-	-	V	-	-	-	-	-	-	I	-	-	-	-	I
	B. buteo	-	-	-	-	-	-	-	V	-	-	-	-	-	-	I	-	-	-	-	V
	F. peregrinus	-	-	-	-	-	-	-	V	-	-	-	-	-	-	I	-	-	-	-	I
	D. novaehollandiae	-	-	-	-	-	-	-	V	-	-	-	-	-	-	I	-	-	-	I	I
	S. camelus	-	-	-	-	-	-	-	V	-	-	-	-	-	-	I	-	-	-	I	I
	A. haastii	-	-	-	-	-	-	-	V	-	-	-	-	-	-	I	-	-	-	-	I
	R. americana	-	-	-	-	-	-	-	V	-	-	-	-	-	-	I	-	-	-	I	I
	G. gallus	-	-	-	-	-	-	-	V	-	-	-	-	-	-	I	-	-	-	-	I
	C. ciconia	-	-	-	-	-	-	-	V	-	-	-	-	-	-	I	-	-	-	-	V
	C. boyciana	-	-	-	-	-	-	-	V	-	-	-	-	-	-	I	-	-	-	-	V
**Amphibians**	*M. luschani*	-	-	-	-	-	-	-	-	-	-	-	-	-	-	-	-	-	-	-	I
	X. laevis	-	-	-	-	-	-	-	-	-	-	-	-	-	-	-	-	-	-	-	-
