# Supplementary material for: Adaptive Evolution and Functional Redesign of Core Metabolic Proteins in Snakes
Source: PLoS One. 2008 May 21;3(5):e2201. doi: 10.1371/journal.pone.0002201 (PMC2376058; doi:10.1371/journal.pone.0002201)
Supplement: Text S1 — Detailed results of branch-site analyses of positive selection. (0.05 MB PDF) [file pone.0002201.s029.pdf]

Results of Branch-Site, Site Specific Selection Analyses

For analyses of the branch leading to all snakes, the ln likelihood value of M2a(null) was -433795.05 versus that for M2a(alt.) at -433376.37. The likelihood ratio test favors the alternative model with positive selection allowed on the branch leading to all snakes at $P \ll 0.001$. The same test for positive selection at sites along the branch leading to the Alethinophidia yielded ln likelihood values of M2a(null) = -433795.05 and M2a(alt) = -433465.10. The likelihood ratio test also strongly supported the alternative model allowing some sites to be under positive selection along the Alethinophidian branch at $P \ll 0.001$. Based on these two analyses, the estimates of sites that are under positive selection along these two branches are provided in Table 1 (all proteins) and Table S3 for COI, based on Bayes empirical Bayes posterior probability support for each site.
